# Chemoenzymatic Late‐Stage Modifications Enable Downstream Click‐Mediated Fluorescent Tagging of Peptides

**DOI:** 10.1002/anie.202215979

**Published:** 2023-03-10

**Authors:** Alessandro Colombano, Luca Dalponte, Sergio Dall'Angelo, Claudia Clemente, Mohannad Idress, Ahmad Ghazal, Wael E. Houssen

**Affiliations:** ^1^ Institute of Medical Sciences University of Aberdeen Ashgrove Road West Aberdeen AB25 2ZD UK; ^2^ Department of Chemistry University of Aberdeen Aberdeen AB24 3UE UK; ^3^ Abzena, Babraham Research Campus Cambridge UK

**Keywords:** Cyanobactins, Cyclic Peptides, Late-Stage Modification, Prenyltransferases, RiPPs

## Abstract

Aromatic prenyltransferases from cyanobactin biosynthetic pathways catalyse the chemoselective and regioselective intramolecular transfer of prenyl/geranyl groups from isoprene donors to an electron‐rich position in these macrocyclic and linear peptides. These enzymes often demonstrate relaxed substrate specificity and are considered useful biocatalysts for structural diversification of peptides. Herein, we assess the isoprene donor specificity of the N1‐tryptophan prenyltransferase AcyF from the anacyclamide A8P pathway using a library of 22 synthetic alkyl pyrophosphate analogues, of which many display reactive groups that are amenable to additional functionalization. We further used AcyF to introduce a reactive moiety into a tryptophan‐containing cyclic peptide and subsequently used click chemistry to fluorescently label the enzymatically modified peptide. This chemoenzymatic strategy allows late‐stage modification of peptides and is useful for many applications.

## Graphical abstract

The promiscuity of N1‐tryptophan prenyltransferase AcyF was screened with 22 unnatural pyrophosphates, which exhibited reactive groups suitable for copper‐catalyzed azide‐alkyne cycloaddition, metathesis, and inverse‐electron‐demand Diels‐Alder (IEDDA) reactions. A 10‐mer tryptophan‐containing macrocyclic peptide was tailored by AcyF, and the resulting modified peptide was successfully labelled with a tetrazine–fluorescein conjugate by IEDDA.

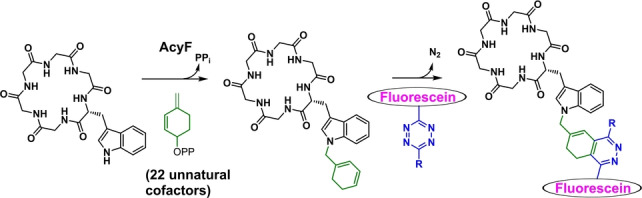

## Introduction

Cyanobactins are a family of cyanobacterial modified linear and macrocyclic peptides that are produced through post‐translational modification of ribosomally encoded precursor peptides.[^1^, ^2^, ^3^] These post‐translational modifications include heterocyclization to generate thiazoline, oxazoline and methyloxazoline,[^4^, ^5^, ^6^, ^7^] oxidation of azolines to the corresponding azoles,^[8]^ N‐methylation of histidine,[^9^, ^10^] N‐to‐C‐macrocyclization[^11^, ^12^, ^13^, ^14^] and forward and reverse prenylation and geranylation.[^15^, ^16^, ^17^, ^18^, ^19^, ^20^, ^21^, ^22^, ^23^, ^24^, ^25^, ^26^, ^27^, ^28^] Cyanobactin prenyltransferases belong to the aromatic prenyltransferases ABBA superfamily, which are named after the αββα succession of secondary structure elements and are characterized by having a β‐barrel core that is surrounded by a ring of solvent‐exposed α‐helices.[^16^, ^29^, ^30^, ^31^, ^32^] The central barrel forms a catalytic chamber where substrate binding and catalysis occurs.[^19^, ^29^]

Cyanobactin prenyltransferases have recently received considerable interest as they can incorporate C_5_ prenyl and C_10_ geranyl groups with high residue‐ and regiospecificity in linear and cyclic peptides. Consequently, they enhance the structural diversification of peptide libraries and more importantly increase the lipophilicity of the peptides and thus enhance their ATP‐independent cell permeability.[^33^, ^34^] Peptides, especially macrocyclic peptides, hold great therapeutic potential, and their poor cellular permeability and negligible oral bioavailability are considered the main hurdle for their development as therapeutics.[^35^, ^36^, ^37^, ^38^] Cyanobactin prenyltransferases have broad substrate promiscuity as they only need a small motif within peptide substrates for recognition, hence they represent robust tools for synthetic biology.^[20]^

Several cyanobactin prenyltransferases have been identified to date, and they catalyse the O‐prenylation of Tyr, Thr and Ser in the forward or reverse orientation,[^19^, ^20^] the forward prenylation of Trp indole at C3 and N1,[^21^, ^22^, ^23^] prenylation at the N‐ or C‐terminus of linear peptides,[^24^, ^25^] forward prenylation of the Arg guanidinium N^ω^ position,[^17^, ^26^] O‐geranylation on Tyr^[16]^ and C2‐geranylation of histidine.^[18]^ Cyanobactins containing reverse O‐prenylated Tyr have been shown to undergo a Claisen rearrangement to yield forward C‐prenylated Tyr.^[19]^

However, to date, cyanobactin prenyltransferases have shown strict specificity for the isoprene donor. For instance, prenyltransferases that catalyse the transfer of the C_5_ dimethylallyl moiety from dimethylallyl pyrophosphate (**1**, DMAPP), cannot transfer larger isoprenes such as (C_10_) geranyl or (C_15_) farnesyl units from geranyl pyrophosphate (GPP) or farnesyl pyrophosphate (FPP), respectively. Crystal‐structure comparison between PirF, a Tyr O‐geranyltransferase from the piricyclamide pathway, and PagF, a Tyr forward O‐prenyltransferase from the prenylagaramide pathway, revealed a small‐to‐large single amino acid substitution in the vicinity of the isoprene‐binding pocket which restricts the accommodation of the bulkier GPP in PagF.^[16]^ Mutation of this single amino acid completely switched the donor specificity from a C_5_ prenyl‐ to a C_10_ geranyltransferase.^[16]^

Interestingly, the tolerance of few non‐cyanobactin highly promiscuous aromatic prenyltransferases towards unnatural allylic and benzylic alkyl donors has been studied.[^39^, ^40^, ^41^] These include SirD,^[42]^ FgaPT2^[30]^ and CdpNT.^[43]^ These enzymes have shown high tolerance for diverse non‐native alkyl donors, although this screening was only done using amino acid substrates including l‐Tyr in the case of SirD and l‐Trp in case of FgaPT2, in addition to few amino acid derived substrates, such as indolocarbazole analogues.[^39^, ^40^, ^41^] These substrates were chosen to mimic the natural substrates of the enzymes. In a recent study, the Elshahawi group^[44]^ demonstrated that CdpNPT can incorporate a reactive hydroxy‐bearing allyl moiety into small Trp‐containing dipeptides. Four different products were obtained, in which the allyl moiety was installed at different positions on the indole ring.^[44]^ Alterations of prenyltransferase regiospecificity have also been observed when using unnatural cofactors.[^39^, ^40^, ^41^, ^44^]

The presence of a specifically reacting or bio‐orthogonal group on the side chain of a cyclic peptide is a highly desirable feature. Such a group can be employed to study and/or improve binding affinity or activity,^[45]^ to attach a fluorescent probe to study permeability,^[46]^ to connect additional building blocks to enhance activity or improve drug delivery,[^47^, ^48^] or to facilitate the target identification of the compound. However, these approaches, require the incorporation of the desired group from the beginning.

**Figure 1:**
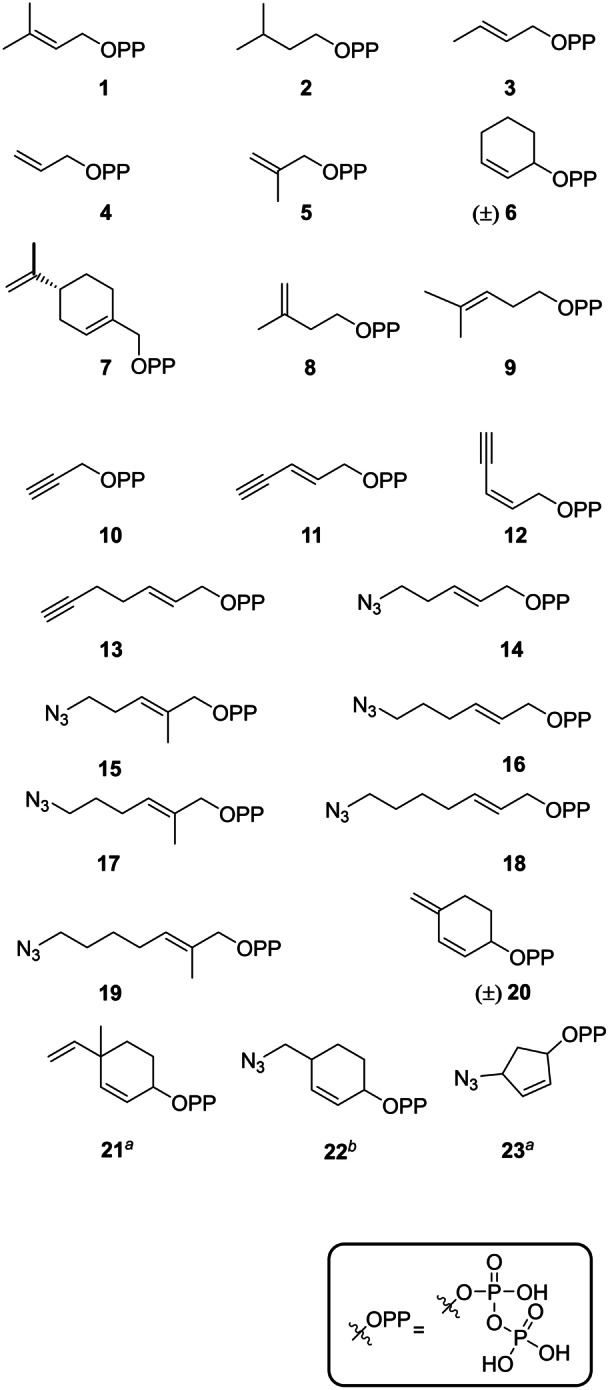
Library of synthetic pyrophosphates used in this study. [a] Compound was isolated as a 1 : 1 mixture of diastereoisomers. [b] Compound was isolated as a 1:0.4 mixture of diastereoisomers.

In this study, we synthesised new allyl cofactors, many of which bear highly reactive groups amenable to further functionalization using click and metathesis reactions (Figure 1). We tested the tolerance of the N1‐tryptophan prenyltransferase, AcyF from the anacyclamide A8P pathway^[23]^ for these cofactors using a 10mer macrocyclic Trp‐containing cyclic peptide. Furthermore, we demonstrated, for the first time, the use of click chemistry to fluorescently label the enzymatically modified peptide.

## Results and Discussion

The synthetic alkyl pyrophosphates used in this study are shown in Figure 1. We started our investigation with dimethylallyl pyrophosphate (**1**), which is the natural AcyF cofactor, and we explored the tolerance of the enzyme towards the corresponding saturated analogue **2**. Compounds **3**–**5** were selected to assess the importance of the presence and/or position of DMAPP methyl groups. Alkyl donors **6** and **7** were designed as rigid analogues of DMAPP, and **8** and **9** served as probes to test the impact of allyl moiety homologation. Compounds **10**–**13** were designed to test the effect of DMAPP alkene replacement or conjugation/homologation with terminal alkynes, as well as tolerance of the enzyme towards lengthy hydrocarbon chains. The design of azides **14**–**19** followed a similar strategy, whereas the use of **20**–**23** was inspired by the successful incorporation of **6** (see below).

Importantly, the incorporation of compounds **10**–**19**, **22** or **23** on cyclic peptides could readily enable additional functionalization by copper‐catalysed azide–alkyne cycloaddition (CuAAC).^[49]^ Moreover, **1**, **3**–**5**, **7**–**9** and **21** display terminal olefin functionalities, which could be exploited in olefin metathesis reactions.[^50^, ^51^] Finally, peptides modified with compounds such as **4**, **6**, **20** or **21** could, in principle, be submitted to inverse‐electron‐demand Diels‐Alder (IEDDA) reactions.^[52]^

The pyrophosphate library was employed as tool to explore AcyF tolerance towards the incorporation of unnatural alkyl donors on cyclic peptides. For this purpose, the library was screened in enzymatic reaction assays consisting of AcyF (20 μM), MgCl_2_ (12 mM), pyrophosphate alkyl donor **1**–**23** (1 mM) and cyclo‐[TSQIWGSPVP] (**24**) (100 μM) as the alkyl acceptor in a buffer containing 150 mM NaCl, 10 mM HEPES (pH 7.5) and 3 mM TCEP. Reactions were incubated at 37 °C for 72 h and monitored by liquid chromatography–high resolution mass spectrometry (LC‐HRMS; Figures S2–S45).

The results showed that cofactors **1**, **3**, **6**, **8**, **20** and **22** were accepted by the enzyme. From these results, we noticed the following structure‐activity relationships.

Double‐bond elimination from DMAPP as in **2** or homologation of the allylic pyrophosphate moiety as in **9** led to no reaction, while elimination of the DMAPP methyl group as in **3** resulted in only trace amounts of desired product. Elimination of DMAPP methyl groups as in **4** or double‐bond replacement with a terminal alkyne as in cofactors **10**–**13** resulted in no reaction. A shift of the DMAPP double bond from the 2‐ to the 3‐position in **8** resulted in successful conversion. However, it should be noted that **1** and **8** could independently generate the same product. Additionally, **8** could isomerize to native substrate **1** under the assay conditions. Similarly, synthesis of **8** from the corresponding chloride/bromide affords isoprene instead, owing to a competing rearrangement.^[53]^ Unfortunately, none of the linear azides **14**–**19** were accepted, suggesting rather strict steric requirements in the active site of the enzyme.

Surprisingly, elimination of the methyl group at the 3‐position of DMAPP and constriction into a cyclohexene unit as in **6** led to successful alkylation. This finding prompted the design and synthesis of compounds **20**–**23**. Interestingly, diene **20** and azide **22** were accepted. Unexpectedly, reactions with **20** and **22** yielded a mixture of six and three products, respectively, whose exact mass was consistent with cofactor incorporation, but with different retention times, as determined by LC‐HRMS (Figures S38 and S42). In both cases, the formation of a major product was noticed, alongside multiple minor products. Moreover, products associated with **22** exhibited an exact mass which reflected loss of HN_3_.

These findings could be due to several factors. First, it has been shown that employment of unnatural cofactors can lead to variations in the alkylation regiospecificity of prenyltransferases, resulting in the formation of mixtures of alkylated regioisomers.[^44^, ^54^, ^55^, ^56^, ^57^, ^58^]

Moreover, compounds **20** and **22** contain one and two stereogenic carbon atoms, respectively. Therefore, the corresponding alkylated cyclic peptides could theoretically be formed as mixtures of diastereoisomers. Finally, the carbocationic intermediates[^30^, ^59^] involved in prenylation could rearrange before being intercepted by nucleophilic residues, leading to unexpected products. It is unclear whether the observed loss of HN_3_ was due to the compound ionization in the HRMS ion source^[60]^ or to a structural rearrangement upon enzymatic incorporation; such rearrangement could, in principle, lead to the same product obtained by reaction with **20**.

Having established that AcyF showed promiscuity towards the isoprene donor, our goal was to enzymatically install a cofactor‐derived moiety onto peptide **24** and then further functionalize the enzymatically modified peptide through click chemistry techniques. Even though AcyF has been reported as an N1‐tryptophan prenyltransferase,^[23]^ full structure elucidation of the putative peptide structure was deemed essential before any additional functionalization attempts.

**Figure 2:**
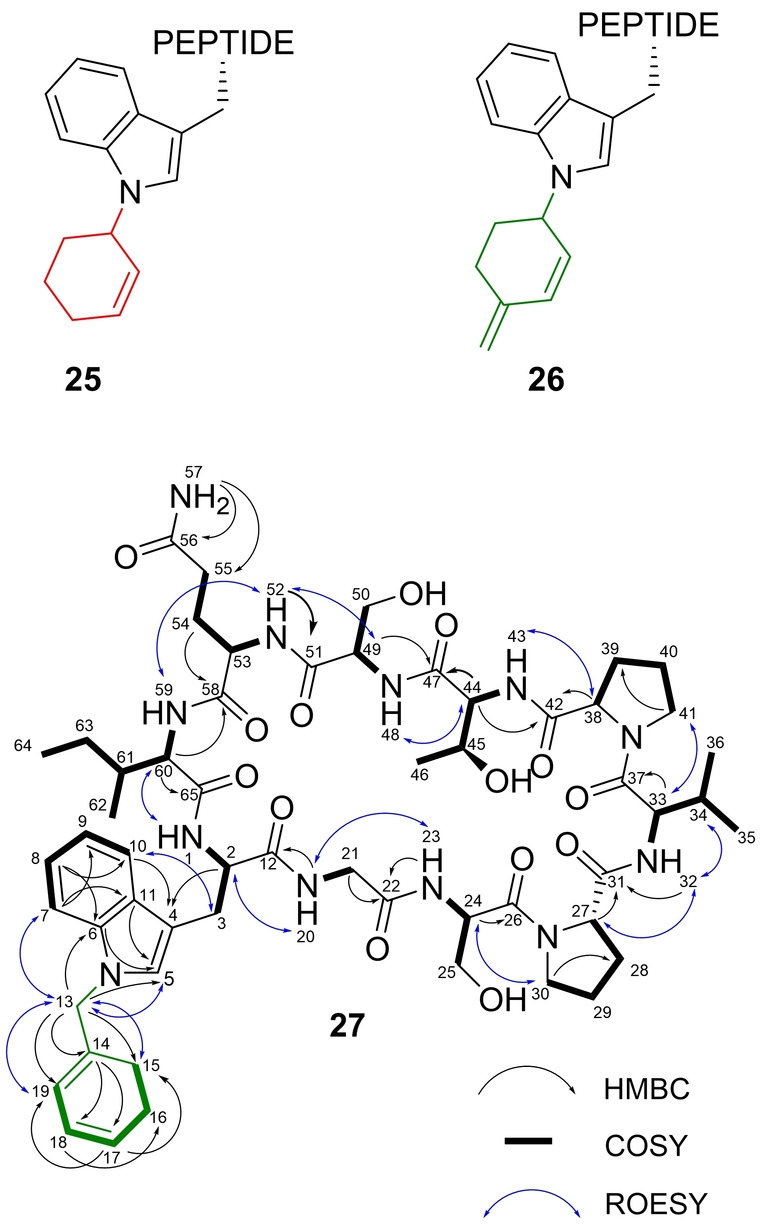
Top: Putative structure of products arising from the AcyF‐catalysed reaction between peptide **24** and alkyl donor **6** (red) and **20** (green), leading to compound **25** and **26**, respectively. Bottom: Key 2D NMR correlations of **27** in DMSO‐*d_6_*. The enzymatically added moiety is represented in green.

AcyF showed favourable turnover of compound **6**, which was efficiently appended onto **24** affording the corresponding modified peptide **25** (Figure 2, top). Compound **25** seemed a good candidate for our purpose. Therefore, the analytical enzymatic reaction protocol was scaled up, and the desired **25** was isolated by semipreparative HPLC. Unfortunately, the modified peptide showed suboptimal stability, owing to decomposition to corresponding unmodified **24** upon gentle heating at 37 °C in the rotary evaporator bath (Figure S46).

We subsequently turned our attention to the diene **20**. Different reaction conditions were tested to improve the reaction yield. It was found that reaction with 500 μM cyclic peptide for 24 h at 30 °C provided substrate conversion of 72 %±1.3 (mean±standard deviation, *n=*3). Conversions were estimated by integration of HPLC–UV chromatographic peaks (260 nm).

Steady‐state kinetic parameters of AcyF with **20** and **1** were calculated to understand how enzyme activity is compromised while using the non‐native analogue (Figure S47). For compound **20**, the *K*_m_ value was determined to be 0.44±0.03 mM, the *k*_cat_ value was 0.013±0.001 s^−1^ and the catalytic efficiency *k*_cat_/*K*_m_ was found to be 29.4±3.9 s^−1^ M^−1^. For **1**, the *K*_m_ value was calculated to be 0.25±0.01 mM, the *k*_cat_ value was 0.013±0.0008 s^−1^ and the catalytic efficiency *k*_cat_/*K*_m_ was determined to be 51.4±5.2 s^−1^ M^−1^. Our results show that AcyF affinity for **20** is almost two times lower than for **1**, while the *k*_cat_ value is the same for both cofactors, and the catalytic efficiency is lower for **20**.

Having optimized conditions, a large‐scale enzymatic reaction was carried out, and the major product **26** formed was purified (Figure 2, top and Figure S48).

No decomposition was observed upon concentration using a rotary evaporator at 37 °C. Isolation of the other isomers was unfortunately not possible, due to their minute quantities.

1D and 2D NMR data for putative **26** were acquired in DMSO‐*d_6_*, to unambiguously confirm its identity (Figures S49–S56). A total of 77 hydrogen and 56 carbon signals were identified, and 10 NH signals were identified by ^1^H‐^15^N HSQC, consistent with the proposed structure (three exchangeable OH resonances were not observed). Resonances of the main cyclic peptide backbone were assigned by means of COSY, HMBC and ROESY experiments.

A comparison of the data for **26** with NMR data for unmodified **24** previously published by our group^[23]^ revealed interesting findings (Figure S57): The broad ^1^H singlet around 10 ppm, which is typical for the tryptophan indole nitrogen atom, was absent in **26**, suggesting that alkylation had occurred on the indole nitrogen atom. Additionally, **24** showed essentially no ^1^H signals between 4.6 and 6 ppm (a broad singlet at 5.38 ppm likely belongs to an exchangeable OH). However, corresponding data for **26** showed proton resonances of three olefinic CH signals (*δ*_H_=5.83, 5.68, 5.59 ppm) as well as a CH_2_ (*δ*_H_=4.77, 4.73 ppm). These signals were absent in the spectrum of **24** and were most likely due to the enzymatically added moiety.

Surprisingly, the data seemed inconsistent with the putative structure of **26** (Figure 2, top). ^1^H NMR spectrum of **26** should theoretically show two olefinic CH signals around 6.0 ppm (doublet and doublet of doublets), one olefinic CH_2_ signal upfield around 5 ppm (broad singlet) and one CH signal in the same region as well, as can be inferred from ^1^H‐^13^C HSQC spectra of **20** (Figure S58). Mindful of these intriguing differences, we demonstrated that the structure of the major product obtained by AcyF‐catalysed tailoring of peptide **24** with alkyl donor **20** was in fact structure **27** (Figure 2, bottom, Table S3 and Figures S59–S61). Key signals include 13‐CH_2_ (*δ*_H_=4.77, 4.73 ppm, AB system), which displays no vicinal coupling, suggesting the C atom is sandwiched between quaternary carbon atoms or heteroatoms. Long‐range correlations showed its proximity to key tryptophan atoms 5‐CH, 6‐C and 7‐CH. The splitting pattern of 17‐CH into a doublet of triplets (*δ*_H_=5.68 ppm, *J*=9.5, 4.2 Hz) indicated a *cis* alkene relationship with 18‐CH (*δ*_H_=5.83 ppm, *J*=9.5, 5.0 Hz) and a vicinal relationship with allylic 16‐CH_2_ (*δ*_H_=2.08 ppm). Accordingly, 18‐CH is coupled with 17‐CH (*cis* relationship) and 19‐CH (*δ*_H_=5.59 ppm; vicinal relationship), while allylic 16‐CH_2_ showed coupling with 17‐CH and 15‐CH_2_. Finally, 14‐C, 15‐CH_2_ and 19‐CH showed long‐range correlations with 13‐CH_2_.

HRMS of **27** yielded a molecular ion [M+H]^+^ at *m*/*z* 1145.5999 (Figure S62), which is indicative of the molecular formula C_56_H_80_N_12_O_14_ (*δ*=0.8 ppm) and is consistent with incorporation of **20** onto cyclic peptide **24**. MS/MS fragmentation by pulsed‐Q dissociation provided further evidence for the alkylated tryptophan in the sequence: A clear series of *b‐*ions showed cleavage between SP and PT with sequential loss of aminoacidic fragments, which was consistent with the proposed structure (Figure S63).

Formation of **27** could be rationalized by means of resonance structures. It has been proposed that prenyltransferase alkylation occurs via carbocationic intermediates generated in the active site of the enzyme.^[30]^ Pyrophosphate cleavage from **20** would lead to highly stabilized dienyl carbocation **20‐I** (Figure S66). This intermediate allows two additional delocalization sites for the positive charge, leading to resonance structures **20‐II** and **20‐III**. Subsequent nitrogen nucleophilic attack on **20‐III** would lead to isolated compound **27**, while reaction with resonance structures **20‐I** and **20‐II** could explain, at least partially, the formation of the additional isomers identified in the crude reaction mixture. The observed preferential formation of **27** could be kinetically favoured due to faster nucleophilic attack on **20‐III**, which is less sterically hindered than the other resonance structures.

Many reports have shown the incorporation of unnatural pyrophosphate alkyl donors that can be, in principle, additionally functionalized.[^40^, ^41^, ^44^, ^57^, ^61^] However, to the best of our knowledge, there have been no reports demonstrating this. Therefore, we tried to achieve this proof of concept for the first time on compound **27**, by means of click chemistry, which is highly appealing for chemical biology applications.

Tetrazines are a commonly employed click chemistry tool; they have proved to be excellent reagents for inverse‐electron‐demand Diels‐Alder reactions (IEDDA), with applications in many fields, including radiochemistry, imaging, pretargeting and biorthogonal chemistry, among others.[^52^, ^62^] IEDDA reactions with tetrazines are normally conducted between electron‐poor tetrazines and strained/electron‐rich dienophiles. Moreover, the ability of tetrazines to undergo cycloaddition reactions with conjugated dienes as dienophiles has been known since 1959.^[63]^

However, pyrimidyl tetrazines have been shown to successfully react with unactivated dienophiles, such as terminal alkenes.[^64^, ^65^, ^66^]

Typically, the reaction yields dihydropyridazines, which can exist as several tautomeric isomers and are prone to oxidation to the corresponding pyridazines.^[52]^ This dualism is not an obstacle for imaging purposes.[^64^, ^65^, ^66^, ^67^]

**Figure 3:**
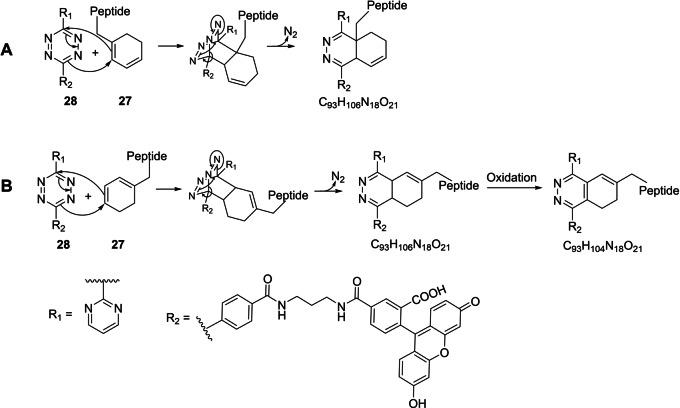
Proposed products obtained by cycloaddition of **27** with commercial tetrazine **28**. A), B) Different cycloaddition chemoselectivity leading to different possible products. In both cases, the initial [4+2] cycloaddition (only one isomer shown) is followed by retro‐Diels‐Alder irreversible elimination of nitrogen, leading to dihydropyridazine‐type derivatives. Dihydro products are susceptible to oxidation, leading to aromatic pyridazines (B), which is not possible in pathway A. Only one dihydropyridazine tautomeric form is shown.

Based on these premises, commercially available fluorescent tetrazine **28** was incubated with **27** in water/DMSO for 24 h at room temperature. As shown in Figure 3, cycloaddition between **27** and **28** could theoretically generate different products, depending on which dienyl alkene acts as the dienophile.

Gratifyingly, a molecular ion [M+2H]^2+^ at *m*/*z* 906.3956, which is indicative of the molecular formula C_93_H_106_N_18_O_21_ (*δ*=−0.7), and a molecular ion [M+2H]^2+^ at *m*/*z* 905.3881, which is indicative of the molecular formula C_93_H_104_N_18_O_21_ (*δ*=−0.4), were consistent with successful fluorescence labelling of **27** by click chemistry (Figure S64). Data were in accordance with the formation of dihydropyridazine‐type (approx. 16 % conversion, as determined by HPLC at 254 nm) and pyridazine‐type products. Moreover, the product chromatographic peaks showed, as expected, absorbance in the visible‐light spectrum (441 nm).

A reaction between unmodified peptide **24** and tetrazine **28** was also set up for comparison; as expected, no clicked products were detected, showing the specificity of the click transformation for enzymatically modified peptide **27** (Figure S65).

Further characterization of the labelled products was beyond the scope of this proof‐of‐concept study. Nevertheless, in principle, we would expect pathway B to be kinetically favoured, due to faster reaction with the least substituted and less sterically hindered alkene. Moreover, our findings are consistent with the formation of pyridazine‐type products. However, this would only be possible in pathway B because pathway A does not allow dihydropyridazine aromatization due to lack of an extractable proton on the heterocyclic ring.

## Conclusion

In summary, we have synthesised a library of unnatural pyrophosphate alkyl donors exhibiting chemically reactive groups which are compatible with appealing techniques such as CuAAC, metathesis and IEDDA. We investigated the promiscuity of the cyanobactin prenyltransferase AcyF towards the prenyl donor and showed that it can achieve late‐stage incorporation of reactive moieties onto cyclic peptides, which then become amenable to additional functionalization. Moreover, we demonstrated that such reactive moieties can be exploited for successful late‐stage functionalization of a complex cyclic peptide by inverse‐electron‐demand Diels–Alder click reactions. To the best of our knowledge, this is the first report which demonstrates the feasibility of this chemoenzymatic route.

## Conflict of interest

The authors declare no conflict of interest.

1

## Supporting information

As a service to our authors and readers, this journal provides supporting information supplied by the authors. Such materials are peer reviewed and may be re‐organized for online delivery, but are not copy‐edited or typeset. Technical support issues arising from supporting information (other than missing files) should be addressed to the authors.

Supporting Information

## Data Availability

The data that support the findings of this study are available in the Supporting Information of this article.
