# Supplementary material for: Chemoenzymatic Late‐Stage Modifications Enable Downstream Click‐Mediated Fluorescent Tagging of Peptides
Source: Angew Chem Int Ed Engl. 2023 Mar 10;62(16):e202215979. doi: 10.1002/anie.202215979 (PMC10946513; doi:10.1002/anie.202215979)
Supplement: Supplementary file 1 — Supporting Information [file ANIE-62-0-s001.pdf]

Angewandte Chemie
Eine Zeitschrift der Gesellschaft Deutscher Chemiker

Supporting Information

Chemoenzymatic Late-Stage Modifications Enable Downstream Click-Mediated Fluorescent Tagging of Peptides

*A. Colombano, L. Dalponte, S. Dall'Angelo, C. Clemente, M. Idress, A. Ghazal, W. E. Houssen**

Contents

1 Chemistry..S1

1.1 General information S1

1.2 Synthetic Routes..S1

Scheme S1 Synthesis of cofactors 11-13. i) CBr_4, PPh3, acetonitrile, then tris(tetrabutylammonium) hydrogen pyrophosphate,12 h; ii) Dess martin periodinane, CH_2Cl_2; iii) triethyl phosphonoacetate, NaH, THF, 30 min; iv) DIBAL-H, THF, -78 °C; v) CBr_4, PPh_3, CH_2Cl_2; vi) tris(tetrabutylammonium) hydrogen pyrophosphate, acetonitrile, 12 h..S2

Scheme S2 Synthesis of cofactors 14-19. i) MnO_2, CH_2Cl_2, PPh_3CHCO_2Me or PPh_3CHCO_2Et or $PPh_3CCH_3CO_2Et$, 24 h; ii) CBr_4, PPh_3, CH_2Cl_2; iii) DIBAL-H, THF, -78 °C; iv) NaN_3, NaI, DMSO, 45 °C, 12 h; v) CBr_4, PPh_3, acetonitrile, then tris(tetrabutylammonium) hydrogen pyrophosphate, 12 h..S2

Scheme S3 Synthesis of cofactors 20-23. i) MsCl, TEA, CH_2Cl_2, 1 h, then DBU, 12 h; ii) $NaBH_4$, $CeCl_3$*$7H_2O$, MeOH; iii) $SOCl_2$, benzotriazole, CH_2Cl_2, 10 min.; iv) tris(tetrabutylammonium) hydrogen pyrophosphate, acetonitrile, 12 h; v) Tosyl chloride, 4-dimethylaminopyridine , triethylamine, CH_2Cl_2, 3 h; vi) NaN_3, DMSO, 45 °C, 12 h; vii) NaN_3, NaI, DMSO, 0 °C to rt, 30 min. S3

1.3 General procedures..S3

1.4 Pyrophosphate synthesis S5

Table S1 HRMS data summary for new synthetic alkyl pyrophosphates S30

1.5 Peptide synthesis S30

Figure S1 LC-MS for purified cyclo-[TSQIWGSPVP] (24)...................................S32

2 Biochemistry..S32

2.1 Expression and purification of AcyF S32

2.2 Enzymatic reaction general protocol..S32

2.3 LC-HRMS of enzymatic reactions and corresponding negative controls..............S34

Table S2 HRMS confirmation of AcyF-Catalysed reactions with 24. Reaction with 20 yielded 6 products while reaction with 22 afforded 3 products...............................S34

Figure S2 LC-HRMS (EIC) of reaction between 24 and 1. Top diagram shows extracted ion current (± 0.5 Da) for the expected product ([M+H]$^+$). Bottom diagram shows HRMS spectrum of the expected product (T_R 22.4 min). S35

Figure S3 LC-HRMS of negative control reaction between 24 and 1. All reaction components were included apart from the enzyme. Top diagram shows total ion current. Bottom diagram shows extracted ion current (± 0.5 Da) for the expected product ([M+H]+), which could not be found. S36

Figure S4 LC-HRMS (EIC) of reaction between 24 and 2. Top diagram shows total ion current. Bottom diagram shows extracted ion current (± 0.5 Da) for the expected product ([M+H]$^+$), which could not be found. S37

Figure S5 LC-HRMS of negative control reaction between 24 and 2. All reaction components were included apart from the enzyme. Top diagram shows total ion current. Bottom diagram shows extracted ion current (± 0.5 Da) for the expected product ([M+H]+), which could not be found. S38

Figure S6 LC-HRMS (EIC) of reaction between 24 and 3. Top diagram shows extracted ion current (± 0.5 Da) for the expected product ([M+H]$^+$). Bottom diagram shows HRMS spectrum of the expected product (T_R 21.5 min). S39

Figure S7 LC-HRMS of negative control reaction between 24 and 3. All reaction components were included apart from the enzyme. Top diagram shows total ion current. Bottom diagram shows extracted ion current (± 0.5 Da) for the expected product ([M+H]+), which could not be found. S40

Figure S8 LC-HRMS of reaction between 24 and 4. Top diagram shows total ion current. Bottom diagram shows extracted ion current (± 0.5 Da) for the expected product ([M+H]$^+$), which could not be found. S41

Figure S9 LC-HRMS of negative control reaction between 24 and 4. All reaction components were included apart from the enzyme. Top diagram shows total ion current. Bottom diagram shows extracted ion current (± 0.5 Da) for the expected product ([M+H]+), which could not be found. S42

Figure S10 LC-HRMS of reaction between 24 and 5. Top diagram shows UV absorbance (260 nm). Bottom diagram shows extracted ion current (± 0.5 Da) for the expected product ([M+H]$^+$), which could not be found. S43

Figure S11 LC-HRMS of negative control reaction between 24 and 5. All reaction components were included apart from the enzyme. Top diagram shows total ion current. Bottom diagram shows extracted ion current (± 0.5 Da) for the expected product ([M+H]+), which could not be found. S44

Figure S12 LC-HRMS (EIC) of reaction between 24 and 6. Top diagram shows extracted ion current (± 0.5 Da) for the expected product ($[M+H]^+$). Bottom diagram shows HRMS spectrum of the expected product (T_R 24.8 min). S45

Figure S13 LC-HRMS of negative control reaction between 24 and 6. All reaction components were included apart from the enzyme. Top diagram shows total ion current. Bottom diagram shows extracted ion current (± 0.5 Da) for the expected product ([M+H]+), which could not be found. S46

Figure S14 LC-HRMS of reaction between 24 and 7. Top diagram shows UV absorbance (260 nm). Bottom diagram shows extracted ion current (± 0.5 Da) for the expected product ($[M+H]^+$), which could not be found...S47

Figure S15 LC-HRMS of negative control reaction between 24 and 7. All reaction components were included apart from the enzyme. Top diagram shows total ion current. Bottom diagram shows extracted ion current (± 0.5 Da) for the expected product ([M+H]+), which could not be found. S48

Figure S16 LC-HRMS (EIC) of reaction between 24 and 8. Top diagram shows extracted ion current (± 0.5 Da) for the expected product ($[M+H]^+$). Bottom diagram shows HRMS spectrum of the expected product (T_R 22.4 min). S49

Figure S17 LC-HRMS of reaction between 24 and 9. Top diagram shows total ion current. Bottom diagram shows extracted ion current (± 0.5 Da) for the expected product ($[M+H]^+$), which could not be found...S50

Figure S18 LC-HRMS of negative control reaction between 24 and 9. All reaction components were included apart from the enzyme. Top diagram shows total ion current. Bottom diagram shows extracted ion current (± 0.5 Da) for the expected product ([M+H]+), which could not be found. S51

Figure S19 LC-HRMS of reaction between 24 and 10. Top diagram shows total ion current. Bottom diagram shows extracted ion current (± 0.5 Da) for the expected product ($[M+H]^+$), which could not be found...S52

Figure S20 LC-HRMS of reaction between 24 and 11. Top diagram shows total ion current. Bottom diagram shows extracted ion current (± 0.5 Da) for the expected product ([M+H]$^+$), which could not be found. S53

Figure S21 LC-HRMS of negative control reaction between 24 and 11. All reaction components were included apart from the enzyme. Top diagram shows total ion current. Bottom diagram shows extracted ion current (± 0.5 Da) for the expected product ([M+H]+), which could not be found. S54

Figure S22 LC-HRMS of reaction between 24 and 12. Top diagram shows total ion current. Bottom diagram shows extracted ion current (± 0.5 Da) for the expected product ([M+H]$^+$), which could not be found. S55

Figure S23 LC-HRMS of negative control reaction between 24 and 12. All reaction components were included apart from the enzyme. Top diagram shows total ion current. Bottom diagram shows extracted ion current (± 0.5 Da) for the expected product ([M+H]+), which could not be found. S56

Figure S24 LC-HRMS of reaction between 24 and 13. Top diagram shows total ion current. Bottom diagram shows extracted ion current (± 0.5 Da) for the expected product ([M+H]$^+$), which could not be found. S57

Figure S25 LC-HRMS of negative control reaction between 24 and 13. All reaction components were included apart from the enzyme. Top diagram shows total ion current. Bottom diagram shows extracted ion current (± 0.5 Da) for the expected product ([M+H]+), which could not be found. S58

Figure S26 LC-HRMS of reaction between 24 and 14. Top diagram shows UV absorbance (260 nm). Bottom diagram shows extracted ion current (± 0.5 Da) for the expected ([M+H]$^+$), which could not be found. S59

Figure S27 LC-HRMS of negative control reaction between 24 and 14. All reaction components were included apart from the enzyme. Top diagram shows total ion current. Bottom diagram shows extracted ion current (± 0.5 Da) for the expected product ([M+H]+), which could not be found. S60

Figure S28 LC-HRMS of reaction between 24 and 15. Top diagram shows UV absorbance (260 nm). Bottom diagram shows extracted ion current (± 0.5 Da) for the expected ([M+H]$^+$), which could not be found. S61

Figure S29 LC-HRMS of negative control reaction between 24 and 15. All reaction components were included apart from the enzyme. Top diagram shows total ion current. Bottom diagram shows extracted ion current (± 0.5 Da) for the expected product ([M+H]+), which could not be found. S62

Figure S30 LC-HRMS of reaction between 24 and 16. Top diagram shows UV absorbance (260 nm). Bottom diagram shows extracted ion current (± 0.5 Da) for the expected ($[M+H]^+$), which could not be found. S63

Figure S31 LC-HRMS of negative control reaction between 24 and 16. All reaction components were included apart from the enzyme. Top diagram shows total ion current. Bottom diagram shows extracted ion current (± 0.5 Da) for the expected product ([M+H]+), which could not be found. S64

Figure S32 LC-HRMS of reaction between 24 and 17. Top diagram shows UV absorbance (260 nm). Bottom diagram shows extracted ion current (± 0.5 Da) for the expected ($[M+H]^+$), which could not be found. S65

Figure S33 LC-HRMS of negative control reaction between 24 and 17. All reaction components were included apart from the enzyme. Top diagram shows total ion current. Bottom diagram shows extracted ion current (± 0.5 Da) for the expected product ([M+H]+), which could not be found. S66

Figure S34 LC-HRMS of reaction between 24 and 18. Top diagram shows UV absorbance (260 nm). Bottom diagram shows extracted ion current (± 0.5 Da) for the expected product ($[M+H]^+$), which could not be found. S67

Figure S35 LC-HRMS of negative control reaction between 24 and 18. All reaction components were included apart from the enzyme. Top diagram shows total ion current. Bottom diagram shows extracted ion current (± 0.5 Da) for the expected product ([M+H]+), which could not be found. S68

Figure S36 LC-HRMS of reaction between 24 and 19. Top diagram shows UV absorbance (260 nm). Bottom diagram shows extracted ion current (± 0.5 Da) for the expected product ($[M+H]^+$), which could not be found. S69

Figure S37 LC-HRMS of negative control reaction between 24 and 19. All reaction components were included apart from the enzyme. Top diagram shows total ion current. Bottom diagram shows extracted ion current (± 0.5 Da) for the expected product ([M+H]+), which could not be found. S70

Figure S38 LC-HRMS of reaction between 24 and 20. Top diagram shows the extracted ion current (± 0.5 Da) for the expected product. Six products were observed at T_R 18.5, 19.9, 27.7, 29.3, 34.0 and 34.9 min. Bottom diagrams show the high resolution mass spectrum of each product. S74

Figure S39 LC-HRMS of negative control reaction between 24 and 20. All reaction components were included apart from the enzyme. Top diagram shows total ion current. Bottom diagram shows extracted ion current (± 0.5 Da) for the expected product ([M+H]+), which could not be found. S75

Figure S40 LC-HRMS of reaction between 24 and 21. Top diagram shows UV absorbance (260 nm). Bottom diagram shows extracted ion current (± 0.5 Da) for the expected product ($[M+H]^+$), which could not be found. S76

Figure S41 LC-HRMS of negative control reaction between 24 and 21. All reaction components were included apart from the enzyme. Top diagram shows total ion current. Bottom diagram shows extracted ion current (± 0.5 Da) for the expected product ([M+H]+), which could not be found. S77

Figure S42 LC-HRMS of reaction between 24 and 22. Top diagram shows the extracted ion current for the expected product ($M-HN_3$). Three products were observed at T_R 4.56, 4.64 and 4.74 min. Bottom diagram show the high resolution mass spectrum of each product. S79

Figure S43 LC-HRMS of negative control reaction between 24 and 22. All reaction components were included apart from the enzyme. Top diagram shows total ion current. Bottom diagram shows extracted ion current (± 0.5 Da) for the expected product ($M-HN_3$), which could not be found. S80

Figure S44 LC-HRMS of reaction between 24 and 23. Top diagram shows UV absorbance (260 nm). Bottom diagram shows extracted ion current (± 0.5 Da) for the expected product ($[M+H]^+$), which could not be found S81

Figure S45 LC-HRMS of negative control reaction between 24 and 23. All reaction components were included apart from the enzyme. Top diagram shows total ion current. Bottom diagram shows extracted ion current (± 0.5 Da) for the expected product ([M+H]+), which could not be found S82

2.4 Isolation of 25 ....S83

Figure S46 Top diagram shows 25 (12.2 min.) immediately after HPLC purification. Bottom diagram shows partial decomposition of 25 (11.8 min) to 24 (10.2 min) after heating at 37 °C in the rotary evaporator bath. S83

2.5 Kinetic parameters determination S84

Figure S47 Michaelis-Menton kinetics for AcyF using 0.5 mM 24 and variable concentrations of 1 (a) or 20 (b) (0.01- 2 mM) in 10 mM HEPES buffer, 150 mM NaCl, MgCl2 12 mM, pH 7 at 30 °C. Experiments were done in duplicates and error bars indicate the SE of the mean. S84

2.6 Isolation of 26/27 .... S86

Figure S48 LC-MS for isolated 26/27. .... S86

2.6.1 NMR data for 26/27 .... S87

Figure S49 ^{1}H NMR (800 MHz) for 26/27 in DMSO-d_6 .... S88

Figure S50 ^{1}H-^{1}H COSY NMR for 26/27 in DMSO-d_6. .... S89

Figure S51 ^{1}H-^{13}C HSQC NMR for 26/27 in DMSO-d_6. .... S90

Figure S52 ^{1}H-^{15}N HSQC NMR for 26/27 in DMSO-d_6. .... S91

Figure S53 ^{1}H-^{13}C HMBC NMR for 26/27 in DMSO-d_6. .... S92

Figure S54 ROESY NMR for 26/27 in DMSO-d_6. .... S93

Figure S55 ^{13}C NMR (201 MHz) for 26/27 in DMSO-d_6. .... S94

Figure S56 ^{13}C DEPT NMR for 26/27 in DMSO-d_6. .... S95

Figure S57 Comparison of ^{1}H NMR for 24 (top) and 26/27 (bottom) in DMSO-d_6. S26 does not display tryptophan NH resonance (10.8 ppm) and shows 4 additional signals between 4.6 and 6 ppm. S96

Figure S58 Comparison of the ^{1}H-^{13}C HSQC spectra of 20 (left, D_2O) and 26/27 (right, DMSO-d_6). S96

Table S3 NMR data for 27 in DMSO-d_6 .... S98

Figure S59 Partially assigned ^{1}H-^{1}H COSY and ^{1}H-^{13}C HSQC NMR for isolated 27. .... S99

Figure S60 Partially assigned ^{1}H-^{13}C HSQC and ^{1}H-^{13}C HMBC NMR for isolated isolated 27. .... S100

Figure S61 Partially assigned ROESY NMR for isolated isolated 27.....................S101

2.6.2 HRMS and MS/MS...S102

Figure S62 HRMS of 27. ....S102

Figure S63 Pulsed-Q dissociation MS/MS for isolated 27. Predominant species observed were *b*-ions. Ions *b*6-*b*5 are consistent with loss of alkylated tryptophan. S108

2.7 Labelling of 27 with pyrimidyl-tetrazine-5-FAM (28) S109

Figure S64 LC-HRMS (441/254 nm) for click labelling of 27 (5.40 min) with fluorescent tetrazine 28 (4.62 min). Dihydropyridazine-type (5.54 min) and pyridazine-type (5.18 min) products were detected. S112

Figure S65 LC-HRMS (441 nm) for control labelling between unmodified peptide 24 (5.5 min.) and tetrazine 28 (6.8 min). As expected, no clicked products were observed. S114

Figure S66 Proposed formation mechanism for 27. The carbocation generated from 20 can exist in 3 different resonance structures (20-I, 20-II and 20-III). Indole nitrogen nucleophilic attack to resonance structure 20-III structure could lead to isolated compound 27..S115

3 References S116

4 NMR spectra of pyrophosphates 2-23 ....S117

5 NMR spectra of new intermediates S180

1 Chemistry

1.1 General information

All reactions were carried out under nitrogen atmosphere and using dry solvents. Dry solvents were obtained from commercial sources and used without further purification. Reactions were monitored by thin-layer chromatography (TLC), unless otherwise noted. TLCs were performed on Merck silica gel glass plates (60 F_{254}). Visualization was accomplished by UV light (254 nm) or staining with a ceric ammonium molybdate, $KMnO_4$ and ninhydrin solution. Flash chromatography was performed using Silica gel (60 Å, particle size 40-63 μm) purchased from Merck, unless otherwise noted. ^{1}H NMR, ^{13}C NMR, ^{31}P NMR spectra were recorded on a Bruker AVANCE III 400 NMR spectrometer or on a 600 MHz Bruker Ascend 2 channel HD instrument with a 5 mm CPTCI Prodigy ^{1}H/^{13}C/^{19}F probe with SampleCase 24 holder sample changer and calibrated using residual non deuterated solvent as internal reference. ^{13}C NMR spectra were recorded with complete proton decoupling. Chemical shifts (δ) are reported in parts per million (ppm) and coupling constants (*J*) are given in Hertz (Hz). The following abbreviations are used for spin multiplicity: s = singlet, d = doublet, t = triplet, q = quartet, dd = doublet-doublet, dt = doublet-triplet, tt = triplet-triplet, m = multiplet, br = broad. When necessary, resonances were assigned using two-dimensional experiments (COSY and HSQC). Low resolution mass analyses were performed using Agilent 1200 HPLC system coupled to Agilent G6120 single quadrupole detector equipped with an electrospray ionization (ESI) source in direct infusion modality. ESI-MS spectra were recorded in positive mode, unless otherwise noted. High resolution mass spectrometry analyses were performed by from National Mass Spectrometry Facility at Swansea University

1.2 Synthetic Routes

Compounds **1/8** are commercially available. Compounds **2-7** and **9** were directly synthesized from their corresponding alkyl halides. Compound **10** was prepared as previously described[1]. Synthesis of **11-13** is described in **Scheme S1.** Synthesis of **14-19** is described in **Scheme S2.** Synthesis of **20-23** is described in **Scheme S3.**

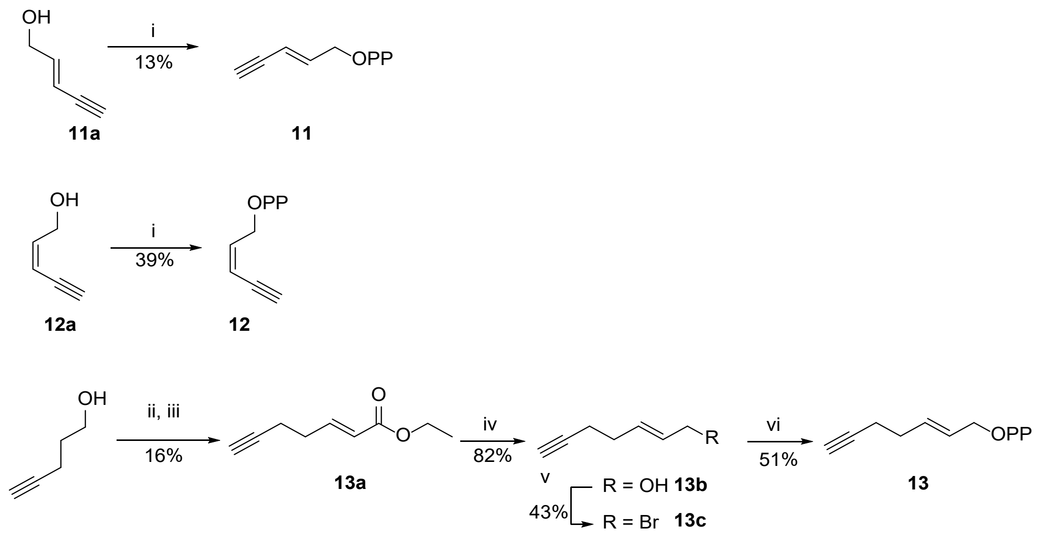

Scheme S1 Synthesis of cofactors **11-13**. i) CBr_4, PPh3, acetonitrile, then tris(tetrabutylammonium) hydrogen pyrophosphate,12 h; ii) Dess martin periodinane, CH_2Cl_2; iii) triethyl phosphonoacetate, NaH, THF, 30 min; iv) DIBAL-H, THF, -78 °C; v) CBr_4, PPh_3, CH_2Cl_2; vi) tris(tetrabutylammonium) hydrogen pyrophosphate, acetonitrile, 12 h.

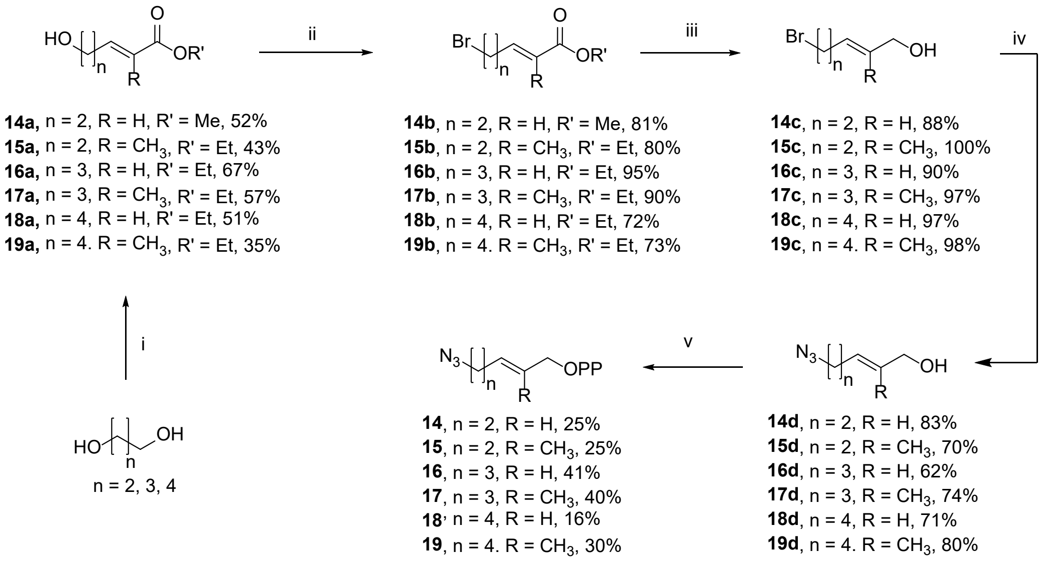

Scheme S2 Synthesis of cofactors **14-19**. i) MnO_2, CH_2Cl_2, PPh_3CHCO_2Me or PPh_3CHCO_2Et or $PPh_3CCH_3CO_2Et$, 24 h; ii) CBr_4, PPh_3, CH_2Cl_2; iii) DIBAL-H, THF, -78 °C; iv) NaN_3, NaI, DMSO, 45 °C, 12 h; v) CBr_4, PPh_3, acetonitrile, then tris(tetrabutylammonium) hydrogen pyrophosphate, 12 h.

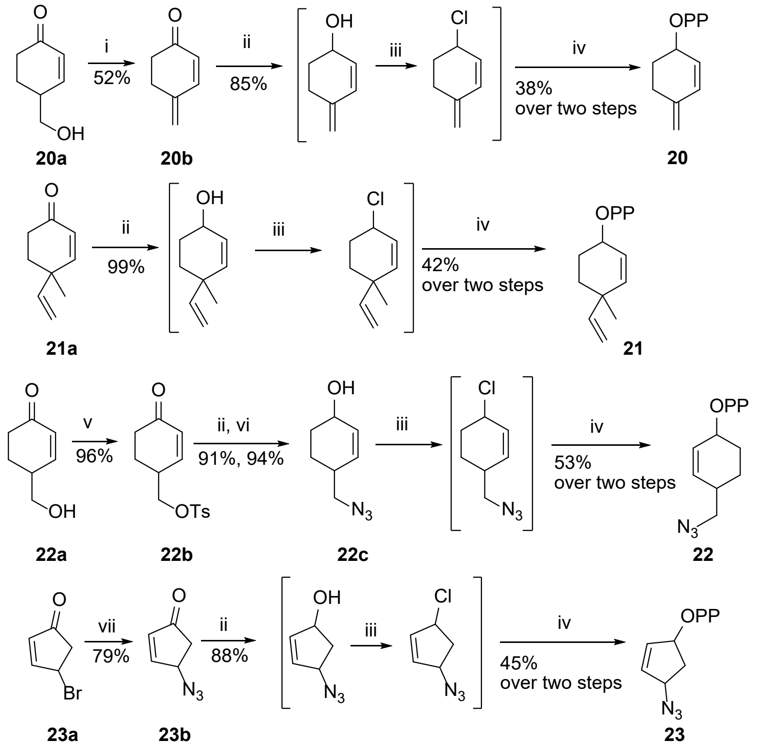

Scheme S3 Synthesis of cofactors **20**-**23**. i) MsCl, TEA, CH_2Cl_2, 1 h, then DBU, 12 h; ii) $NaBH_4$, $CeCl_3$*$7H_2O$, MeOH; iii) $SOCl_2$, benzotriazole, CH_2Cl_2, 10 min.; iv) tris(tetrabutylammonium) hydrogen pyrophosphate, acetonitrile, 12 h; v) Tosyl chloride, 4-dimethylaminopyridine , triethylamine, CH_2Cl_2, 3 h; vi) NaN_3, DMSO, 45 °C, 12 h; vii) NaN_3, NaI, DMSO, 0 °C to rt, 30 min.

1.3 General procedures

General procedure A, for the reduction of α,β-unsaturated esters.

Diisobutylaluminum hydride solution (DIBAL-H) 1 M in hexane (2–2.5 equiv.) was dropwise added to a solution of unsaturated ester (1 equiv.) in tetrahydrofuran (THF) (0.5 M) at -78 °C and stirred at the same temperature until the starting material was fully consumed (by TLC, usually 10’–40’). The reaction was quenched at -78 °C with sodium sulfate saturated aqueous solution, warmed to room temperature until white flakes formed, filtered and extracted with Et_2O. The organic layer was washed with HCl 1 M aqueous solution, brine, dried over sodium sulfate and concentrated under reduced pressure. The product was used in next step without further purification, unless otherwise noted.

General procedure B, for the synthesis of alkyl pyrophosphates from alkyl halides.

This procedure was adapted from the original protocol of Davisson *et al*[2].

Tris(tetrabutylammonium) hydrogen pyrophosphate (1.5-2 equiv.) was portion wise added to a solution of bromide/chloride (1 equiv.) in acetonitrile (0.5 M, referred to pyrophosphate salt) under nitrogen atmosphere, which had been cooled to 0 °C. The reaction was stirred overnight at room temperature. The solvent was removed under reduced pressure and the residue was dissolved in the ion exchange buffer (IEB). IEB consisted of 1:49 (v/v) isopropanol and 25 mM ammonium bicarbonate in water. The clear solution was slowly passed through a column packed with 30 equivalents of DOWEX AG 50W-X8 (100-200 mesh) resin (ammonium form) that had been equilibrated with 2 column volumes of IEB. The column was eluted with strictly 2 column volumes at a flow rate of 1 column volume/15 min. The eluate was freeze-dried to yield a white solid. The ammonium form of the resin was generated by washing DOWEX AG 50W-X8 (100-200 mesh) resin (hydrogen form) on a fritted glass with 4 x 20 mL concentrated ammonium hydroxide aqueous solution followed by water until the pH of the filtrate was 7. The resin was then washed with 2 x 20 mL IEB and slurry packed into the column. The white solid recovered after freeze-drying was dissolved in 1.5 mL of 0.1 M ammonium bicarbonate solution in water. 6 mL of 1:1 (v/v) acetonitrile and isopropanol were then added and thoroughly vortexed until a white precipitate formed. The mixture was centrifuged at 2000 rpm for five minutes before the supernatant was collected. The process was repeated three times. The combined supernatants were concentrated under reduced pressure at 40 °C and freeze-dried overnight. The resulting solid was dissolved in a minimum amount of chromatography buffer (isopropanol/acetonitrile/0.1 M ammonium bicarbonate aqueous solution) and purified by flash chromatography on cellulose. The eluate was freeze-dried overnight affording the title compound as a white fluffy solid.

<u>General procedure C, for the one-pot synthesis of alkyl pyrophosphates via Appel reaction.</u>

PPh_3 (1.2–1.5 equiv.) was added to a solution of alcohol (1 equiv.) and CBr_4 (1.2 - 1.5 equiv.) in acetonitrile (0.5 M, referred to pyrophosphate salt) at 0 °C and the reaction was monitored at room temperature. After completion (as monitored by TLC, usually 10'-30') the reaction was cooled to 0 °C and solid tris(tetrabutylammonium) hydrogen pyrophosphate (1.5 equiv.) was portion wise added; the reaction was stirred overnight at room temperature. The solvent was removed under reduced pressure, the residue was diluted with a minimum amount of IEB (see general procedure B) and washed with DCM (x3). The IEB layer was then processed as described in general procedure B.

General procedure D, for the synthesis of α,β-unsaturated esters from diols[3].

Manganese dioxide (20 equiv.) was added to a solution of diol (1 equiv.) and Wittig phosphorane (2.5 equiv.) in DCM (0.1 M) and the reaction was stirred for 24 hours. The mixture was filtered over celite, concentrated under reduced pressure, and purified by flash chromatography to afford the desired compound.

General procedure E, Appel reaction.

PPh_3 (1.2 equiv.) was added to a solution of alcohol (1 equiv.) and CBr_4 (1.2 equiv.) in DCM (0.6 M) at 0 °C and the reaction was monitored at room temperature. After completion (by TLC, usually 30'- 1 hour) the solvent was removed under reduced pressure and the residue was purified by flash chromatography, to afford the desired bromide.

General procedure F, synthesis of azides from alkyl bromides

Sodium azide (2 equiv.) was added to a solution of bromide (1 equiv.) and NaI (0.1 equiv.) in DMSO (0.5 M) and the mixture was heated at 45 °C overnight. The mixture was cooled to room temperature, diluted with water and extracted with Et_2O. The combined organic layers were washed with brine, dried over sodium sulfate and concentrated under reduced pressure. Purification by flash chromatography afforded the desired azide.

1.4 Pyrophosphate synthesis

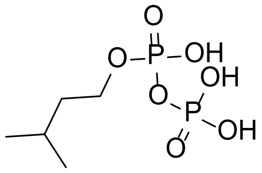

Isopentyl trihydrogen diphosphate (2)

1-Bromo-3-methylbutane (28 µL, 0.23 mmol) in acetonitrile (5 mL) was treated with tris(tetrabutylammonium) hydrogen pyrophosphate (424 mg, 0.47 mmol) according to general procedure B. Purification by flash chromatography (5:2.5:2.5 isopropanol/acetonitrile/0.1 M ammonium bicarbonate) afforded the desired compound.

Spectroscopic data were in agreement with the literature[2].

^{1}H NMR (400 MHz, D_2O) δ = 3.87 (dt, J = 6.8 Hz, 2H), 1.61 (m, 1H), 1.44 (dd, J = 6.9 Hz, 2H), 0.81 (d, J = 6.6 Hz, 6H).

^{13}C NMR (151 MHz, D_2O) δ = 65.0 (d, *J*= 5.8 Hz), 38.7 (d, *J* = 7.1 Hz), 24.1, 21.8.

^{31}P NMR (162 MHz, D_2O) δ = -7.8 (d, *J* = 20.4 Hz, 1P), -10.4 (d, *J* = 20.4 Hz, 1P).

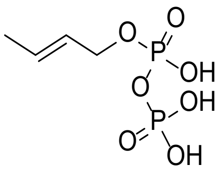

(*E*)-But-2-en-1-yl trihydrogen diphosphate (3)

Crotyl chloride (23 µL, 0.23 mmol) in acetonitrile (4 mL) was treated with tris(tetrabutylammonium) hydrogen pyrophosphate (424 mg, 0.47 mmol) according to general procedure B. Purification by flash chromatography (5:2.5:2.5 isopropanol/acetonitrile/0.1 M ammonium bicarbonate) afforded the desired compound.

Spectroscopic data were in agreement with the literature[4].

^{1}H NMR (400 MHz, D_2O) δ = 5.82-5.93 (m, 1H), 5.65-5.74 (m, 1H), 4.37 (t, *J* = 6.6 Hz, 2H), 1.71 (dd, *J* = 6.4, 1.4 Hz, 3H).

^{13}C NMR (151 MHz, D_2O) δ = 130.9, 126.7 (d, *J* = 7.5 Hz), 66.6 (d, *J* = 5.0 Hz), 17.0.

^{31}P NMR (162 MHz, D_2O) δ = -6.5 (m, 1P), -10.4 (m, 1P).

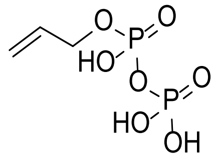

Allyl trihydrogen diphosphate (4)

Allyl bromide (20 µL, 0.23 mmol) in acetonitrile (5 mL) was treated with tris(tetrabutylammonium) hydrogen pyrophosphate (424 mg, 0.47 mmol) according to general procedure B. Purification by flash chromatography (5:2.5:2.5 isopropanol/acetonitrile/0.1 M ammonium bicarbonate) afforded the desired compound.

R_f 0.14 (5:2.5:2.5 isopropanol/acetonitrile/0.1 M ammonium bicarbonate);

^{1}H NMR (600 MHz, D_2O) δ = 6.07 – 5.97 (m, 1H), 5.39 (dd, *J* = 17.2, 1.5 Hz, 1H), 5.23 (dd, *J* = 10.5, 1.3 Hz, 1H), 4.47 – 4.42 (m, 2H).

^{13}C NMR (151 MHz, D_2O) δ = 134.4 (d, *J* = 7.4 Hz), 116.8, 66.6 (d, *J* = 5.1 Hz).

^{31}P NMR (162 MHz, D_2O) δ = -6.4 (d, *J* = 21.9 Hz, 1P), -10.3 (d, *J* = 21.9 Hz, 1P).

HRMS (ESI, *m/z*): calcd. for $C_3H_7O_7P_2$ $[M-H]^-$: 216.9672, found 216.9678

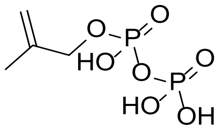

2-Methylallyl trihydrogen diphosphate (5)

3-Bromo-2-methylpropene (75 µL, 0.74 mmol) in acetonitrile (2.2 mL) was treated with tris(tetrabutylammonium) hydrogen pyrophosphate (1 g, 1.11 mmol) according to general procedure B. Purification by flash chromatography (4:3:3 isopropanol/acetonitrile/0.1 M ammonium bicarbonate) afforded the desired compound (144 mg, 68%).

Rf 0.4 (4:3:3 isopropanol/acetonitrile/0.1 M ammonium bicarbonate);

^{1}H NMR (400 MHz, D_2O) δ = 4.97 (br s, 1H), 4.84 (br s, 1H), 4.25 (d, *J* = 6.9 Hz, 2H), 1.66 (s, 3H).

^{13}C NMR (151 MHz, D_2O) δ = 142.7 (d, *J* = 7.1 Hz), 111.2, 69.0 (d, *J* = 5.3 Hz), 18.4.

^{31}P NMR (162 MHz, D_2O) δ = -6.5 (br s, 1P), -10.4. (br s, 1P)

HRMS (ESI, *m/z*): calcd. for $C_4H_9O_7P_2$ [M-H]$^-$: 230.9829, found 230.9834.

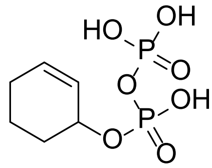

(±)-Cyclohex-2-en-1-yl trihydrogen diphosphate (6)

3-Bromocyclohexene (26 µL, 0.23 mmol) in acetonitrile (5 mL) was treated with tris(tetrabutylammonium) hydrogen pyrophosphate (424 mg, 0.47 mmol) according to general procedure B. Purification by flash chromatography (5:2.5:2.5 isopropanol/acetonitrile/0.1 M ammonium bicarbonate) afforded the desired compound.

Rf 0.3 (5:2.5:2.5 isopropanol/acetonitrile/0.1 M ammonium bicarbonate);

^{1}H NMR (600 MHz, D_2O) δ = 6.02 – 5.95 (m, 1H), 5.91 – 5.83 (m, 1H), 2.10 – 2.02 (m, 1H), 2.01 – 1.89 (m, 2H), 1.86 – 1.76 (m, 1H), 1.76 – 1.66 (m, 1H), 1.63 – 1.56 (m, 1H).

^{13}C NMR (151 MHz, D_2O) δ = 132.4, 127.5 (d, *J* = 5.1 Hz), 70.3 (d, *J* = 5.5 Hz), 29.6 (d, *J* = 3.5 Hz), 24.3, 18.4.

^{31}P NMR (162 MHz, D_2O) δ = -6.8 (d, *J* = 21.2 Hz, 1P), -10.9 (d, *J* = 21.7 Hz, 1P).

HRMS (ESI, *m/z*): calcd. for $C_6H_{11}O_7P_2$ [M-H]$^-$: 256.9985, found 256.9987

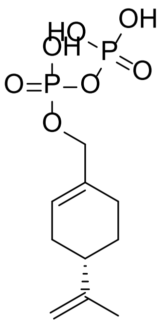

((S)-4-(Prop-1-en-2-yl)cyclohex-1-en-1-yl)methyl trihydrogen diphosphate (7)

(*S*)-Perillyl bromide[5] (108 mg, 0.50 mmol) in acetonitrile (2 mL) was treated with tris(tetrabutylammonium) hydrogen pyrophosphate (677 mg, 0.75 mmol) according to general procedure B. Purification by flash chromatography (4:3:3 isopropanol/acetonitrile/0.1 M ammonium bicarbonate) afforded the desired compound (161 mg, 88%).

R_f 0.3 (4:3:3 isopropanol/acetonitrile/0.1 M ammonium bicarbonate);

^{1}H NMR (400 MHz, D_2O) δ = 5.91 – 5.85 (m, 1H), 4.34 (d, *J* = 6.2 Hz, 2H), 2.28 – 2.09 (m, 4H), 2.07 – 1.94 (m, 1H), 1.93 – 1.83 (m, 1H), 1.78 (s, 3H), 1.58 – 1.43 (m, 1H).

^{13}C NMR (101 MHz, D_2O) δ = 152.2, 134.6 (d, *J* = 8.1 Hz), 124.9, 108.0, 69.9 (d, *J* = 5.7 Hz), 40.5, 29.9, 27.0, 25.5, 20.0.

^{31}P NMR (162 MHz, D_2O) δ = -8.2 – -8.7 (m, 1P), -10.7 (d, *J* = 21.3 Hz, 1P).

HRMS (ESI, *m/z*): calcd. for $C_{10}H_{17}O_7P_2$ $[M-H]^-$: 311.0455, found 311.0454.

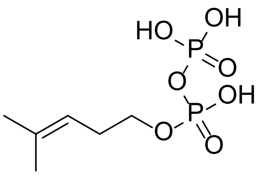

4-Methylpent-3-en-1-yl trihydrogen diphosphate (9)

5-Bromo-2-methyl-2-pentene (31 μL, 0.23 mmol) in acetonitrile (5 mL) was treated with tris(tetrabutylammonium) hydrogen pyrophosphate (424 mg, 0.47 mmol) according to general procedure B. Purification by flash chromatography (5:2.5:2.5 isopropanol/acetonitrile/0.1 M ammonium bicarbonate) afforded the desired compound.

Spectroscopic data were in agreement with the literature[4].

^{1}H NMR (400 MHz, D_2O) δ = 5.25 (tt, *J* = 7.1, 1.5 Hz, 1H), 3.91 (q, *J* = 6.7 Hz, 2H), 2.37 (q, *J* = 7.0 Hz, 2H), 1.73 (d, *J* = 1.6 Hz, 3H), 1.67 (d, *J* = 1.4 Hz, 3H).

^{13}C NMR (151 MHz, D_2O) δ = 135.9, 120.0, 65.5 (d, *J* = 5.6 Hz), 29.0 (d, *J* = 6.9 Hz), 24.8, 17.2.

^{31}P NMR (162 MHz, D_2O) δ = -6.4 (br s, 1P), -10.3 (br s, 1P).

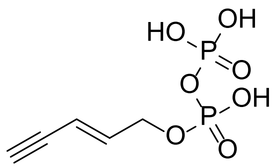

(*E*)-Pent-2-en-4-yn-1-yl trihydrogen diphosphate (11)

(*E*)-2-Penten-4-yn-1-ol[6] (49 mg, 0.60 mmol) in acetonitrile (2 mL) was treated with PPh_3 (189 mg, 0.72 mmol), CBr_4 (238 mg, 0.72 mmol) and tris(tetrabutylammonium) hydrogen pyrophosphate (812 mg, 0.90 mmol) according to general procedure C. Purification by flash chromatography (4:3:3 isopropanol/acetonitrile/0.1 M ammonium bicarbonate) afforded the desired compound (22 mg, 13%).

R$_f$ 0.5 (4:3:3 isopropanol/acetonitrile/0.1 M ammonium bicarbonate);

^{1}H NMR (400 MHz, D_2O) δ = 6.41 (dt, *J* = 16.0, 5.1 Hz, 1H), 5.86 (ddt, *J* = 16.0, 2.1, 1.8 Hz, 1H), 4.52 (m, 2H), 3.31 (d, *J* = 2.2 Hz, 1H).

^{13}C NMR (101 MHz, D_2O) δ = 141.5 (d, *J* = 7.5 Hz), 109.5, 82.1, 78.7, 65.1 (d, *J* = 4.8 Hz).

^{31}P NMR (162 MHz, D_2O) δ = -6.76 (br s, 1P), -10.51 (br s, 1P).

HRMS (ESI, *m/z*): calcd. for $C_5H_7O_7P_2$ $[M-H]^-$: 240.9672, found 240.9672.

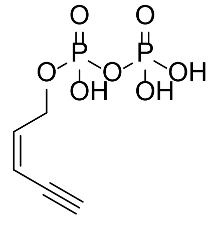

(*Z*)-Pent-2-en-4-yn-1-yl trihydrogen diphosphate (12)

(*Z*)-2-Penten-4-yn-1-ol[6] (54 mg, 0.66 mmol) in acetonitrile (2 mL) was treated with PPh_3 (260 mg, 0.99 mmol), CBr_4 (327 mg, 0.99 mmol) and tris(tetrabutylammonium) hydrogen pyrophosphate (890 mg, 0.99 mmol) according to general procedure C. Purification by flash chromatography (4:3:3 isopropanol/acetonitrile/0.1 M ammonium bicarbonate) afforded the title compound (74 mg, 39%).

R*$_f$* 0.5 (4:3:3 isopropanol/acetonitrile/0.1 M ammonium bicarbonate);

^{1}H NMR (400 MHz, D_2O) δ = 6.29 (dt, *J* = 11.0, 6.4 Hz, 1H), 5.68 (dd, *J* = 11.0, 1.8 Hz, 1H), 4.76 – 4.68 (m, 2H), 3.60 (d, *J* = 2.4 Hz, 1H).

^{13}C NMR (101 MHz, D_2O) δ = 141.1 (d, *J* = 7.5 Hz), 109.9, 84.4, 79.3, 63.6 (d, *J* = 4.8 Hz).

^{31}P NMR (162 MHz, D_2O) δ = -6.59 (d, *J* = 22.1 Hz, 1P), -10.31 (d, *J* = 21.9 Hz, 1P).

HRMS (ESI, *m/z*): calcd. for $C_5H_7O_7P_2$ $[M-H]^-$: 240.9672, found 240.9669.

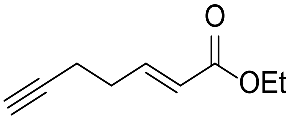

Ethyl (*E*)-hept-2-en-6-ynoate (13a)

Dess-Martin periodinane (3.5 g, 8.2 mmol, 1.5 equiv.) was portion wise added to a solution of commercial 4-pentyn-1-ol (460 mg, 5.47 mmol, 1 equiv.) in DCM (25 mL) which had been cooled to 0 °C (ice bath). The reaction mixture was warmed to room temperature and stirred for 1 hour at room temperature. The reaction was diluted with Et_2O (30 mL) and 30 mL of sat. aq. $NaHCO_3$ solution containing 25% of $Na_2S_2O_3$ were added. The obtained mixture was stirred for 10 minutes, and the layers were separated. The aqueous layer was extracted with Et_2O, washed with sat. aq. $NaHCO_3$ solution, brine, dried over Na_2SO_4 and evaporated under reduced pressure. The crude aldehyde was isolated as a yellow oil which was used in the following step without further purification and characterization.

Triethyl phosphonoacetate (1.2 mL, 6.12 mmol, 1.5 equiv.) was dropwise added to a suspension of NaH 60% w/w (244 mg, 6.12 mmol, 1.5 equiv.) in dry THF (13 mL) which had been cooled to 0 °C. The suspension was stirred at the same temperature for 30 minutes, at the end of which it became a yellow solution. A solution of the freshly prepared aldehyde (335 mg, 4.09 mmol, 1 equiv.) in dry THF (3 mL) was subsequently dropwise added at 0 °C. The resulting solution was stirred at 0 °C for 5 minutes and at RT for 30 minutes. After completion, the reaction was quenched with sat. aq. NH_4Cl solution and extracted with Et_2O. The organic layer was washed with brine, dried over Na_2SO_4 and concentrated under reduced pressure. Purification by flash chromatography (95:5 hexane/EtoAc) afforded the title compound (100 mg, 16% over two steps) as a colourless oil. Spectroscopic data are in accordance with the literature[7].

^{1}H NMR (400 MHz, $CDCl_3$): *δ* = 6.96 (dt, J = 15.7, *J* = 6.6 Hz, 1H), 5.88 (dt, *J* = 15.7, 1.6 Hz, 1H), 4.19 (q, *J* = 7.2 Hz, 2H), 2.46 – 2.39 (m, 2H), 2.37 – 2.32 (m, 2H), 1.99 (t, *J* = 2.6 Hz, 1H), 1.28 (t, *J* = 7.1 Hz, 3H).

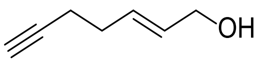

(*E*)-Hept-2-en-6-yn-1-ol (13b)

Ester **13a** (100 mg, 0.66 mmol) in THF (1 mL) was treated with DIBAL-H (1.64 mL) according to general procedure A, to give the corresponding unsaturated alcohol (59 mg, 82%) as a colourless oil, which was directly used in the next step without further purification.

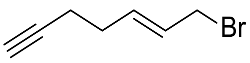

(*E*)-7-Bromohept-5-en-1-yne (13c)

Alcohol **13b** (59 mg, 0.54 mmol) in DCM (1 mL) was treated with PPh_3 (169 mg, 0.64 mmol) and CBr_4 (213 mg, 0.64 mmol) according to general procedure E. Purification by flash chromatography (100% pentane) afforded the desired compound (40 mg, 43%) as colourless oil. Spectroscopic data were in agreement with the literature[8].

^{1}H NMR (400 MHz, $CDCl_3$) δ = 5.88 – 5.71 (m, 2H), 3.95 (d, *J* = 6.5 Hz, 2H), 2.33 – 2.22 (m, 4H), 1.97 (t, *J* = 2.3 Hz, 1H).

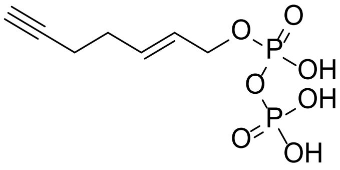

(*E*)-Hept-2-en-6-yn-1-yl trihydrogen diphosphate (13)

A solution of **13c** (40 mg, 0.23 mmol) in acetonitrile (0.7 mL) was treated with tris(tetrabutylammonium) hydrogen pyrophosphate (315 mg, 0.35 mmol) according to general procedure B. Purification by flash chromatography (4:3:3 isopropanol/acetonitrile/0.1 M ammonium bicarbonate) afforded the desired compound (38 mg, 51%).

R_f 0.5 (4:3:3 isopropanol/acetonitrile/0.1 M ammonium bicarbonate);

^{1}H NMR (600 MHz, D_2O) δ = 5.91 – 5.78 (m, 1H), 5.76 – 5.61 (m, 1H), 4.35 (t, *J* = 6.7 Hz, 2H), 2.34 – 2.15 (m, 5H).

^{13}C NMR (101 MHz, D_2O) δ = 133.0, 127.0 (d, *J* = 7.5 Hz), 85.5, 69.5, 66.5 (d, *J* = 5.2 Hz), 30.4, 17.4.

^{31}P NMR (162 MHz, D_2O) δ = -8.28 (br s, 1P), -10.57 (d, *J* = 21.3 Hz, 1P).

HRMS (ESI, *m/z*): calcd. for $C_7H_{11}O_7P_2$ $[M-H]^-$: 268.9985, found 268.9986.

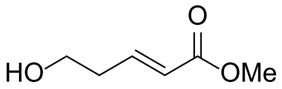

Methyl (*E*)-5-hydroxypent-2-enoate (14a)

1,3-Propanediol (475 µL, 6.57 mmol), methyl (triphenylphosphoranylidene)acetate (5.5 g, 16.4 mmol) and MnO_2 (11.5 g, 131 mmol) in DCM (160 mL) were reacted according to general procedure D. Purification by flash chromatography (55:45 hexane/EtOAc) afforded the known ester (445 mg, 52%) as pale orange oil.

Spectroscopic data were in agreement with the literature[9].

^{1}H NMR (400 MHz, $CDCl_3$): δ = 6.98 (dt, *J* = 15.4, 7.3 Hz, 1H), 5.94 (dt, *J* = 15.7, 1.4 Hz, 1H), 3.78 (t, *J* = 6.4 Hz, 2H), 3.74 (s, 3H), 2.48 (dq, *J* = 6.2, 1.4 Hz, 2H).

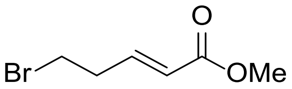

Methyl (*E*)-5-bromopent-2-enoate (14b)

14a (277 mg, 2.13 mmol) in DCM (4 mL) was treated with PPh_3 (670 mg, 2.55 mmol) and CBr_4 (846 mg, 2.55 mmol) according to general procedure E. Purification by flash chromatography (gradient 98:2 to 9:1 hexane/EtOAc) afforded the desired compound (334 mg, 81%) as pale-yellow oil.

Spectroscopic data were in agreement with the literature[10].

^{1}H NMR (400 MHz, $CDCl_3$) δ = 6.91 (dt, *J* = 15.7, 6.9 Hz, 1H), 5.92 (dt, *J* = 15.7, 1.5 Hz, 1H), 3.74 (s, 3H), 3.45 (t, *J* = 6.8 Hz, 2H), 2.78 (qd, *J* = 6.8, 1.5 Hz, 2H).

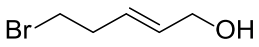

(*E*)-5-Bromopent-2-en-1-ol (14c)

14b (316 mg, 1.64 mmol) in THF (5 mL) was treated with DIBAL-H (4.1 mL) according to general procedure A, to give the corresponding unsaturated alcohol (237 mg, 88%) as a colourless oil, which was directly used in the next step without further purification.

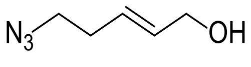

(*E*)-5-Azidopent-2-en-1-ol (14d)

14c (237 mg, 1.44 mmol), sodium iodide (22 mg, 0.14 mmol) and sodium azide (187.2 mg, 2.88 mmol) in DMSO (3 mL) were reacted according to general procedure F. Purification by flash chromatography (6:4 hexane/EtOAc) afforded the desired azide (151 mg, 83%) as a pale-yellow oil.

Rf 0.35 (6:4 hexane/EtOAc);

^{1}H NMR (400 MHz, $CDCl_3$) δ = 5.78 – 5.68 (m, 1H), 5.68 – 5.58 (m, 1H), 4.10 – 4.04 (m, 2H), 3.29 (t, *J* = 6.9 Hz, 2H), 2.37 – 2.27 (m, 2H), 2.16 (br s, 1H).

^{13}C NMR (101 MHz, $CDCl_3$) δ = 132.3, 127.7, 63.2, 50.8, 31.8.

MS (ESI, *m/z*): calcd. for $C_5H_9N_3ONa$ $[M+Na]^+$: 150.07, found 150.1.

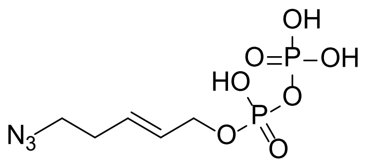

(*E*)-5-Azidopent-2-en-1-yl trihydrogen diphosphate (14)

14d (82 mg, 0.64 mmol) in acetonitrile (2 mL) was treated with PPh_3 (203 mg, 0.77 mmol), CBr_4 (255 mg, 0.77 mmol) and tris(tetrabutylammonium) hydrogen pyrophosphate (866 mg, 0.96 mmol) according to general procedure C. Purification by flash chromatography (4:3:3 isopropanol/acetonitrile/0.1 M ammonium bicarbonate) afforded the desired compound (54 mg, 25%).

Rf 0.55 (4:3:3 isopropanol/acetonitrile/0.1 M ammonium bicarbonate);

^{1}H NMR (400 MHz, D_2O) δ = 5.89 – 5.73 (m, 2H), 4.42 (dd, *J* = 7.1, 4.8 Hz, 2H), 3.42 (t, *J* = 6.8 Hz, 2H), 2.44 – 2.33 (m, 2H).

^{13}C NMR (101 MHz, D_2O) δ = 130.6, 128.5 (d, *J* = 7.8 Hz), 66.3 (d, *J* = 5.2 Hz), 50.3, 31.0.

^{31}P NMR (162 MHz, D_2O) δ = -7.6 (d, *J* = 21.4 Hz, 1P), -10.6 (d, *J* = 21.5 Hz, 1P).

HRMS (ESI, *m/z*): calcd. for $C_5H_{10}N_3O_7P_2$ $[M-H]^-$: 285.9999, found 285.9999.

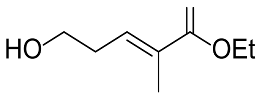

Ethyl (*E*)-5-hydroxy-2-methylpent-2-enoate (15a)

1,3-Propanediol (475 µL, 6.57 mmol), (carbethoxyethylidene)triphenylphosphorane (6.0 g, 16.4 mmol) and MnO_2 (11.4 g, 131 mmol) in DCM (50 mL) were reacted according to general procedure D. Purification by flash chromatography (6:4 hexane/EtOAc) afforded the known

ester (447 mg, 43%) as colourless oil. Spectroscopic data were in agreement with the literature[3].

^{1}H NMR (400 MHz, $CDCl_3$) δ = 6.78 (tq, *J* = 7.4, 1.5 Hz, 1H), 4.20 (q, *J* = 7.1 Hz, 2H), 3.77 (t, *J* = 6.5 Hz, 2H), 2.52 – 2.41 (m, 2H), 1.87 (q, *J* = 1.0 Hz, 3H), 1.53 (s, 1H), 1.30 (t, *J* = 7.1 Hz, 3H).

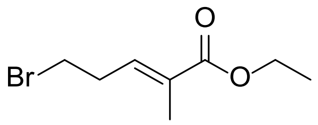

Ethyl (*E*)-5-bromo-2-methylpent-2-enoate (15b)

15a (447 mg, 2.83 mmol) in DCM (5 mL) was treated with PPh_3 (889 mg, 3.39 mmol) and CBr_4 (1.12 g, 2.55 mmol) according to general procedure E. Purification by flash chromatography (gradient 98:2 to 9:1 hexane/EtOAc) afforded the desired compound (503 mg, 80%) as colourless oil.

Spectroscopic data were in agreement with the literature[11].

^{1}H NMR (400 MHz, $CDCl_3$) δ = 6.71 (tq, *J* = 7.3, 1.5 Hz, 1H), 4.21 (q, *J* = 7.1 Hz, 2H), 3.44 (t, *J* = 6.9 Hz, 2H), 2.77 (qd, *J* = 7.1, 0.8 Hz, 2H), 1.88 – 1.83 (m, 2H), 1.30 (t, *J* = 7.1 Hz, 3H).

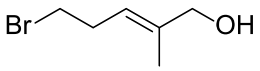

(*E*)-5-Bromo-2-methylpent-2-en-1-ol (15c)

15b (503 mg, 2.28 mmol) in THF (5 mL) was treated with DIBAL-H (5.7 mL) according to general procedure A, to give the corresponding unsaturated alcohol (408 mg, quantitative) as a colourless oil, which was directly used in the next step without further purification.

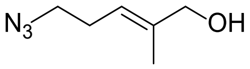

(*E*)-5-Azido-2-methylpent-2-en-1-ol (15d)

15c (408 mg, 2.32 mmol), sodium iodide (35 mg, 0.23 mmol) and sodium azide (302 mg, 4.64 mmol) in DMSO (4 mL) were reacted according to general procedure F. Purification by flash chromatography (6:4 hexane/Et_2O) afforded the desired azide (230 mg, 70%) as a pale-yellow oil.

R_f 0.3 (6:4 hexane/Et_2O);

^{1}H NMR (400 MHz, CD_2Cl_2) δ = 5.40 (tq, *J* = 7.2, 1.5 Hz, 1H), 3.96 (s, 2H), 3.28 (t, *J* = 7.0 Hz, 2H), 2.39 – 2.31 (m, 2H), 2.25 (br s, 1H), 1.67 (s, 3H).

^{13}C NMR (101 MHz, CD_2Cl_2) δ = 138.5, 120.9, 68.3, 51.4, 27.7, 13.8.

MS (ESI, *m/z*): calcd. for $C_6H_{11}N_3ONa$ $[M+Na]^+$: 164.09, found 164.1.

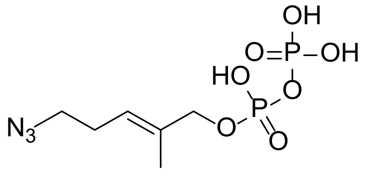

(*E*)-5-Azido-2-methylpent-2-en-1-yl trihydrogen diphosphate (15)

15d (107 mg, 0.76 mmol) in acetonitrile (2 mL) was treated with PPh_3 (239 mg, 0.91 mmol), CBr_4 (302 mg, 0.91 mmol) and tris(tetrabutylammonium) hydrogen pyrophosphate (1.03 g, 1.14 mmol) according to general procedure C. Purification by flash chromatography (4:3:3 isopropanol/acetonitrile/0.1 M ammonium bicarbonate) afforded the desired compound (66 mg, 25%).

***R**$_f$* 0.5 (4:3:3 isopropanol/acetonitrile/0.1 M ammonium bicarbonate);

^{1}H NMR (400 MHz, D_2O) δ = 5.48 (t, *J* = 7.3 Hz, 1H), 4.26 (d, *J* = 6.3 Hz, 2H), 3.30 (t, *J* = 6.8 Hz, 2H), 2.36 – 2.26 (m, 2H), 1.64 (s, 3H).

^{13}C NMR (101 MHz, D_2O) δ = 135.0 (d, *J* = 7.9 Hz), 123.7, 71.1 (d, *J* = 5.4 Hz), 50.5, 26.8, 13.0.

^{31}P NMR (162 MHz, D_2O) δ = -7.8 (d, *J* = 21.5 Hz, 1P), -10.6 (d, *J* = 21.5 Hz, 1P).

HRMS (ESI, *m/z*): calcd. for $C_6H_{12}N_3O_7P_2$ $[M-H]^-$: 300.0156, found 300.0155.

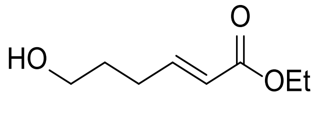

Ethyl (*E*)-6-hydroxyhex-2-enoate (16a)

1,4-Butanediol (492 µL, 5.55 mmol), (carbethoxymethylene)triphenylphosphorane (4.8 g, 13.9 mmol) and MnO_2 (9.6 g, 111 mmol) in DCM (40 mL) were reacted according to general procedure D. Purification by flash chromatography (55:45 hexane/EtOAc) afforded the known ester (590 mg, 67%) as pale orange oil.

Spectroscopic data were in agreement with the literature[3].

^{1}**H NMR** (400 MHz, $CDCl_3$) δ = 6.98 (dt, *J* = 15.7, 7.0 Hz, 1H), 5.85 (dt, *J* = 15.6, 1.6 Hz, 1H), 4.19 (q, *J* = 7.1 Hz, 2H), 3.68 (t, *J* = 6.4 Hz, 2H), 2.36 – 2.26 (m, 2H), 1.74 (p, *J* = 7.7 Hz, 2H), 1.41 (s, 1H), 1.29 (t, *J* = 7.1 Hz, 3H).

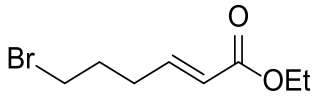

Ethyl (*E*)-6-bromohex-2-enoate (16b)

16a (590 mg, 3.73 mmol) in DCM (6 mL) was treated with PPh_3 (1.18 g, 4.48 mmol) and CBr_4 (1.48 g, 4.48 mmol) according to general procedure E. Purification by flash chromatography (9:1 hexane/EtOAc) afforded the desired compound (785 mg, 95%) as colourless oil.

Spectroscopic data were in agreement with the literature[12].

^{1}**H NMR** (400 MHz, $CDCl_3$) δ = 6.91 (dt, *J* = 15.7, 7.0 Hz, 1H), 5.88 (dt, *J* = 15.7, 1.6 Hz, 1H), 4.19 (q, *J* = 7.1 Hz, 2H), 3.42 (t, *J* = 6.5 Hz, 2H), 2.43 – 2.33 (m, 2H), 2.08 – 1.97 (m, 2H), 1.29 (t, *J* = 7.1 Hz, 3H).

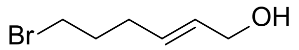

(*E*)-6-Bromohex-2-en-1-ol (16c)

16b (785 mg, 3.55 mmol) in THF (7 mL) was treated with DIBAL-H (8.9 mL) according to general procedure A, to give the corresponding unsaturated alcohol (572 mg, 90%) as a colourless oil, which was directly used in the next step without further purification.

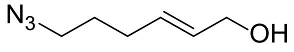

(*E*)-6-Azidohex-2-en-1-ol (16d)

16c (572 mg, 3.19 mmol), sodium iodide (48 mg, 0.32 mmol) and sodium azide (415 mg, 4.38 mmol) in DMSO (5 mL) were reacted according to general procedure F. Purification by flash chromatography (4:6 hexane/Et_2O) afforded the desired azide (279 mg, 62%) as a pale-yellow oil.

***R**f* 0.4 (4:6 hexane/Et_2O);

^{1}**H NMR** (400 MHz, CD_2Cl_2) δ = 5.67 – 5.61 (m, 2H), 4.07 – 4.01 (m, 2H), 3.28 (t, *J* = 6.9 Hz, 2H), 2.18 – 2.05 (m, 3H), 1.67 (p, *J* = 7.7 Hz, 2H).

^{13}**C NMR** (101 MHz, CD_2Cl_2) δ = 131.0, 130.9, 63.5, 51.2, 29.6, 28.7.

MS (ESI, *m/z*): calcd. for $C_6H_{11}N_3ONa$ $[M+Na]^+$: 164.09, found 164.1.

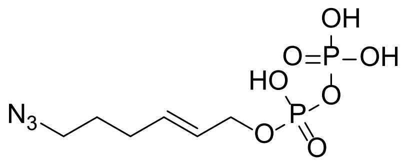

(*E*)-6-Azidohex-2-en-1-yl trihydrogen diphosphate (16)

16d (106 mg, 0.75 mmol) in acetonitrile (2 mL) was treated with PPh_3 (236 mg, 0.90 mmol), CBr_4 (299 mg, 0.90 mmol) and tris(tetrabutylammonium) hydrogen pyrophosphate (1.02 g, 1.13 mmol) according to general procedure C. Purification by flash chromatography (4:3:3 isopropanol/acetonitrile/0.1 M ammonium bicarbonate) afforded the desired compound (107 mg, 41%).

R_f 0.5 (4:3:3 isopropanol/acetonitrile/0.1 M ammonium bicarbonate);

^{1}H NMR (400 MHz, D_2O) δ = 5.86 (dt, *J* = 15.5, 6.7 Hz, 1H), 5.73 (dt, *J* = 15.5, 6.0 Hz, 1H), 4.41 (t, *J* = 6.5 Hz, 2H), 3.35 (t, *J* = 6.9 Hz, 2H), 2.22 – 2.12 (m, 2H), 1.72 (p, *J* = 7.1 Hz, 2H).

^{13}C NMR (101 MHz, D_2O) δ = 133.9, 126.6 (d, *J* = 7.6 Hz), 66.5 (d, *J* = 5.2 Hz), 50.5, 28.6, 27.3.

^{31}P NMR (162 MHz, D_2O) δ = -7.2 (d, *J* = 21.6 Hz, 1P), -10.6 (d, *J* = 21.5 Hz, 1P).

HRMS (ESI, *m/z*): calcd. for $C_6H_{12}N_3O_7P_2$ $[M-H]^-$: 300.0156, found 300.0155

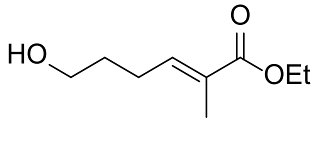

Ethyl (*E)*-6-hydroxy-2-methylhex-2-enoate (17a)

1,4-Butanediol (492 µL, 5.55 mmol), (carbethoxyethylidene)triphenylphosphorane (5.04 g, 13.9 mmol) and MnO_2 (9.6 g, 111 mmol) in DCM (40 mL) were reacted according to general procedure D. Purification by flash chromatography (6:4 hexane/EtOAc) afforded the known ester (543 mg, 57%) as pale-yellow oil.

Spectroscopic data were in agreement with the literature[3].

^{1}H NMR (400 MHz, $CDCl_3$) δ = 6.76 (tq, *J* = 7.5, 1.5 Hz, 1H), 4.19 (q, *J* = 7.1 Hz, 2H), 3.68 (t, *J* = 6.4 Hz, 2H), 2.33 – 2.22 (m, 2H), 1.84 (t, *J* = 1.2 Hz, 3H), 1.72 (d, *J* = 7.5 Hz, 2H), 1.45 (s, 1H), 1.29 (t, *J* = 7.1 Hz, 3H).

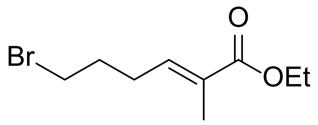

Ethyl (*E*)-6-bromo-2-methylhex-2-enoate (17b)

17a (543 mg, 3.15 mmol) in DCM (5 mL) was treated with PPh_3 (991 mg, 3.78 mmol) and CBr_4 (1.3 g, 3.78 mmol) according to general procedure E. Purification by flash chromatography (9:1 hexane/EtOAc) afforded the desired compound (666 mg, 90%) as colourless oil.

Spectroscopic data were in agreement with the literature[13].

^{1}H NMR (400 MHz, $CDCl_3$) δ = 6.70 (tq, *J* = 7.5, 1.5 Hz, 1H), 4.19 (q, *J* = 7.1 Hz, 2H), 3.42 (t, *J* = 6.5 Hz, 2H), 2.41 – 2.30 (m, 2H), 2.07 – 1.95 (m, 2H), 1.87 (d, *J* = 1.2 Hz, 3H), 1.30 (t, *J* = 7.1 Hz, 3H).

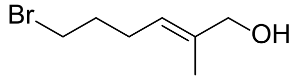

(*E*)-6-Bromo-2-methylhex-2-en-1-ol (17c)

17b (666 mg, 2.83 mmol) in THF (6 mL) was treated with DIBAL-H (7.1 mL) according to general procedure A, to give the corresponding unsaturated alcohol (530 mg, 97%) as a colourless oil, which was directly used in the next step without further purification.

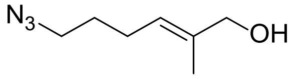

(*E*)-6-Azido-2-methylhex-2-en-1-ol (17d)

17c (530 mg, 2.74 mmol), sodium iodide (40 mg, 0.27 mmol) and sodium azide (357 mg, 4.49 mmol) in DMSO (5 mL) were reacted according to general procedure F. Purification by flash chromatography (5:5 hexane/Et_2O) afforded the desired azide (314 mg, 74%) as a pale-yellow oil.

R_f 0.3 (5:5 hexane/Et_2O);

^{1}H NMR (400 MHz, CD_2Cl_2) δ = 5.42 – 5.33 (m, 1H), 3.95 (s, 2H), 3.27 (t, *J* = 6.9 Hz, 2H), 2.18 – 2.07 (m, 2H), 2.01 – 1.93 (m, 1H), 1.71 – 1.59 (m, 5H).

^{13}C NMR (101 MHz, CD_2Cl_2) δ = 136.7, 124.2, 68.7, 51.3, 29.0, 25.0, 13.7.

MS (ESI, *m/z*): calcd. for $C_7H_{13}N_3ONa$ $[M+Na]^+$: 178.11, found 178.1.

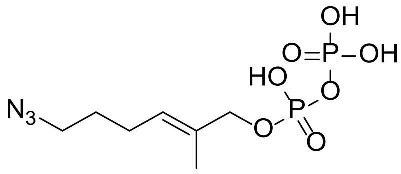

(*E*)-6-Azido-2-methylhex-2-en-1-yl trihydrogen diphosphate (17)

17d (117 mg, 0.75 mmol) in acetonitrile (2 mL) was treated with PPh_3 (236 mg, 0.90 mmol), CBr_4 (300 mg, 0.90 mmol) and tris(tetrabutylammonium) hydrogen pyrophosphate (1.02 g, 1.12 mmol) according to general procedure C. Purification by flash chromatography (4:3:3 isopropanol/acetonitrile/0.1 M ammonium bicarbonate) afforded the desired compound (110 mg, 40%).

R_f 0.5 (4:3:3 isopropanol/acetonitrile/0.1 M ammonium bicarbonate);

^{1}H NMR (400 MHz, D_2O) δ = 5.61 – 5.52 (m, 1H), 4.33 (d, *J* = 6.1 Hz, 2H), 3.34 (t, *J* = 6.9 Hz, 2H), 2.20 – 2.10 (m, 2H), 1.73 – 1.62 (m, 5H).

^{13}C NMR (101 MHz, D_2O) δ = 133.1 (d, *J* = 8.0 Hz), 127.1, 71.4 (d, *J* = 5.6 Hz), 50.6, 27.6, 24.1, 12.9.

^{31}P NMR (162 MHz, D_2O) δ = -7.7 (d, *J* = 21.4 Hz, 1P), -10.6 (d, *J* = 21.3 Hz, 1P).

HRMS (ESI, *m/z*): calcd. for $C_7H_{14}N_3O_7P_2$ $[M-H]^-$: 314.0312, found 314.0311

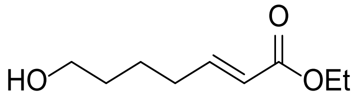

Ethyl (*E*)-7-hydroxyhept-2-enoate (18a)

1,5-Pentanediol (504 µL, 4.80 mmol), (carbethoxymethylene)triphenylphosphorane (4.01 g, 11.5 mmol) and MnO_2 (8.35 g, 96 mmol) in DCM (40 mL) were reacted according to general procedure D. Purification by flash chromatography (55:45 hexane/EtOAc) afforded the known ester (421 mg, 51%) as colourless oil.

Spectroscopic data were in agreement with the literature[3].

^{1}H NMR (400 MHz, $CDCl_3$) δ = 6.96 (dt, *J* = 15.7, 7.0 Hz, 1H), 5.83 (dt, *J* = 15.6, 1.6 Hz, 1H), 4.18 (q, *J* = 7.1 Hz, 2H), 3.66 (t, *J* = 6.1 Hz, 2H), 2.29 – 2.15 (m, 2H), 1.67 – 1.48 (m, 4H), 1.45 (s, 1H), 1.28 (t, *J* = 7.1 Hz, 3H).

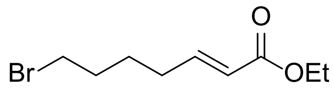

Ethyl (*E*)-7-bromohept-2-enoate (18b)

18a (421 mg, 2.44 mmol) in DCM (4 mL) was treated with PPh_3 (769 mg, 2.93 mmol) and CBr_4 (972 mg, 2.93 mmol) according to general procedure E. Purification by flash chromatography (95:5 hexane/EtOAc) afforded the desired compound (416 mg, 72%) as colourless oil.

Spectroscopic data were in agreement with the literature[14].

^{1}H NMR (400 MHz, $CDCl_3$) δ = 6.94 (dt, *J* = 15.6, 6.9 Hz, 1H), 5.84 (dt, *J* = 15.6, 1.6 Hz, 1H), 4.19 (q, *J* = 7.1 Hz, 2H), 3.41 (t, *J* = 6.7 Hz, 2H), 2.24 (dtd, *J* = 7.3, 7.2, 1.5 Hz, 2H), 1.95 – 1.83 (m, 2H), 1.69 – 1.57 (m, 2H), 1.29 (t, *J* = 7.1 Hz, 3H).

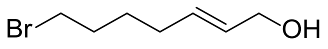

(*E*)-7-Bromohept-2-en-1-ol (18c)

18b (416 mg, 1.77 mmol) in THF (4 mL) was treated with DIBAL-H (4.4 mL) according to general procedure A, to give the corresponding unsaturated alcohol (331 mg, 97%) as a white solid, which was directly used in the next step without further purification.

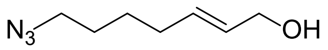

(*E*)-7-Azidohept-2-en-1-ol (18d)

18c (331 mg, 1.71 mmol), sodium iodide (26 mg, 0.17 mmol) and sodium azide (222 mg, 3.42 mmol) in DMSO (4 mL) were reacted according to general procedure F. Purification by flash chromatography (5:5 hexane/Et_2O) afforded the desired azide (230 mg, 71%) as a colourless oil.

Spectroscopic data were in agreement with the literature[15].

^{1}H NMR (400 MHz, CD_2Cl_2) δ = 5.71 – 5.58 (m, 2H), 4.03 (d, *J* = 4.4 Hz, 2H), 3.26 (t, *J* = 6.9 Hz, 2H), 2.12 – 2.02 (m, 3H), 1.66 – 1.54 (m, 2H), 1.54 – 1.38 (m, 2H).

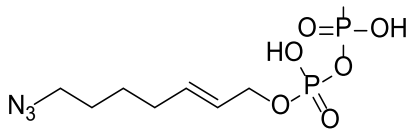

(*E*)-7-Azidohept-2-en-1-yl trihydrogen diphosphate (18)

18d (117 mg, 0.75 mmol) in acetonitrile (2 mL) was treated with PPh_3 (298 mg, 0.90 mmol), CBr_4 (236 mg, 0.90 mmol) and tris(tetrabutylammonium) hydrogen pyrophosphate (1.02 g,

1.13 mmol) according to general procedure C. Purification by flash chromatography (4:3:3 isopropanol/acetonitrile/0.1 M ammonium bicarbonate) afforded the desired compound (43 mg, 16%).

Rf 0.6 (4:3:3 isopropanol/acetonitrile/0.1 M ammonium bicarbonate);

^{1}H NMR (400 MHz, D_2O) δ = 5.91 (dt, *J* = 15.5, 6.6 Hz, 1H), 5.71 (dtt, *J* = 15.5, 6.3, 1.2 Hz, 1H), 4.40 (t, *J* = 6.4 Hz, 2H), 3.34 (t, *J* = 6.9 Hz, 2H), 2.21 – 2.04 (m, 2H), 1.69 – 1.58 (m, 2H), 1.55 – 1.43 (m, 2H).

^{13}C NMR (101 MHz, D_2O) δ = 135.2, 125.9 (d, *J* = 7.6 Hz), 66.7 (d, *J* = 5.0 Hz), 51.0, 30.9, 27.5, 25.3.

^{31}P NMR (162 MHz, D_2O) δ = -7.4 (d, *J* = 20.3 Hz, 1P), -10.5 (d, *J* = 21.1 Hz, 1P).

HRMS (ESI, *m/z*): calcd. for $C_7H_{14}N_3O_7P_2$ $[M-H]^-$: 314.0312, found 314.0312

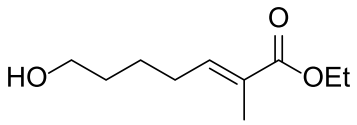

Ethyl (*E*)-7-hydroxy-2-methylhept-2-enoate (19a)

1,5-Pentanediol (504 µL, 4.80 mmol), (carbethoxyethylidene)triphenylphosphorane (4.17 g, 11.5 mmol) and MnO_2 (8.4 g, 96 mmol) in DCM (40 mL) were reacted according to general procedure D. Purification by flash chromatography (6:4 hexane/EtOAc) afforded the known ester (314 mg, 35%) as colourless oil.

Spectroscopic data were in agreement with the literature[3].

^{1}H NMR (400 MHz, $CDCl_3$) δ = 6.78 (tq, *J* = 7.4, 1.5 Hz, 1H), 4.21 (q, *J* = 7.1 Hz, 2H), 3.69 (t, *J* = 6.3 Hz, 2H), 2.29 – 2.18 (m, 2H), 1.86 (q, *J* = 1.0 Hz, 3H), 1.69 – 1.48 (m, 4H), 1.45 (s, 1H), 1.32 (t, *J* = 7.1 Hz, 3H).

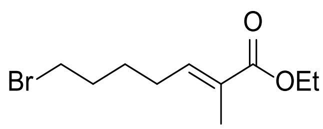

Ethyl (*E*)-7-bromo-2-methylhept-2-enoate (19b)

19a (314 mg, 1.69 mmol) in DCM (3 mL) was treated with PPh_3 (769 mg, 2.02 mmol) and CBr_4 (671 mg, 2.02 mmol) according to general procedure E. Purification by flash chromatography (95:5 hexane/EtOAc) afforded the desired compound (286 mg, 73%) as colourless oil.

***R**~f~* 0.3 (95:5 hexane/EtOAc);

^{1}H NMR (400 MHz, $CDCl_3$) δ = 6.73 (tq, *J* = 7.4, 1.5 Hz, 1H), 4.19 (q, *J* = 7.1 Hz, 2H), 3.42 (t, *J* = 6.7 Hz, 2H), 2.26 – 2.16 (m, 2H), 1.94 – 1.85 (m, 2H), 1.85 – 1.82 (m, 3H), 1.67 – 1.55 (m, 2H), 1.30 (t, *J* = 7.1 Hz, 3H).

^{13}C NMR (101 MHz, $CDCl_3$) δ = 168.1, 141.1, 128.5, 60.5, 33.4, 32.3, 27.8, 27.2, 14.3, 12.4.

MS (ESI, *m/z*): calcd. for $C_{10}H_{17}O_2Br$ $[M+H]^+$: 249.04, found 249.1, 251.0.

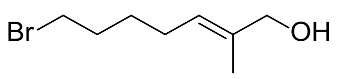

(*E*)-7-Bromo-2-methylhept-2-en-1-ol (19c)

19b (307 mg, 1.23 mmol) in THF (3 mL) was treated with DIBAL-H (3.1 mL) according to general procedure A, to give the corresponding unsaturated alcohol (250 mg, 98%) as a white solid, which was directly used in the next step without further purification.

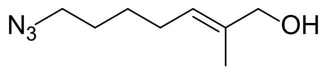

(*E*)-7-Azido-2-methylhept-2-en-1-ol (19d)

19c (250 mg, 1.21 mmol), sodium iodide (18 mg, 0.12 mmol) and sodium azide (157 mg, 2.41 mmol) in DMSO (3 mL) were reacted according to general procedure F. Purification by flash chromatography (5:5 hexane/Et2O) afforded the desired azide (164 mg, 80%) as a colourless oil.

***R**~f~* 0.3 (5:5 hexane/Et_2O);

^{1}H NMR (400 MHz, $CDCl_3$) δ = 5.35 (tq, *J* = 7.2, 1.4 Hz, 1H), 3.94 (s, 2H), 3.23 (t, *J* = 6.9 Hz, 2H), 2.09 (s, 1H), 2.07 – 1.99 (m, 2H), 1.62 (s, 3H), 1.60 – 1.53 (m, 2H), 1.47 – 1.35 (m, 2H).

^{13}C NMR (101 MHz, $CDCl_3$) δ = 135.4, 125.3, 68.6, 51.4, 28.4, 27.0, 26.6, 13.7.

MS (ESI, *m/z*): calcd. for $C_8H_{15}N_3ONa$ $[M+Na]^+$: 192.12, found 192.1.

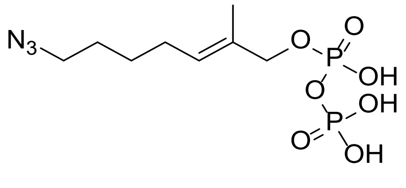

(*E*)-7-Azido-2-methylhept-2-en-1-yl trihydrogen diphosphate (19)

19d (108 mg, 0.64 mmol) in acetonitrile (2 mL) was treated with PPh_3 (203 mg, 0.77 mmol), CBr_4 (255 mg, 0.77 mmol) and tris(tetrabutylammonium) hydrogen pyrophosphate (866 mg, 0.96 mmol) according to general procedure C. Purification by flash chromatography (4:3:3 isopropanol/acetonitrile/0.1 M ammonium bicarbonate) afforded the desired compound (82 mg, 30%).

Rf 0.6 (4:3:3 isopropanol/acetonitrile/0.1 M ammonium bicarbonate);

^{1}H NMR (400 MHz, D_2O) δ = 5.59 (td, *J* = 7.3, 1.3 Hz, 1H), 4.34 (d, *J* = 6.0, 1.1 Hz, 2H), 3.34 (t, *J* = 6.9 Hz, 2H), 2.13 (dt, *J* = 7.2 Hz, 2H), 1.70 (s, 3H), 1.68 – 1.58 (m, 2H), 1.51 – 1.42 (m, 2H).

^{13}C NMR (101 MHz, D_2O) δ = 132.4 (d, *J* = 8.2 Hz), 128.5, 71.7 (d, *J* = 5.6 Hz), 51.1, 27.6, 26.5, 25.7, 13.0.

^{31}P NMR (162 MHz, D_2O) δ = -8.2 (d, *J* = 19.4 Hz, 1P), -10.6 (d, *J* = 21.3 Hz, 1P).

HRMS (ESI, *m/z*): calcd. for $C_8H_{16}N_3O_7P_2$ $[M-H]^-$: 328.0469, found 328.0468.

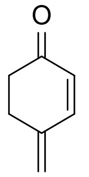

4-Methylenecyclohex-2-en-1-one (20b)

Triethylamine (TEA, 560 μL, 4.02 mmol, 2.5 equiv.) and methanesulfonyl chloride (187 μL, 2.42 mmol, 1.5 equiv.) were added dropwise to a solution of 4-(hydroxymethyl)-2-cyclohexene-1-one[16] (203 mg, 1.61 mmol, 1 equiv.) in DCM (2 mL) cooled to 0°C. The reaction was stirred for 1 hour at room temperature. The mixture was then cooled to 0°C, 1,8-diazabicyclo[5.4.0]undec-7-ene (DBU; 481 μL, 3.22 mmol, 2 equiv.) was added dropwise and the mixture was stirred overnight at room temperature. The reaction was quenched with sat. aq. $NaHCO_3$ solution and extracted with Et_2O. The organic layer was washed with sat. aq. $NaHCO_3$ solution and brine, dried over sodium sulfate and concentrated under reduced pressure. Purification by flash chromatography (9:1 hexane/EtOAc) afforded the title ketone (174 mg, 52%) as a colourless oil

Spectroscopic data were in agreement with the literature[17].

^{1}H NMR (400 MHz, $CDCl_3$) δ = 7.08 (d, *J* = 9.9 Hz, 1H), 5.96 (d, *J* = 9.9 Hz, 1H), 5.35 – 5.32 (m, 1H), 5.29 (s, 1H), 2.80 – 2.71 (m, 2H), 2.57 – 2.48 (m, 2H).

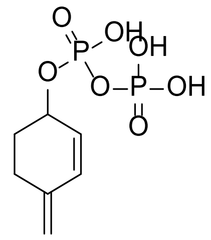

(±)-4-Methylenecyclohex-2-en-1-yl trihydrogen diphosphate (20)

Sodium borohydride (35 mg, 0.92 mmol, 1.1 equiv.) was added portion wise to a solution of **(20b)** (90 mg, 0.83 mmol, 1 equiv.) and cerium(III) chloride heptahydrate (343, 0.92 mmol, 1.1 equiv.) in methanol (2 mL) at 0 °C and the reaction was stirred for 2 hours at room temperature. The reaction was diluted with Et_2O, quenched with sat. aq. NH_4Cl solution and extracted with Et_2O. The combined organic layers were washed with brine, filtered over sodium sulfate and concentrated under reduced pressure. The crude product (77 mg, 85%) was directly used in the next step without further purification.

The crude alcohol (77 mg, 0.70 mmol, 1 equiv.) in DCM (2 mL) was treated with a 1.5 M solution of thionyl chloride/benzotriazole/DCM (580 µL, 0.87 mmol, 1.25 equiv.), according to the procedure of Chaudhari[18]. After 10 minutes, the reaction was diluted with Et_2O, filtered, and washed with water and 1M NaOH aqueous solution. The organic layer was washed with brine, dried over sodium sulfate and concentrated under reduced pressure. The crude chloride was directly used in the next step without further purification.

The freshly prepared chloride was subsequently dissolved in acetonitrile (2 mL) and treated with tris(tetrabutylammonium) hydrogen pyrophosphate (947 mg, 1.05 mmol) according to general procedure B. Purification by flash chromatography (4:3:3 isopropanol/acetonitrile/0.1 M ammonium bicarbonate) afforded the desired compound (85 mg, 38%).

***R**$_f$* 0.65 (4:3:3 isopropanol/acetonitrile/0.1 M ammonium bicarbonate);

^{1}H NMR (400 MHz, D_2O) δ = 6.33 (d, *J* = 10.0 Hz, 1H), 6.01 (dd, *J* = 10.0, 3.2 Hz, 1H), 4.98 (s, 2H), 2.59 – 2.48 (m, 1H), 2.46 – 2.33 (m, 1H), 2.15 – 2.03 (m, 1H), 1.91 – 1.79 (m, 1H).

^{13}C NMR (101 MHz, D_2O) δ = 142.5, 131.5, 130.4 (d, *J* = 4.9 Hz), 112.8, 70.3 (d, *J* = 5.4 Hz), 29.8 (d, *J* = 3.9 Hz), 26.4.

^{31}P NMR (162 MHz, D_2O) δ = -7.23 (d, *J* = 21.7 Hz, 1P), -11.03 (d, *J* = 21.6 Hz, 1P).

HRMS (ESI, *m/z*): calcd. for $C_7H_{11}O_7P_2$ $[M-H]^-$: 268.9985, found 268.9990.

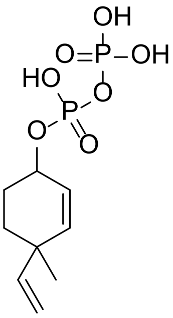

4-Methyl-4-vinylcyclohex-2-en-1-yl trihydrogen diphosphate (21)

Sodium borohydride (99 mg, 2.60 mmol, 1.1 equiv.) was added portion wise to a solution of **(±)**4-methyl-4-vinylcyclohex-2-enone[16] (327 mg, 2.37 mmol, 1 equiv.) and cerium(III) chloride heptahydrate (969, 2.60 mmol, 1.1 equiv.) in methanol (3 mL) at 0 °C and the reaction was stirred for 2 hour at room temperature. The reaction was diluted with Et_2O, quenched with sat. aq. NH_4Cl solution and extracted with Et_2O. The combined organic layers were washed with brine, filtered over sodium sulfate and concentrated under reduced pressure. The crude product (325 mg, 99%) was directly used in the next step without further purification.

The crude alcohol (105 mg, 0.77 mmol, 1 equiv.) in DCM (1 mL) was treated with a 1.5 M solution of thionyl chloride/benzotriazole/DCM (633 µL, 0.95 mmol, 1.25 equiv.), according to the procedure of Chaudhari[18]. After 10 minutes, the reaction was diluted with Et_2O, filtered, and washed with water and 1M NaOH aqueous solution. The organic layer was washed with brine, dried over sodium sulfate and concentrated under reduced pressure. The crude chloride was directly use in the next step without further purification.

The freshly prepared chloride was subsequently dissolved in acetonitrile (3 mL) and treated with tris(tetrabutylammonium) hydrogen pyrophosphate (1.39 g, 1.54 mmol) according to general procedure B. Purification by flash chromatography (4:3:3 isopropanol/acetonitrile/0.1 M ammonium bicarbonate) afforded the desired compound (111 mg, 42%) as a 1:1 mixture of diastereoisomers which were not separated.

R_f 0.65 (4:3:3 isopropanol/acetonitrile/0.1 M ammonium bicarbonate);

^{1}H NMR (400 MHz, D_2O) δ = 5.97 – 5.79 (m, 1H), 5.71 (d, *J* = 10.1 Hz, 0.6H), 5.64 (d, *J* = 10.1 Hz, 0.3H), 5.41 (t, *J* = 16.0, 0.3H), 5.13 – 4.92 (m, overlapped with solvent signal), 4.69 – 4.65 (m, overlapped with solvent signal), 2.00 (m, 0.4H), 1.87 (m, 1.3H), 1.74 – 1.46 (m, 2.4H), 1.08 (m, 3H).

^{13}C NMR (101 MHz, D_2O) δ = 146.8, 146.3, 138.8, 137.7, 127.6, 126.4 (d, *J* = 5.2 Hz), 112.4, 112.0, 71.2, 69.4 (d, *J* = 5.7 Hz), 37.9, 37.8, 32.0, 30.4, 26.9, 26.7, 26.4, 26.0.

^{31}P NMR (162 MHz, D_2O) δ = -6.9 (d, *J* = 21.4 Hz, 1P), -10.6 – -11.1 (m, 1P).

HRMS (ESI, *m/z*): calcd. for $C_9H_{15}O_7P_2$ $[M-H]^-$: 297.0298, found 297.0297.

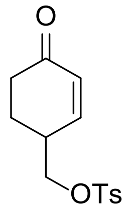

(±)-(4-Oxocyclohex-2-en-1-yl)methyl 4-methylbenzenesulfonate (22b)

4-Toluenesulfonyl chloride (841 mg, 4.41 mmol, 1.5 equiv.) was added to a solution of (±)-4-(hydroxymethyl)-2-cyclohexene-1-one[16] (371 mg, 2.94 mmol, 1 equiv.), DMAP (36 mg, 0.29 mmol, 0.1 equiv.) and triethylamine (615 µL, 4.41 mmol, 1.5 equiv.) in DCM (4 mL) at 0 °C and the mixture was stirred at room temperature for three hours. The reaction was diluted with Et_2O, quenched with sat. aq. NaHCO3 solution and extracted with Et_2O. The combined organic layers were washed with brine, filtered over sodium sulfate and concentrated under reduced pressure. The residue was purified by flash chromatography (6:4 hexane/EtOAc) to afford the desired tosylate (792 mg, 96%) as a white solid.

Spectroscopic data are in accordance with the literature, and the tosylate is unstable under air at room temperature[19].

^{1}H NMR (400 MHz, $CDCl_3$) δ = 7.86 – 7.76 (m, 2H), 7.43 – 7.33 (m, 2H), 6.74 (ddd, *J* = 10.3, 2.7, 1.5 Hz, 1H), 6.04 (ddd, *J* = 10.2, 2.6, 0.8 Hz, 1H), 4.08 – 3.99 (m, 2H), 2.91 – 2.75 (m, 1H), 2.52 – 2.46 (m, 1H), 2.47 (s, 3H), 2.42 – 2.29 (m, 1H), 2.15 – 2.05 (m, 1H), 1.89 – 1.70 (m, 1H).

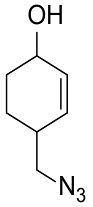

4-(Azidomethyl)cyclohex-2-en-1-ol (22c)

Sodium borohydride (118 mg, 3.11 mmol, 1.1 equiv.) was added portion wise to a solution of tosylate **22b** (792 mg, 2.83 mmol, 1 equiv.) and cerium(III) chloride heptahydrate (1.16 g, 3.11 mmol, 1.1 equiv.) in methanol (8 mL) at 0 °C and the reaction was stirred for 1 hour at room temperature. The reaction was diluted with Et_2O, quenched with sat. aq. NH_4Cl solution and extracted with Et_2O. The combined organic layers were washed with brine, filtered over sodium

sulfate and concentrated under reduced pressure. The crude product (726 mg, 91%) was directly used in the next step without further purification.

Sodium azide (334 mg, 5.14 mg, 2 equiv.) was added portion wise to a solution of tosylate (726 mg, 2.57 mmol, 1 equiv.) in DMSO (8 mL) and the mixture was heated at 45 °C overnight. The mixture was cooled to room temperature, diluted with water and extracted with Et_2O. The combined organic layers were washed with brine, dried over sodium sulfate and concentrated under reduced pressure. Purification by flash chromatography (5:5 hexane/EtOAc) afforded the desired azide (370 mg, 94%) as colourless oil, as a mixture of diastereoisomers which were not separated (diastereomeric ratio 1:0.4, as determined by NMR).

R_f 0.4 (6:4 hexane/EtOAc);

^{1}H NMR (400 MHz, $CDCl_3$) δ = 5.94 – 5.86 (m, 0.4H), 5.86 – 5.77 (m, 1H), 5.73 (dd, *J* = 10.1, 2.6 Hz, 0.4H), 5.66 (d, *J* = 10.2 Hz, 1H), 4.27 – 4.20 (m, 1H), 4.19 – 4.14 (m, 0.4H), 3.27 (d, *J* = 6.9 Hz, 0.8H), 3.24 – 3.15 (m, 2H), 2.45 – 2.34 (m, 1H), 2.34 – 2.22 (m, 0.4H), 2.14 – 2.03 (m, 1H), 1.97 – 1.85 (m, 1H), 1.84 – 1.65 (m, 2.2H), 1.63 – 1.43 (m, 1.4H), 1.43 – 1.22 (m, 1.4H).

^{13}C NMR (101 MHz, $CDCl_3$) δ =133.1, 131.3, 130.0, 66.6, 64.3, 56.1, 55.9, 36.0 (2C), 31.3, 29.8, 24.6, 21.9.

MS (ESI, *m/z*): calcd. for $C_7H_{11}N_3O$ $[M+Na]^+$: 176.09, found 176.1

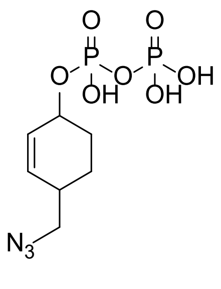

4-(Azidomethyl)cyclohex-2-en-1-yl trihydrogen diphosphate (22)

Compound **22c** (109 mg, 0.71 mmol, 1 equiv.) in DCM (1 mL) was treated with a 1.5 M solution of thionyl chloride/benzotriazole/DCM (593 µL, 0.89 mmol, 1.25 equiv.), according to the procedure of Chaudhari[18]. After 10 minutes, the reaction was diluted with Et_2O, filtered, and washed with water and 1M NaOH aqueous solution. The organic layer was washed with brine, dried over sodium sulfate and concentrated under reduced pressure. The crude chloride was directly used in the next step without further purification.

The freshly prepared chloride was subsequently dissolved in acetonitrile (3 mL) and treated with tris(tetrabutylammonium) hydrogen pyrophosphate (1.28 g, 1.42 mmol) according to general procedure B. Purification by flash chromatography (4:3:3 isopropanol/acetonitrile/0.1

M ammonium bicarbonate) afforded the desired compound (136 mg, 53%) as a 1:0.4 mixture of diastereoisomers which were not separated.

Rf 0.6 (4:3:3 isopropanol/acetonitrile/0.1 M ammonium bicarbonate);

^{1}H NMR (400 MHz, D_2O) δ = 6.03 – 5.61 (m, 2.5H), 5.31 (m, 0.1H), 3.56 (dd, *J* = 12.6, 5.5 Hz, 0.6H), 3.33 – 3.12 (m, 2H), 2.35 (m, 0.3H), 2.25 (t, *J* = 7.2 Hz, 0.5H), 2.15 – 1.78 (m, 3H), 1.76 – 1.21 (m, 3H).

^{13}C NMR (101 MHz, D_2O) δ = 132.9, 131.6, 130.6 (d, *J* = 5.0 Hz), 128.9 (d, *J* = 5.2 Hz), 126.1 (d, *J* = 2.1 Hz), 71.1 (d, *J* = 5.5 Hz), 69.5 (d, *J* = 6.0 Hz), 68.9 (d, *J* = 5.6 Hz), 55.5, 55.3, 52.4, 37.8 (d, *J* = 5.9 Hz), 35.1, 28.7, 27.6 (d, *J* = 3.6 Hz), 23.9, 23.7, 21.3, 20.4.

^{31}P NMR (162 MHz, D_2O) δ = -6.6 – -7.4 (m, 1P), -10.6 – -11.3 (m, 1P).

HRMS (ESI, *m/z*): calcd. for $C_7H_{12}N_3O_7P_2$ $[M-H]^-$: 312.0156, found 312.0155.

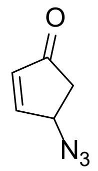

(±)-4-Azidocyclopent-2-en-1-one (23b)

Sodium azide (403 mg, 6.2 mmol, 2 equiv.) was added to a solution of 4-bromocyclopent-2-enone[20] (499 mg, 3.1 mmol, 1 equiv.) and NaI (45 mg, 0.3 mmol, 0.1 equiv.) in DMSO (10 mL) at 0 °C and the mixture was stirred for 30 minutes at room temperature. The mixture was diluted with water and extracted with Et_2O. The combined organic layers were washed with brine, dried over sodium sulfate and concentrated under reduced pressure. Purification by flash chromatography (8:2 hexane/EtOAc) afforded the desired azide (300 mg, 79%) as pale yellow oil.

Spectroscopic data were in agreement with the literature[21].

^{1}H NMR (400 MHz, $CDCl_3$) δ = 7.54 (dd, *J* = 5.7, 2.5 Hz, 1H), 6.35 (dd, *J* = 5.7, 1.5 Hz, 1H), 4.71 – 4.63 (m, 1H), 2.78 (dd, *J* = 18.7, 6.6 Hz, 1H), 2.36 (dd, *J* = 18.7, 2.4 Hz, 1H).

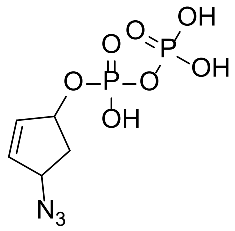

4-Azidocyclopent-2-en-1-yl trihydrogen diphosphate (23)

Sodium borohydride (101 mg, 2.68 mmol, 1.1 equiv.) was added portion wise to a solution of **(23b)** (300 mg, 2.40 mmol, 1 equiv.) and cerium(III) chloride heptahydrate (998, 2.68 mmol, 1.1 equiv.) in methanol (5 mL) at 0 °C and the reaction was stirred for 2 hour at room temperature. The reaction was diluted with Et_2O, quenched with sat. aq. NH_4Cl solution and extracted with Et_2O. The combined organic layers were washed with brine, filtered over sodium sulfate and concentrated under reduced pressure. The crude product (270 mg, 88%) was directly used in the next step without further purification.

The crude alcohol (101 mg, 0.81 mmol, 1 equiv.) in DCM (2 mL) was treated with a 1.5 M solution of thionyl chloride/benzotriazole/DCM (675 µL, 1.01 mmol, 1.25 equiv.), according to the procedure of Chaudhari[18]. After 10 minutes, the reaction was diluted with Et_2O, filtered, and washed with water and 1M NaOH aqueous solution. The organic layer was washed with brine, dried over sodium sulfate and concentrated under reduced pressure. The crude chloride was directly use in the next step without further purification.

The freshly prepared chloride was subsequently dissolved in acetonitrile (2.5 mL) and treated with tris(tetrabutylammonium) hydrogen pyrophosphate (1.1 g, 1.22 mmol) according to general procedure B. Purification by flash chromatography (4:3:3 isopropanol/acetonitrile/0.1 M ammonium bicarbonate) afforded the desired compound (123 mg, 45% over two steps) as a 1:1 mixture of diastereoisomers which were not separated.

R_f 0.3 (4:3:3 isopropanol/acetonitrile/0.1 M ammonium bicarbonate);

^{1}H NMR (400 MHz, D_2O) δ = 6.21 (d, *J* = 5.6 Hz, 1H), 6.18 (d, *J* = 5.6 Hz, 1H), 6.09 (dd, *J* = 5.5, 2.7 Hz, 1H), 6.03 (dd, *J* = 5.6, 2.2 Hz, 1H), 5.98 (dd, *J* = 5.5, 2.1 Hz, 1H), 5.74 (dq, *J* = 6.3, 2.2 Hz, 1H), 5.38 (s, 1H), 5.15 (s, 1H), 4.43 (s, 1H), 4.31 (d, *J* = 6.8 Hz, 1H), 2.91 – 2.65 (m, 1H), 2.48 (d, *J* = 17.9 Hz, 1H), 2.30 (ddd, *J* = 14.9, 7.2, 2.8 Hz, 1H), 2.20 (ddd, *J* = 14.8, 6.6, 3.4 Hz, 1H), 1.78 (dt, *J* = 14.7, 3.8 Hz, 1H).

^{13}C NMR (101 MHz, D_2O) δ = 136.6 (d, *J* = 4.6 Hz), 136.1 (d, *J* = 5.0 Hz), 135.7, 133.7, 132.9, 126.1, 79.9 (t, *J* = 4.9, 4.1 Hz), 78.4 (d, *J* = 4.7 Hz), 72.3 (d, *J* = 5.4 Hz), 65.9, 64.5, 39.3 (d, *J* = 3.5 Hz), 38.3 (d, *J* = 3.6 Hz), 37.8 (d, *J* = 2.3 Hz).

^{31}P NMR (162 MHz, D_2O) δ = -6.9 (br s, 1P), -11.3 (br s, 1P)

HRMS (ESI, *m/z*): calcd. for $C_5H_8N_3O_7P_2$ $[M-H]^-$: 283.9843, found 283.9848.

Cofactor	Product Molecular Formula	Predicted Mass [M - H]$^-$	Observed Mass	Delta (ppm)
**4**	$C_3H_8O_7P_2$	216.9672	216.9678	2.7654
**5**	$C_4H_{10}O_7P_2$	230.9829	230.9834	2.1647
**6**	$C_6H_{12}O_7P_2$	256.9985	256.9987	0.7782
**7**	$C_{10}H_{18}O_7P_2$	311.0455	311.0454	-0.3215
**11**	$C_5H_8O_7P_2$	240.9672	240.9672	0.0000
**12**	$C_5H_8O_7P_2$	240.9672	240.9669	-1.2450
**13**	$C_7H_{12}O_7P_2$	268.9985	268.9986	0.3717
**14**	$C_5H_{11}N_3O_7P_2$	285.9999	285.9999	0.0000
**15**	$C_6H_{13}N_3O_7P_2$	300.0156	300.0155	-0.3333
**16**	$C_6H_{13}N_3O_7P_2$	300.0156	300.0155	-0.3333
**17**	$C_7H_{15}N_3O_7P_2$	314.0312	314.0311	-0.3184
**18**	$C_7H_{15}N_3O_7P_2$	314.0312	314.0312	0.0000
**19**	$C_8H_{17}N_3O_7P_2$	328.0469	328.0468	-0.3048
**20**	$C_7H_{12}O_7P_2$	268.9985	268.9990	1.8587
**21**	$C_9H_{16}O_7P_2$	297.0298	297.0297	-0.3367
**22**	$C_7H_{13}N_3O_7P_2$	312.0156	312.0155	-0.3205
**23**	$C_5H_9N_3O_7P_2$	283.9843	283.9848	1.7607

Table S1 HRMS data summary for new synthetic alkyl pyrophosphates

1.5 Peptide synthesis

Peptides were prepared exploiting the 9-fluorenylmethoxycarbonyl (Fmoc) solid phase microwave assisted peptide synthesis (SPPS) on a Liberty Blue^{TM} Automated Microwave Peptide Synthesizer (CEM). Coupling agents used were 1 M *N,N'*-diisopropylcarbodiimide (DIC) solution in dimethylformamide (DMF) and 1 M ethyl cyano(hydroxyimino)acetate (Oxyma) pure as additive. Fmoc deprotection was performed by a 20% piperidine solution in DMF.

Crude peptides were purified by semi-preparative HPLC.

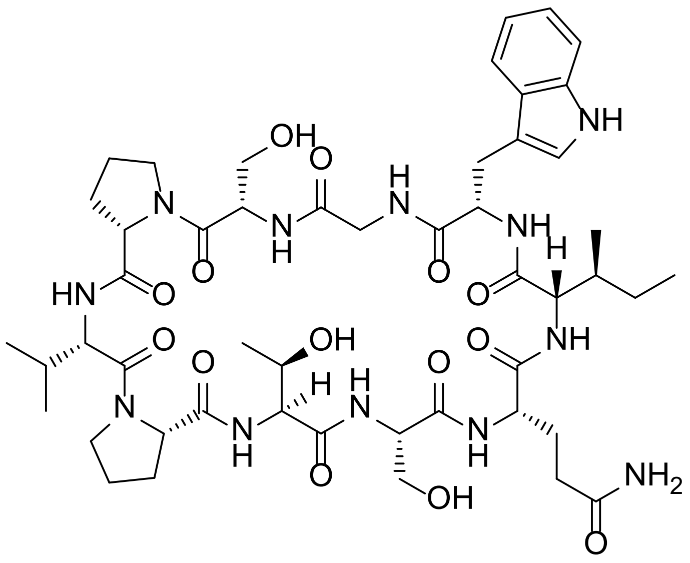

Cyclo-[TSQIWGSPVP] (24)

Compound **24** was synthesised by chemical cyclization of protected linear peptide NH_2-SPVPTSQIWG-COOH.

Protected linear SPVPTSQIWG was synthesised according to general methods using H-Gly-2-ClTrt resin (pre-loaded resin). The following modifications were applied: *N,N*-Diisopropylethylamine (DIPEA) 0.1 M was added to Oxyma pure solution, and the coupling cycle was set to 60 °C/5 minutes. Cleavage of the protected peptide from the resin was performed by treating the resin with 2,2,2-trifluoroethanol/dichloromethane 2:8 for one hour and repeated three times in total. The cleavage cocktail was removed under a stream of air and the peptide was precipitated by addition of cold diethyl ether and hexane.

Chemical cyclization was performed as follows: a solution of *N,N*-Diisopropylethylamine (56 µL, 320 µmol, 5 equiv.) and crude protected SPVPTSQIWG (101 mg, 64 µmol, 1 equiv.) in dichloromethane (13 mL) was treated with ((7-azabenzotriazol-1-yloxy)tripyrrolidinophosphonium hexafluorophosphate) (PyAOP) (100 mg, 192 µmol, 3 equiv.). The mixture was stirred for 40 hours at room temperature, before solvent removal under reduced pressure. Side chains protecting groups were removed with a cleavage solution of 95% trifluoroacetic acid (TFA), 2.5% triisopropylsilane (TIS) and 2.5% of water for 3 h at room temperature. TFA was removed in a stream of air and the peptide precipitated by addition of cold diethyl ether. The crude peptide was purified by semi-preparative HPLC.

Column ACE 5 C18-HL 250x10.0 mm, solvent A water + 0.1% TFA, B acetonitrile + 0.1% TFA; flow 4.7 mL/min. Gradient: 0 min 5% B; 15 min 100% B.

T_R = 9.5 minutes.

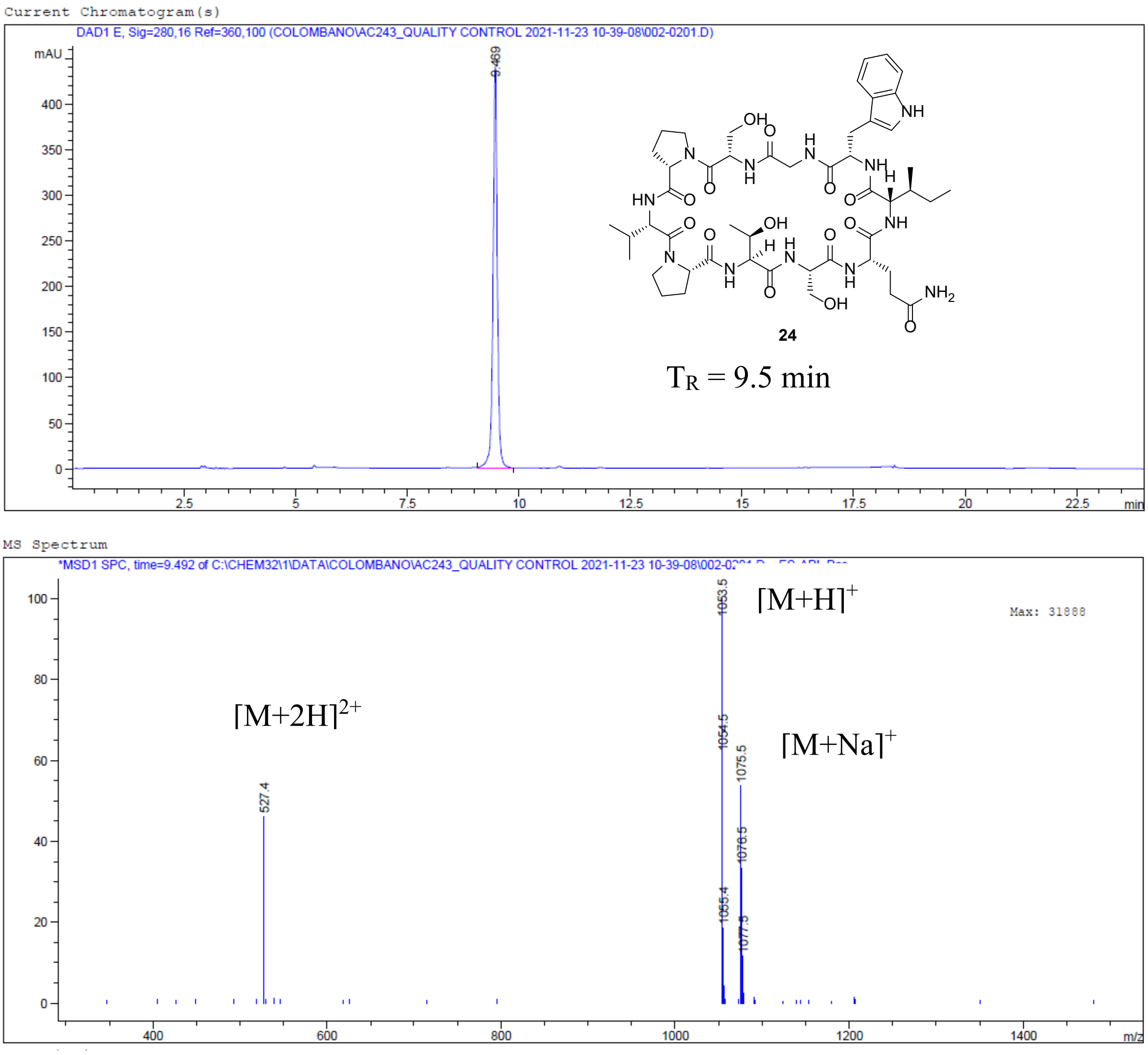

Figure S1 LC-MS for purified cyclo-[TSQIWGSPVP] (**24**)

2 Biochemistry

2.1 Expression and purification of AcyF

AcyF was recombinantly produced in in *E.coli* BL21(DE3) and purified as previously described[22].

2.2 Enzymatic reaction general protocol

Each reaction mixture contained 100 µM substrate, 1% dimethyl sulfoxide (DMSO), 1 mM pyrophosphate cofactor, 12 mM $MgCl_2$, 20 µM enzyme in a buffer containing 150 mM NaCl, 10 mM HEPES (pH 7.5), and 3 mM TCEP. All reaction mixtures were incubated at 37 °C for 72 hours and analysed by LC−HRMS.

Samples were analysed with LC-HRMS (Q-TOF MS, Maxis II, Bruker Daltonics, United States); Phenomenex Kinetex XB-C18 (2.6 µm, 100 x 2.1mm); solvent A (water + 0.1% formic acid), solvent B (acetonitrile + 0.1% formic acid); a linear gradient of 5–100% B over 15 min at a flow rate of 1 mL/min was used.

2.3 LC-HRMS of enzymatic reactions and corresponding negative controls

Cofactor	Product Molecular Formula	Predicted Mass [M + H]$^+$	Observed Mass	Delta (ppm)
**1**	$C_{54}H_{80}N_{12}O_{14}$	1121.5990	1121.6022	2.8531
**2**	$C_{54}H_{82}N_{12}O_{14}$	1123.6146	-	-
**3**	$C_{53}H_{79}N_{12}O_{14}$	1107.5833	1107.5859	2.3475
**4**	$C_{52}H_{76}N_{12}O_{14}$	1093.5677	-	-
**5**	$C_{53}H_{78}N_{12}O_{14}$	1107.5833	-	-
**6**	$C_{55}H_{80}N_{12}O_{14}$	1133.5990	1133.6018	2.4700
**7**	$C_{59}H_{86}N_{12}O_{14}$	1187.645922	-	-
**8**	$C_{54}H_{80}N_{12}O_{14}$	1121.5990	1121.5997	0.6241
**9**	$C_{55}H_{82}N_{12}O_{14}$	1135.6146	-	-
**10**	$C_{52}H_{74}N_{12}O_{14}$	1091.5520	-	-
**11**	$C_{54}H_{76}N_{12}O_{14}$	1117.5677	-	-
**12**	$C_{54}H_{76}N_{12}O_{14}$	1117.5677	-	-
**13**	$C_{56}H_{80}N_{12}O_{14}$	1145.598972	-	-
**14**	$C_{54}H_{79}N_{15}O_{14}$	1162.6003	-	-
**15**	$C_{55}H_{81}N_{15}O_{14}$	1176.6160	-	-
**16**	$C_{55}H_{81}N_{15}O_{14}$	1176.6160	-	-
**17**	$C_{56}H_{83}N_{15}O_{14}$	1190.6317	-	-
**18**	$C_{56}H_{83}N_{15}O_{14}$	1190.6317	-	-
**19**	$C_{57}H_{85}N_{15}O_{14}$	1204.6473	-	-
**20**	$C_{56}H_{80}N_{12}O_{14}$	1145.5990	1145.5999	0.8101
**20**	$C_{56}H_{80}N_{12}O_{14}$	1145.5990	1145.5975	-1.2849
**20**	$C_{56}H_{80}N_{12}O_{14}$	1145.5990	1145.5969	-1.8087
**20**	$C_{56}H_{80}N_{12}O_{14}$	1145.5990	1145.5979	-0.9358
**20**	$C_{56}H_{80}N_{12}O_{14}$	1145.5990	1145.5991	0.1117
**20**	$C_{56}H_{80}N_{12}O_{14}$	1145.5990	1145.5991	0.1117
**21**	$C_{59}H_{86}N_{12}O_{14}$	1187.6459	-	-
**22***	$C_{56}H_{80}N_{12}O_{14}$	1145.5990	1145.6005	1.3338
**22***	$C_{56}H_{80}N_{12}O_{14}$	1145.5990	1145.6016	2.2940
**22***	$C_{56}H_{80}N_{12}O_{14}$	1145.5990	1145.5990	0.0244
**23**	$C_{54}H_{77}N_{15}O_{14}$	1160.5847	-	-

*Predicted mass for [M-HN3+H]+

Table S2 HRMS confirmation of AcyF-Catalysed reactions with **24**. Reaction with **20** yielded 6 products while reaction with **22** afforded 3 products.

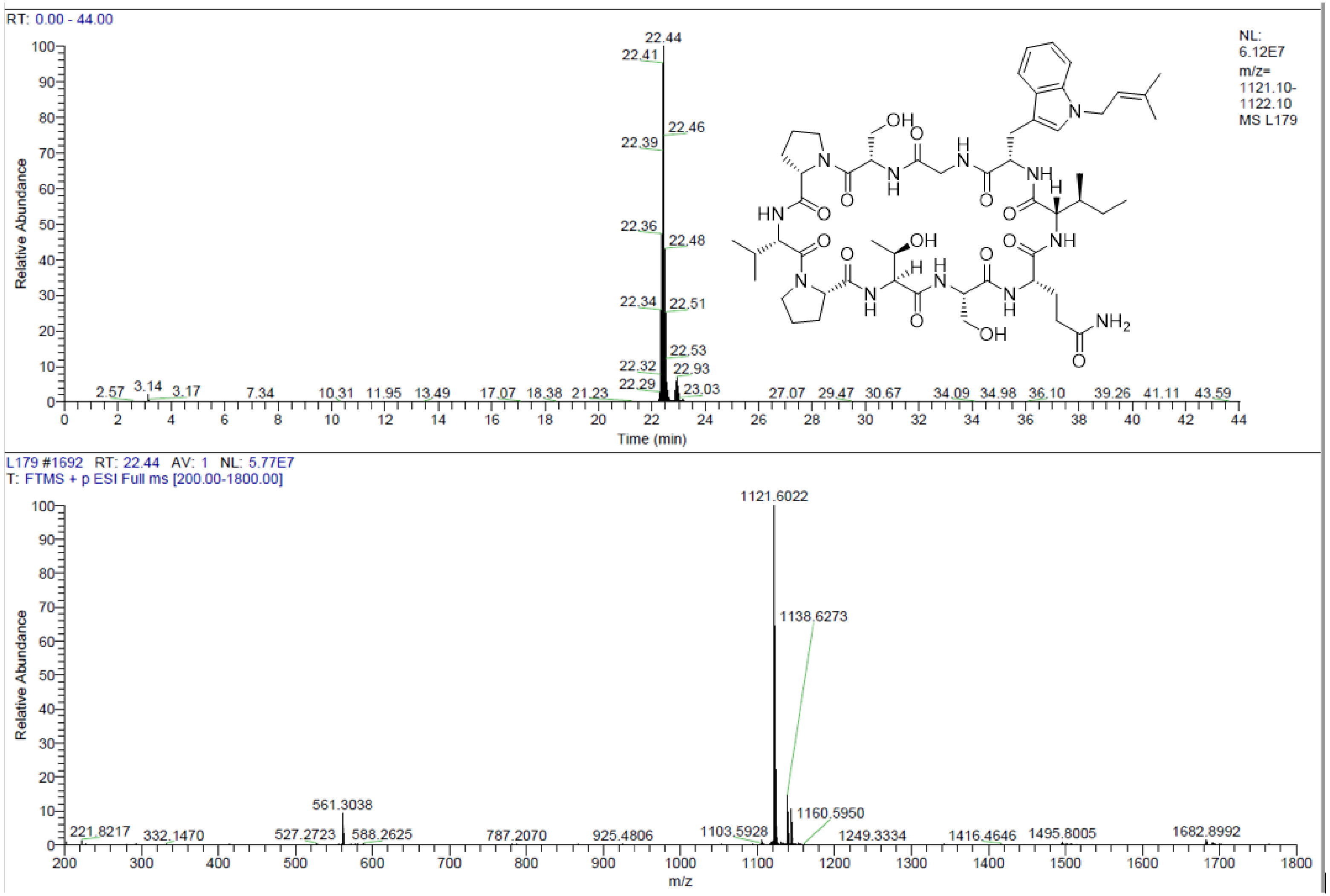

Figure S2 LC-HRMS (EIC) of reaction between **24** and **1**. Top diagram shows extracted ion current (± 0.5 Da) for the expected product ([M+H]$^+$). Bottom diagram shows HRMS spectrum of the expected product (T_R 22.4 min).

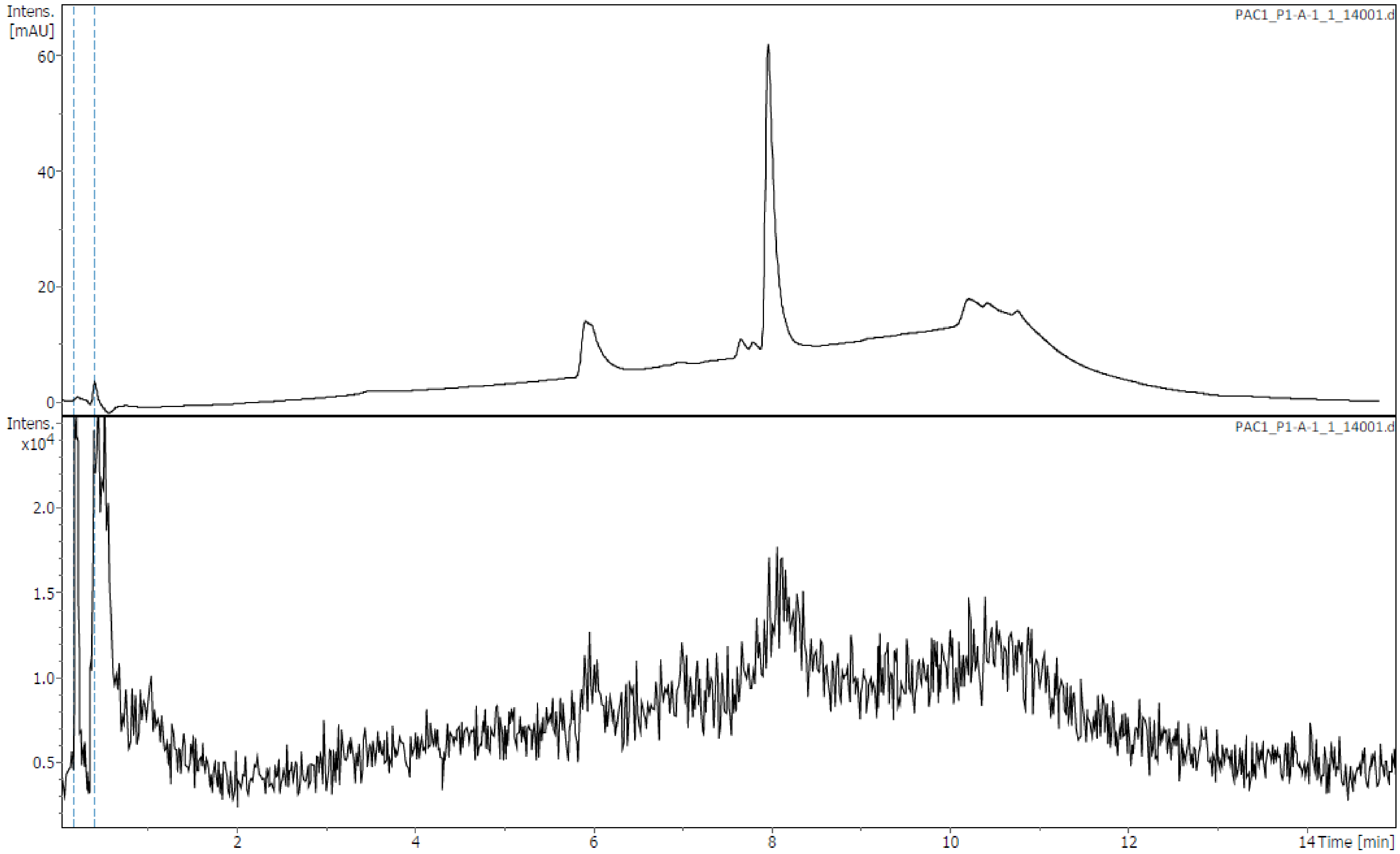

Figure S3 LC-HRMS of negative control reaction between **24** and **1**. All reaction components were included apart from the enzyme. Top diagram shows total ion current. Bottom diagram shows extracted ion current (± 0.5 Da) for the expected product ([M+H]+), which could not be found.

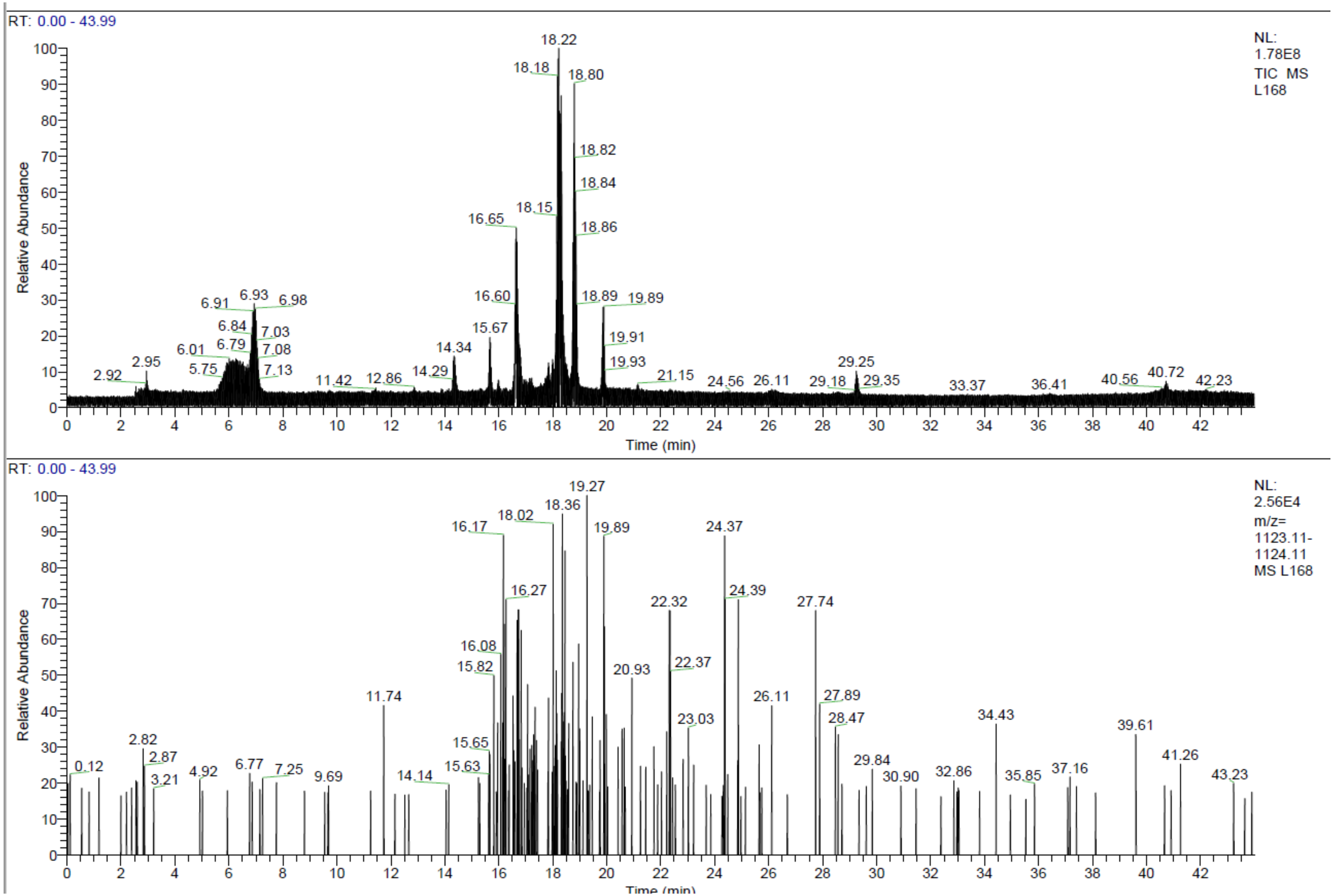

Figure S4 LC-HRMS (EIC) of reaction between **24** and **2**. Top diagram shows total ion current. Bottom diagram shows extracted ion current (± 0.5 Da) for the expected product ($[M+H]^+$), which could not be found.

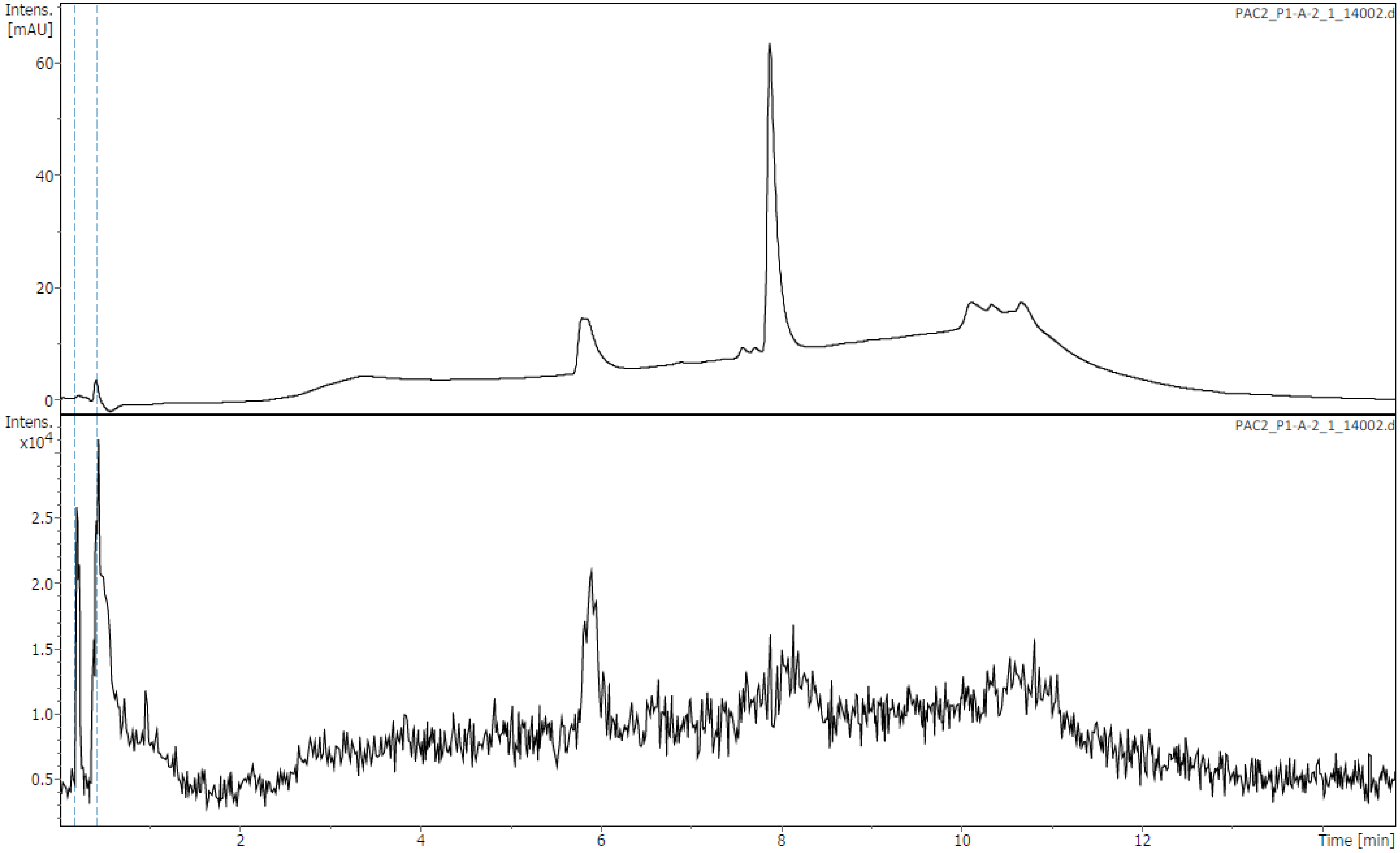

Figure S5 LC-HRMS of negative control reaction between **24** and **2**. All reaction components were included apart from the enzyme. Top diagram shows total ion current. Bottom diagram shows extracted ion current (± 0.5 Da) for the expected product ([M+H]+), which could not be found.

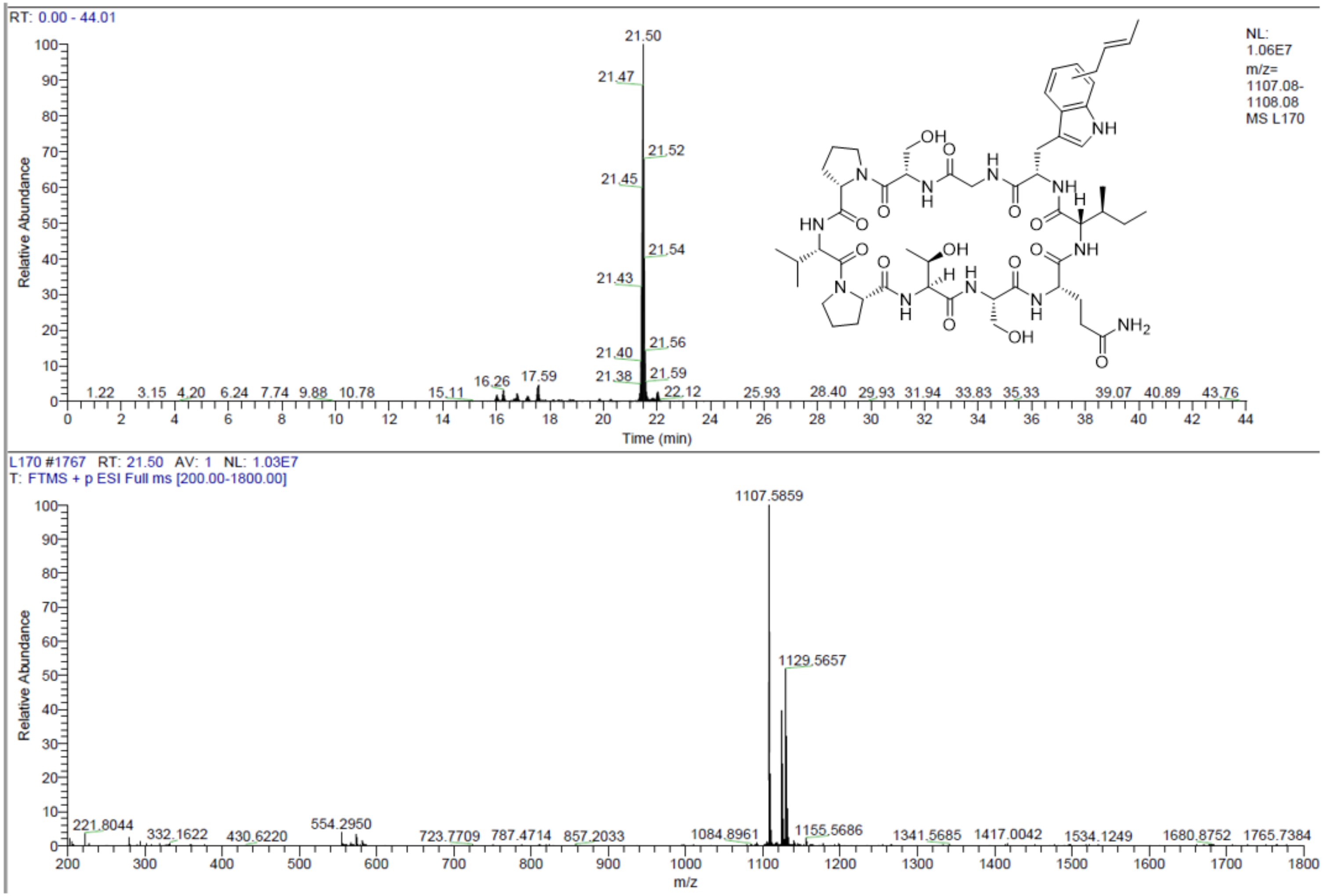

Figure S6 LC-HRMS (EIC) of reaction between **24** and **3**. Top diagram shows extracted ion current (± 0.5 Da) for the expected product ($[M+H]^+$). Bottom diagram shows HRMS spectrum of the expected product (T_R 21.5 min).

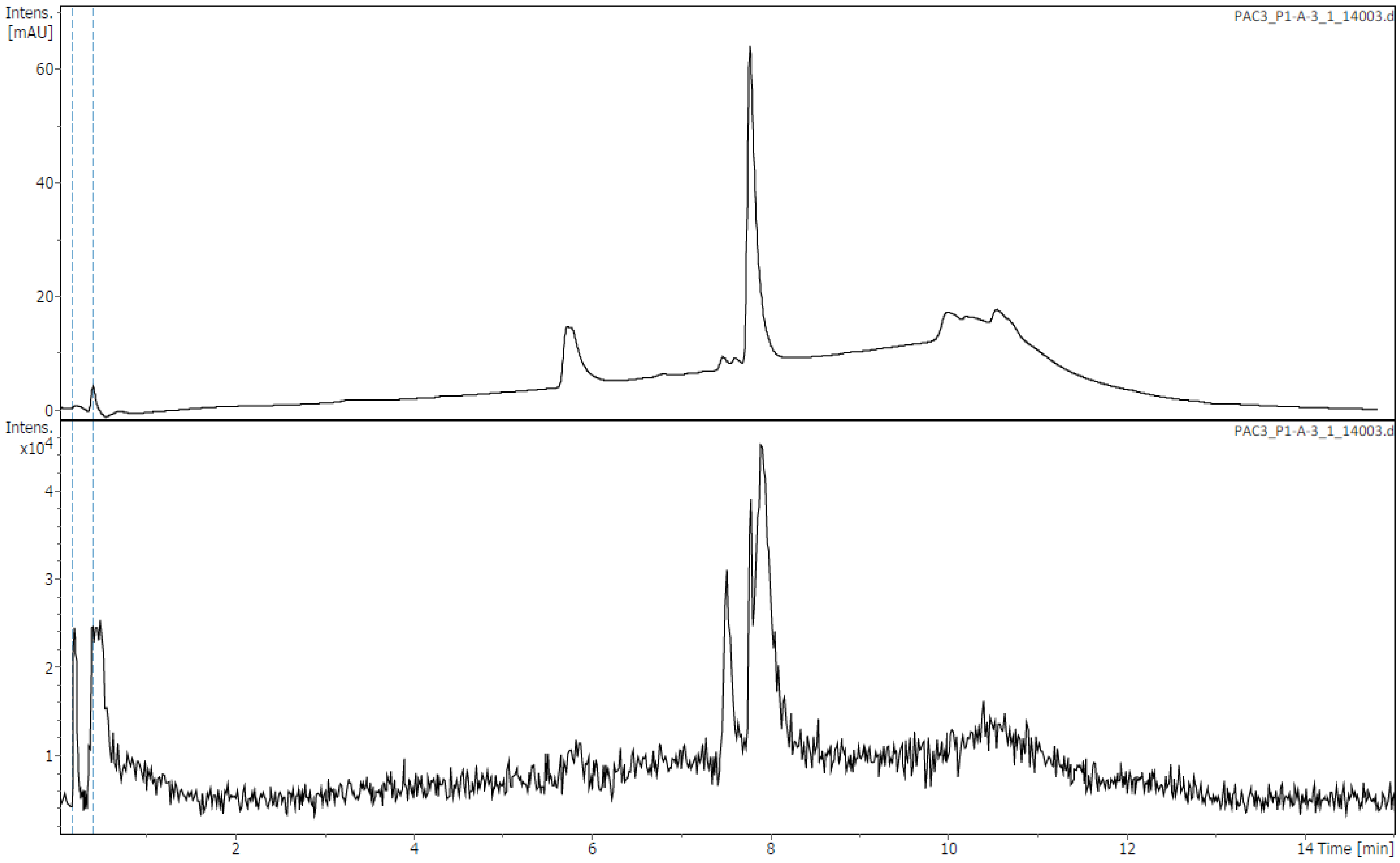

Figure S7 LC-HRMS of negative control reaction between **24** and **3**. All reaction components were included apart from the enzyme. Top diagram shows total ion current. Bottom diagram shows extracted ion current (± 0.5 Da) for the expected product ([M+H]+), which could not be found.

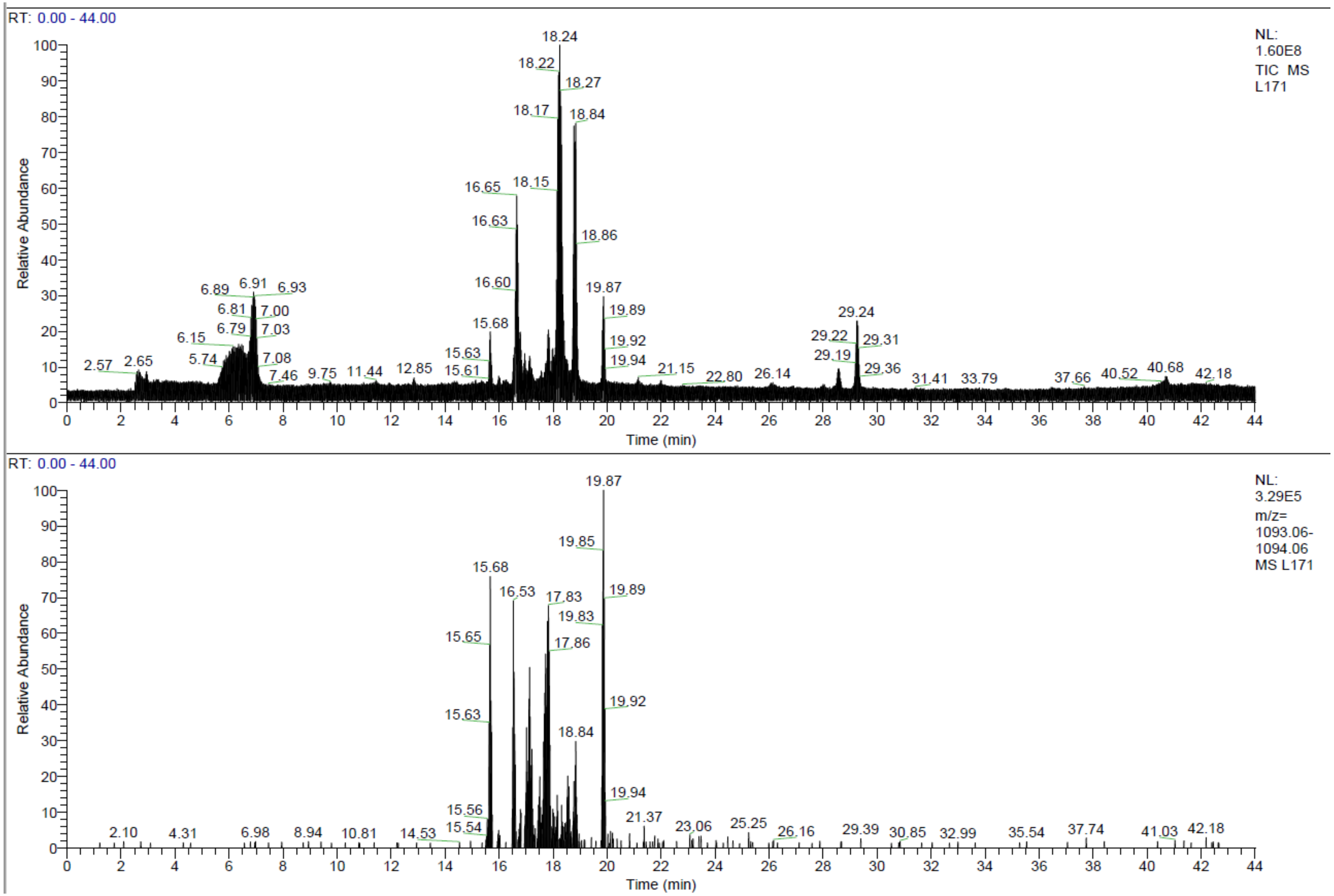

Figure S8 LC-HRMS of reaction between **24** and **4**. Top diagram shows total ion current. Bottom diagram shows extracted ion current (± 0.5 Da) for the expected product ($[M+H]^+$), which could not be found.

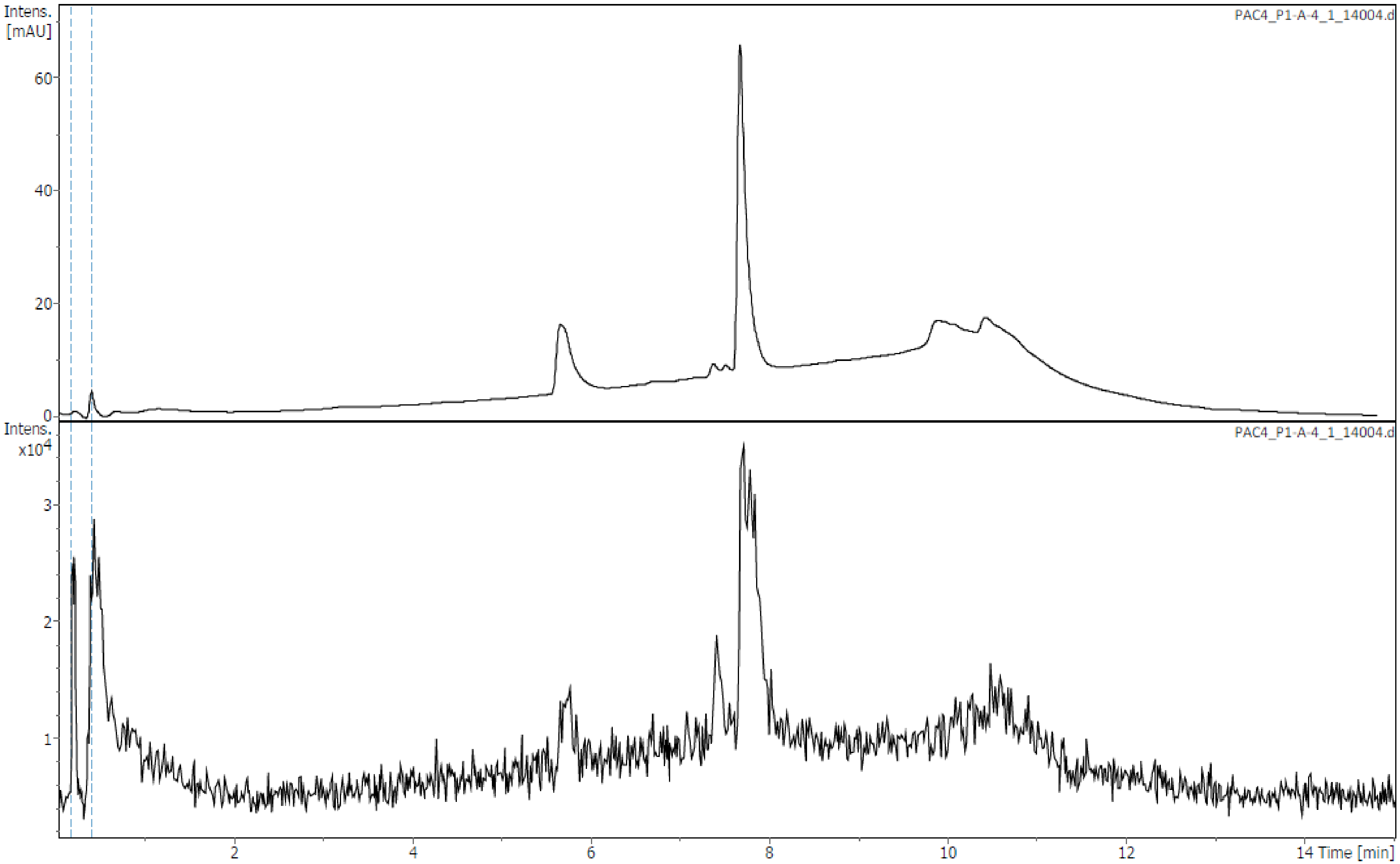

Figure S9 LC-HRMS of negative control reaction between **24** and **4**. All reaction components were included apart from the enzyme. Top diagram shows total ion current. Bottom diagram shows extracted ion current (± 0.5 Da) for the expected product ([M+H]+), which could not be found.

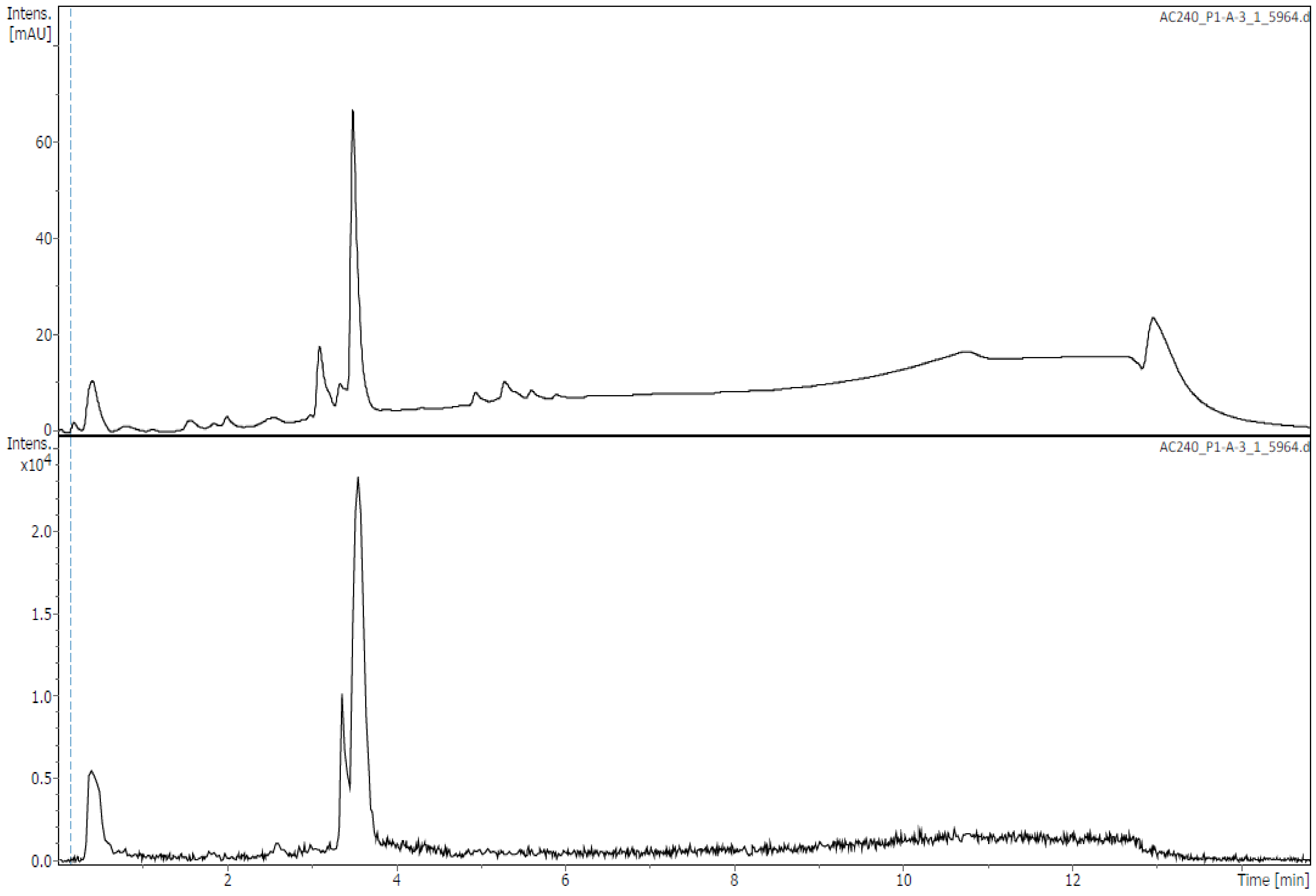

Figure S10 LC-HRMS of reaction between **24** and **5**. Top diagram shows UV absorbance (260 nm). Bottom diagram shows extracted ion current (± 0.5 Da) for the expected product ($[M+H]^+$), which could not be found.

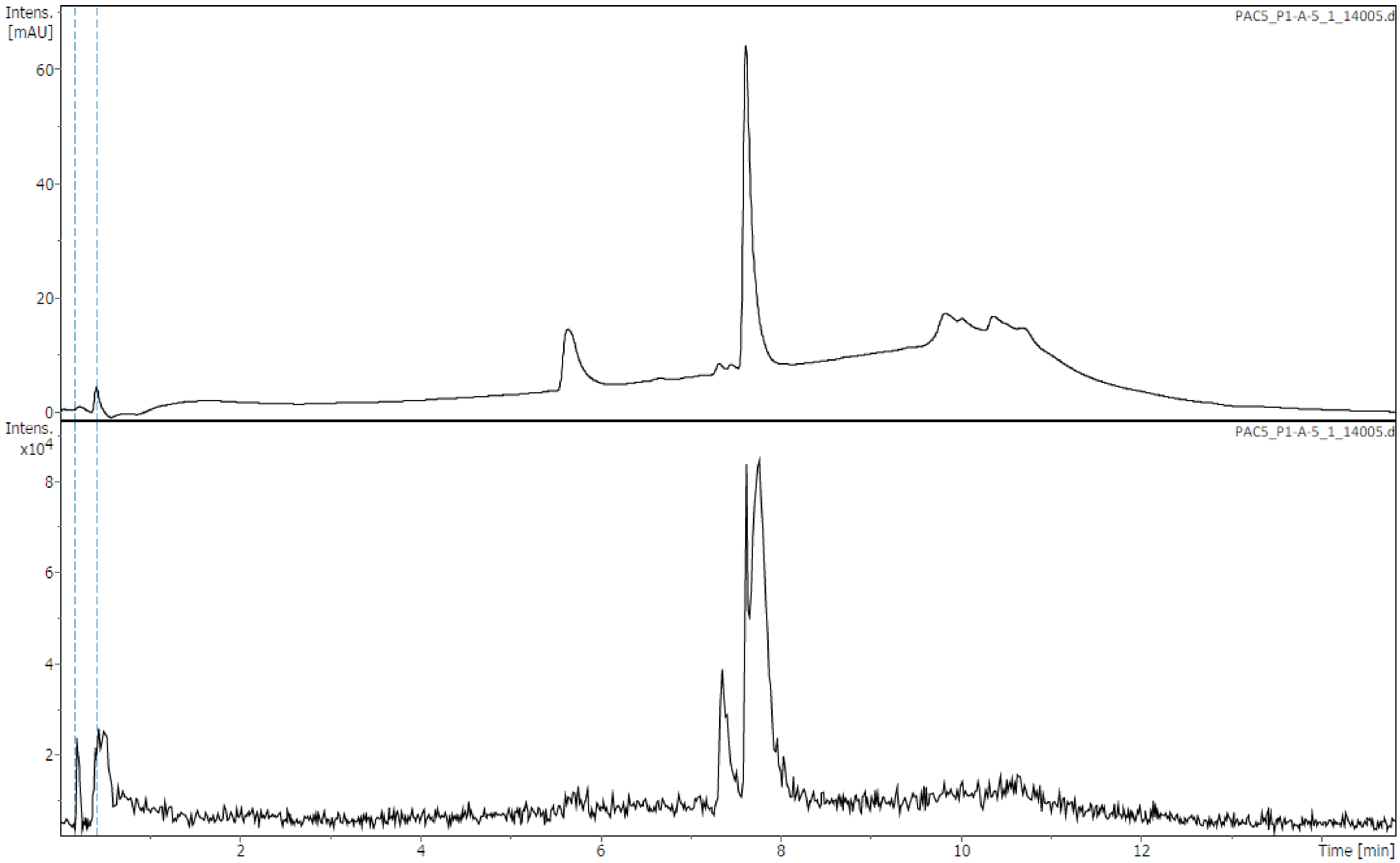

Figure S11 LC-HRMS of negative control reaction between **24** and **5**. All reaction components were included apart from the enzyme. Top diagram shows total ion current. Bottom diagram shows extracted ion current (± 0.5 Da) for the expected product ([M+H]+), which could not be found.

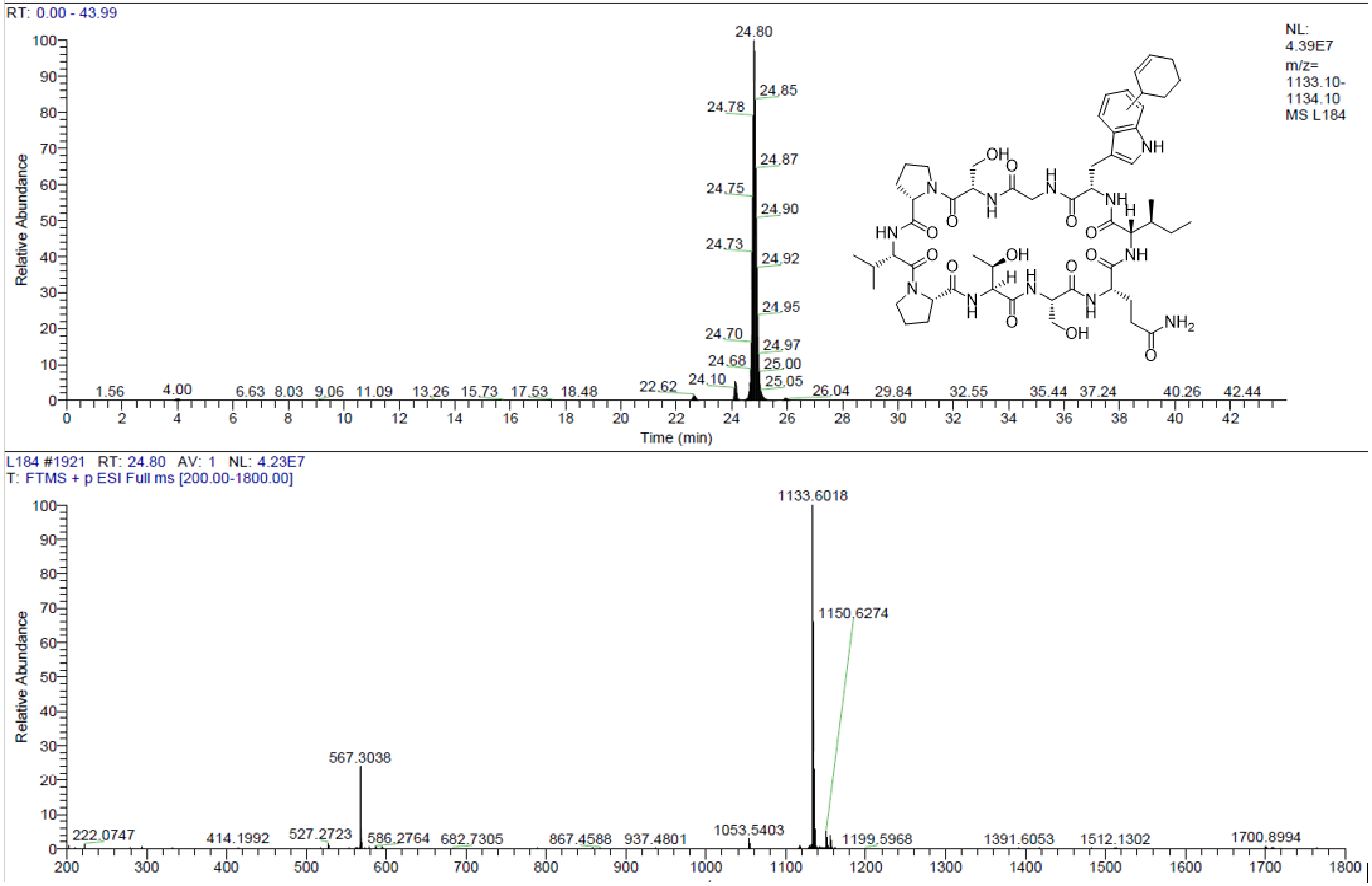

Figure S12 LC-HRMS (EIC) of reaction between **24** and **6**. Top diagram shows extracted ion current (± 0.5 Da) for the expected product ($[M+H]^+$). Bottom diagram shows HRMS spectrum of the expected product (T_R 24.8 min).

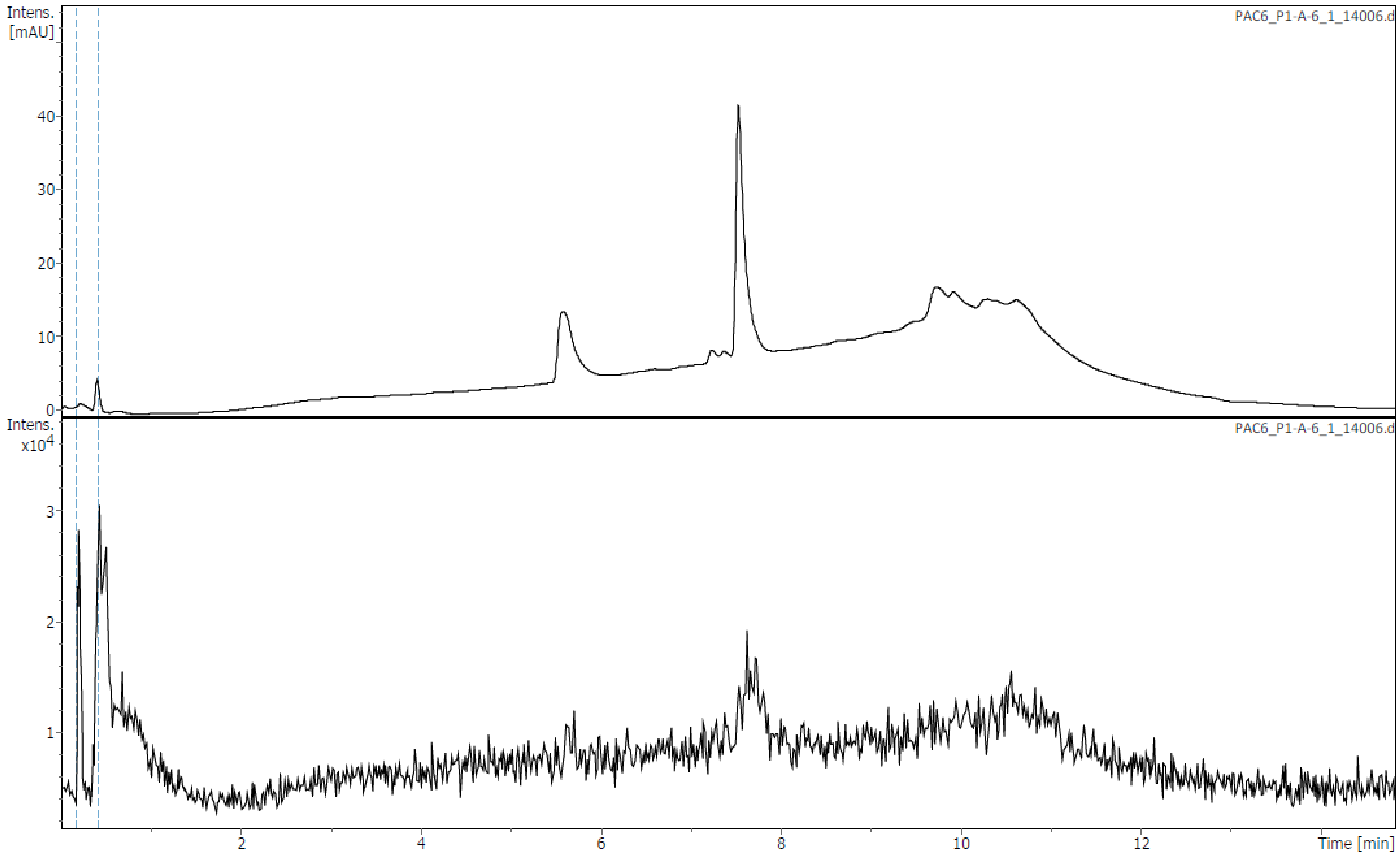

Figure S13 LC-HRMS of negative control reaction between **24** and **6**. All reaction components were included apart from the enzyme. Top diagram shows total ion current. Bottom diagram shows extracted ion current (± 0.5 Da) for the expected product ([M+H]+), which could not be found.

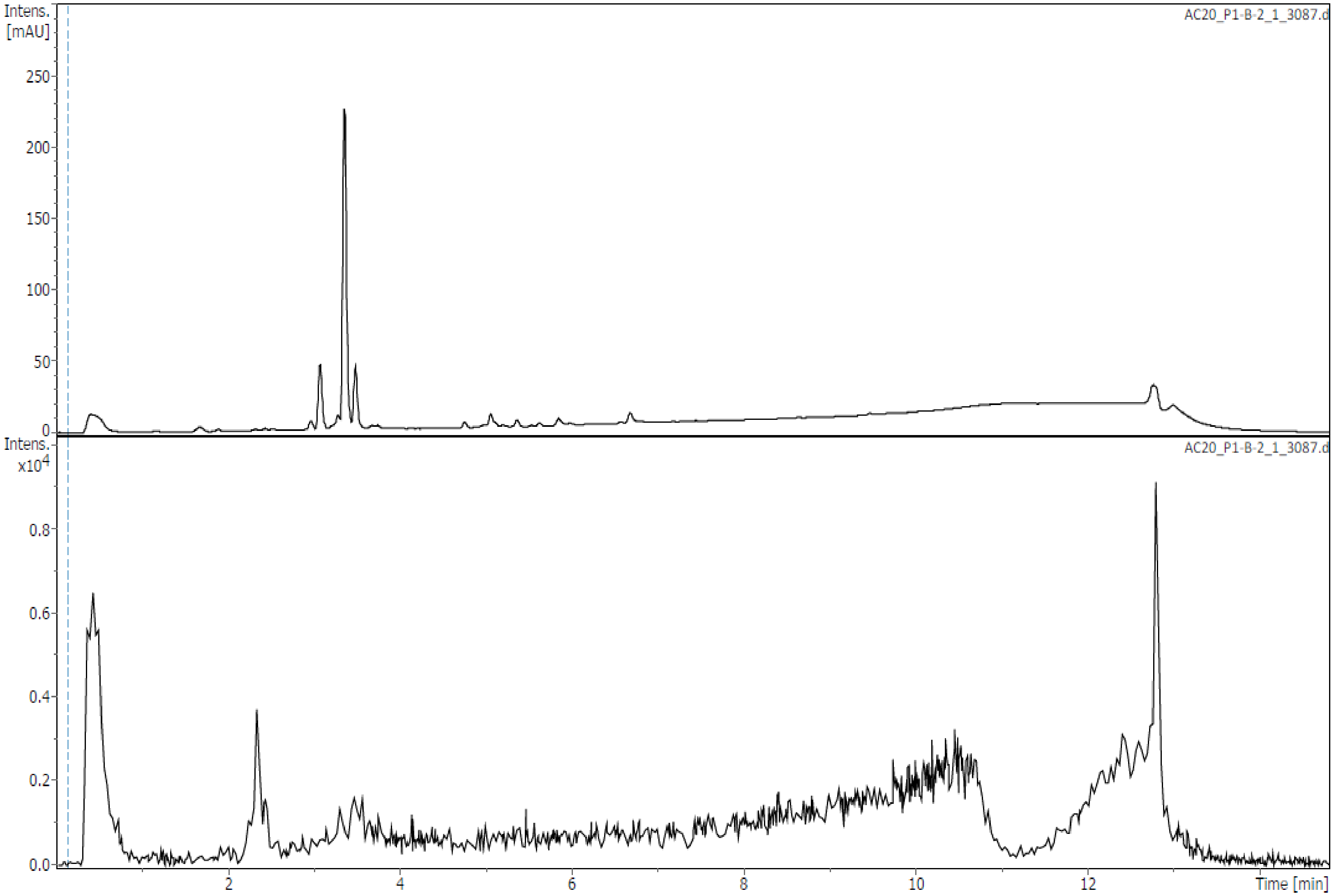

Figure S14 LC-HRMS of reaction between **24** and **7**. Top diagram shows UV absorbance (260 nm). Bottom diagram shows extracted ion current (± 0.5 Da) for the expected product ($[M+H]^+$), which could not be found.

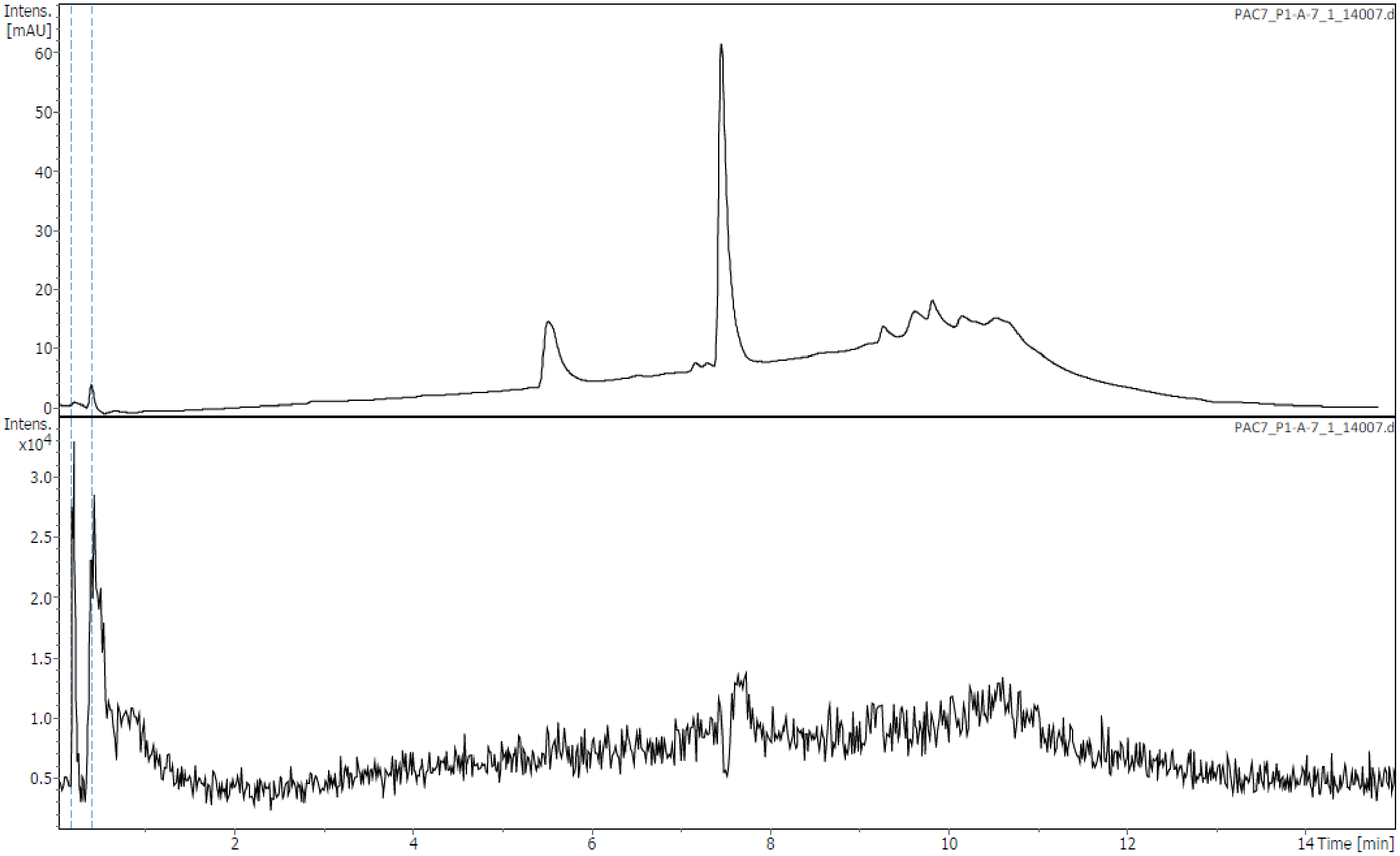

Figure S15 LC-HRMS of negative control reaction between **24** and **7**. All reaction components were included apart from the enzyme. Top diagram shows total ion current. Bottom diagram shows extracted ion current (± 0.5 Da) for the expected product ([M+H]+), which could not be found.

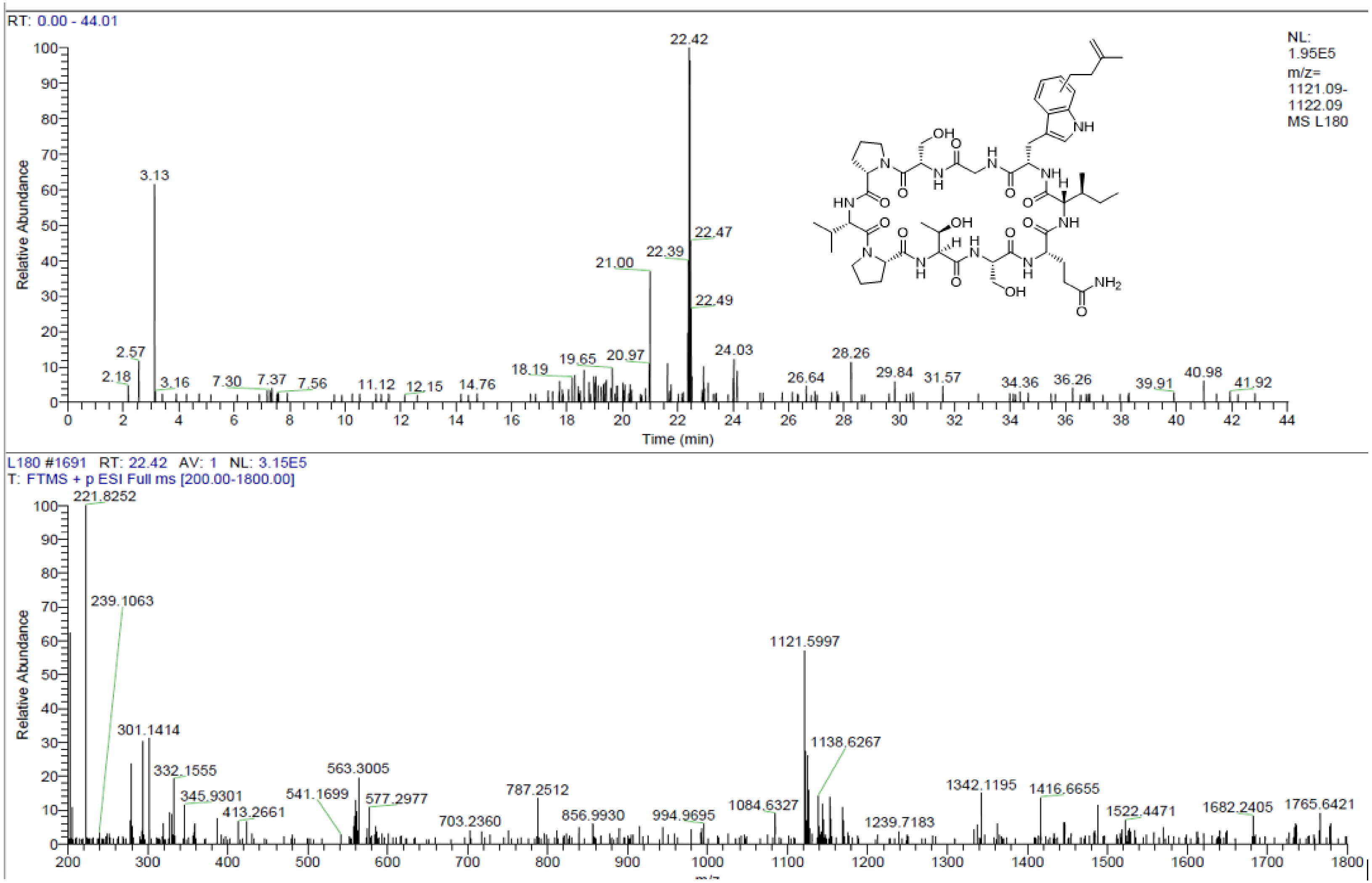

Figure S16 LC-HRMS (EIC) of reaction between **24** and **8**. Top diagram shows extracted ion current (± 0.5 Da) for the expected product ([M+H]$^+$). Bottom diagram shows HRMS spectrum of the expected product (T_R 22.4 min).

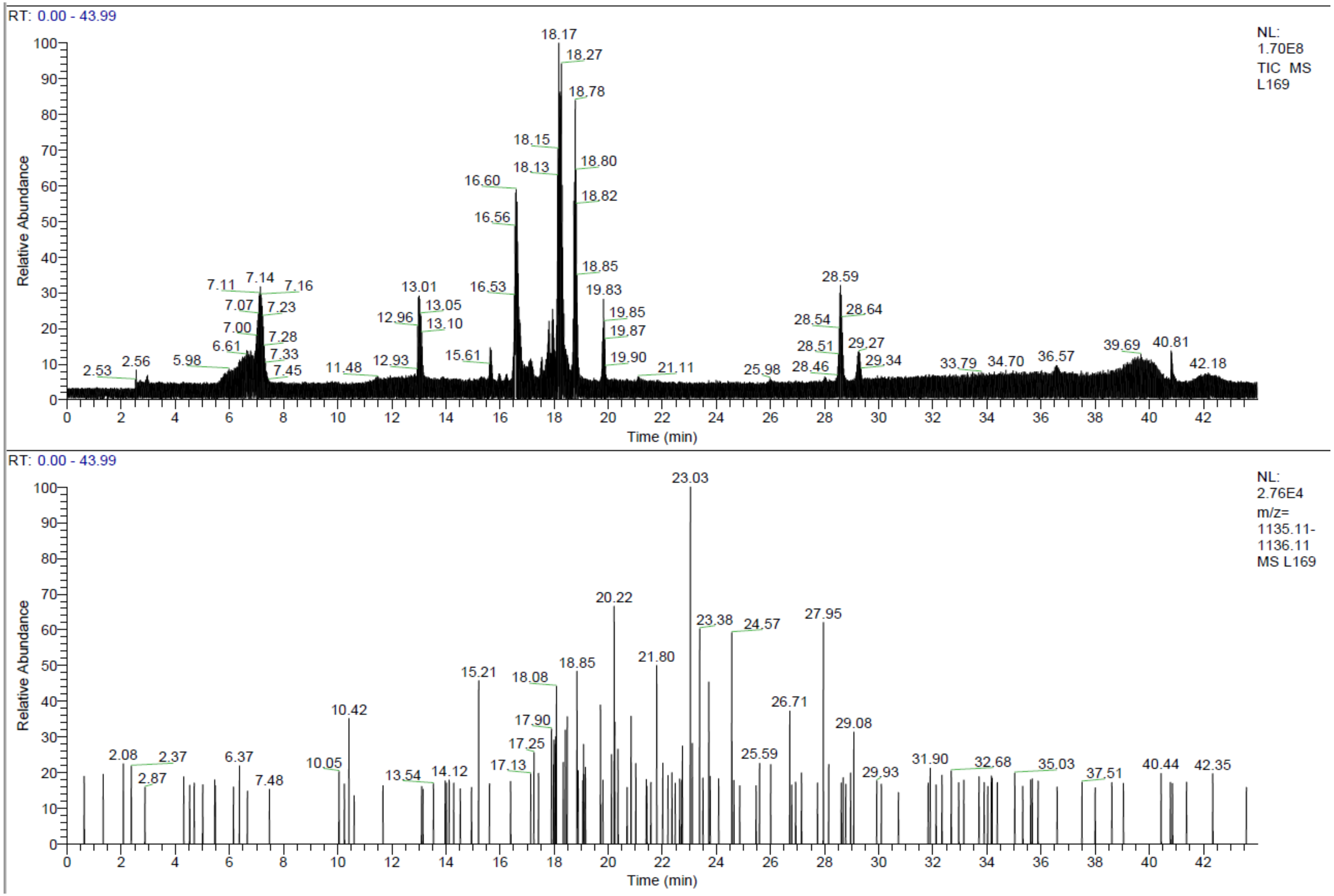

Figure S17 LC-HRMS of reaction between **24** and **9**. Top diagram shows total ion current. Bottom diagram shows extracted ion current (± 0.5 Da) for the expected product ($[M+H]^+$), which could not be found.

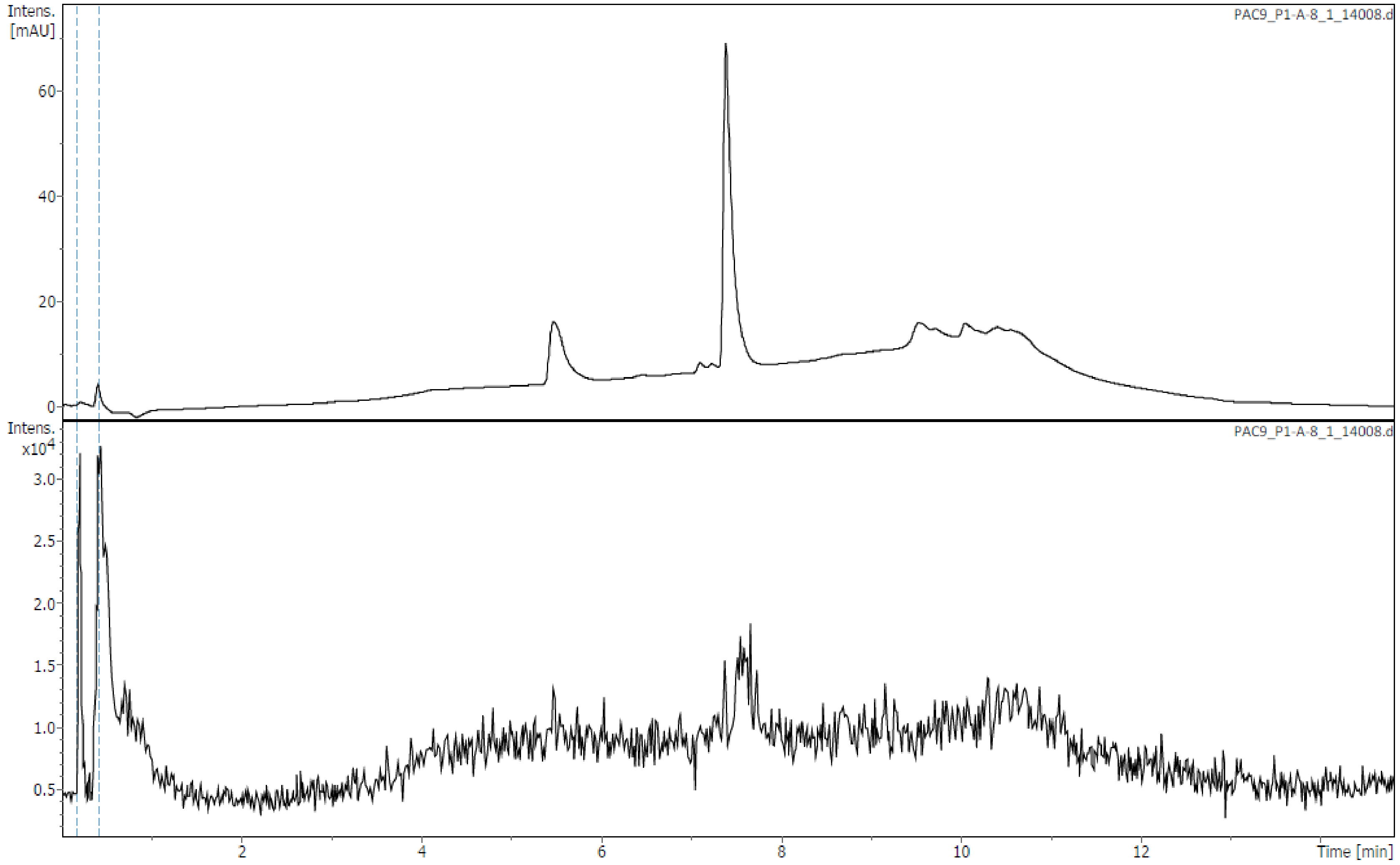

Figure S18 LC-HRMS of negative control reaction between **24** and **9**. All reaction components were included apart from the enzyme. Top diagram shows total ion current. Bottom diagram shows extracted ion current (± 0.5 Da) for the expected product ([M+H]+), which could not be found.

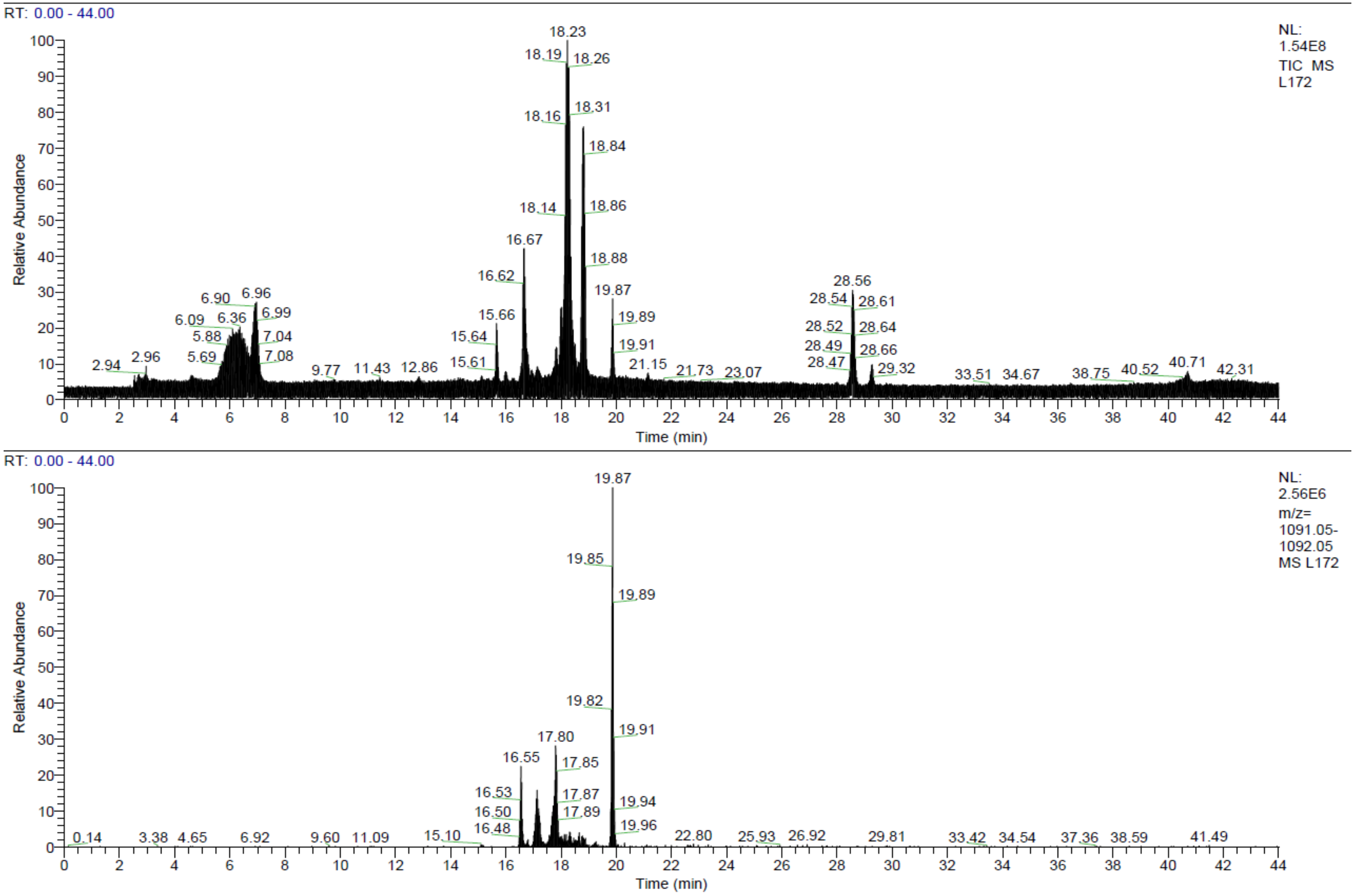

Figure S19 LC-HRMS of reaction between **24** and **10**. Top diagram shows total ion current. Bottom diagram shows extracted ion current (± 0.5 Da) for the expected product ($[M+H]^+$), which could not be found.

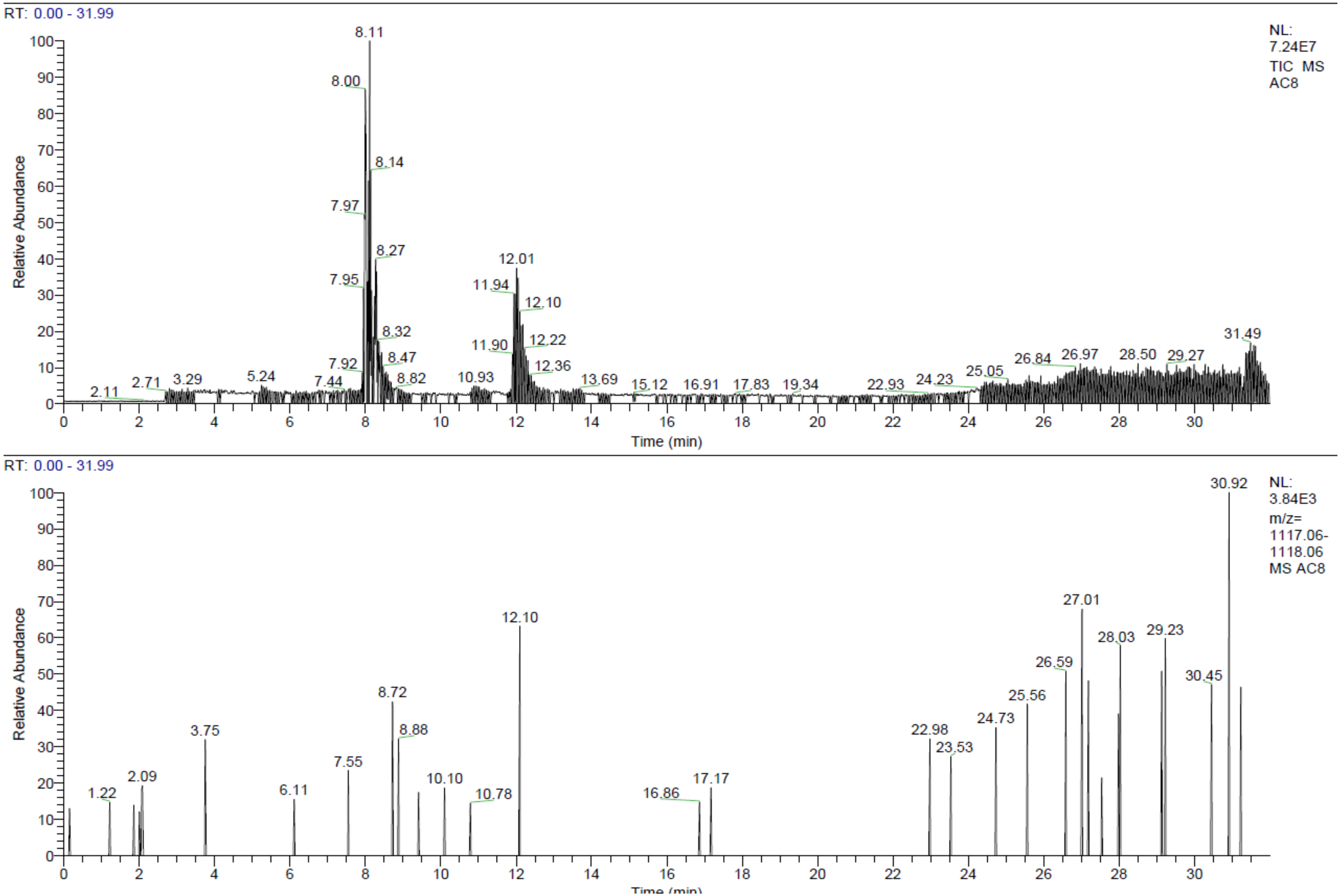

Figure S20 LC-HRMS of reaction between **24** and **11**. Top diagram shows total ion current. Bottom diagram shows extracted ion current (± 0.5 Da) for the expected product ([M+H]$^+$), which could not be found.

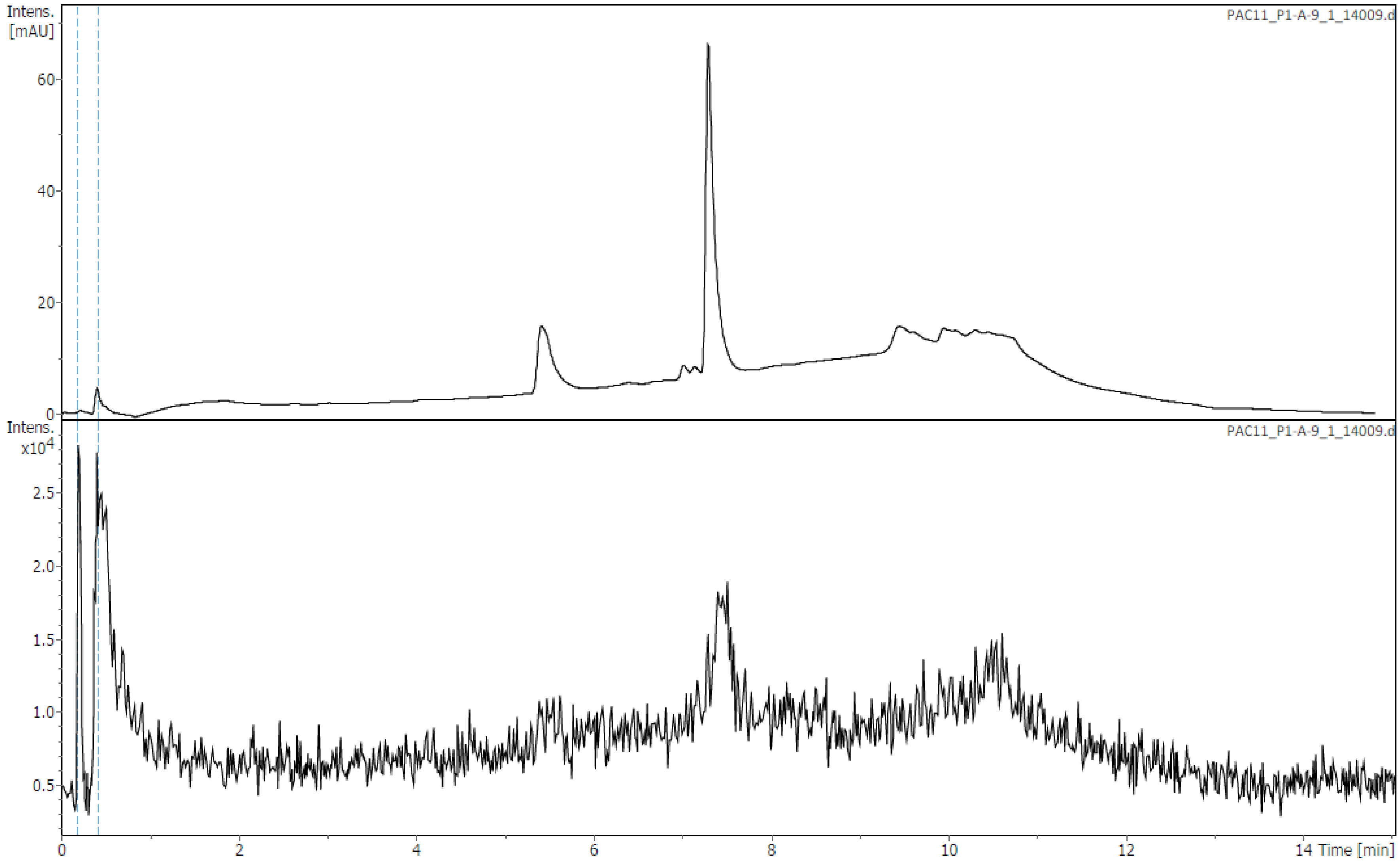

Figure S21 LC-HRMS of negative control reaction between **24** and **11**. All reaction components were included apart from the enzyme. Top diagram shows total ion current. Bottom diagram shows extracted ion current (± 0.5 Da) for the expected product ([M+H]+), which could not be found.

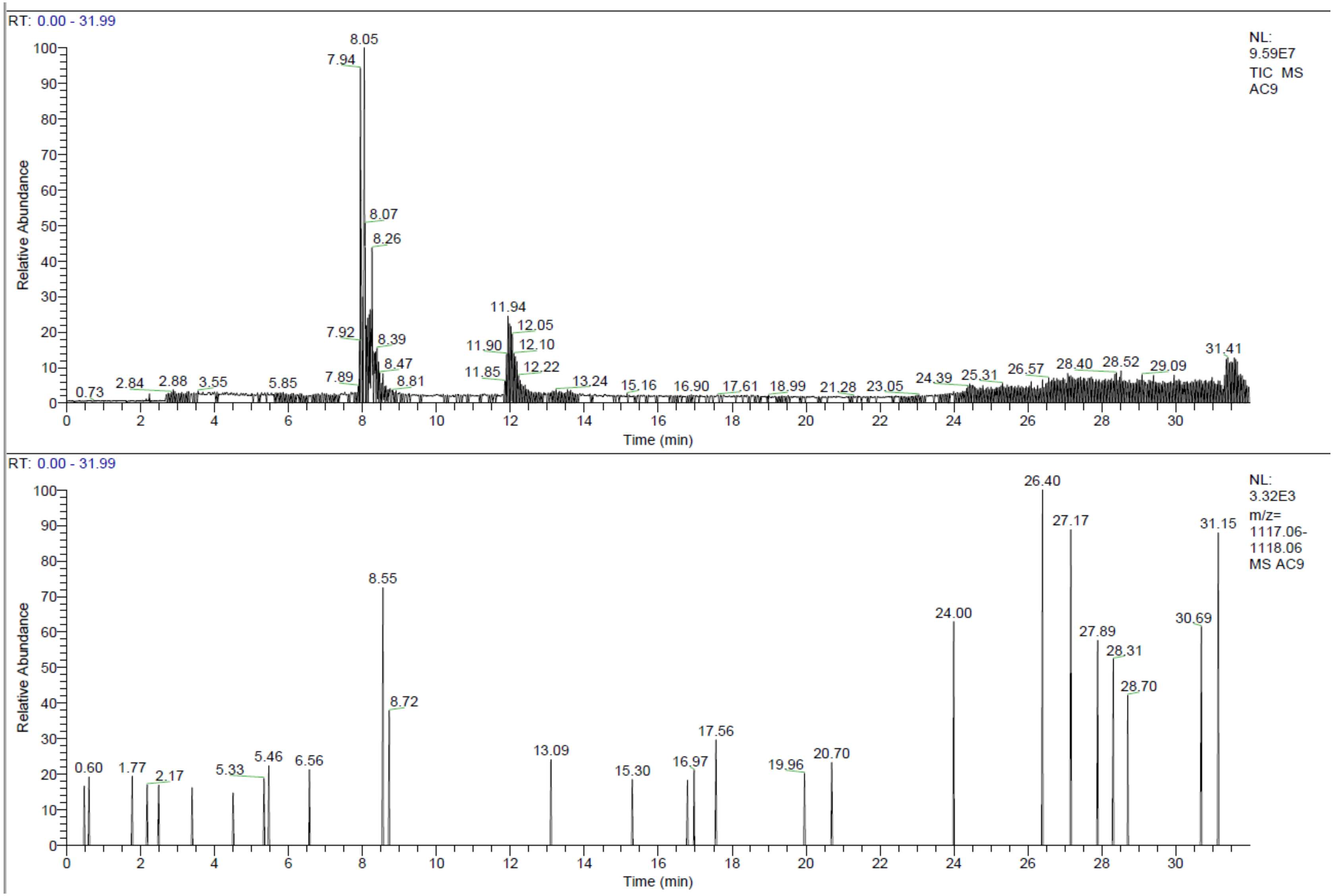

Figure S22 LC-HRMS of reaction between **24** and **12**. Top diagram shows total ion current. Bottom diagram shows extracted ion current (± 0.5 Da) for the expected product ([M+H]$^+$), which could not be found.

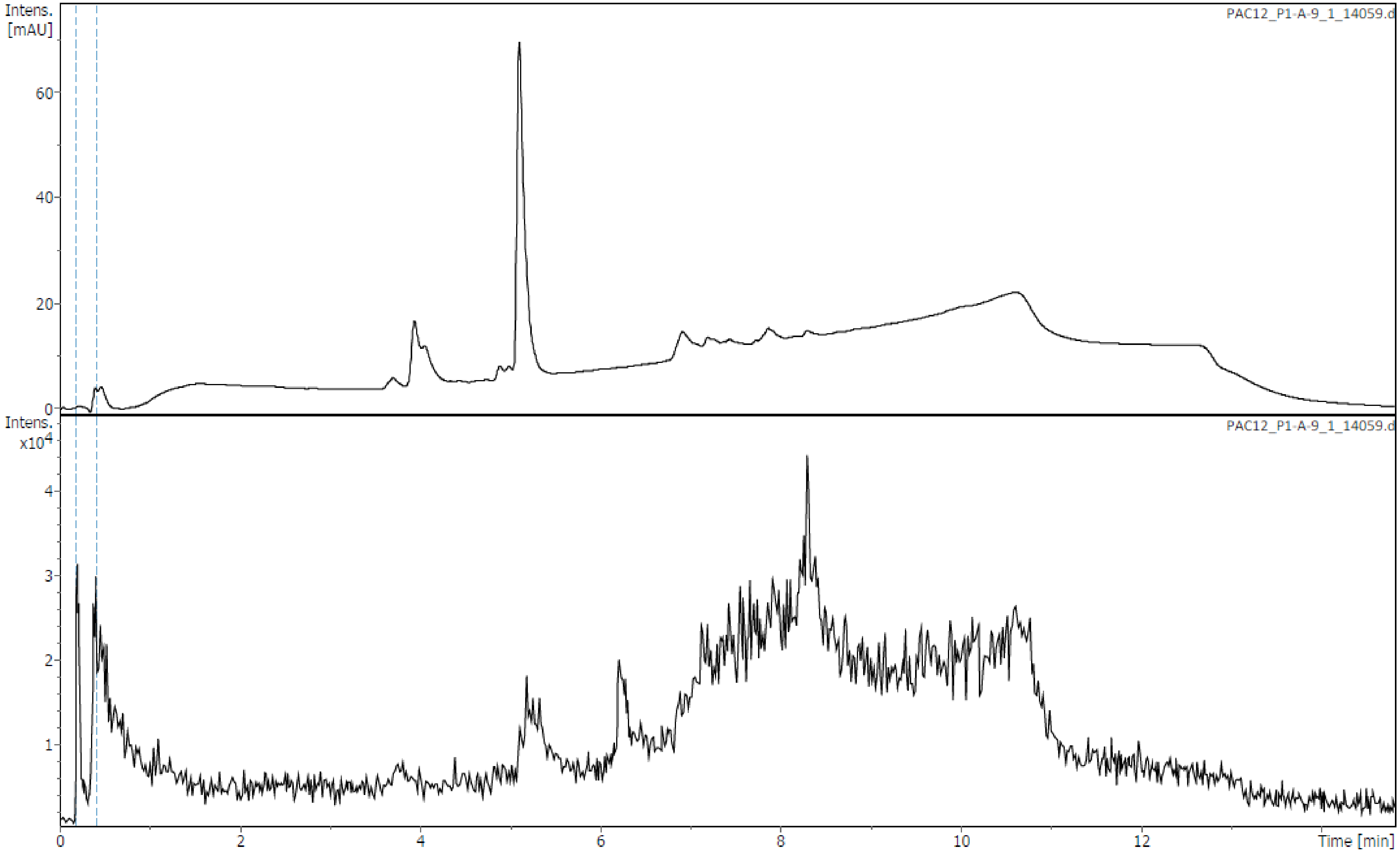

Figure S23 LC-HRMS of negative control reaction between **24** and **12**. All reaction components were included apart from the enzyme. Top diagram shows total ion current. Bottom diagram shows extracted ion current (± 0.5 Da) for the expected product ([M+H]+), which could not be found.

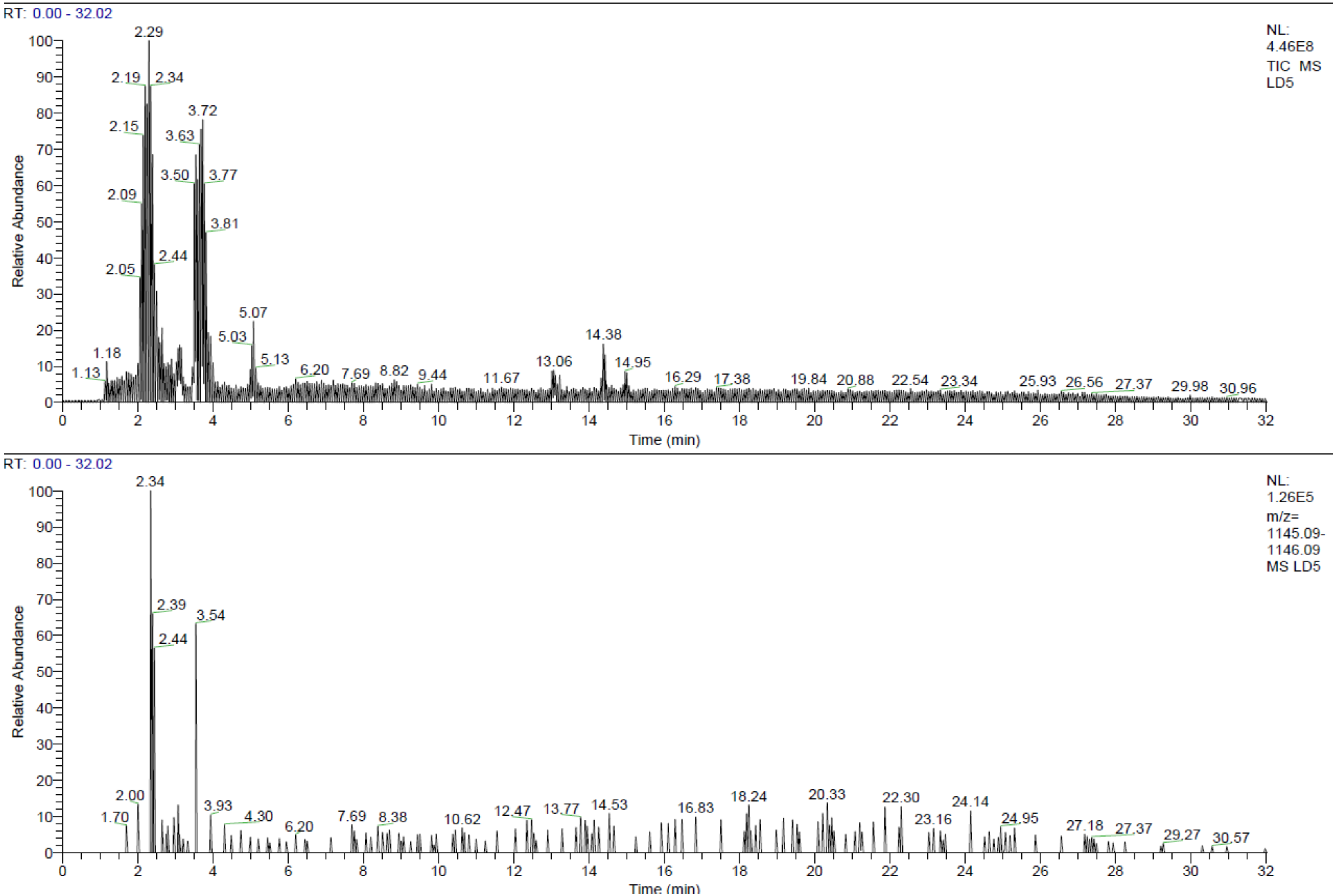

Figure S24 LC-HRMS of reaction between **24** and **13**. Top diagram shows total ion current. Bottom diagram shows extracted ion current (± 0.5 Da) for the expected product ($[M+H]^+$), which could not be found.

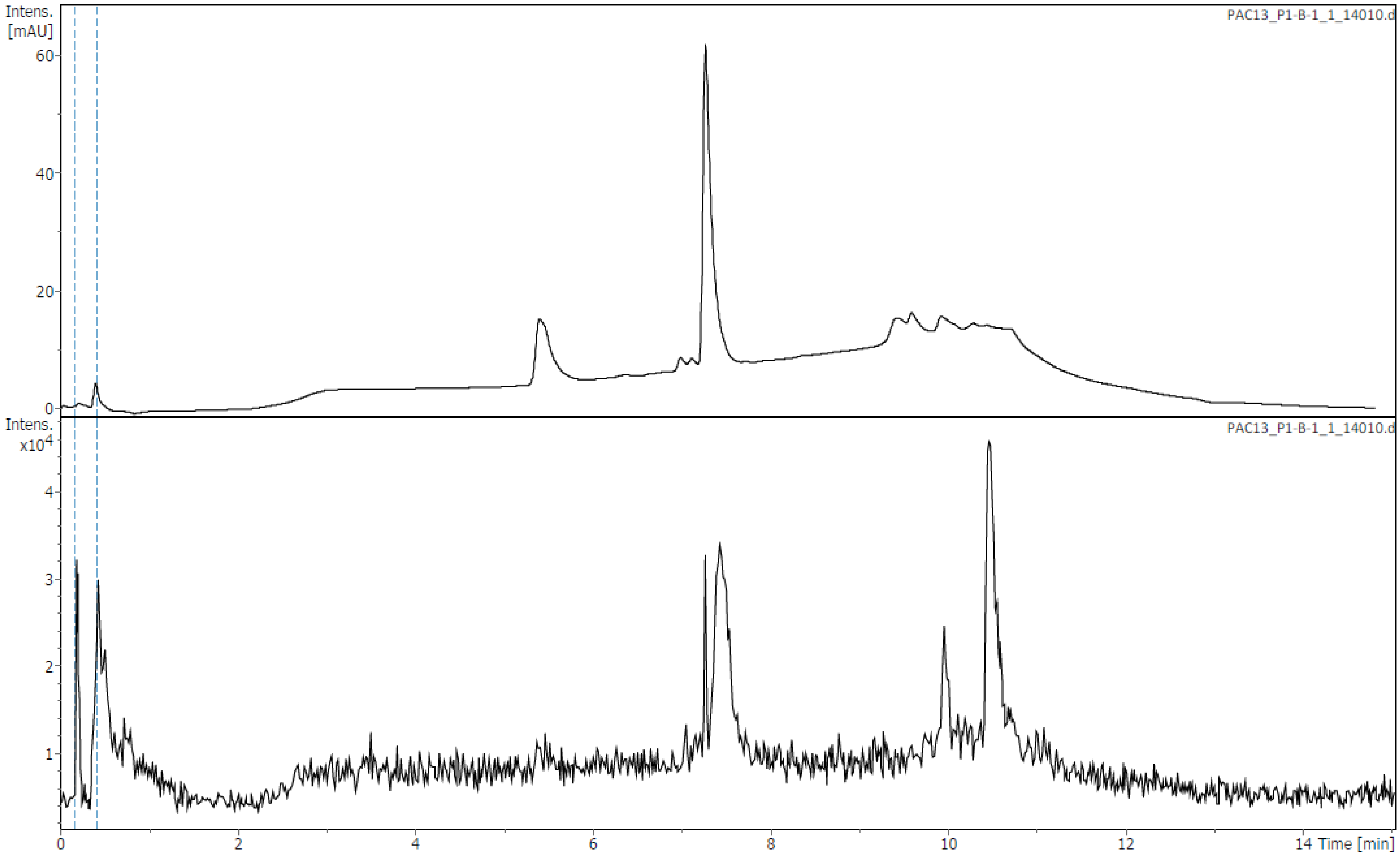

Figure S25 LC-HRMS of negative control reaction between **24** and **13**. All reaction components were included apart from the enzyme. Top diagram shows total ion current. Bottom diagram shows extracted ion current (± 0.5 Da) for the expected product ([M+H]+), which could not be found.

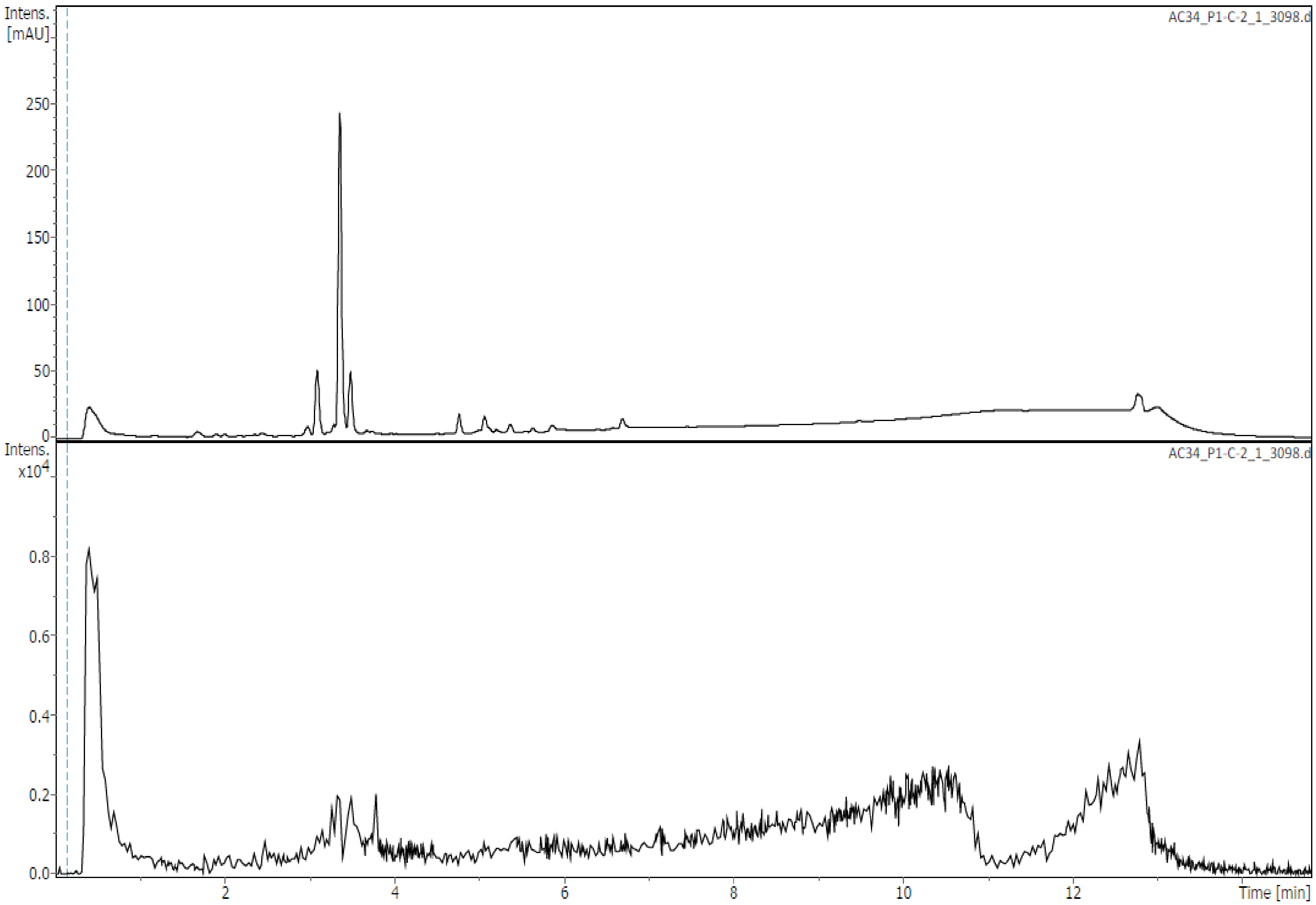

Figure S26 LC-HRMS of reaction between **24** and **14**. Top diagram shows UV absorbance (260 nm). Bottom diagram shows extracted ion current (± 0.5 Da) for the expected ($[M+H]^+$), which could not be found.

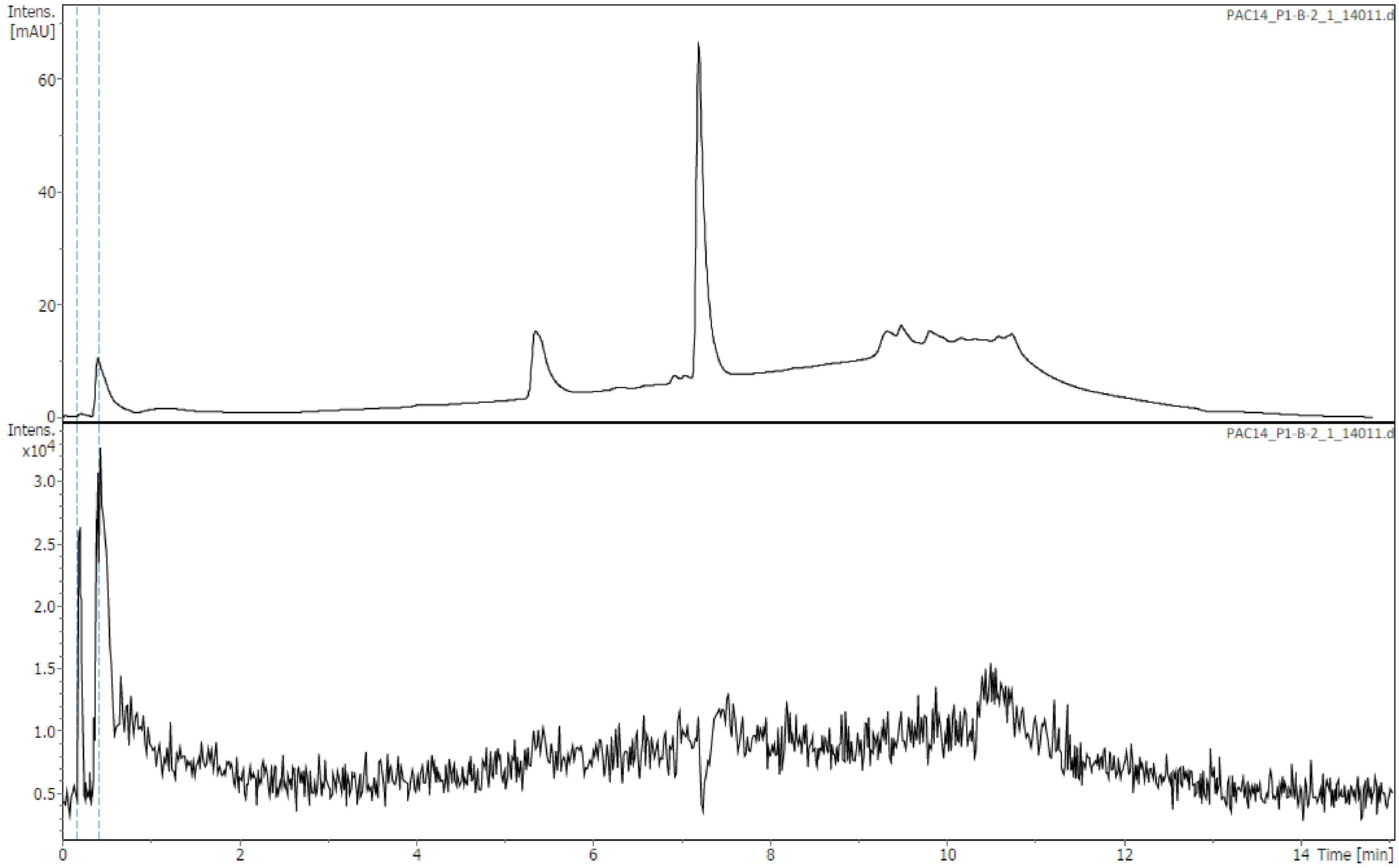

Figure S27 LC-HRMS of negative control reaction between **24** and **14**. All reaction components were included apart from the enzyme. Top diagram shows total ion current. Bottom diagram shows extracted ion current (± 0.5 Da) for the expected product ([M+H]+), which could not be found.

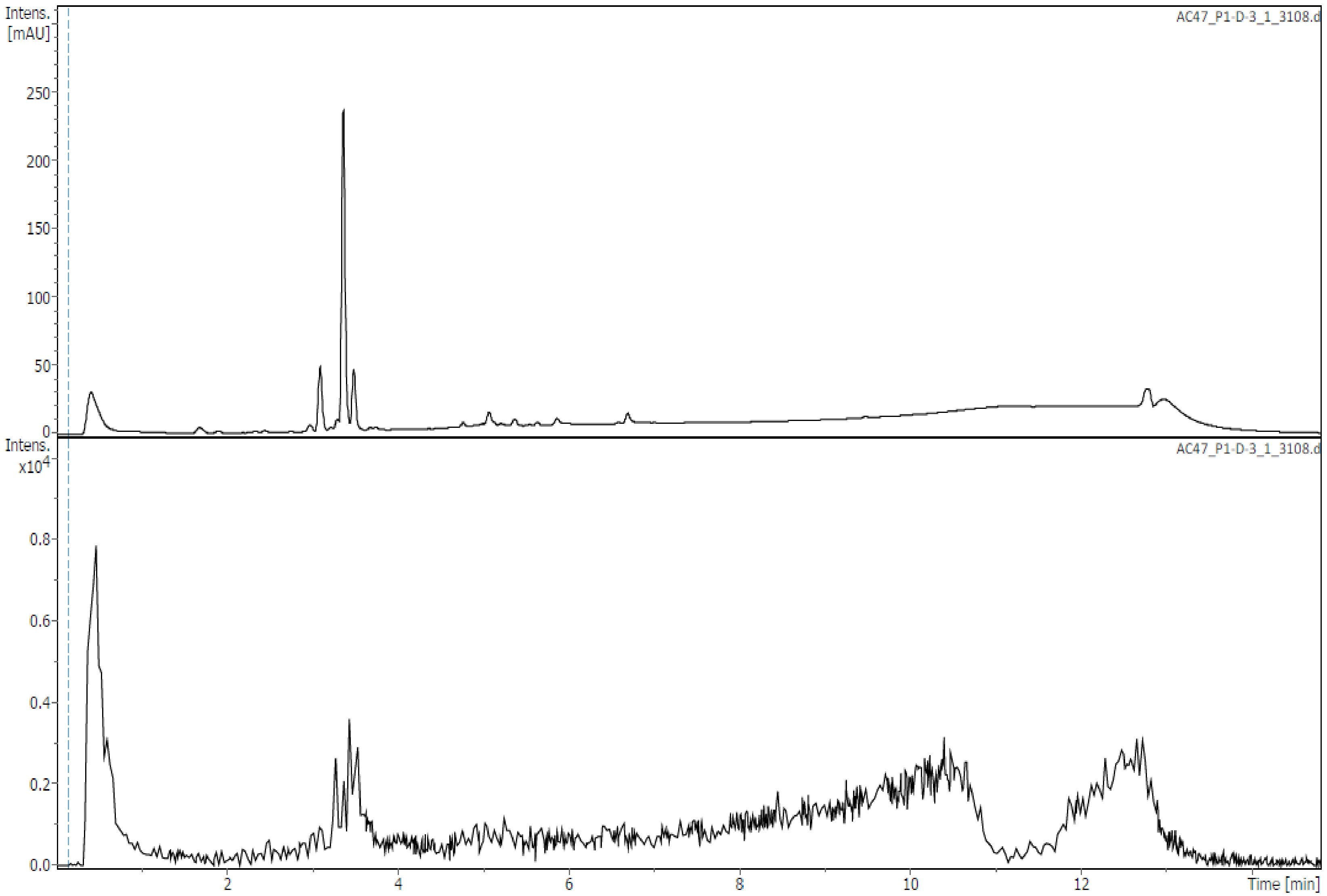

Figure S28 LC-HRMS of reaction between **24** and **15**. Top diagram shows UV absorbance (260 nm). Bottom diagram shows extracted ion current (± 0.5 Da) for the expected ($[M+H]^+$), which could not be found.

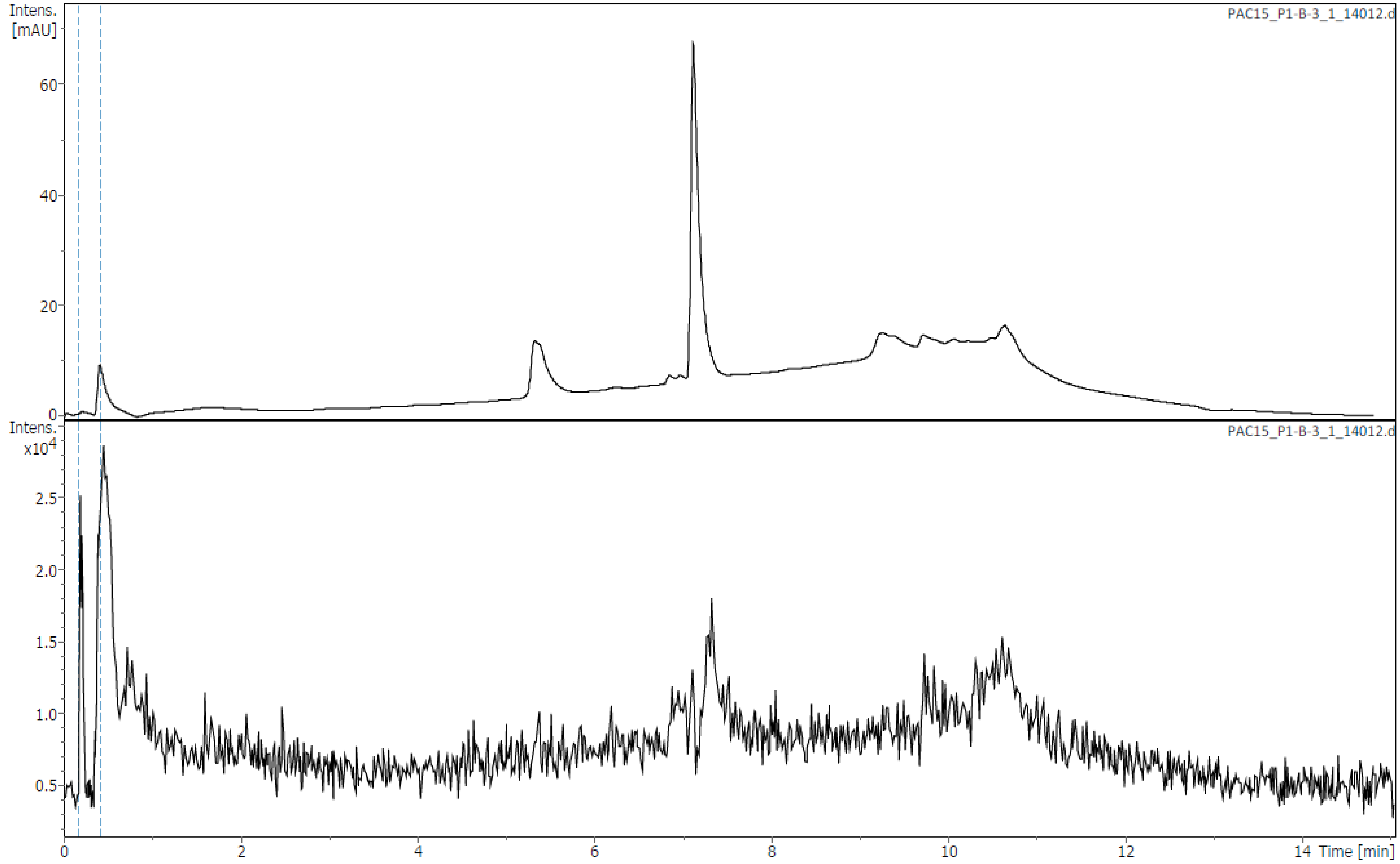

Figure S29 LC-HRMS of negative control reaction between **24** and **15**. All reaction components were included apart from the enzyme. Top diagram shows total ion current. Bottom diagram shows extracted ion current (± 0.5 Da) for the expected product ([M+H]+), which could not be found.

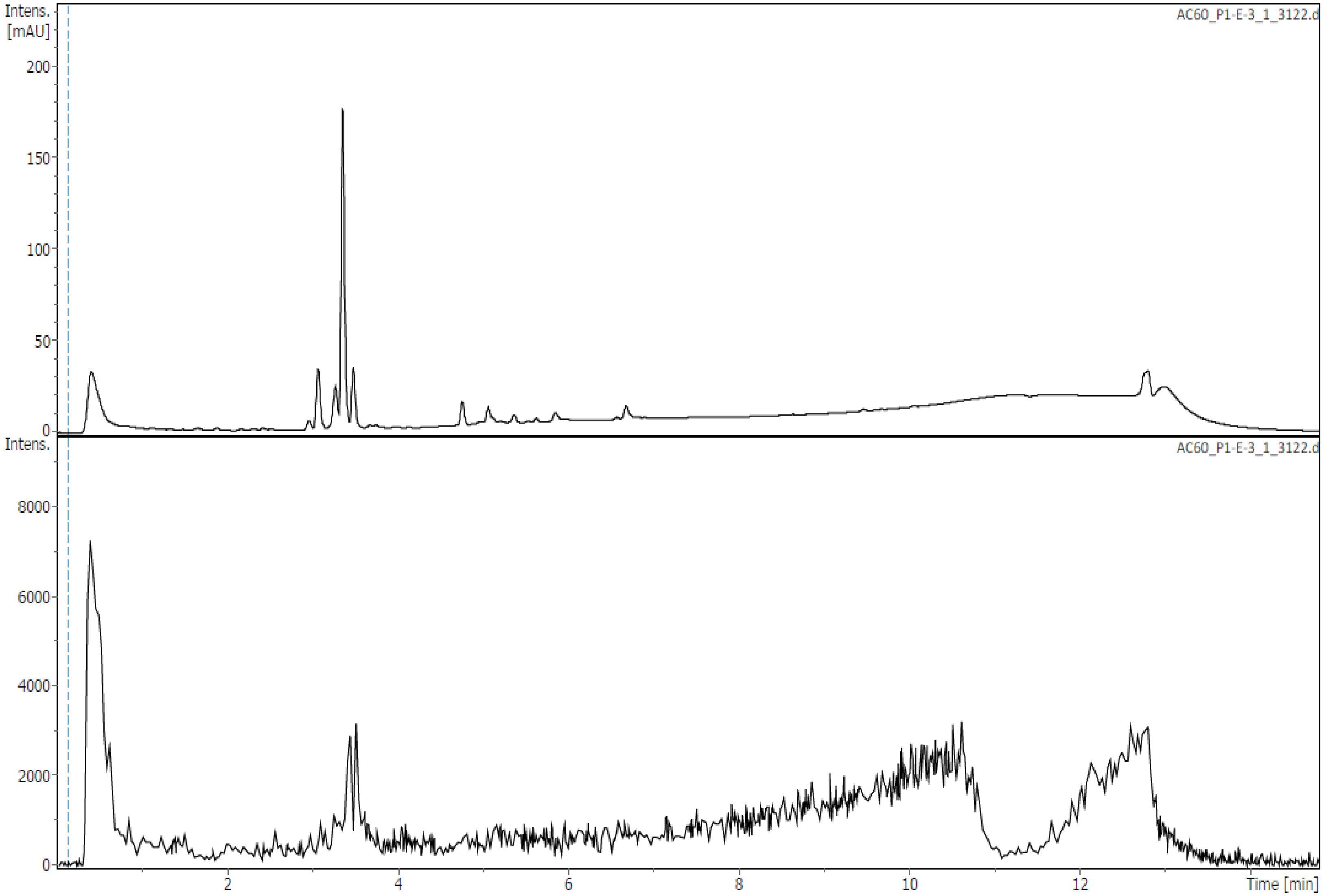

Figure S30 LC-HRMS of reaction between **24** and **16**. Top diagram shows UV absorbance (260 nm). Bottom diagram shows extracted ion current (± 0.5 Da) for the expected ([M+H]$^+$), which could not be found.

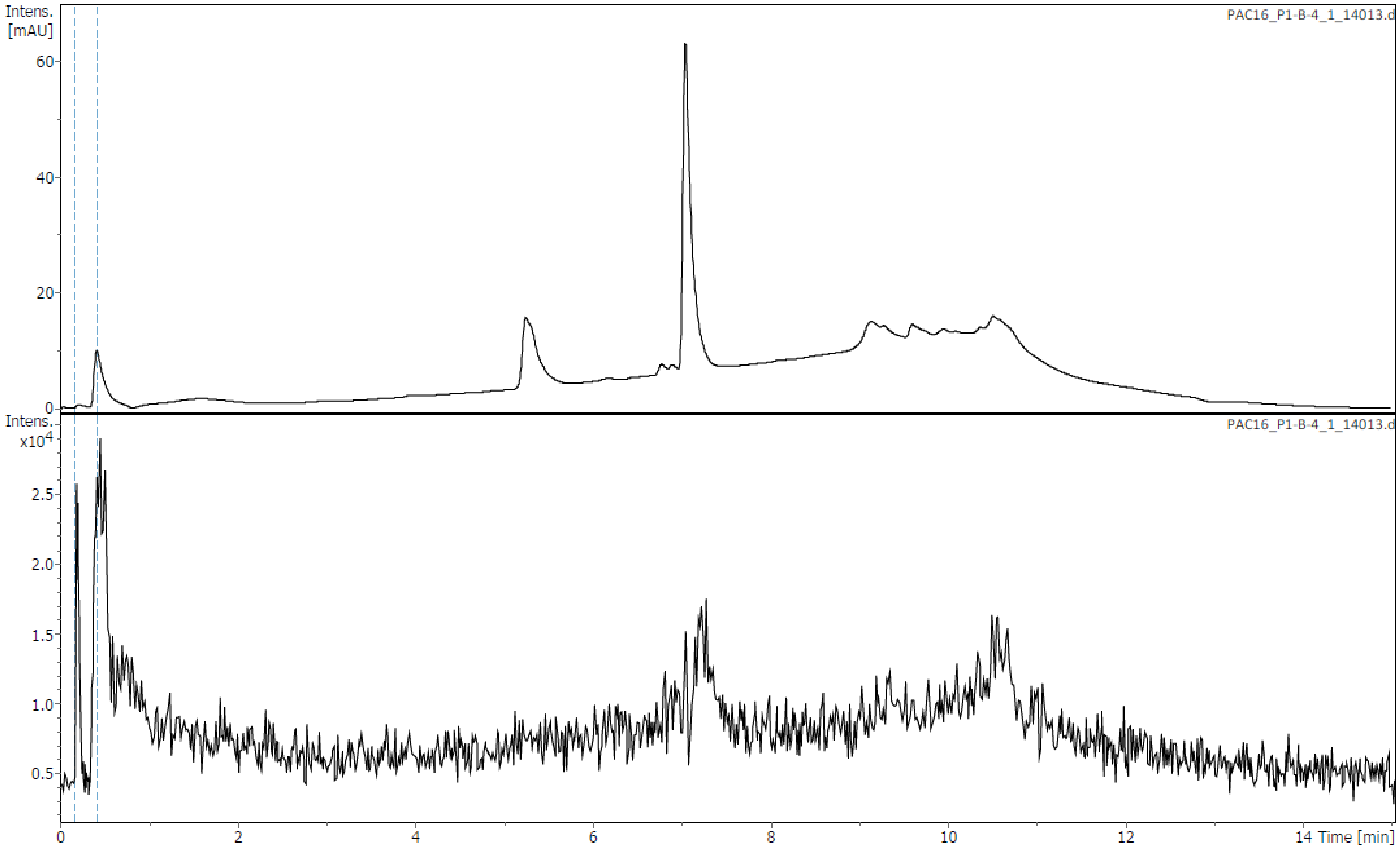

Figure S31 LC-HRMS of negative control reaction between **24** and **16**. All reaction components were included apart from the enzyme. Top diagram shows total ion current. Bottom diagram shows extracted ion current (± 0.5 Da) for the expected product ([M+H]+), which could not be found.

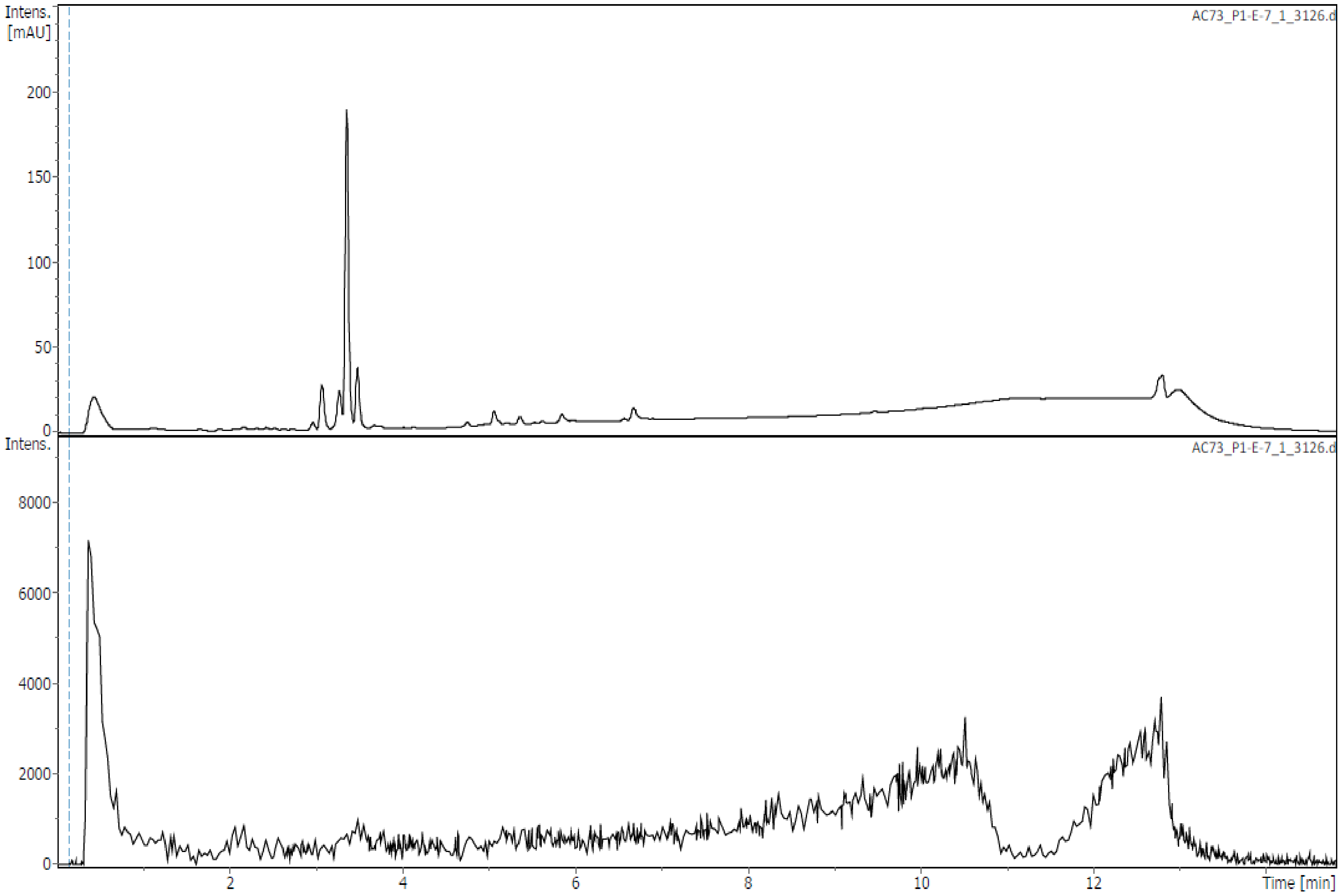

Figure S32 LC-HRMS of reaction between **24** and **17**. Top diagram shows UV absorbance (260 nm). Bottom diagram shows extracted ion current (± 0.5 Da) for the expected ([M+H]$^+$), which could not be found.

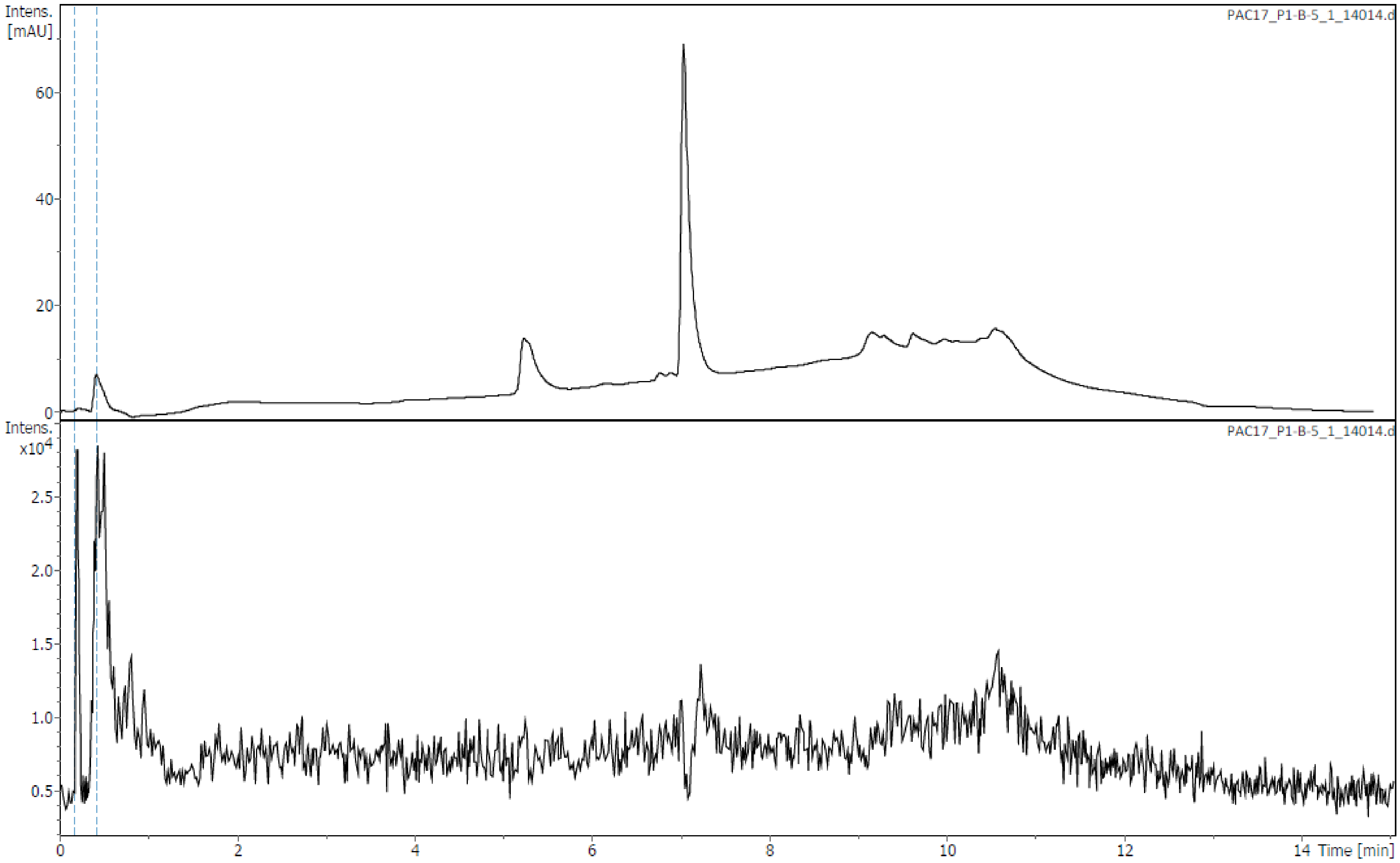

Figure S33 LC-HRMS of negative control reaction between **24** and **17**. All reaction components were included apart from the enzyme. Top diagram shows total ion current. Bottom diagram shows extracted ion current (± 0.5 Da) for the expected product ([M+H]+), which could not be found.

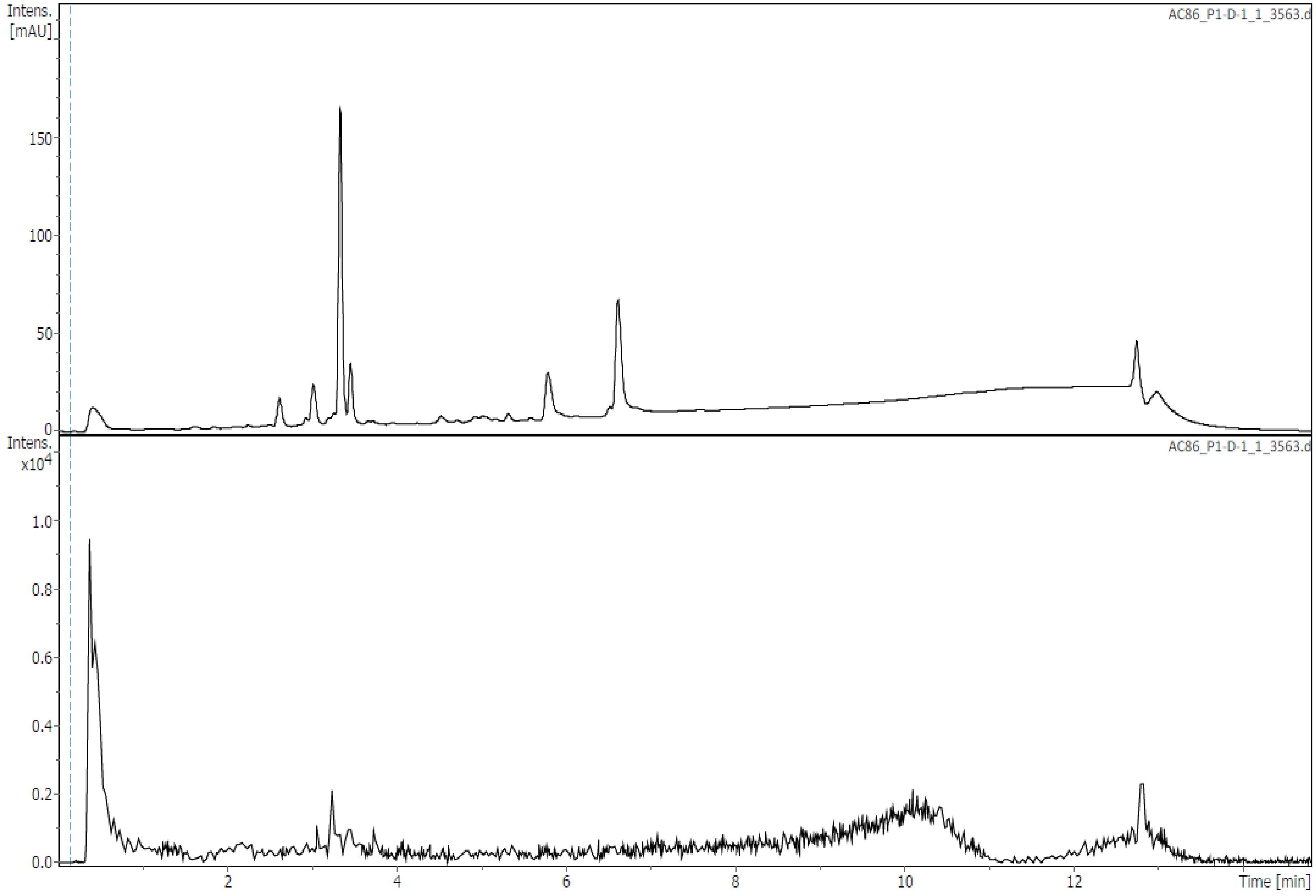

Figure S34 LC-HRMS of reaction between **24** and **18**. Top diagram shows UV absorbance (260 nm). Bottom diagram shows extracted ion current (± 0.5 Da) for the expected product ($[M+H]^+$), which could not be found.

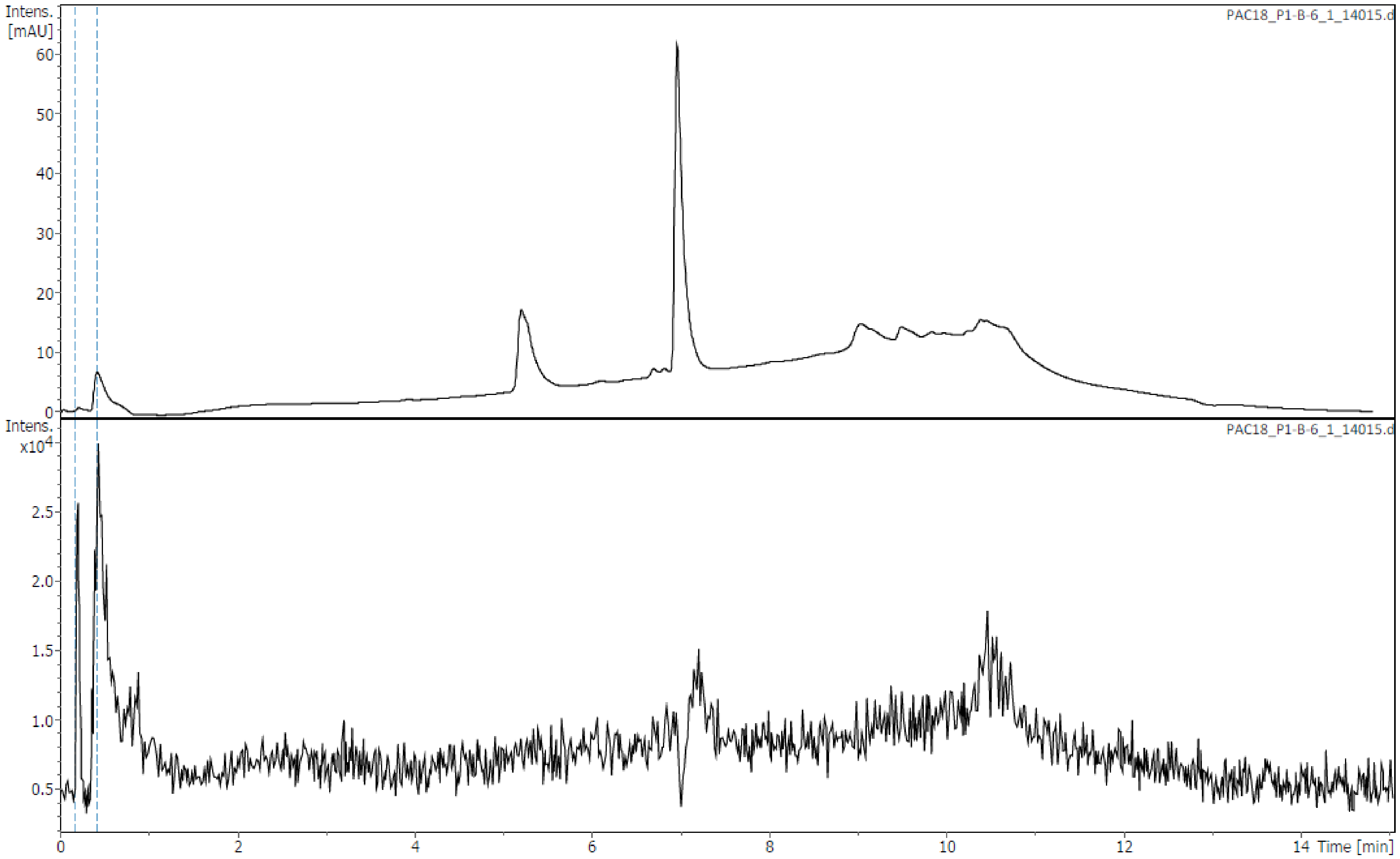

Figure S35 LC-HRMS of negative control reaction between **24** and **18**. All reaction components were included apart from the enzyme. Top diagram shows total ion current. Bottom diagram shows extracted ion current (± 0.5 Da) for the expected product ([M+H]+), which could not be found.

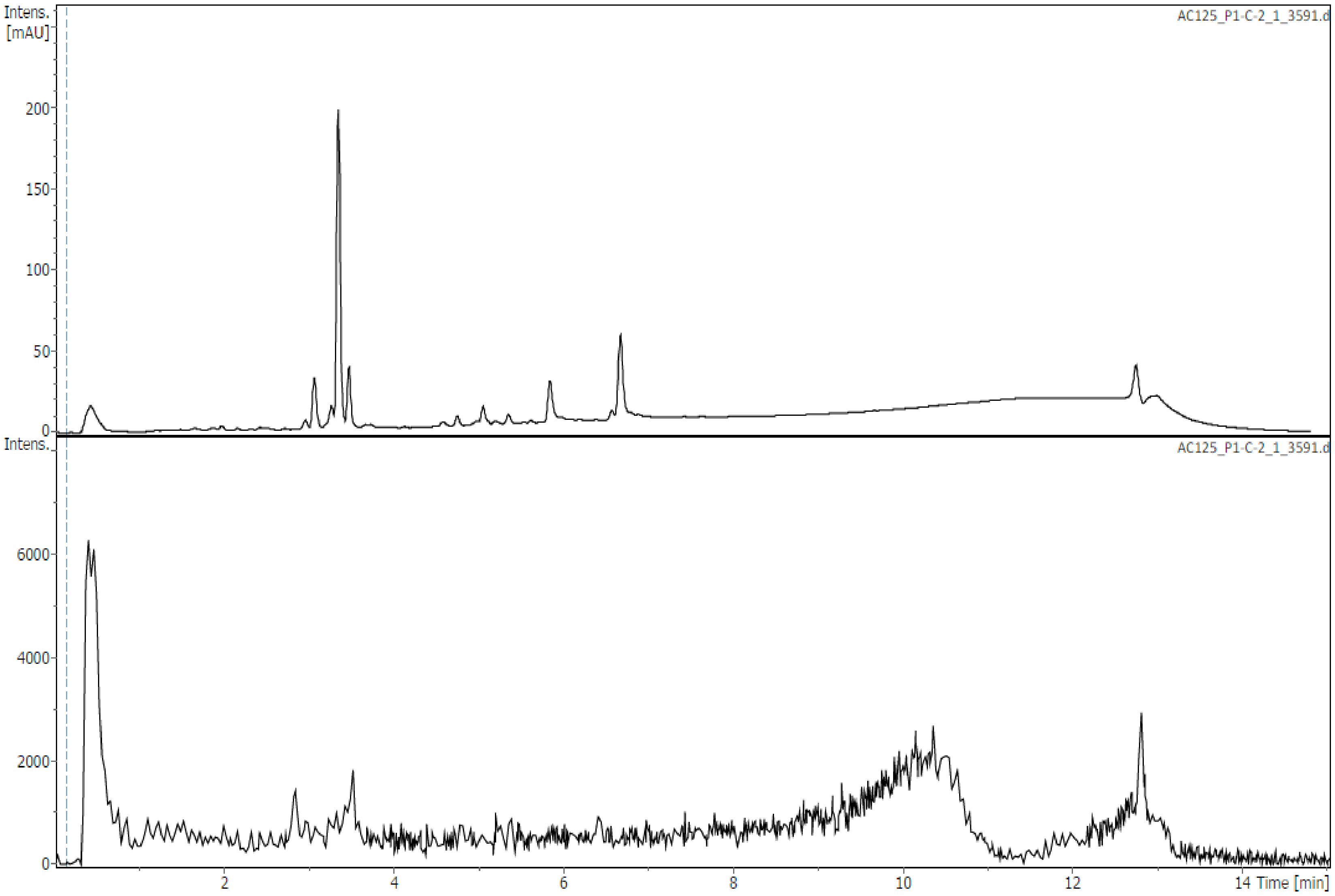

Figure S36 LC-HRMS of reaction between **24** and **19**. Top diagram shows UV absorbance (260 nm). Bottom diagram shows extracted ion current (± 0.5 Da) for the expected product ($[M+H]^+$), which could not be found.

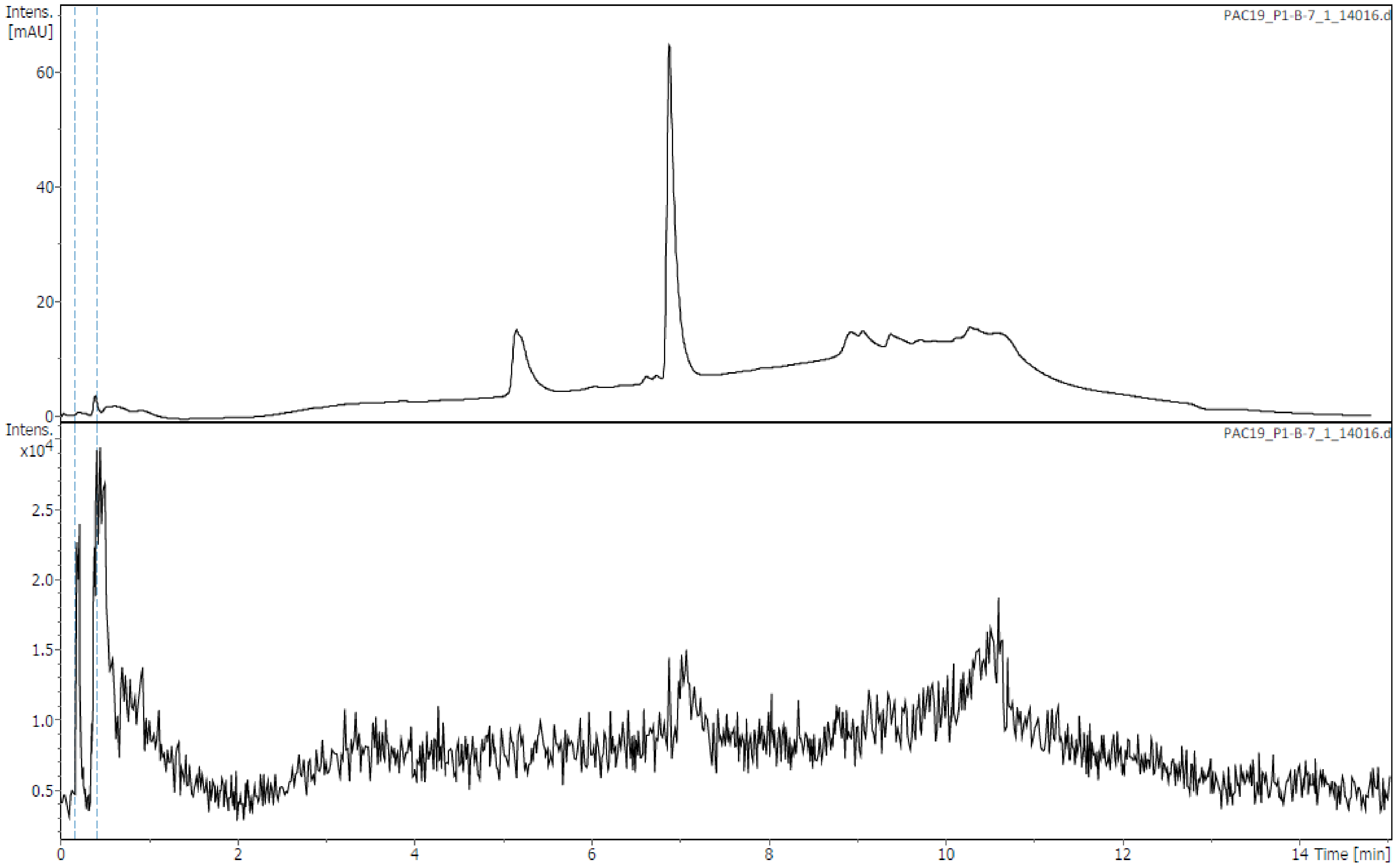

Figure S37 LC-HRMS of negative control reaction between **24** and **19**. All reaction components were included apart from the enzyme. Top diagram shows total ion current. Bottom diagram shows extracted ion current (± 0.5 Da) for the expected product ([M+H]+), which could not be found.

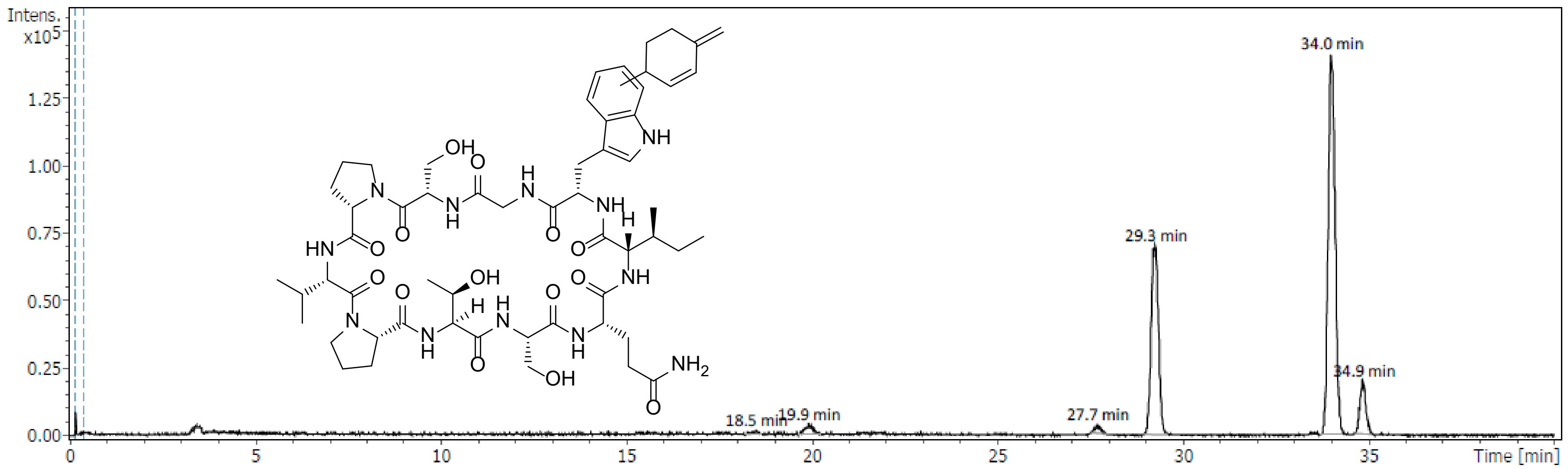
Intens.
x10^5
1.25
1.00
0.75
0.50
0.25
0.00
0
5
10
15
20
25
30
35
Time [min]
34.0 min
29.3 min
34.9 min
27.7 min
18.5 min
19.9 min
OH
O
H
N
NH
H
HN
O
OH
O
N
NH
H
N
H
O
NH2

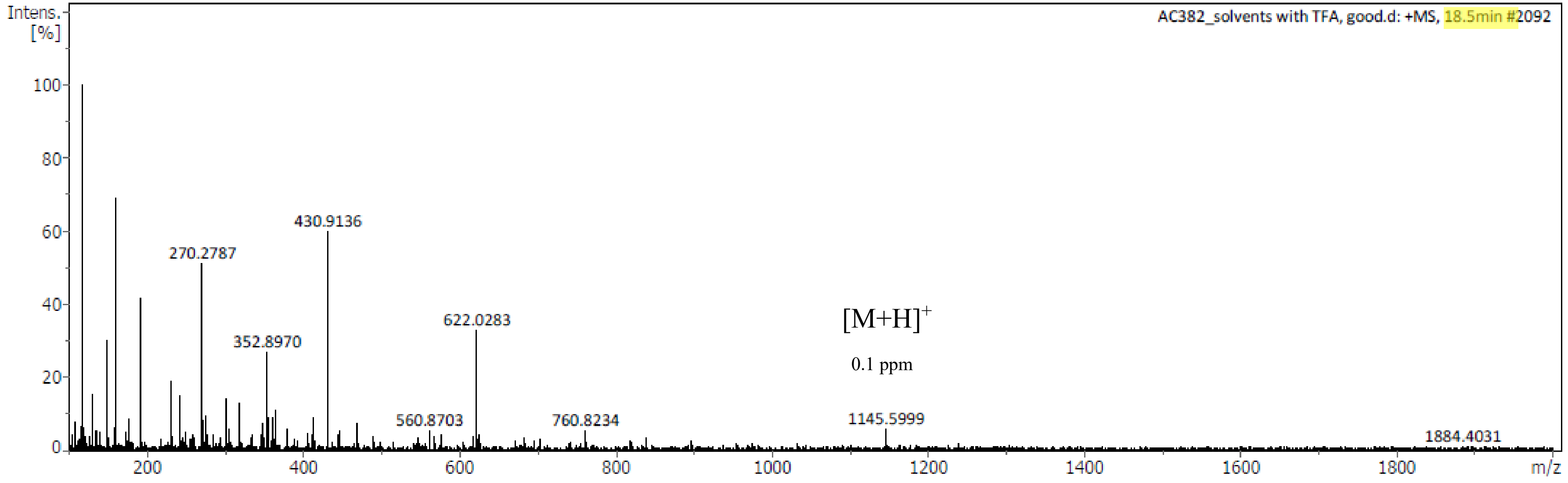
Intens.
[%]
AC382_solvents with TFA, good.d: +MS, 18.5min #2092
100
80
60
40
20
0
270.2787
430.9136
352.8970
622.0283
560.8703
760.8234
1145.5999
1884.4031
[M+H]+
0.1 ppm
200
400
600
800
1000
1200
1400
1600
1800
m/z

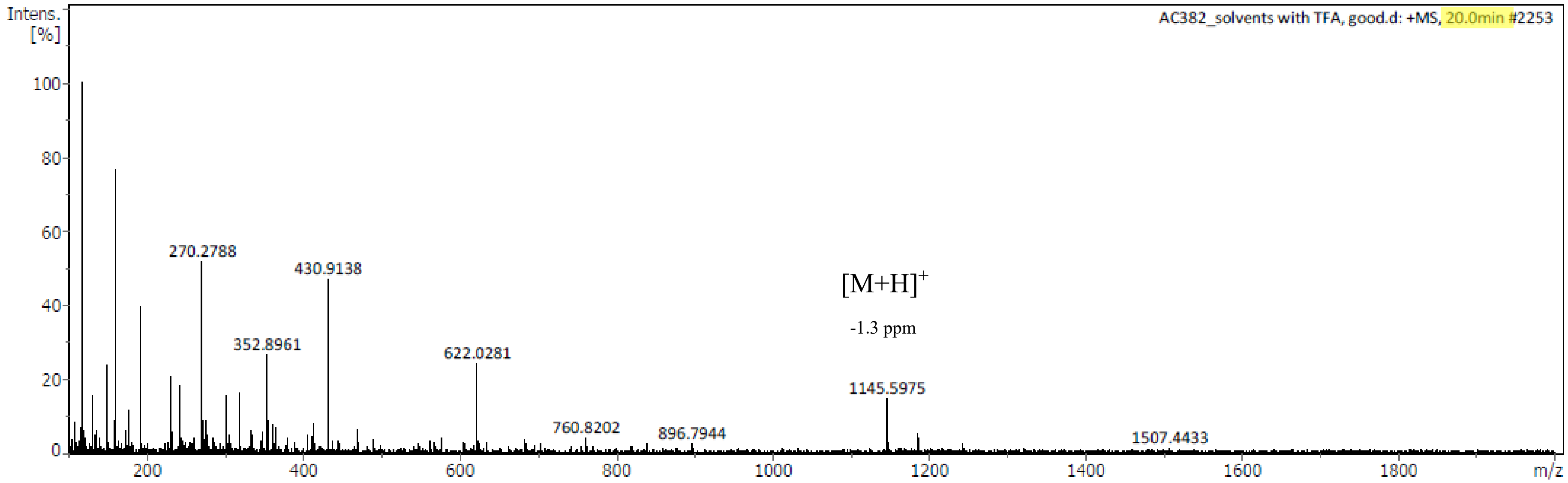
AC382_solvents with TFA, good.d: +MS, 20.0min #2253
Intens. [%]
270.2788
430.9138
352.8961
622.0281
760.8202
896.7944
[M+H]+
-1.3 ppm
1145.5975
1507.4433
m/z

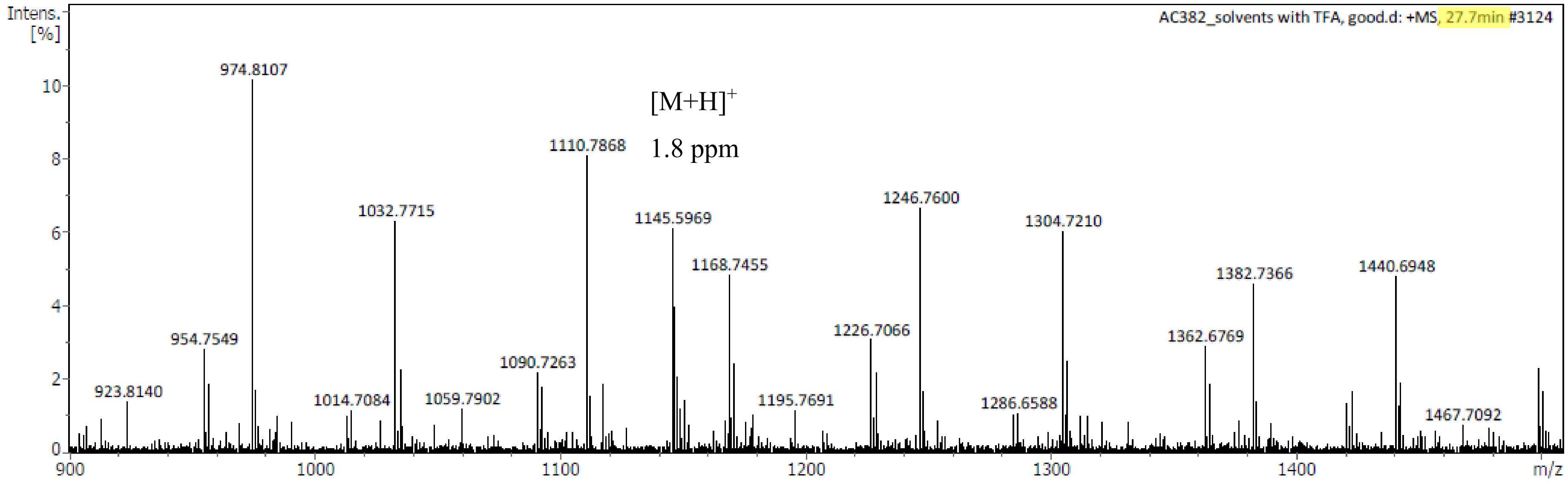
AC382_solvents with TFA, good.d: +MS, 27.7min #3124
Intens. [%]
974.8107
[M+H]+
1.8 ppm
1110.7868
1032.7715
1145.5969
1246.7600
1304.7210
1168.7455
1382.7366
1440.6948
954.7549
1226.7066
1362.6769
1090.7263
923.8140
1014.7084
1059.7902
1195.7691
1286.6588
1467.7092
m/z

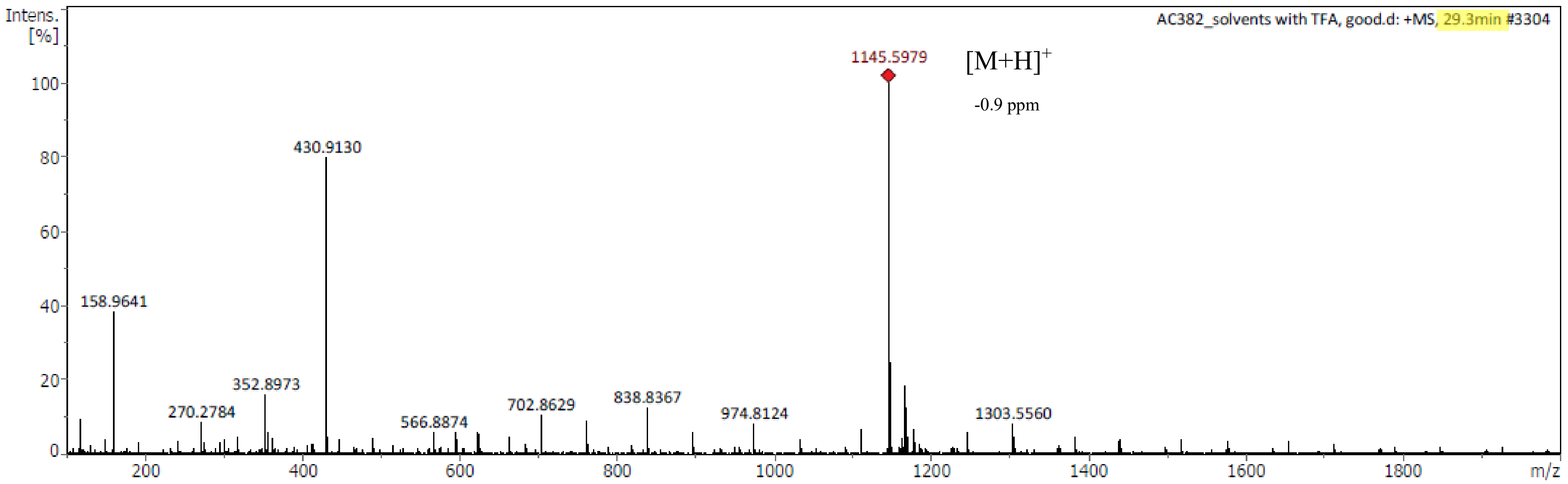
Intens. [%]
AC382_solvents with TFA, good.d: +MS, 29.3min #3304
1145.5979
[M+H]+
-0.9 ppm
430.9130
158.9641
352.8973
270.2784
566.8874
702.8629
838.8367
974.8124
1303.5560
m/z

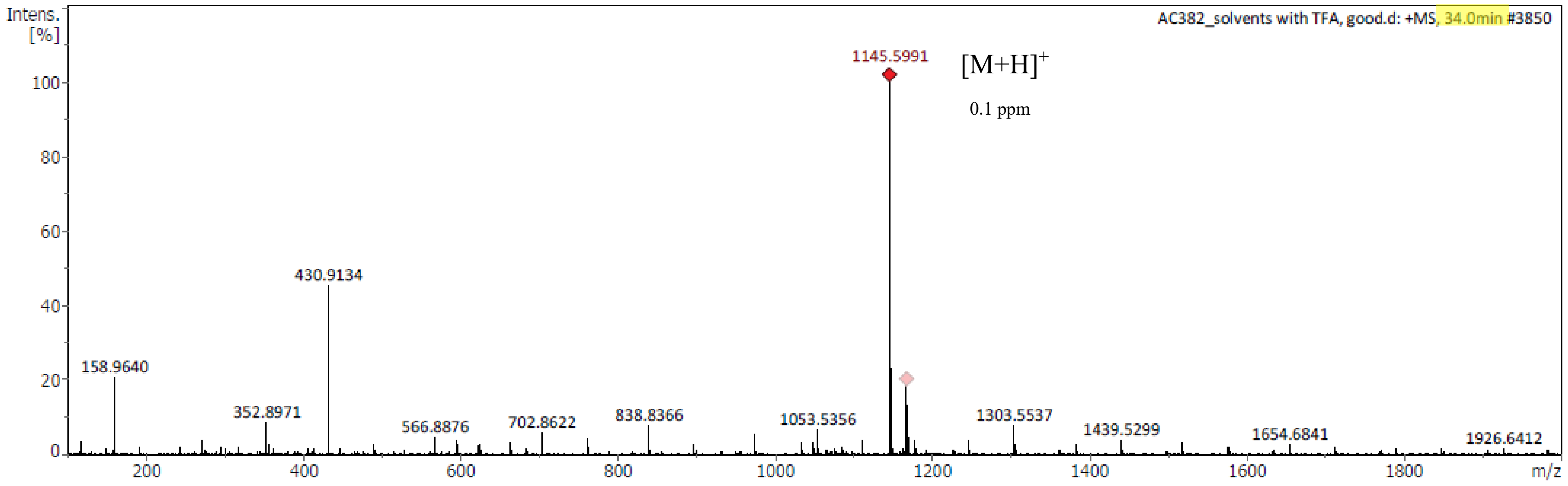
Intens. [%]
AC382_solvents with TFA, good.d: +MS, 34.0min #3850
1145.5991
[M+H]+
0.1 ppm
430.9134
158.9640
352.8971
566.8876
702.8622
838.8366
1053.5356
1303.5537
1439.5299
1654.6841
1926.6412
m/z

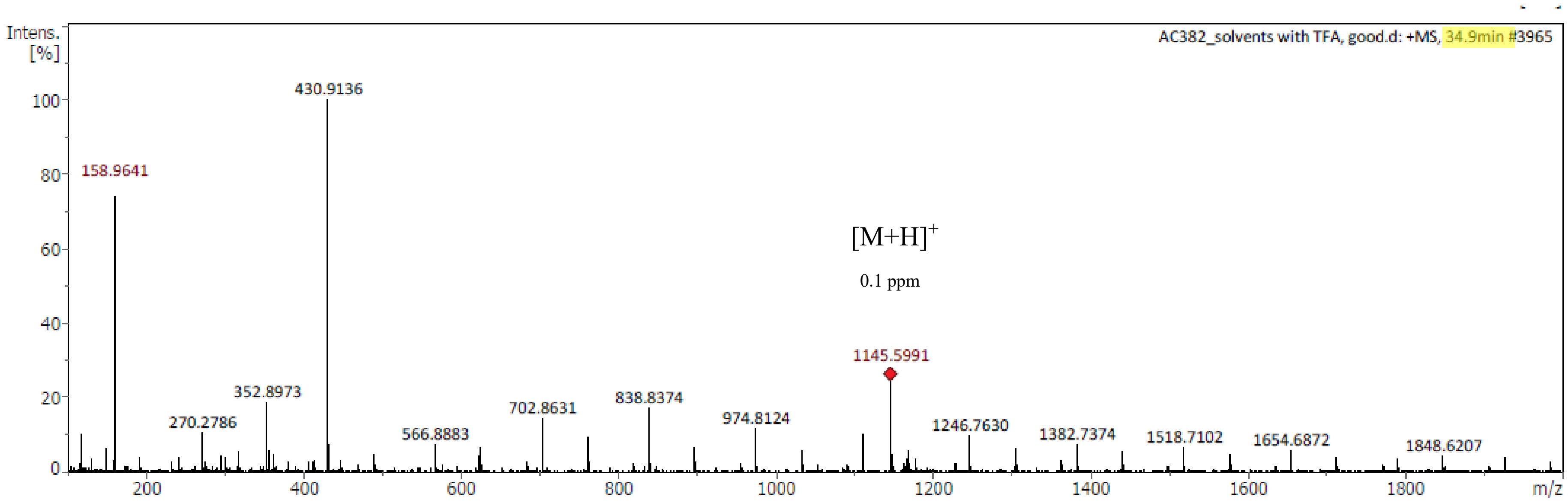

Figure S38 LC-HRMS of reaction between **24** and **20**. Top diagram shows the extracted ion current (± 0.5 Da) for the expected product. Six products were observed at T_R 18.5, 19.9, 27.7, 29.3, 34.0 and 34.9 min. Bottom diagrams show the high resolution mass spectrum of each product.

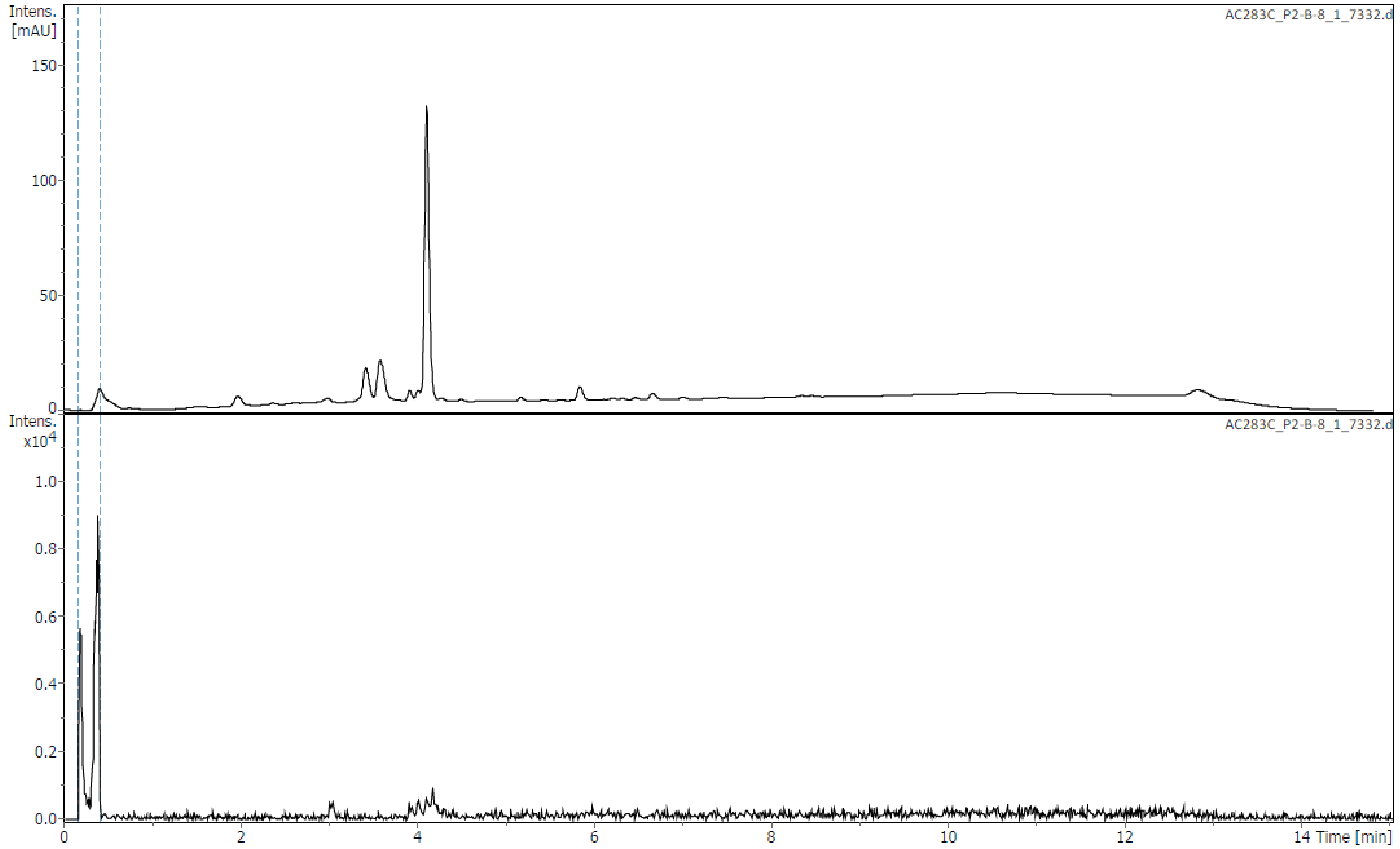

Figure S39 LC-HRMS of negative control reaction between **24** and **20**. All reaction components were included apart from the enzyme. Top diagram shows total ion current. Bottom diagram shows extracted ion current (± 0.5 Da) for the expected product ([M+H]+), which could not be found.

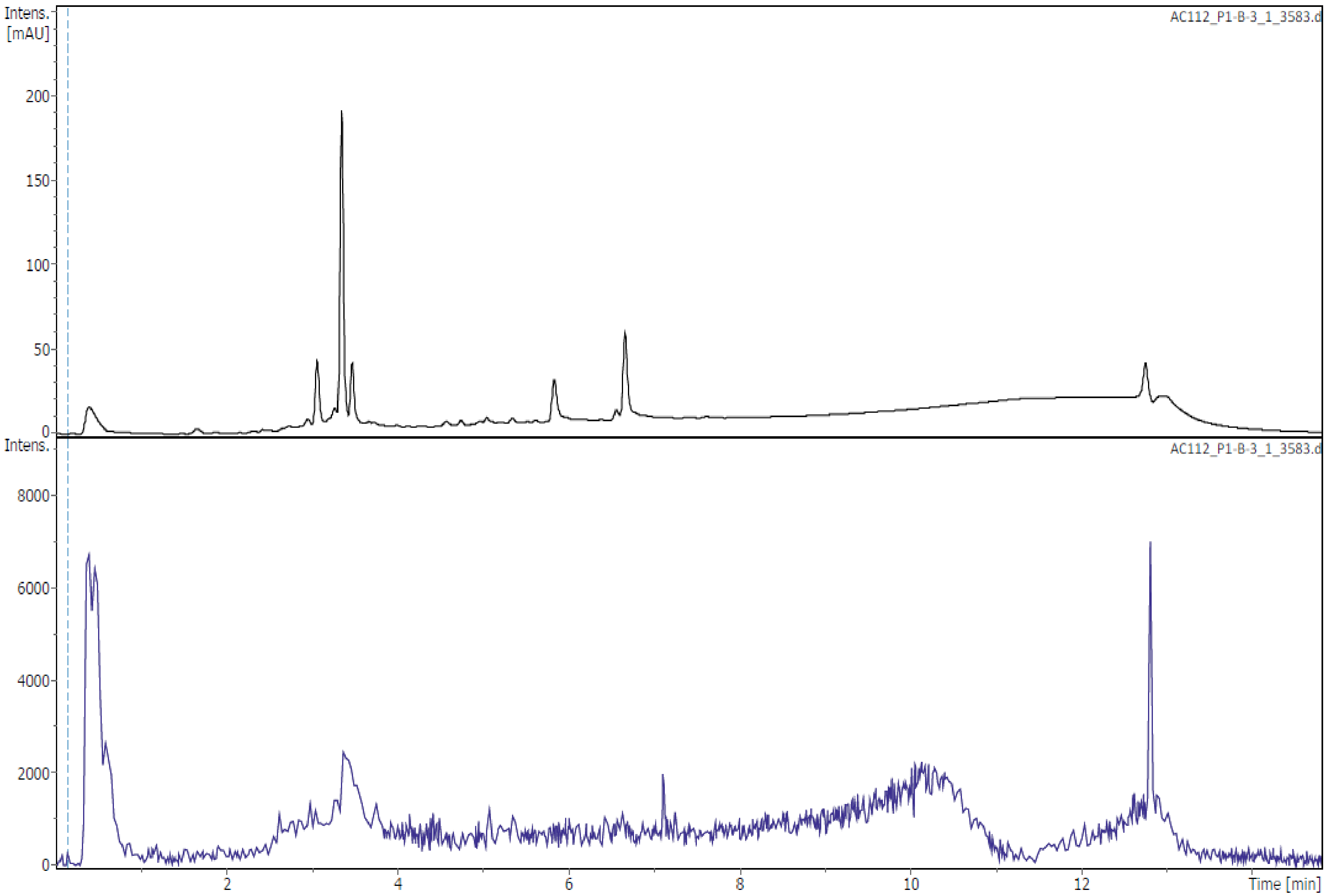

Figure S40 LC-HRMS of reaction between **24** and **21**. Top diagram shows UV absorbance (260 nm). Bottom diagram shows extracted ion current (± 0.5 Da) for the expected product ([M+H]$^+$), which could not be found.

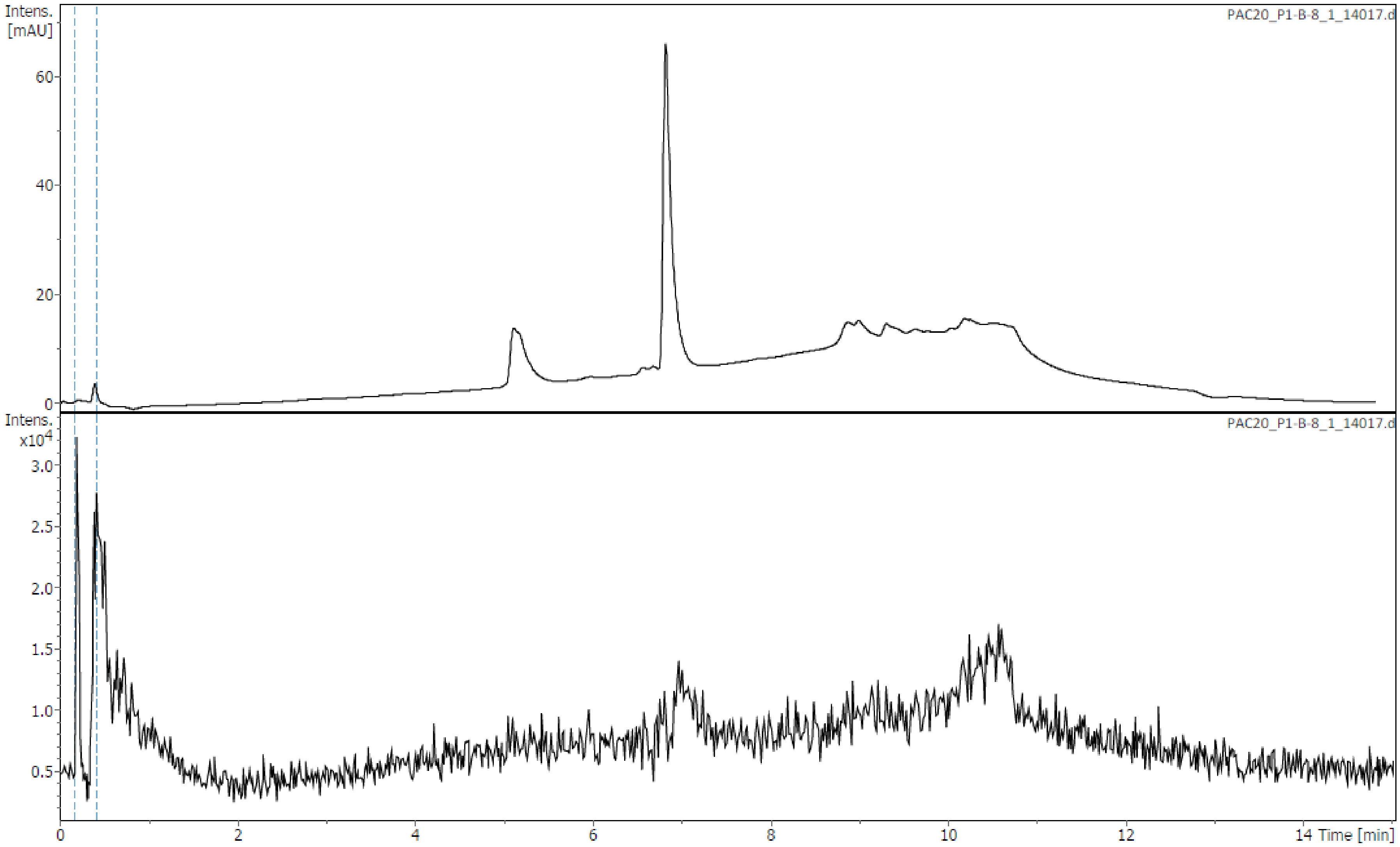

Figure S41 LC-HRMS of negative control reaction between **24** and **21**. All reaction components were included apart from the enzyme. Top diagram shows total ion current. Bottom diagram shows extracted ion current (± 0.5 Da) for the expected product ([M+H]+), which could not be found.

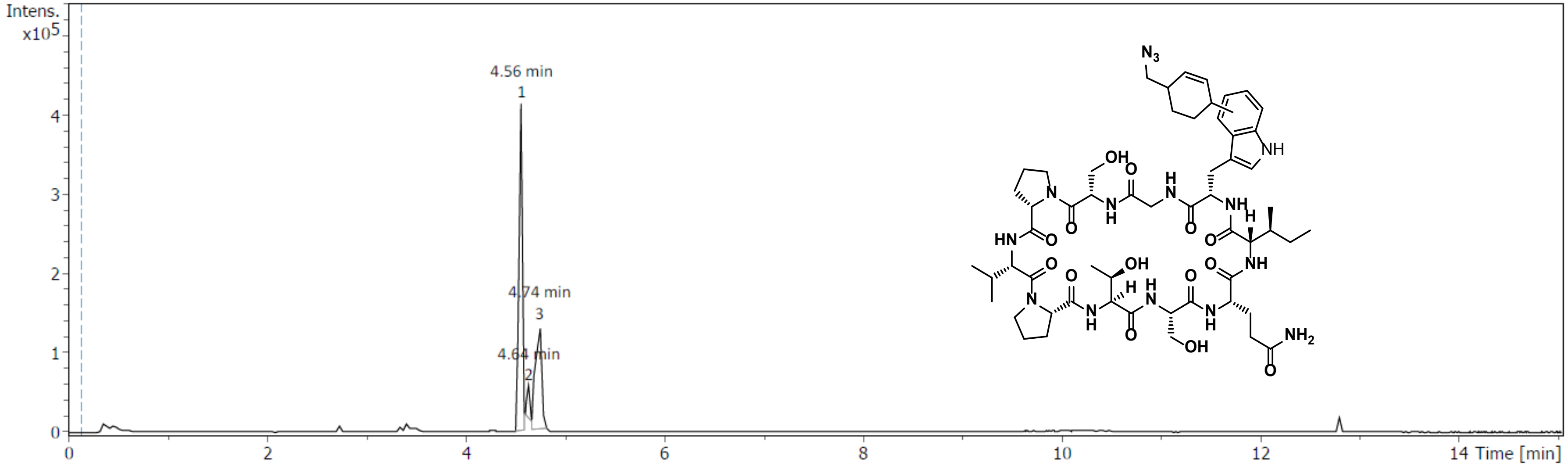

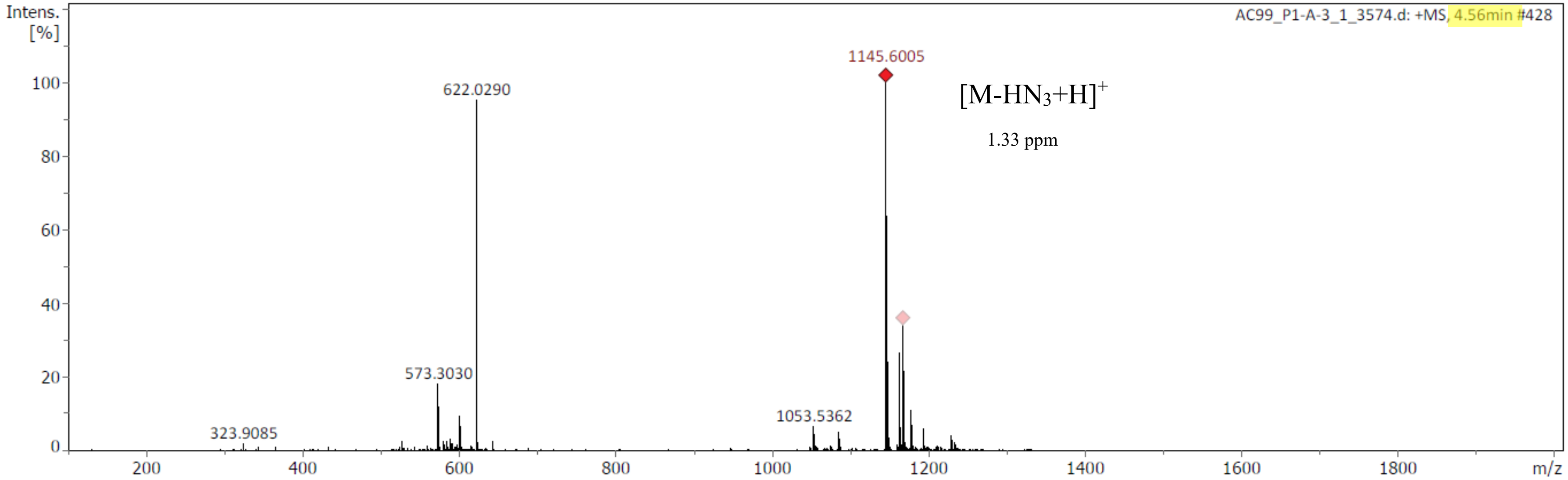

[M-HN$_3$+H]$^+$

1.33 ppm

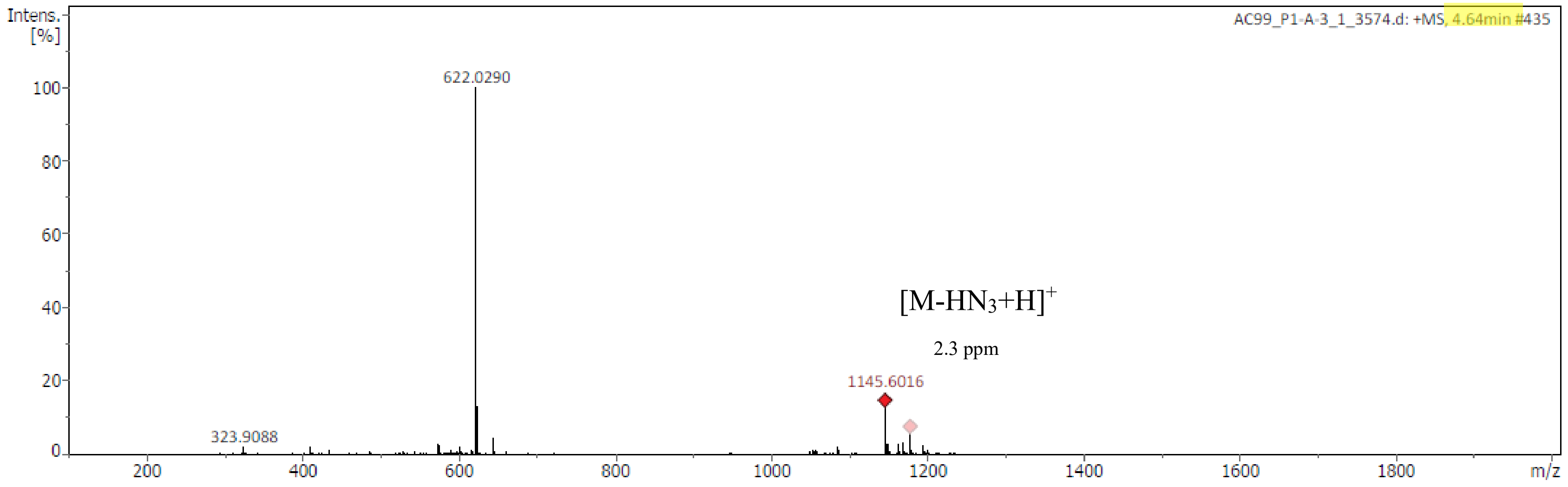

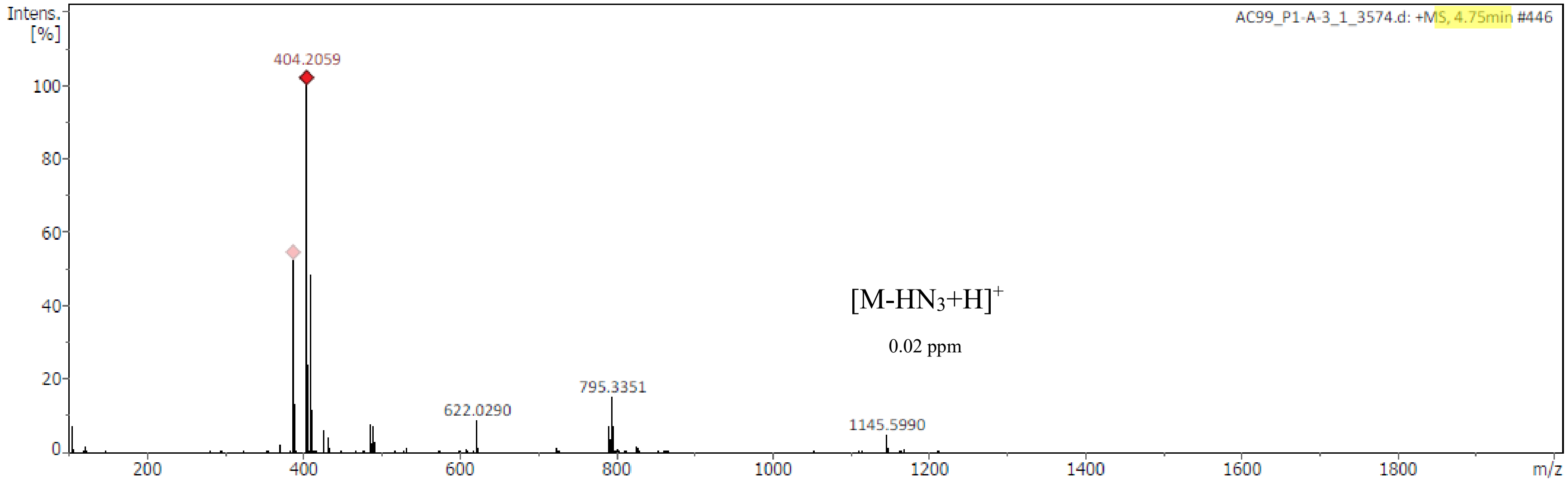

Figure S42 LC-HRMS of reaction between **24** and **22**. Top diagram shows the extracted ion current for the expected product (M-HN_3). Three products were observed at T_R 4.56, 4.64 and 4.74 min. Bottom diagram show the high resolution mass spectrum of each product.

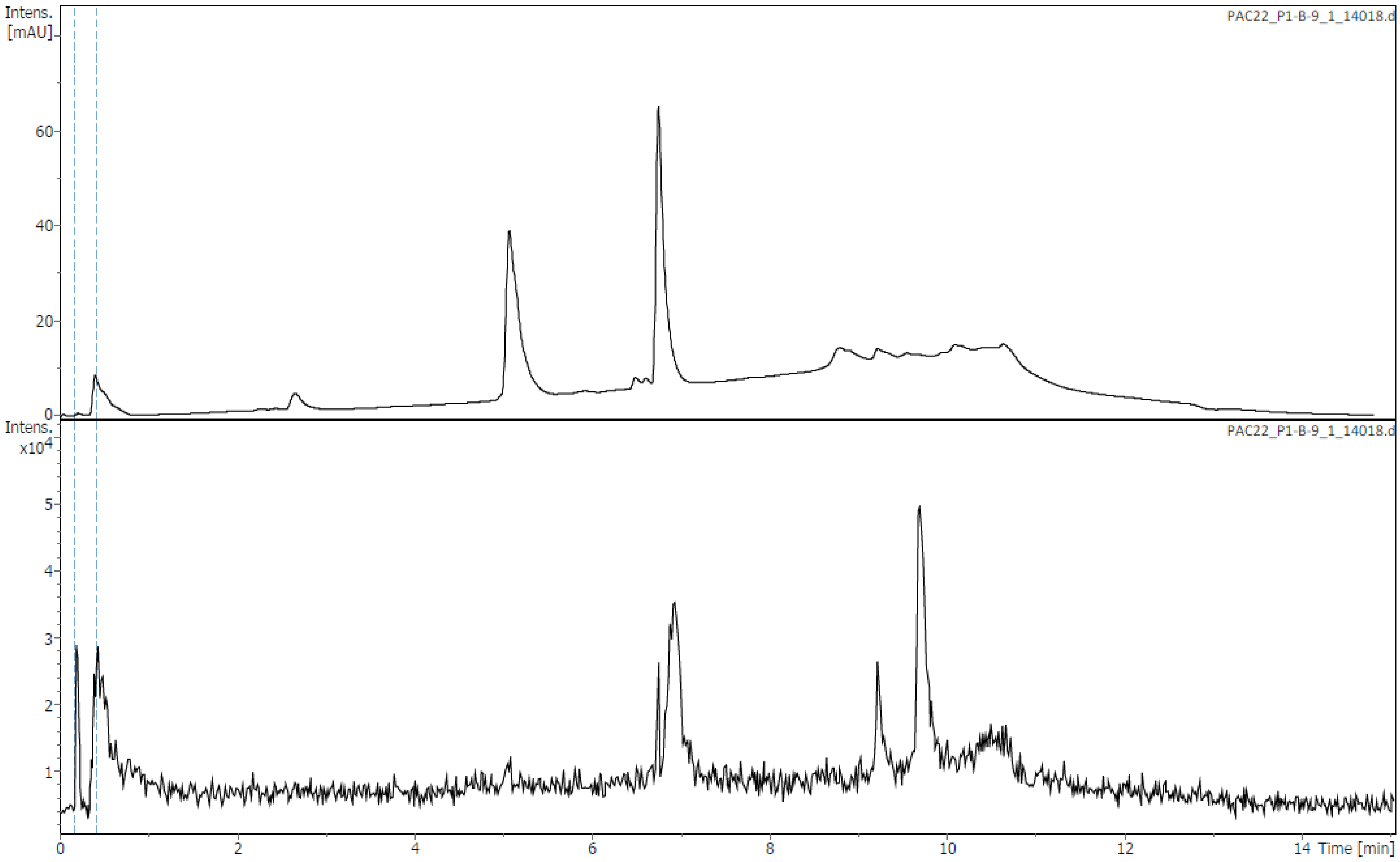

Figure S43 LC-HRMS of negative control reaction between **24** and **22**. All reaction components were included apart from the enzyme. Top diagram shows total ion current. Bottom diagram shows extracted ion current (± 0.5 Da) for the expected product (M-HN_3), which could not be found.

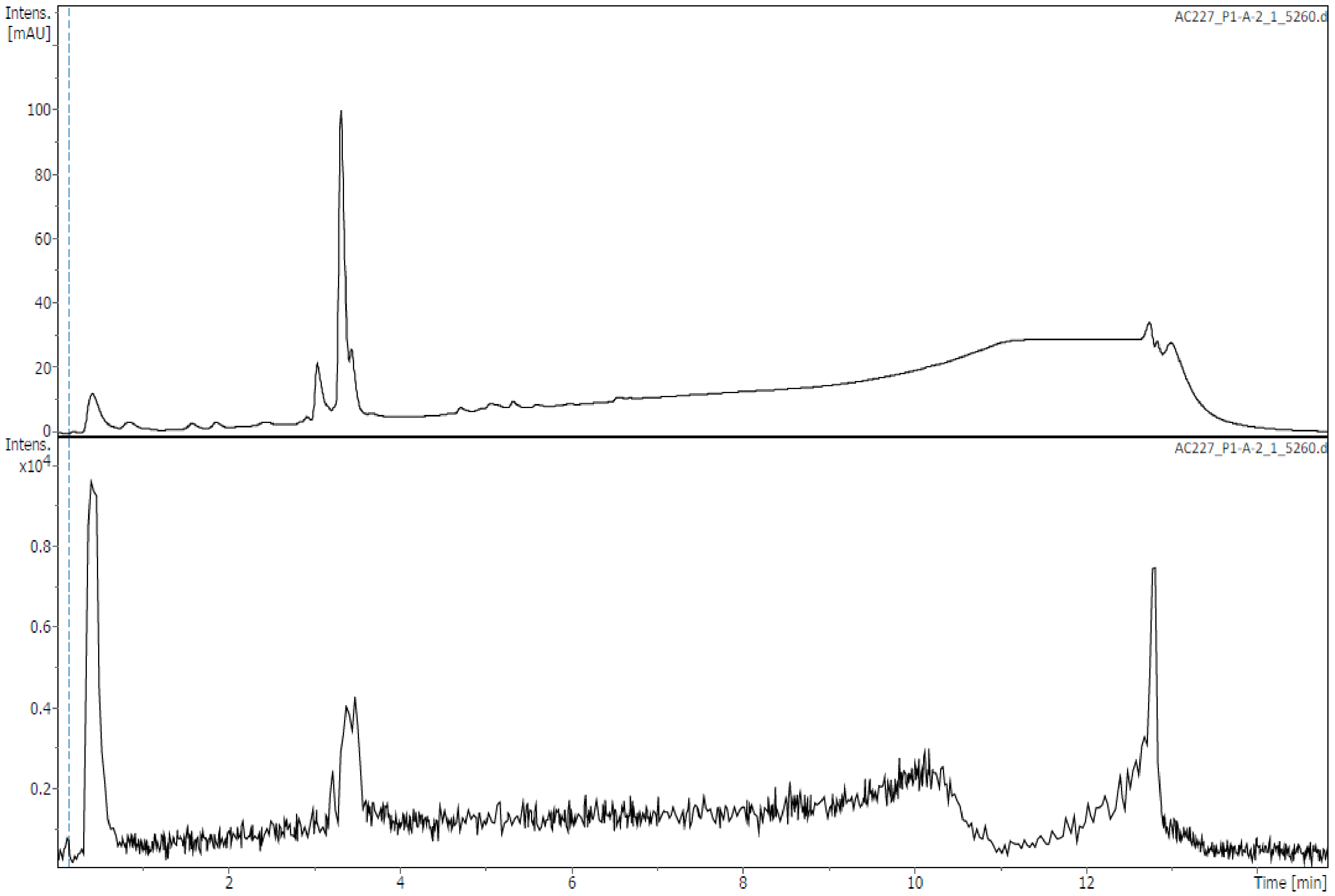

Figure S44 LC-HRMS of reaction between **24** and **23**. Top diagram shows UV absorbance (260 nm). Bottom diagram shows extracted ion current (± 0.5 Da) for the expected product ($[M+H]^+$), which could not be found

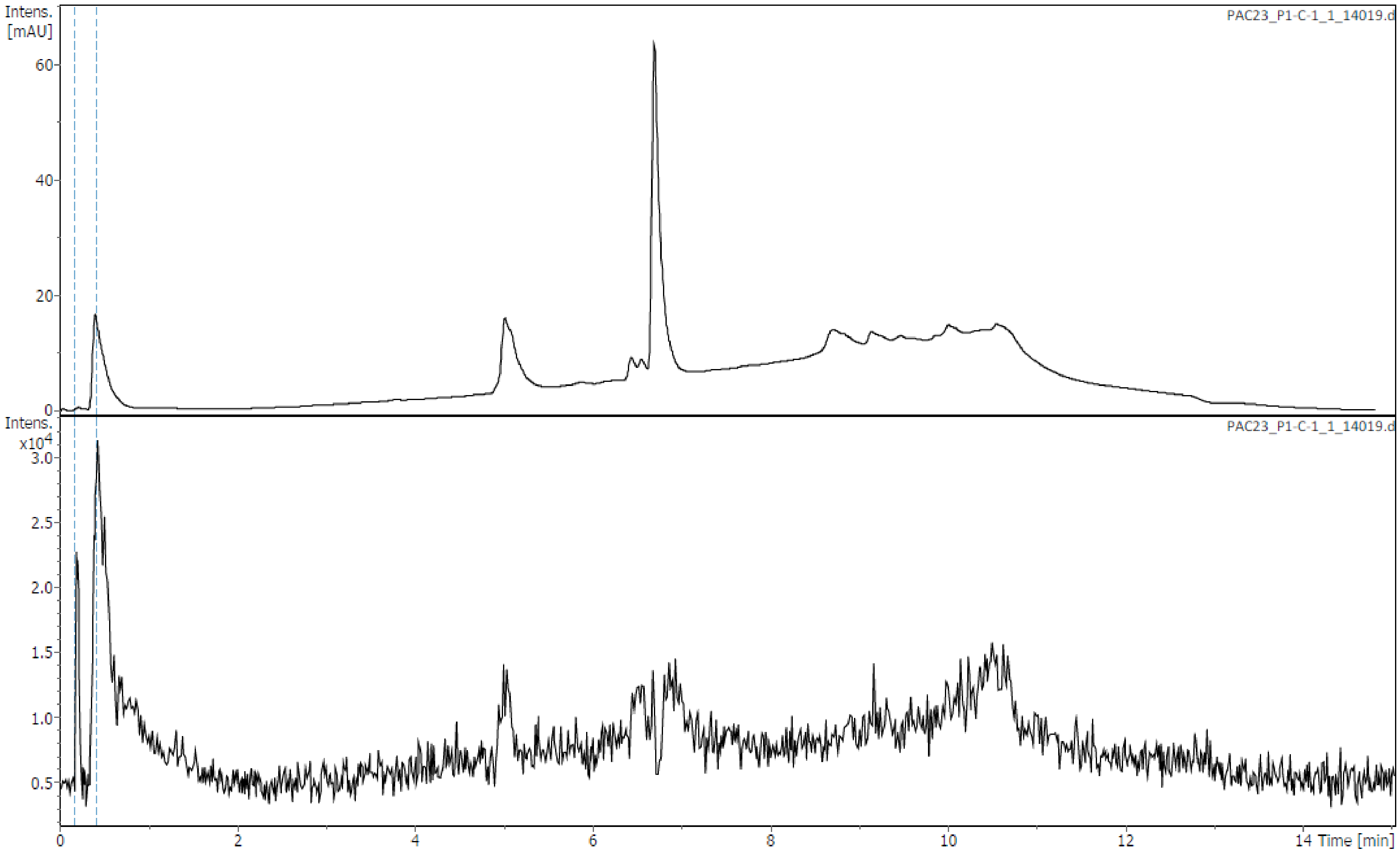

Figure S45 LC-HRMS of negative control reaction between **24** and **23**. All reaction components were included apart from the enzyme. Top diagram shows total ion current. Bottom diagram shows extracted ion current (± 0.5 Da) for the expected product ([M+H]+), which could not be found

2.4 Isolation of 25

General protocol 2.2 was scaled up to 30 mL total reaction volume. After incubation, the reaction was subsequently diluted 1:1 (v/v) with methanol, cleared by centrifugation, concentrated under reduced pressure, and freeze-dried. The residue was purified by semipreparative HPLC (Column ACE 5 C18-HL 250x10.0 mm, solvent A water + 0.1% TFA, B acetonitrile + 0.1% TFA; flow 4.7 mL/min. Gradient: 0 min 5% B; 15 min 100% B) to yield 0.9 mg of desired product.

Unfortunately, the isolated product decomposed upon removal of HPLC solvent mixture with a rotary evaporator set at 37 °C, yielding unmodified starting peptide.

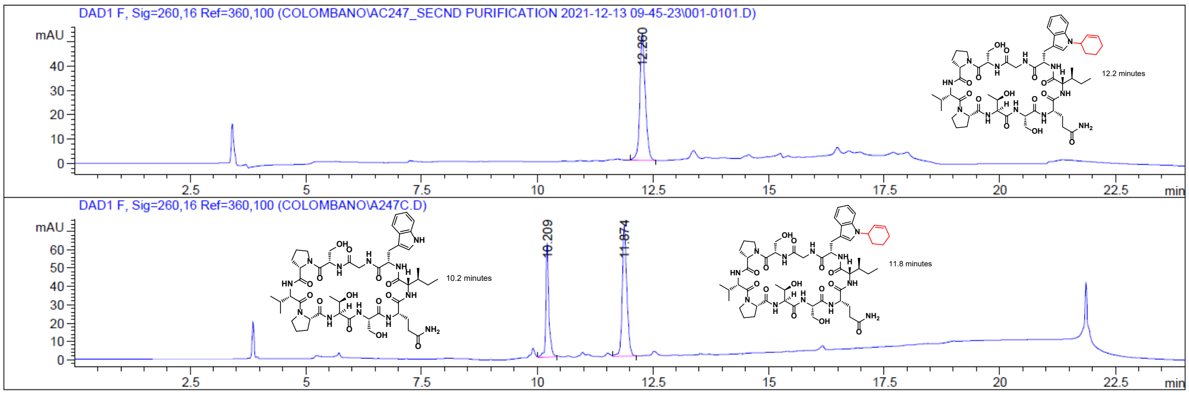

Figure S46 Top diagram shows **25** (12.2 min.) immediately after HPLC purification. Bottom diagram shows partial decomposition of **25** (11.8 min) to **24** (10.2 min) after heating at 37 °C in the rotary evaporator bath.

2.5 Kinetic parameters determination

Steady-state kinetic parameters for both cofactor **20** and **1** were assessed using 0.5 mM of cyclic peptide **24**, 20 µM of AcyF enzyme and variable concentrations of the cofactor **20** or **1** (0.1-2 mM) and were determined on the basis of product formation as monitored by LC-HRMS. The initial velocities (V_0) were determined using "stopped assays" approach[23]. Reactions were stopped at a pre-determined timepoint at which the product formation rate is in the linear phase. This timepoint was determined by sampling the reaction with the highest compound concentration at different time points to determine the time range in which the rate of product formation is linear. Since this is the reaction that reaches its steady state faster than all the others, choosing a stopping time that falls within the linear phase implies that also the reactions with lower cofactor concentrations will necessarily still be in their linear phase. Assays were conducted in triplicate and the kinetic curve was fit to Michaelis–Menten kinetics using Prism 5.04 (GraphPad Software, Inc. La Jolla, CA 92037 USA).

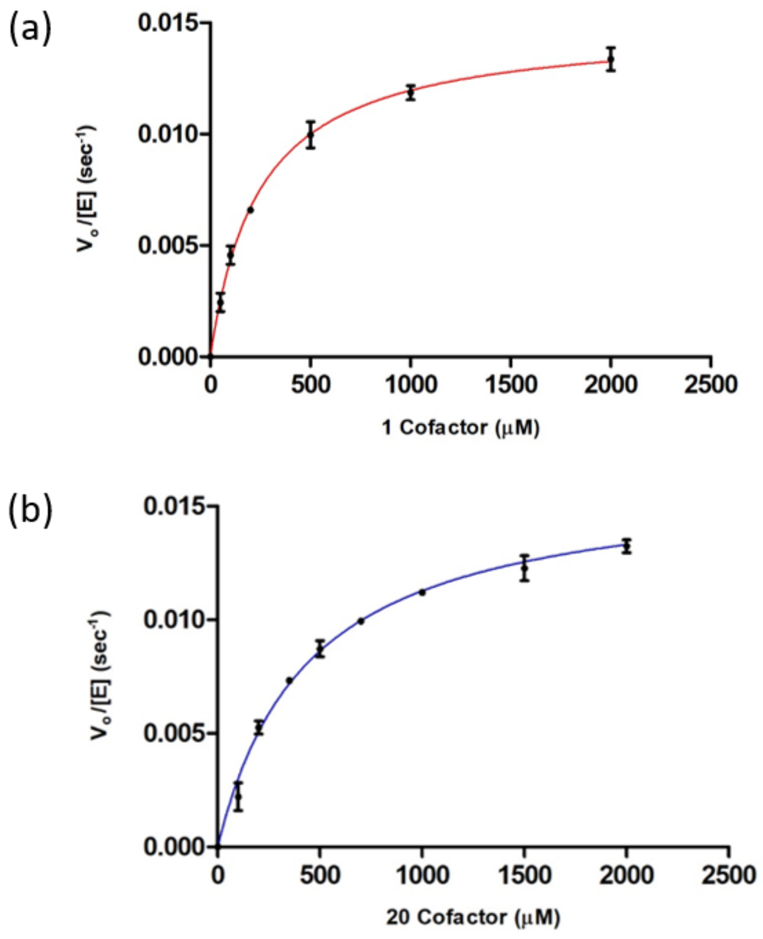

Figure S47 Michaelis-Menton kinetics for AcyF using 0.5 mM **24** and variable concentrations of **1** (a) or **20** (b) (0.01- 2 mM) in 10 mM HEPES buffer, 150 mM NaCl, $MgCl_2$ 12 mM, pH 7 at 30 °C. Experiments were done in duplicates and error bars indicate the SE of the mean.

2.6 Isolation of 26/27

A total volume of 45 mL biochemical reaction was setup using general protocol 2.2, but with 500 µM cyclic peptide instead. The reaction was incubated for 24 hours at 30 ºC and subsequently diluted to 1:1 (v/v) with methanol, cleared by centrifugation, concentrated under reduced pressure, and freeze-dried. The residue was purified by semipreparative HPLC (Column ACE phenyl 300 A, 250x10.0 mm, solvent A water + 0.1% TFA, B acetonitrile + 0.1% TFA; flow 4.7 mL/min. Gradient: 0 min 20% B; 35 min 40% B.), to yield 2.9 mg of desired product.

T_R =24.2 minutes.

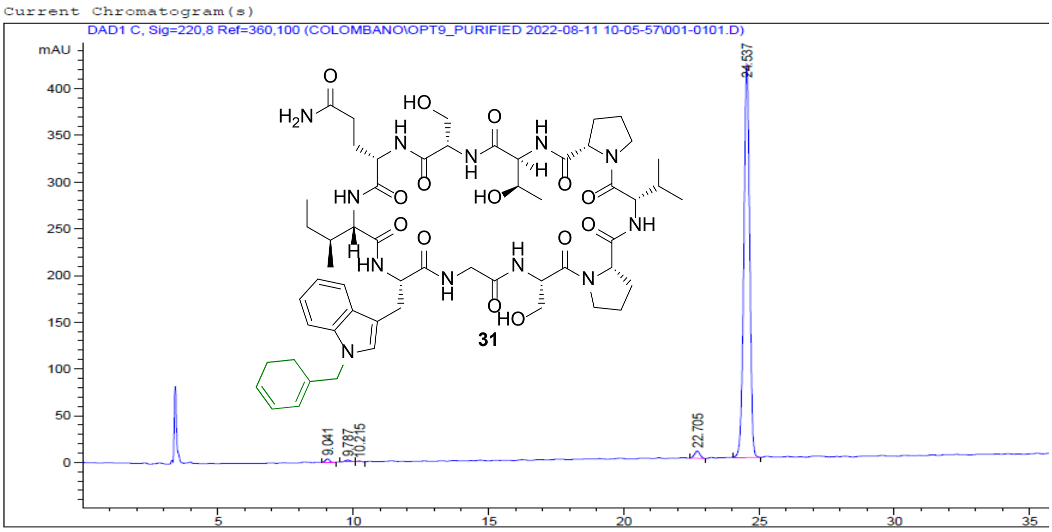

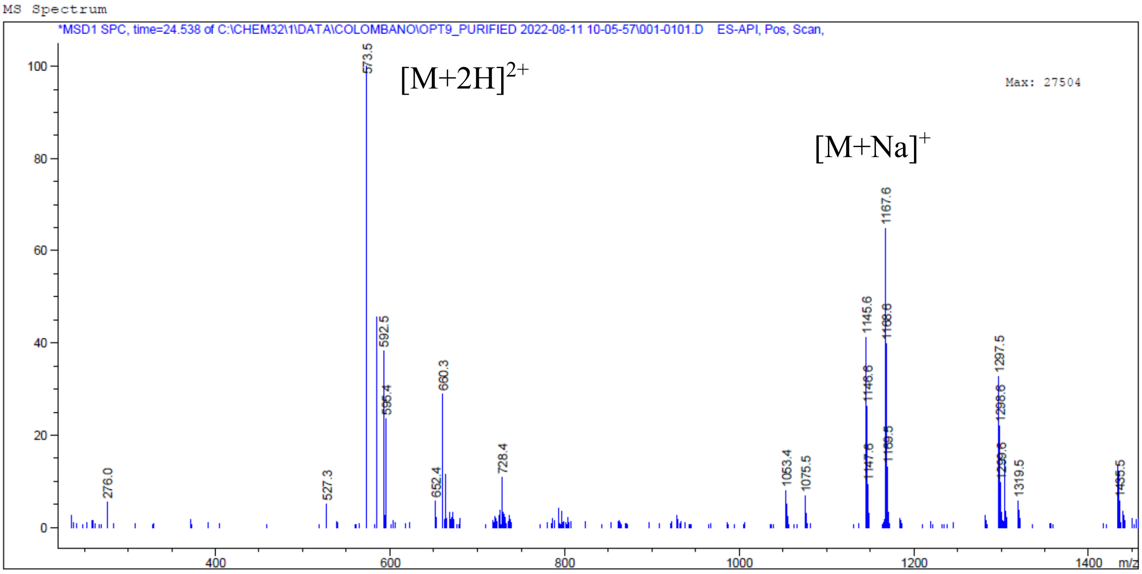

Figure S48 LC-MS for isolated **26/27**.

2.6.1 NMR data for 26/27

The following data were acquired on 800 MHz Bruker UltraStabilised 4 channel instrument with a 5 mm TCI 1H/13C/15N CryoProbe^{TM}.

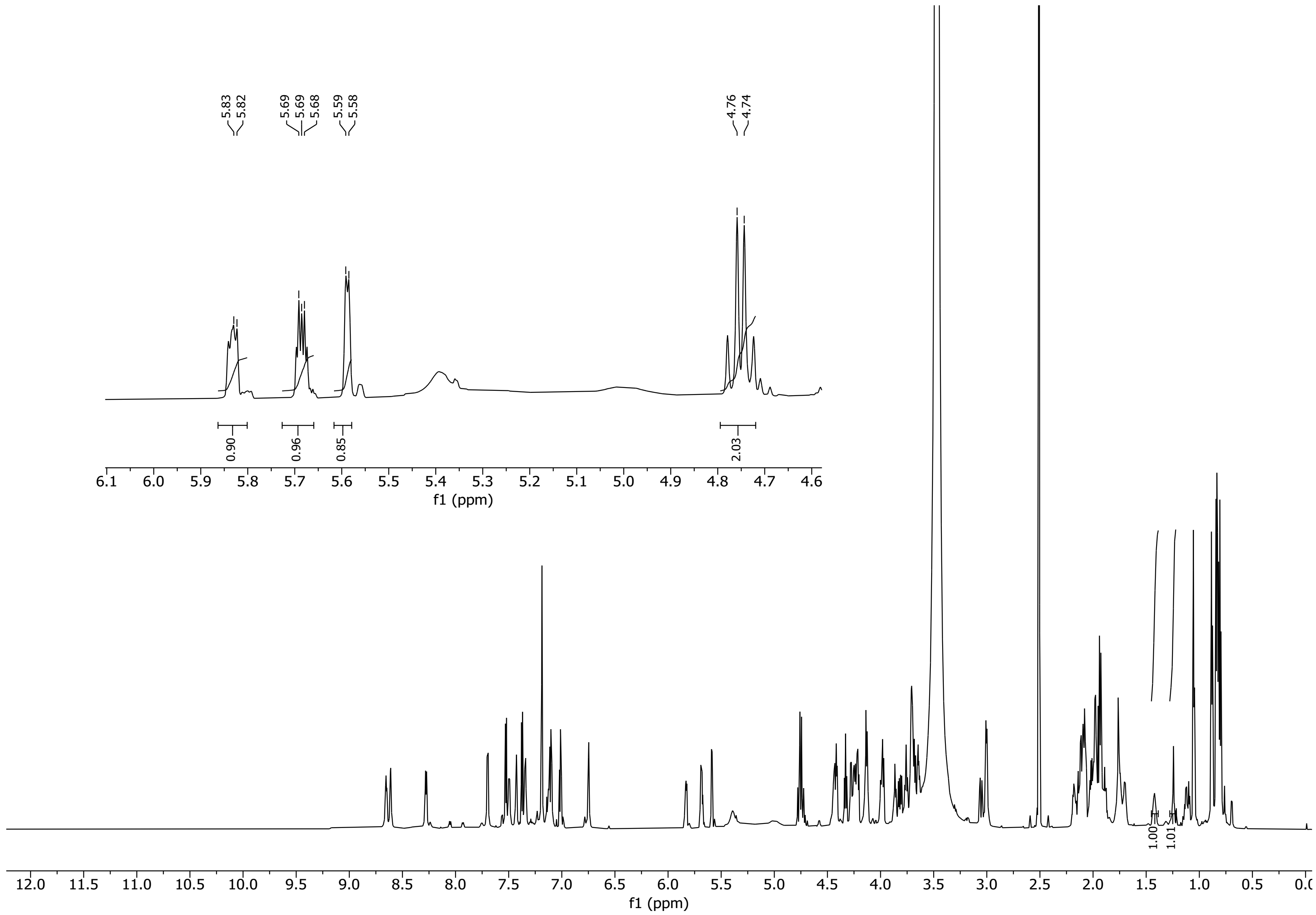

Figure S49 ^{1}H NMR (800 MHz) for **26/27** in DMSO-d_6.

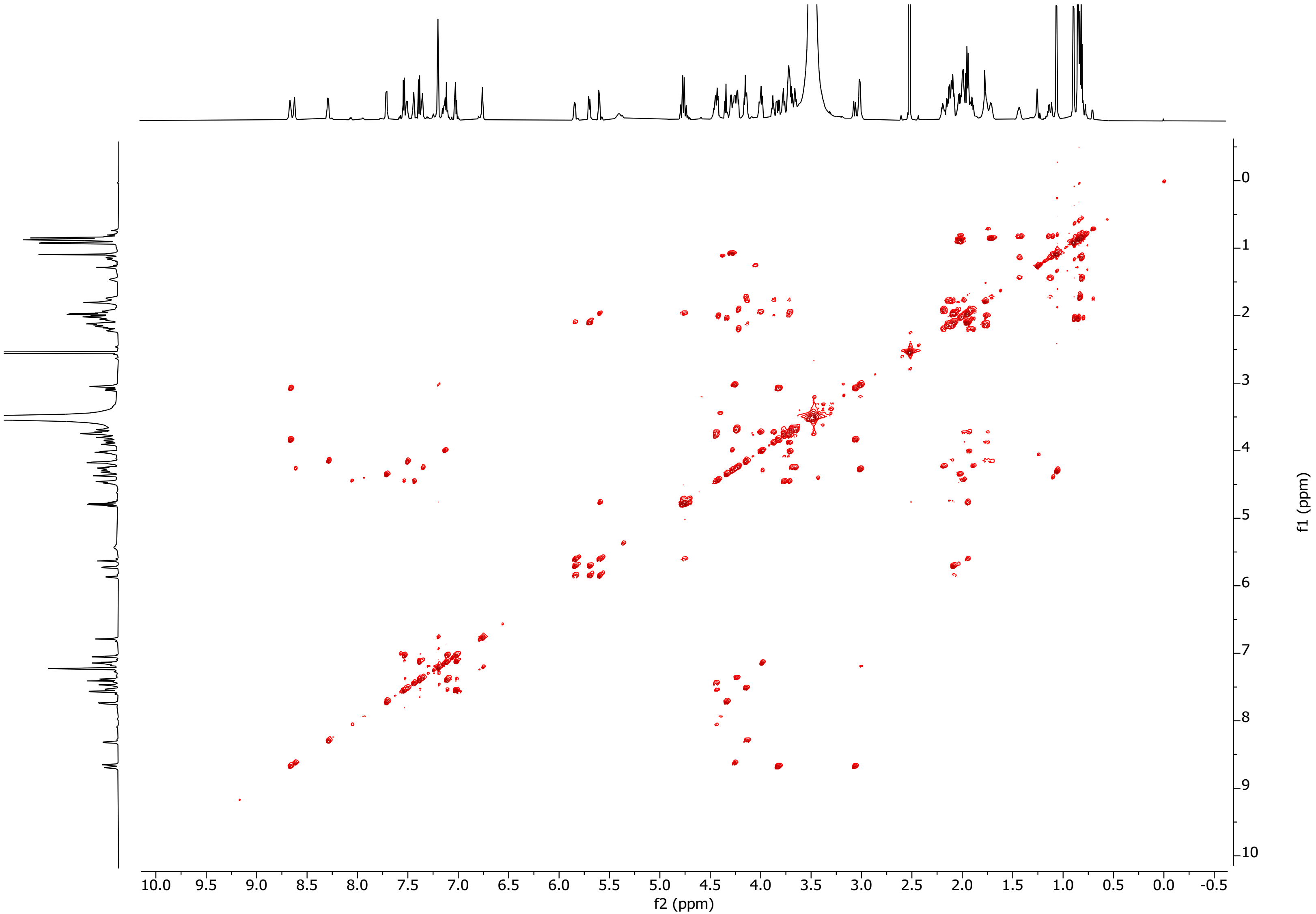

Figure S50 ^{1}H-^{1}H COSY NMR for **26/27** in DMSO-d_6.

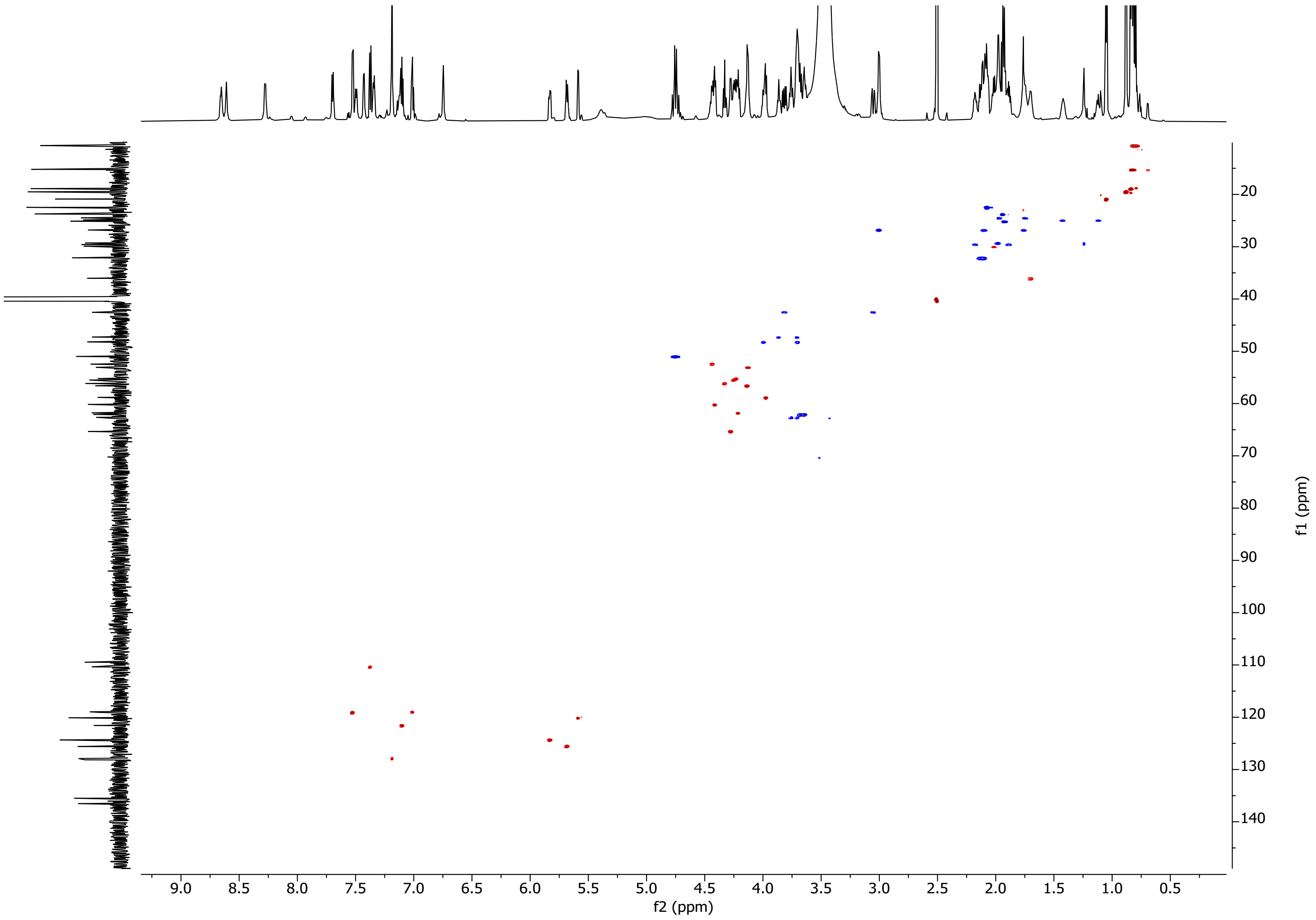

Figure S51 ¹H-¹³C HSQC NMR for **26/27** in DMSO-d_6.

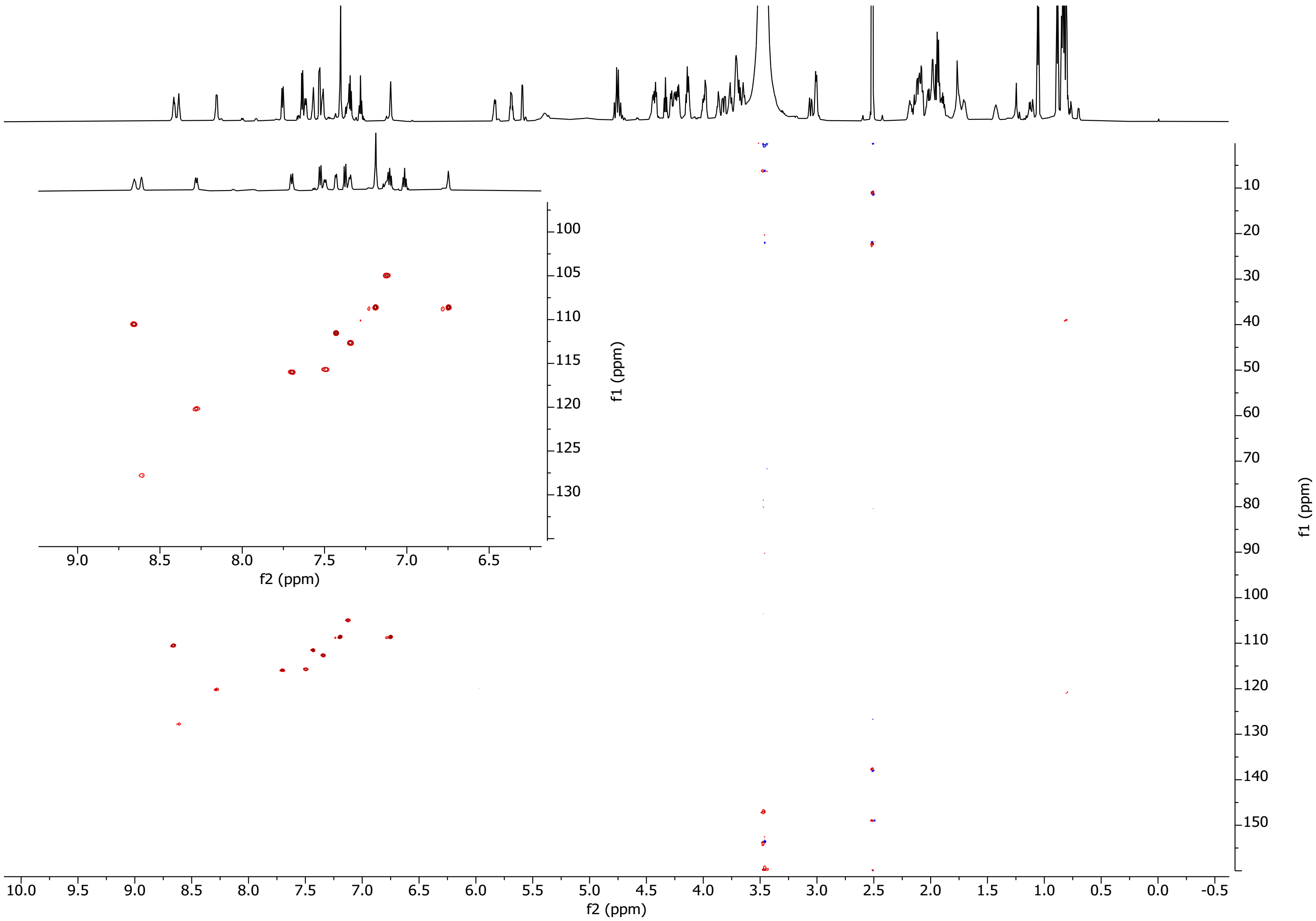

Figure S52 ^{1}H-^{15}N HSQC NMR for **26/27** in DMSO-d_6.

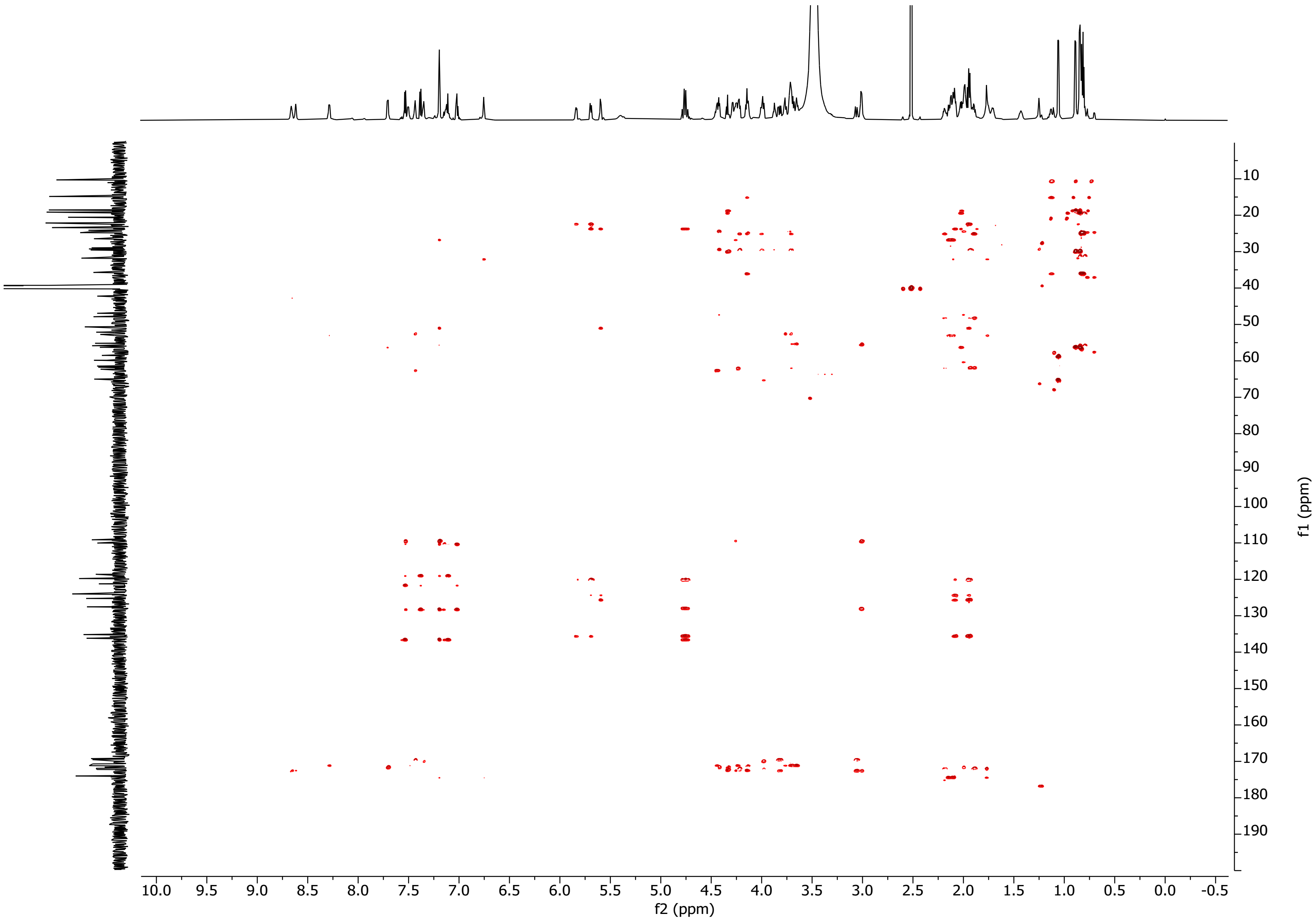

Figure S53 ^{1}H-^{13}C HMBC NMR for **26/27** in DMSO-d_6.

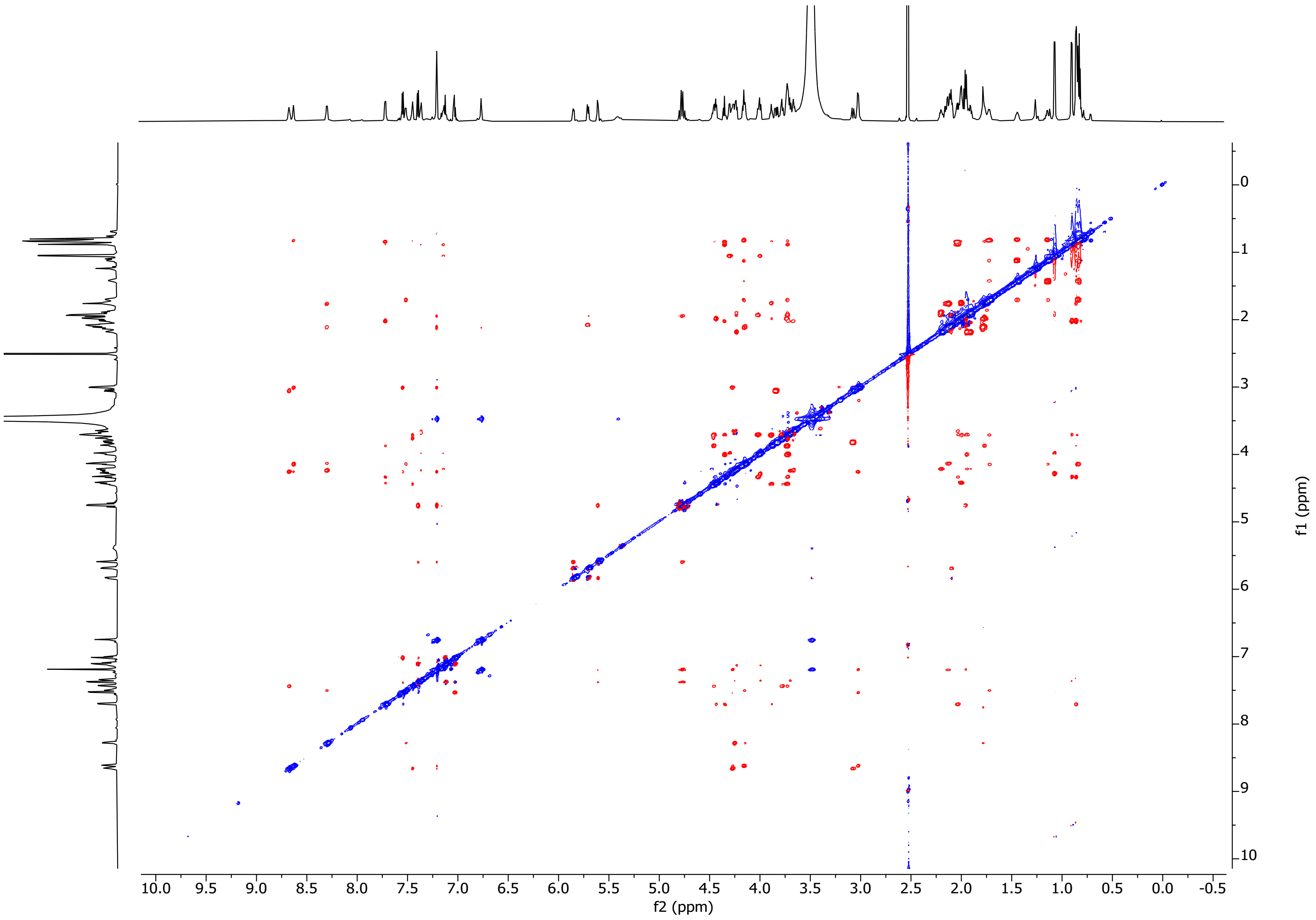

Figure S54 ROESY NMR for **26/27** in DMSO-d_6.

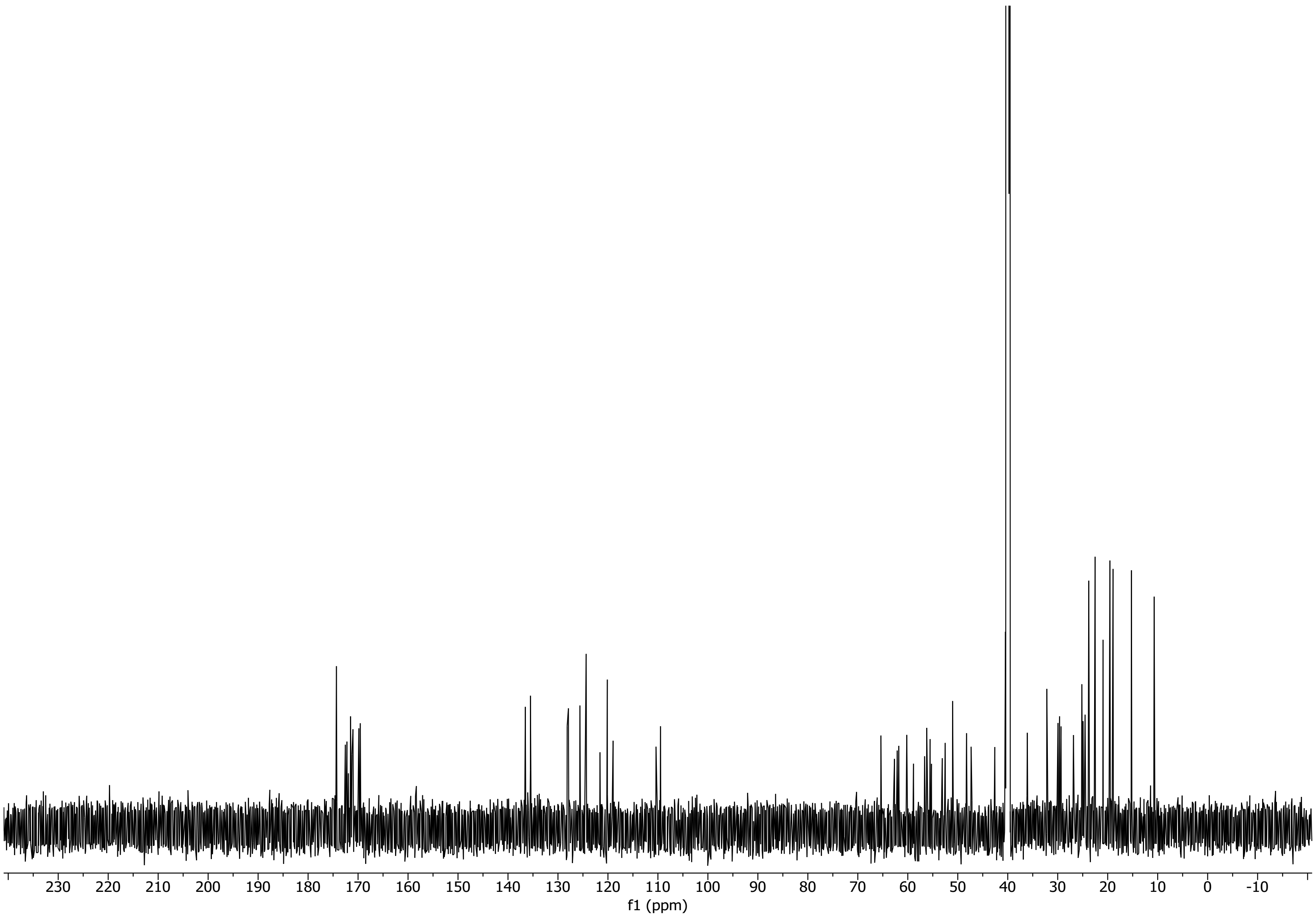

Figure S55 ^{13}C NMR (201 MHz) for **26/27** in DMSO-d_6.

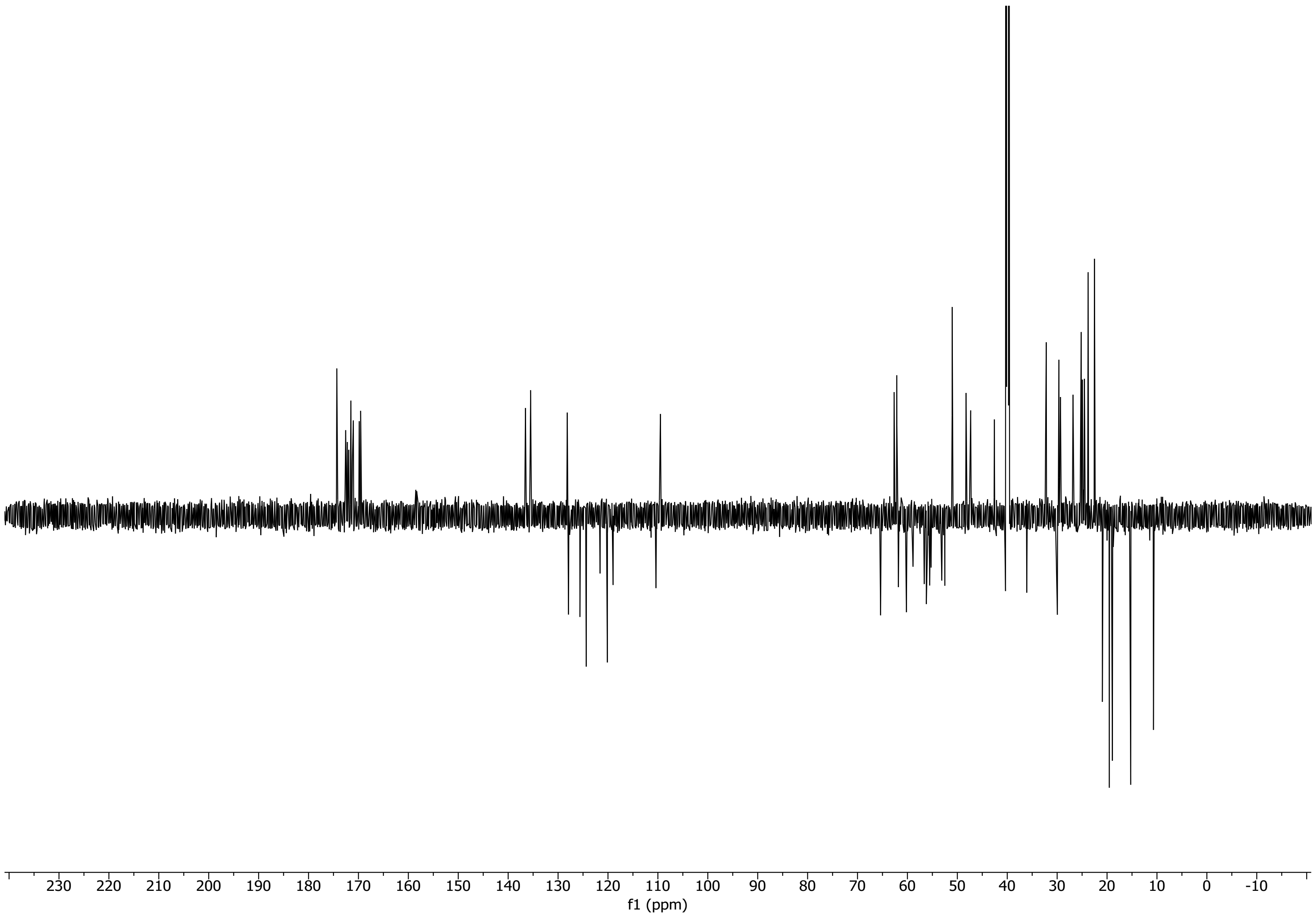

Figure S56 ^{13}C DEPT NMR for **26/27** in DMSO-d_6.

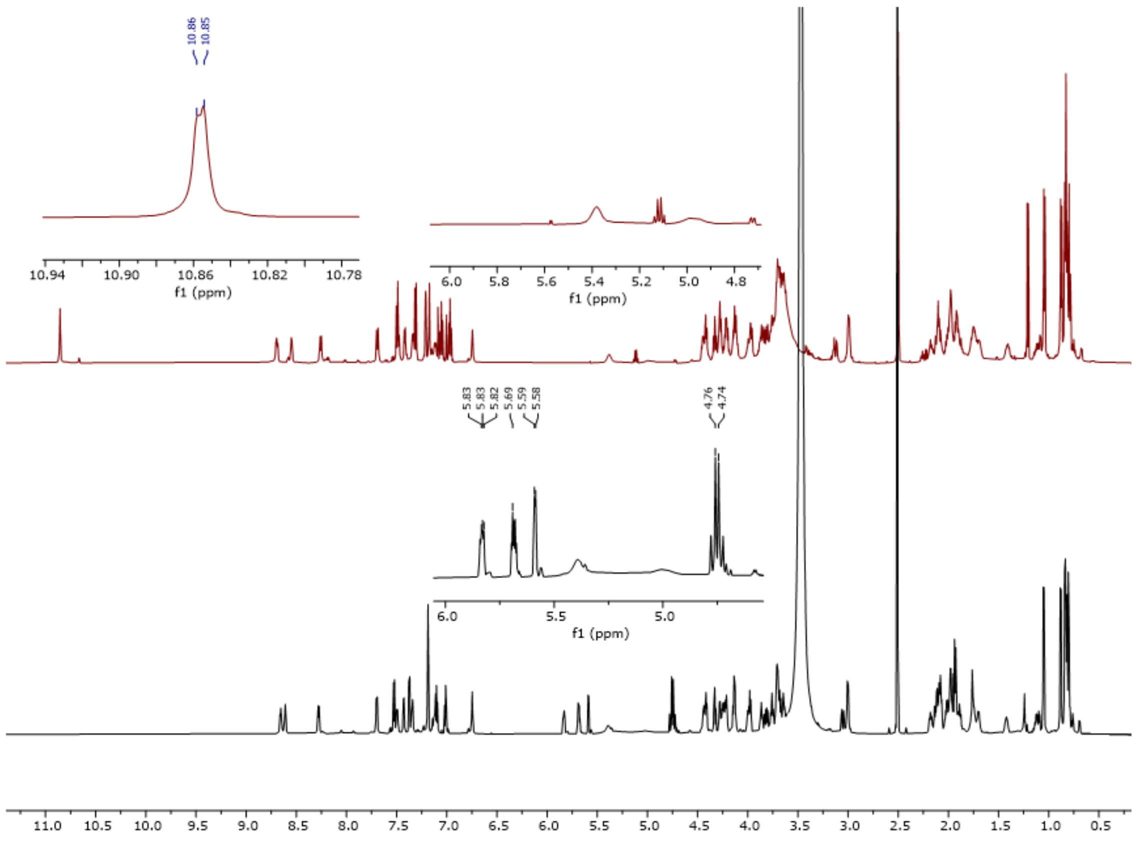

Figure S57 Comparison of ^{1}H NMR for **24** (top) and **26/27** (bottom) in DMSO-d_6. **26** does not display tryptophan NH resonance (10.8 ppm) and shows **4** additional signals between 4.6 and 6 ppm.

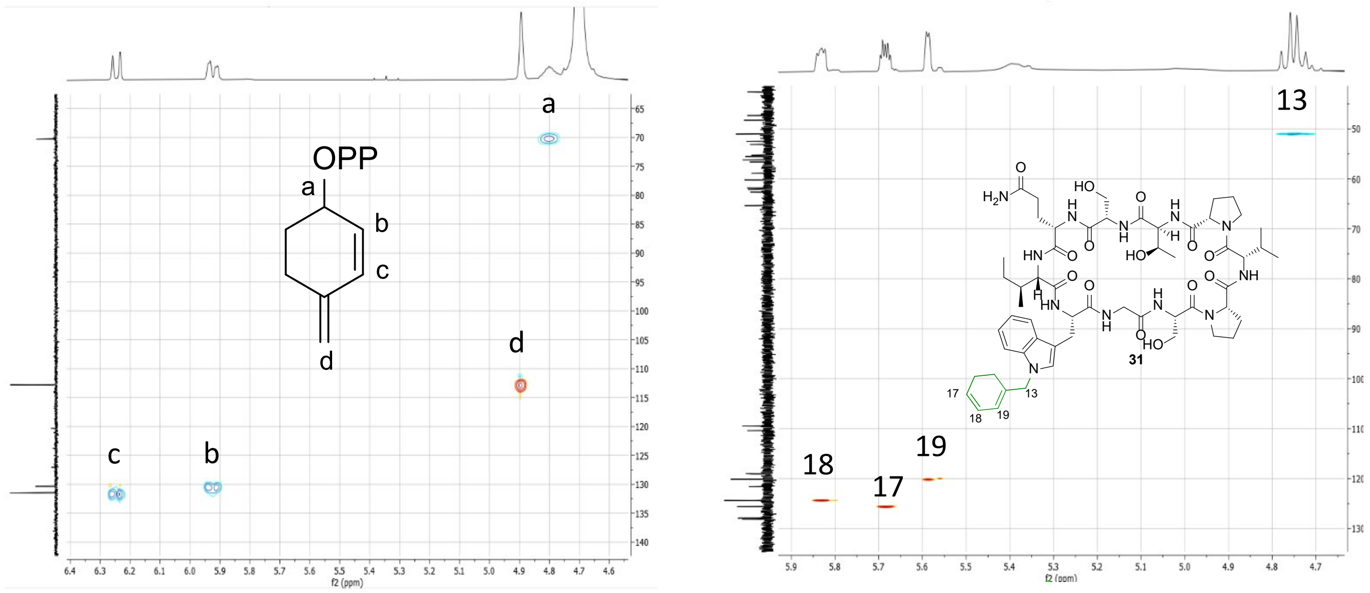

Figure S58 Comparison of the ^{1}H-^{13}C HSQC spectra of **20** (left, D_2O) and **26/27** (right, DMSO-d_6).

Cyclo-[TSQIW(alkyl)GSPVP]			
	Type	^{13}C δ	^{1}H δ, mult, *J* (Hz)
**Tryptophan**			
1	NH	-	8.610, br s
2	CH	55.53	4.250, m
3	CH2	26.82	3.000, 7.5
4	C	109.49	-
5	CH	127.91	7.190, br s
6	C	136.53	-
7	CH	110.37	7.370, d, 8.3
8	CH	121.57	7.100, dd, 7.8, 7.7
9	CH	118.97	7.010, dd, 7.5, 7.4
10	CH	119.08	7.530, d, 7.9
11	C	128.16	-
12	C	172.55	-
**Alkyl group**			
13	CH2	51.05	4.770, d, 16.2
13’	CH2	51.05	4.730, d, 16.2
14	C	135.5	-
15	CH2	23.79	1.936, m
16	CH2	22.52	2.080, m
17	CH	125.61	5.680, dt, 9.5, 4.2
18	CH	124.36	5.830, dd, 9.6, 5.0
19	CH	120.14	5.590, d, 5.0
**Glycine**			
20	NH	-	8.660, m
21	CH2	42.58	3.810, dd, 17.0, 7.8
21’	CH2	42.58	3.050, dd, 17.0, 4.2
22	C	169.55	-
**Serine**			
23	NH	-	7.430, d, 5.1
24	CH	52.5	4.439, m
25	CH2	62.67	3.760, m
25’	CH2	62.67	3.705, m
26	C	171.01	
**Proline**			
27	CH	60.2	4.417, m
28	CH2	29.34	1.980, m
29	CH2	24.53	1.970, m
29’	CH2	24.53	1.747, m
30	CH2	47.32	3.860, m
30’	CH2	47.32	3.705, m
31	C	171.5037	-
**Valine**			
32	NH	-	7.700, d, 8.7
33	CH	56.21	4.330, dd, 8.7, 8.6
34	CH	29.95	2.010, m

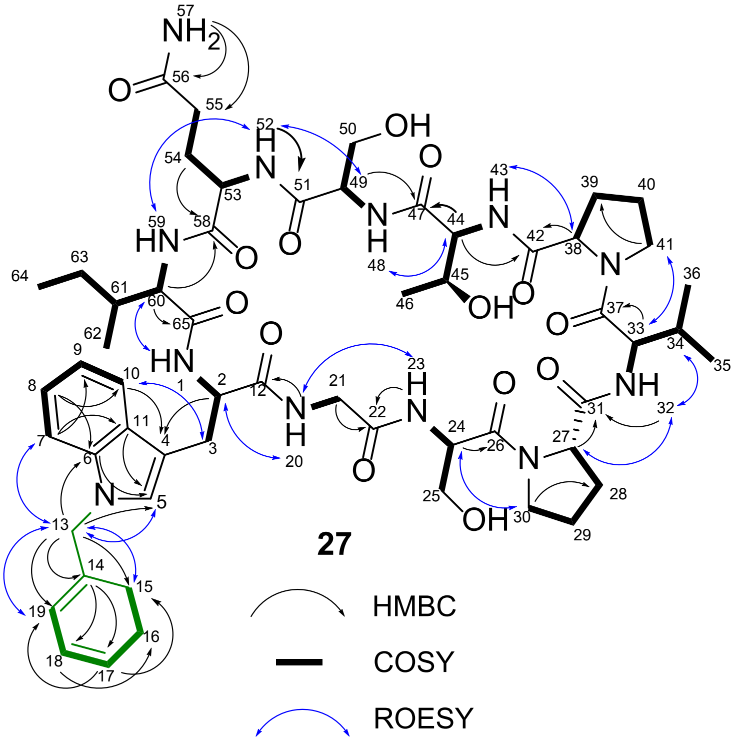

35	CH3	19.55	0.880, d, 6.6
36	CH3	18.93	0.830, m
37	C	172.22	-
**Proline**			
38	CH	61.8	4.210, m
39	CH2	29.63	2.170, m
39'	CH2	29.62	1.890, m
40	CH2	25.17	1.920, m
41	CH2	48.25	3.990, m
41'	CH2	48.25	3.703, m
42	C	171.94	-
**Threonine**			
43	NH	-	7.130, m
44	CH	58.87	3.970, m
45	CH	65.37	4.280, m
46	CH3	20.93	1.050, d, 6.3
47	C	169.85	-
**Serine**			
48	NH	-	7.340, d, 6.3
49	CH	55.27	4.230, m
50	CH2	62.12	3.670, m
50’	CH2	62.12	3.640, m
51	C	171.09	-
**Glutamine**			
52	NH	-	8.280, d, 8.0
53	CH	53.11	4.127, m
54	CH2	26.85	2.100, m
54’	CH2	26.85	1.756, m
55	CH2	32.18	2.113, m
56	C	174.330	-
57	NH	-	7.190, br s
57’	NH	-	6.740, br s
58	C	171.21	-
**Isoleucine**			
59	NH	-	7.490, d, 8.0
60	CH	56.63	4.137, m
61	CH	36.09	1.700, m
62	CH3	15.25	0.820, m
63	CH2	24.94	1.42, m
63’	CH2	24.94	1.110, m
64	CH3	10.7	0.800, m
65	C	172.44	-

Table S3 NMR data for **27** in DMSO-d_6

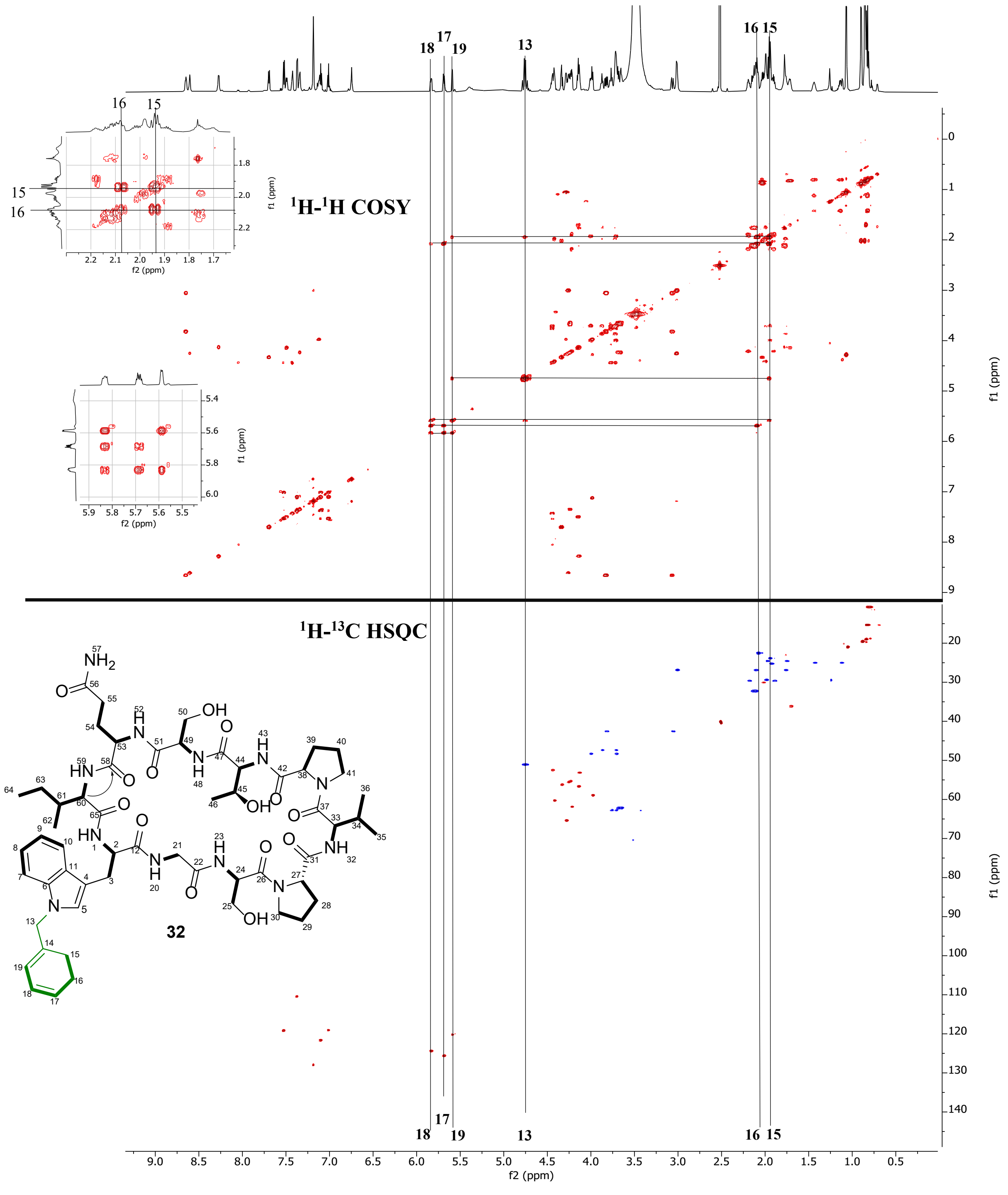

Figure S59 Partially assigned ^{1}H-^{1}H COSY and ^{1}H-^{13}C HSQC NMR for isolated **27**.

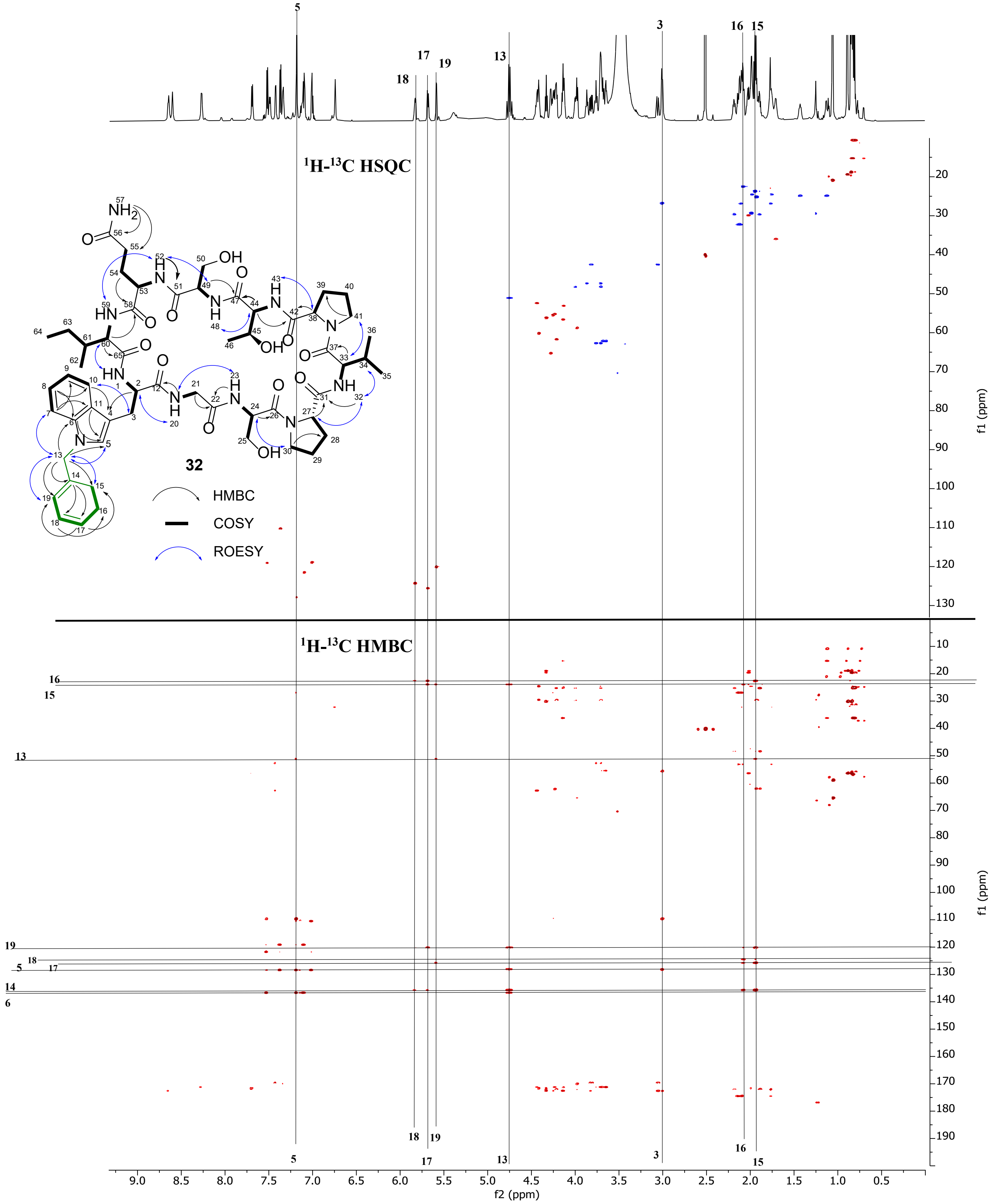

Figure S60 Partially assigned ^{1}H-^{13}C HSQC and ^{1}H-^{13}C HMBC NMR for isolated isolated **27**.

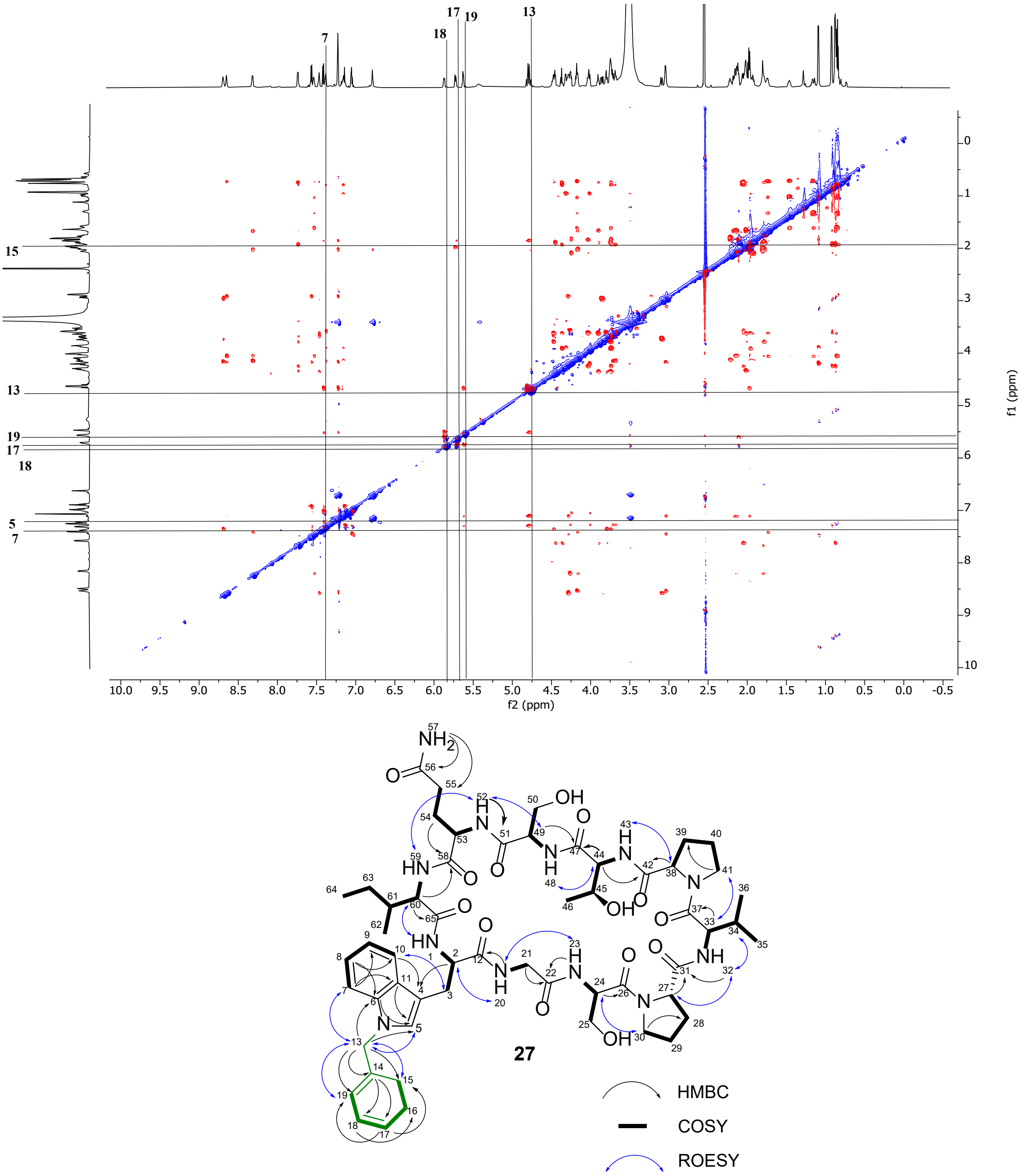

Figure S61 Partially assigned ROESY NMR for isolated isolated **27**.

2.6.2 HRMS and MS/MS

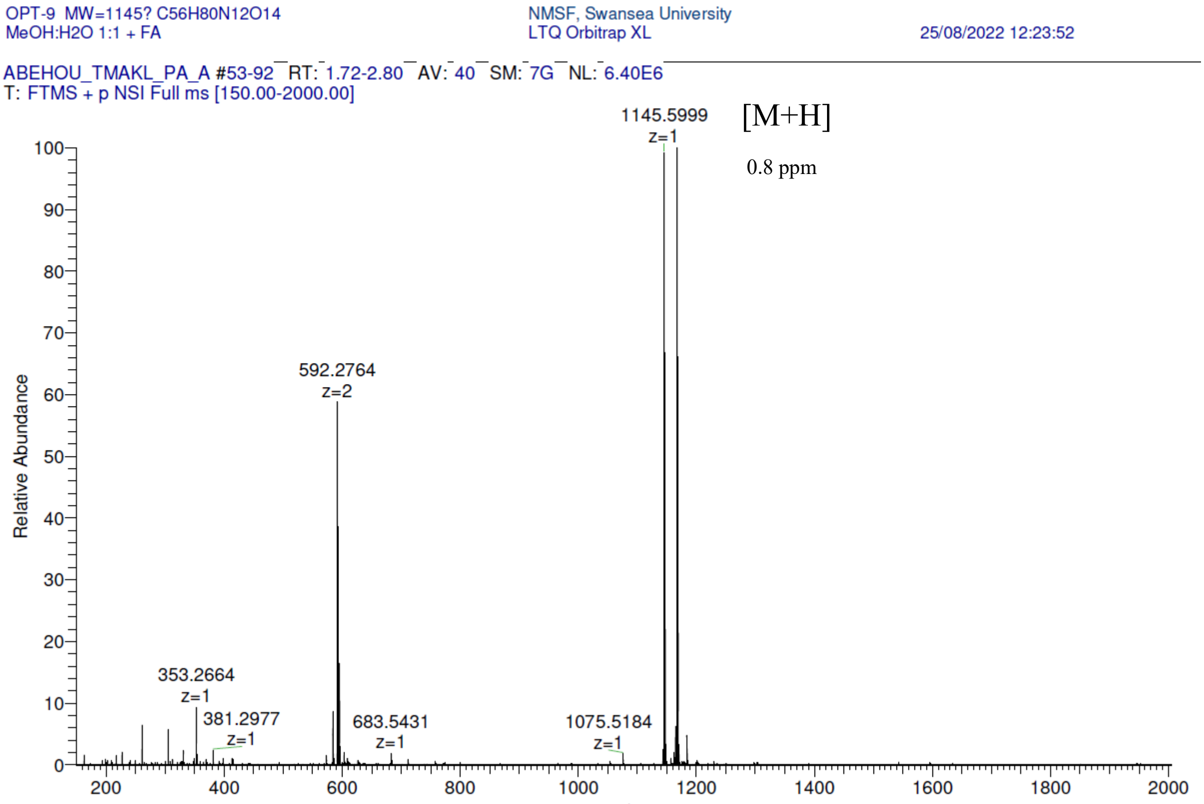

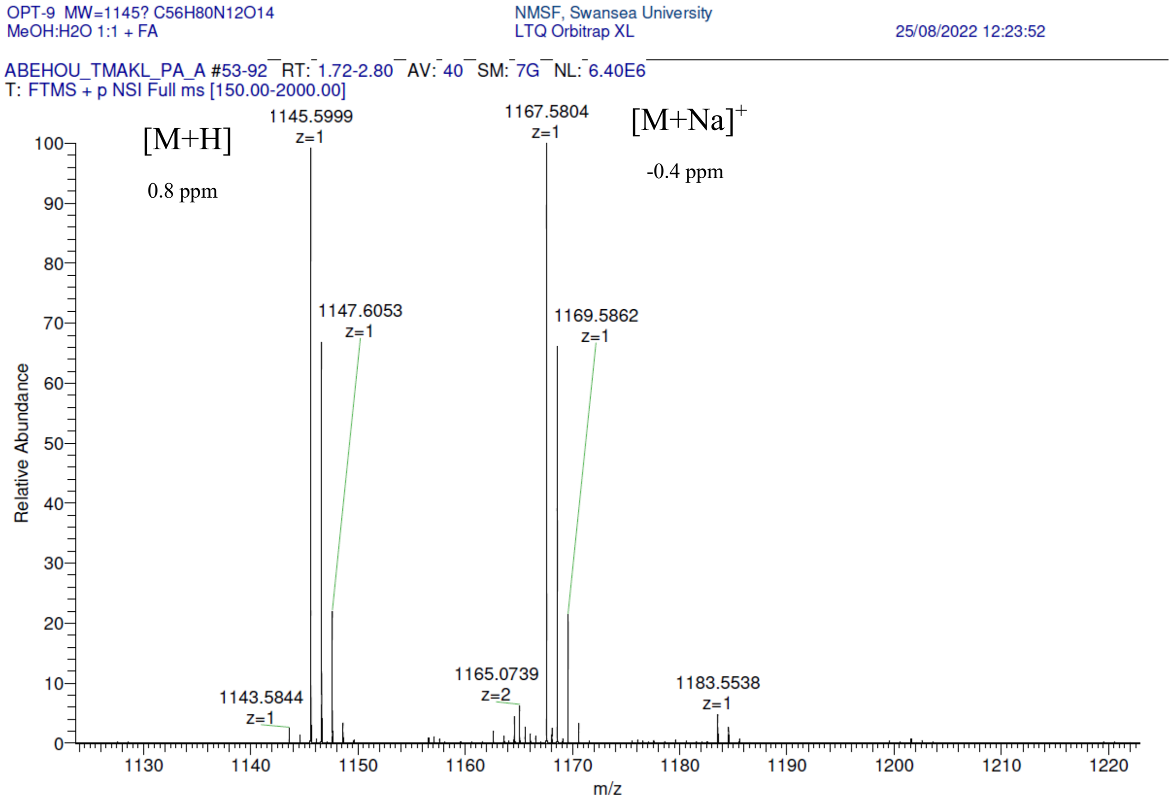

Figure S62 HRMS of **27**.

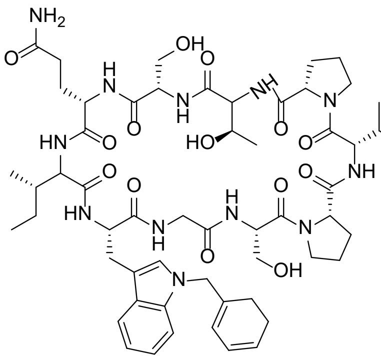

Chemical Formula: $C_{56}H_{80}N_{12}O_{14}$
Exact Mass: 1144.5917

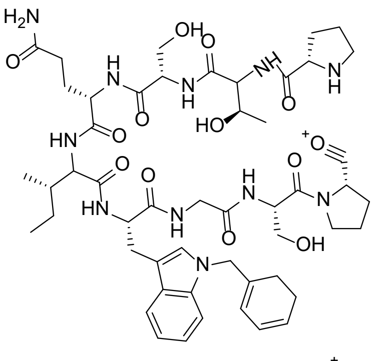

Chemical Formula: $C_{51}H_{72}N_{11}O_{13}^{+}$
Exact Mass: 1046.5306
Δ −1.1 ppm

***b*9, loss of Valine**

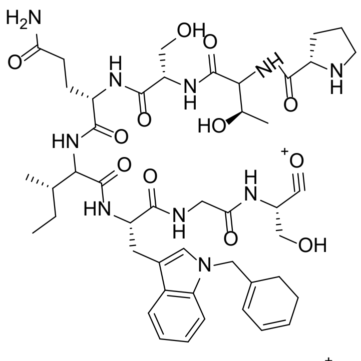

Chemical Formula: $C_{46}H_{65}N_{10}O_{12}^{+}$
Exact Mass: 949.4778
Δ −1.2 ppm

***b*8, loss of valine and proline**

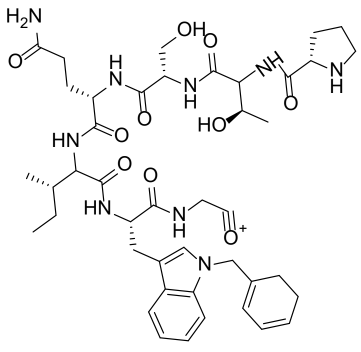

Chemical Formula: $C_{43}H_{60}N_{9}O_{10}^{+}$
Exact Mass: 862.4458
Δ −1.4 ppm

***b*7, loss of valine, proline, serine**

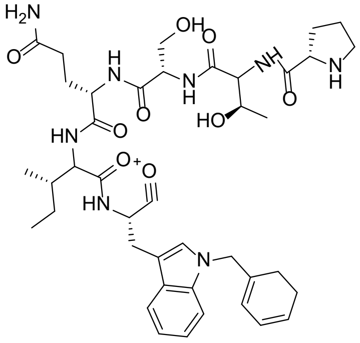

Chemical Formula: $C_{41}H_{57}N_{8}O_{9}^{+}$
Exact Mass: 805.4243
Δ −1.4 ppm

***b*6, loss of valine, proline, serine, glycine**

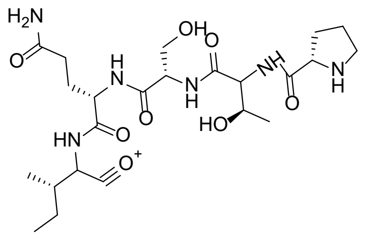

Chemical Formula: $C_{23}H_{39}N_{6}O_{8}^{+}$
Exact Mass: 527.2824
Δ −1.9 ppm

***b*5, loss of valine, proline, serine, glycine, alkylated triptophan**

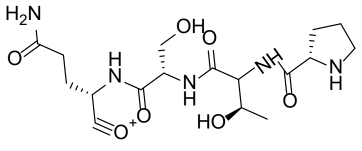

Chemical Formula: $C_{17}H_{28}N_{5}O_{7}^{+}$
Exact Mass: 414.1983
Δ −1.2 ppm

***b*4, loss of valine, proline, serine, glycine, alkylated triptophan, isoleucine**

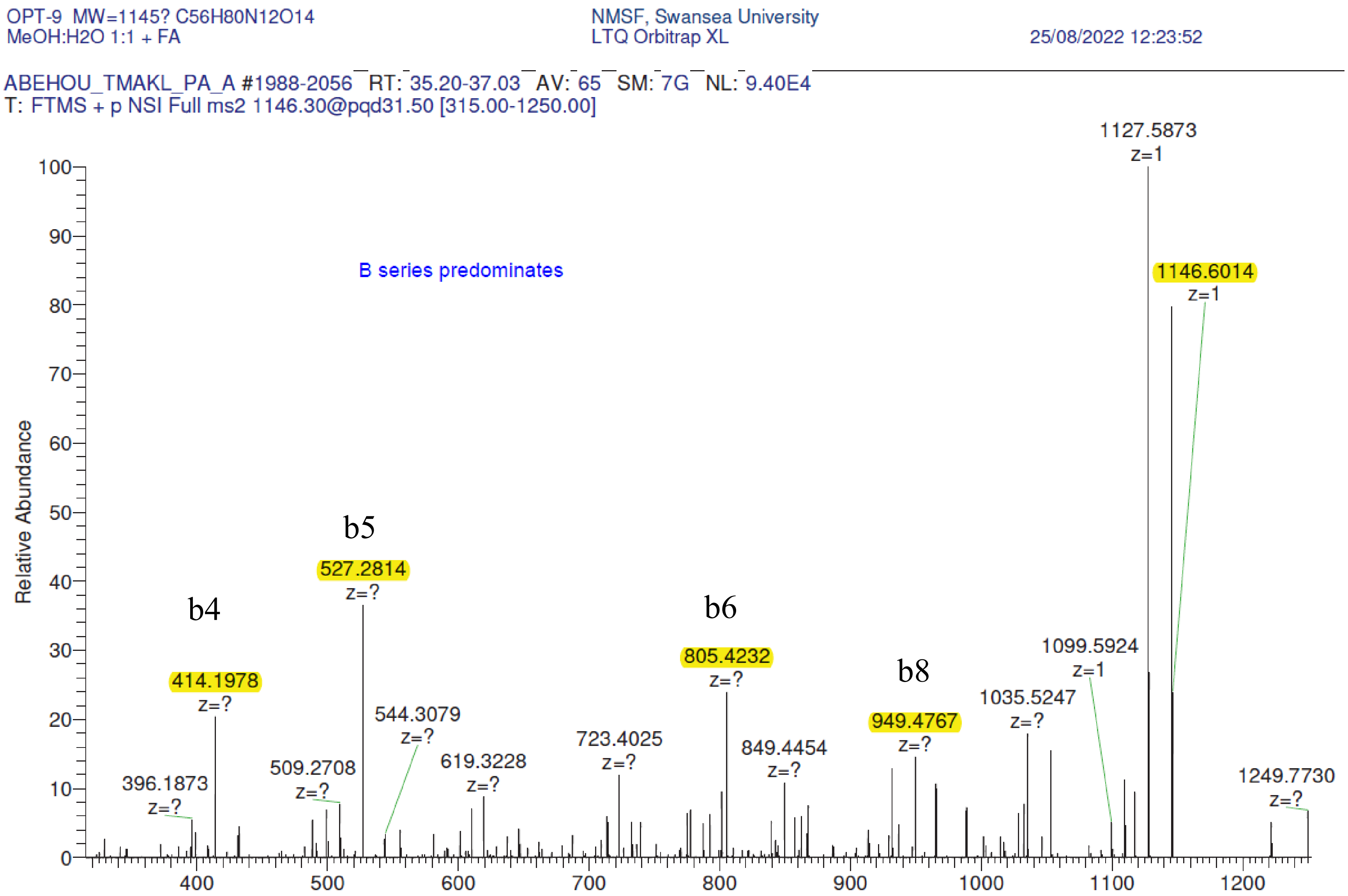
OPT-9 MW=1145? C56H80N12O14
MeOH:H2O 1:1 + FA
NMSF, Swansea University
LTQ Orbitrap XL
25/08/2022 12:23:52
ABEHOU_TMAKL_PA_A #1988-2056 RT: 35.20-37.03 AV: 65 SM: 7G NL: 9.40E4
T: FTMS + p NSI Full ms2 1146.30@pqd31.50 [315.00-1250.00]
B series predominates
Relative Abundance
1127.5873 z=1
1146.6014 z=1
b5
527.2814 z=?
b4
414.1978 z=?
b6
805.4232 z=?
b8
949.4767 z=?
1099.5924 z=1
1035.5247 z=?
544.3079 z=?
396.1873 z=?
509.2708 z=?
619.3228 z=?
723.4025 z=?
849.4454 z=?
1249.7730 z=?

OPT-9 MW=1145? C56H80N12O14
MeOH:H2O 1:1 + FA

NMSF, Swansea University
LTQ Orbitrap XL

25/08/2022 12:23:52

ABEHOU_TMAKL_PA_A #1988-2056 RT: 35.20-37.03 AV: 65 SM: 7G NL: 1.68E4
T: FTMS + p NSI Full ms2 1146.30@pqd31.50 [315.00-1250.00]

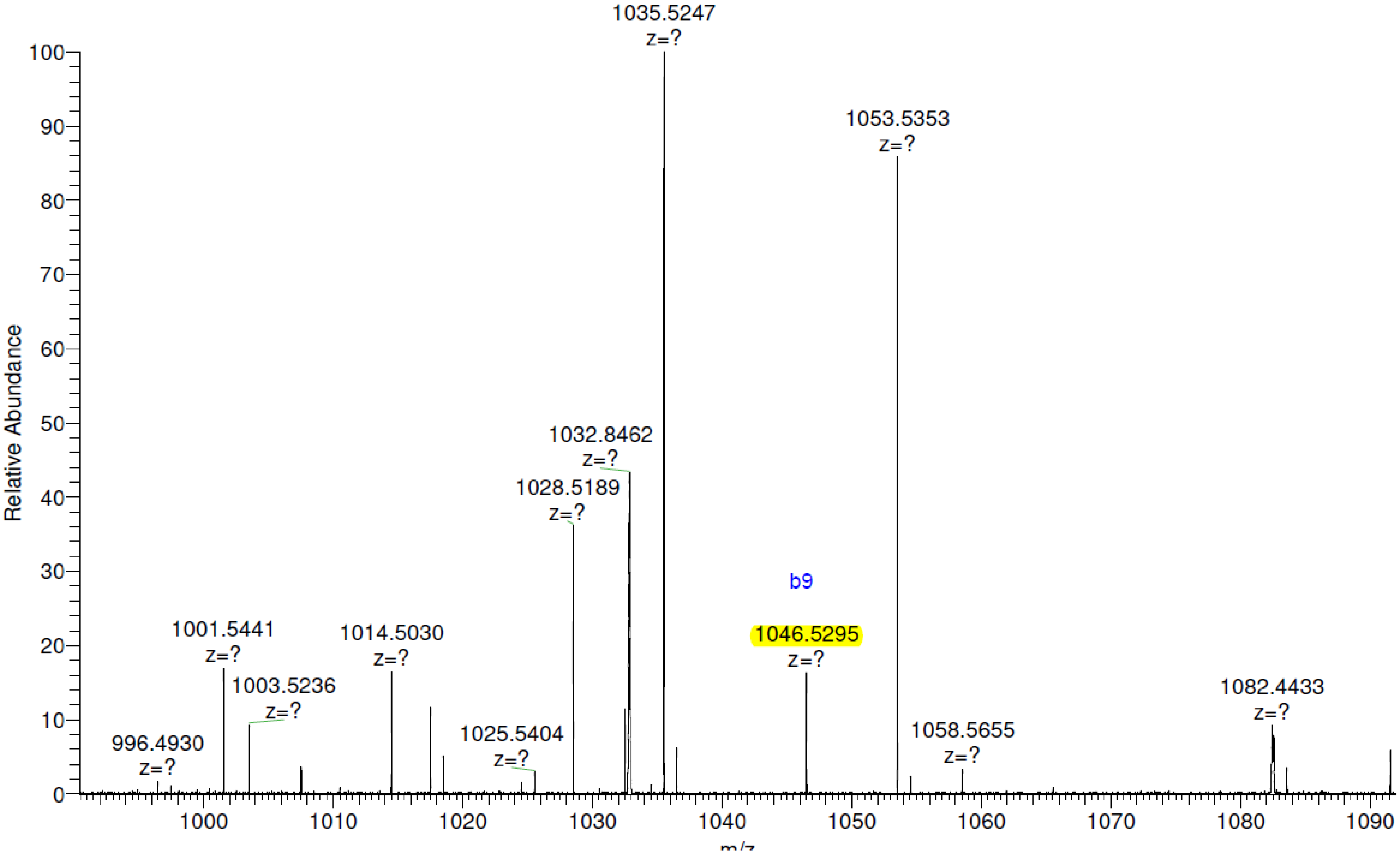

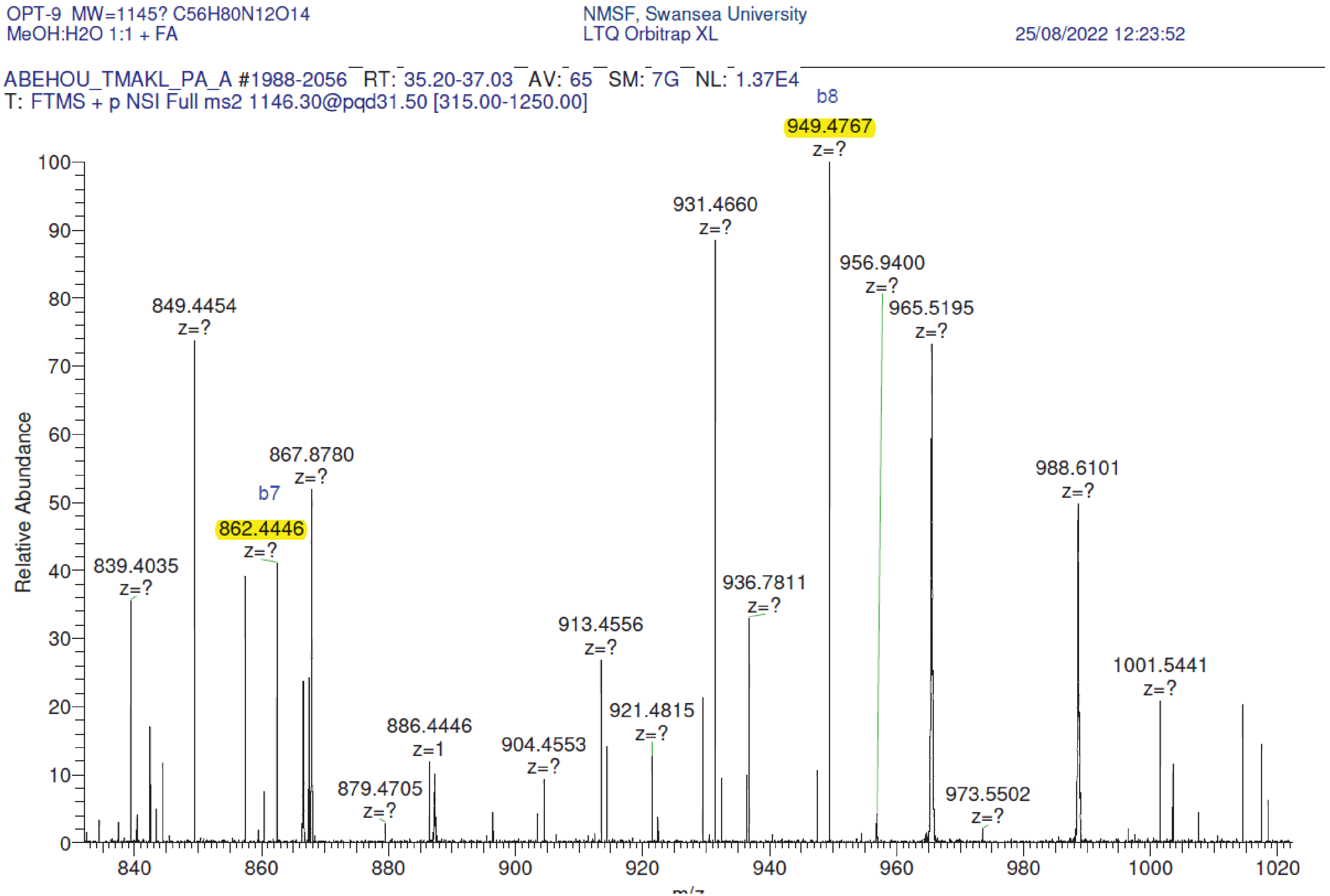

OPT-9 MW=1145? C56H80N12O14
MeOH:H2O 1:1 + FA
NMSF, Swansea University
LTQ Orbitrap XL
25/08/2022 12:23:52
ABEHOU_TMAKL_PA_A #1988-2056 RT: 35.20-37.03 AV: 65 SM: 7G NL: 1.37E4
T: FTMS + p NSI Full ms2 1146.30@pqd31.50 [315.00-1250.00]
b8
949.4767
z=?
931.4660
z=?
956.9400
z=?
965.5195
z=?
849.4454
z=?
867.8780
z=?
b7
862.4446
z=?
839.4035
z=?
988.6101
z=?
936.7811
z=?
913.4556
z=?
1001.5441
z=?
921.4815
z=?
886.4446
z=1
904.4553
z=?
879.4705
z=?
973.5502
z=?
Relative Abundance
m/z

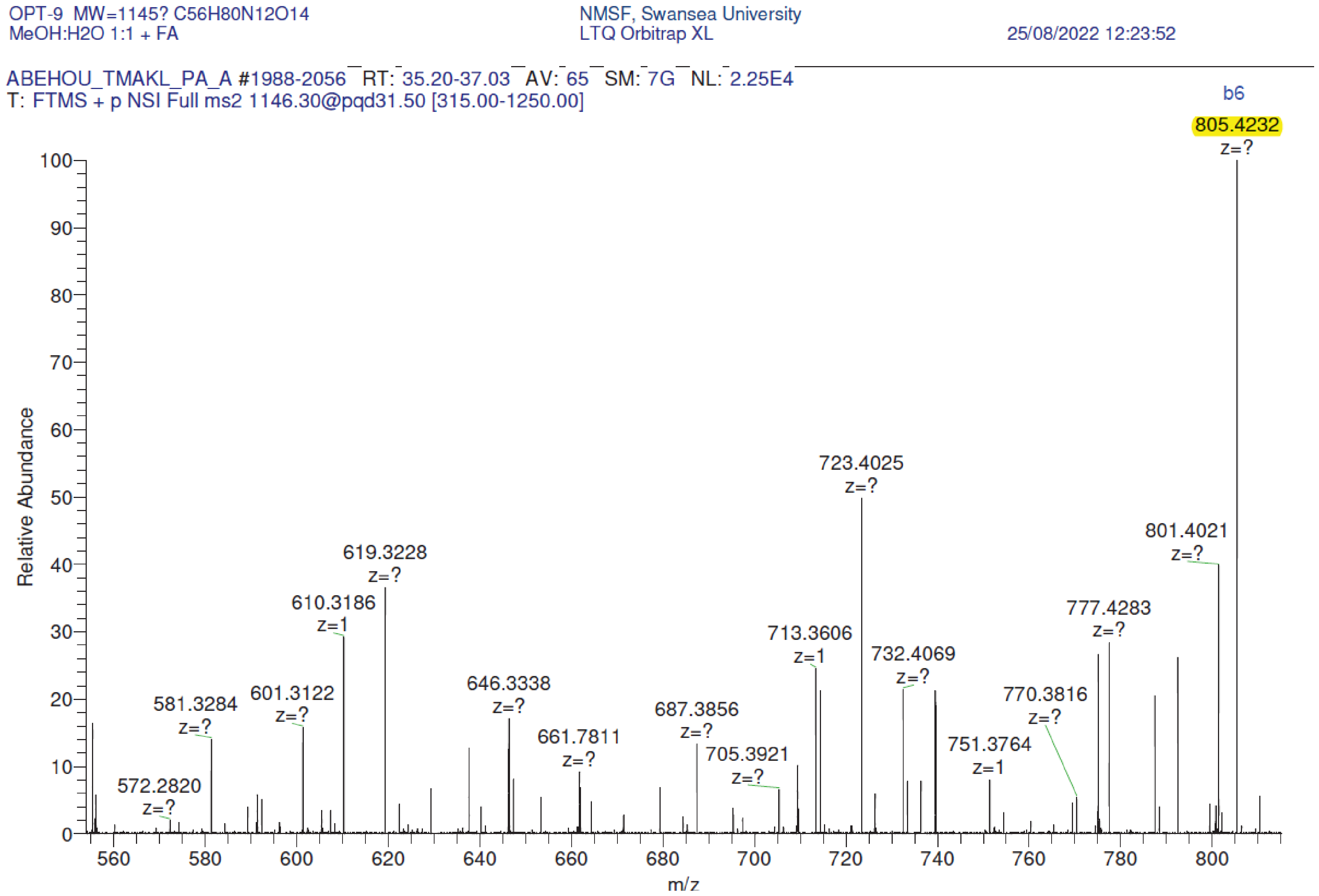

OPT-9 MW=1145? C56H80N12O14
MeOH:H2O 1:1 + FA
NMSF, Swansea University
LTQ Orbitrap XL
25/08/2022 12:23:52
ABEHOU_TMAKL_PA_A #1988-2056 RT: 35.20-37.03 AV: 65 SM: 7G NL: 2.25E4
T: FTMS + p NSI Full ms2 1146.30@pqd31.50 [315.00-1250.00]
b6
805.4232 z=?
723.4025 z=?
801.4021 z=?
619.3228 z=?
610.3186 z=1
777.4283 z=?
713.3606 z=1
732.4069 z=?
646.3338 z=?
770.3816 z=?
601.3122 z=?
581.3284 z=?
687.3856 z=?
751.3764 z=1
661.7811 z=?
705.3921 z=?
572.2820 z=?
Relative Abundance
m/z

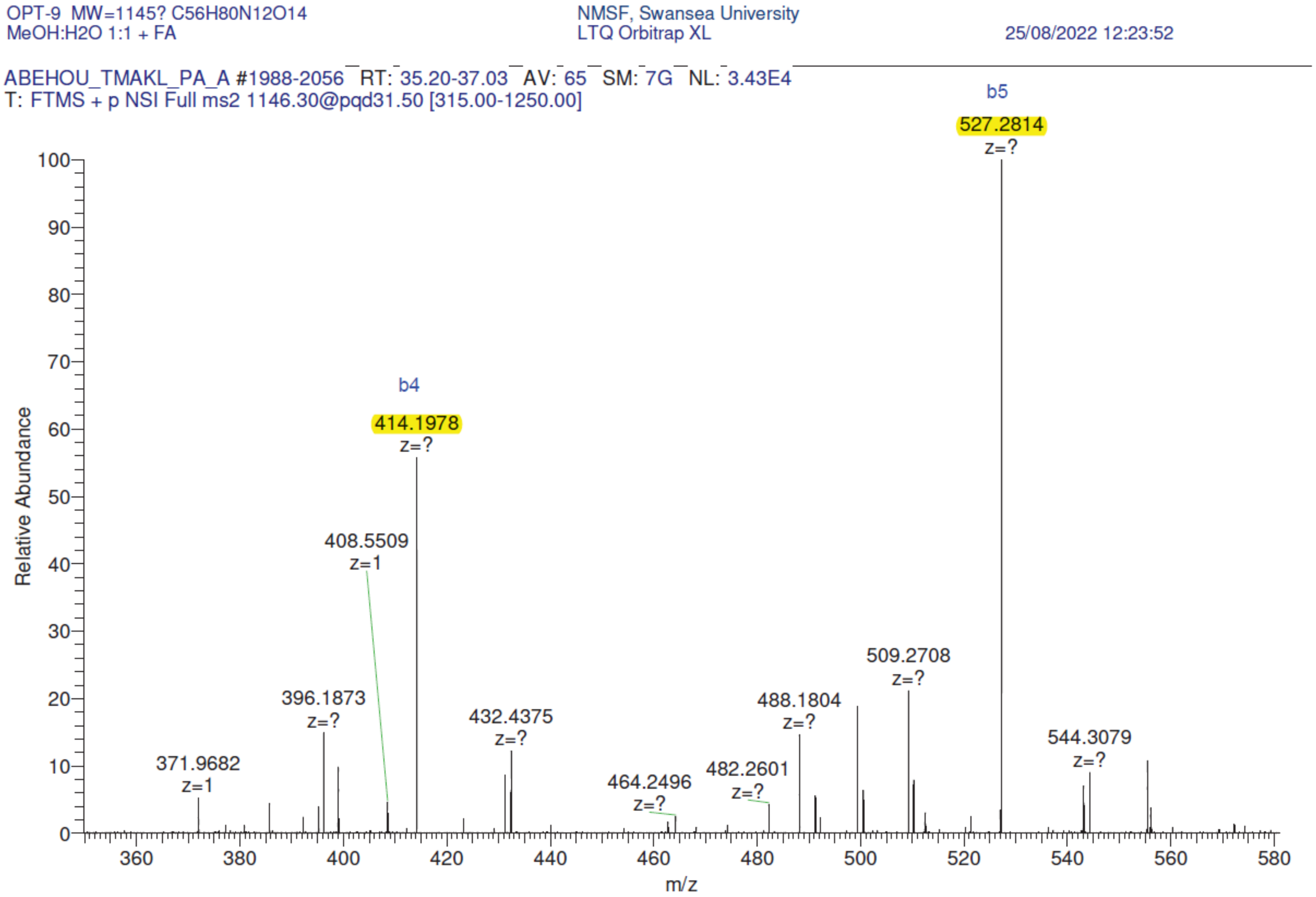

Figure S63 Pulsed-Q dissociation MS/MS for isolated **27**. Predominant species observed were *b*-ions. Ions *b*6-*b*5 are consistent with loss of alkylated tryptophan.

2.7 Labelling of 27 with pyrimidyl-tetrazine-5-FAM (28)

Modified peptide **27** (0.75 mg, 0.65 µmol, 1 eq.) and *N*-(3-aminopropyl)-4-(6-(pyrimidin-2-yl)-1,2,4,5-tetrazin-3-yl)benzamid-5-FAM (pyrimidyl-tetrazine-5-FAM, Jena Bioscience) (**28**) (0.5 mg, 0.72 µmol, 1.1 eq.) were dissolved in a mixture of 100 µL of water and 50 µL of DMSO and the resulting solution was stirred overnight at room temperature in the absence of light. The reaction was analysed by LC-HRMS and showed formation of clicked products (dihydropyridazine-type and pyridazine-type products, Figure S27).

A control reaction was setup in parallel in an identical manner, but with unmodified peptide **24** instead of **27** (Figure S28).

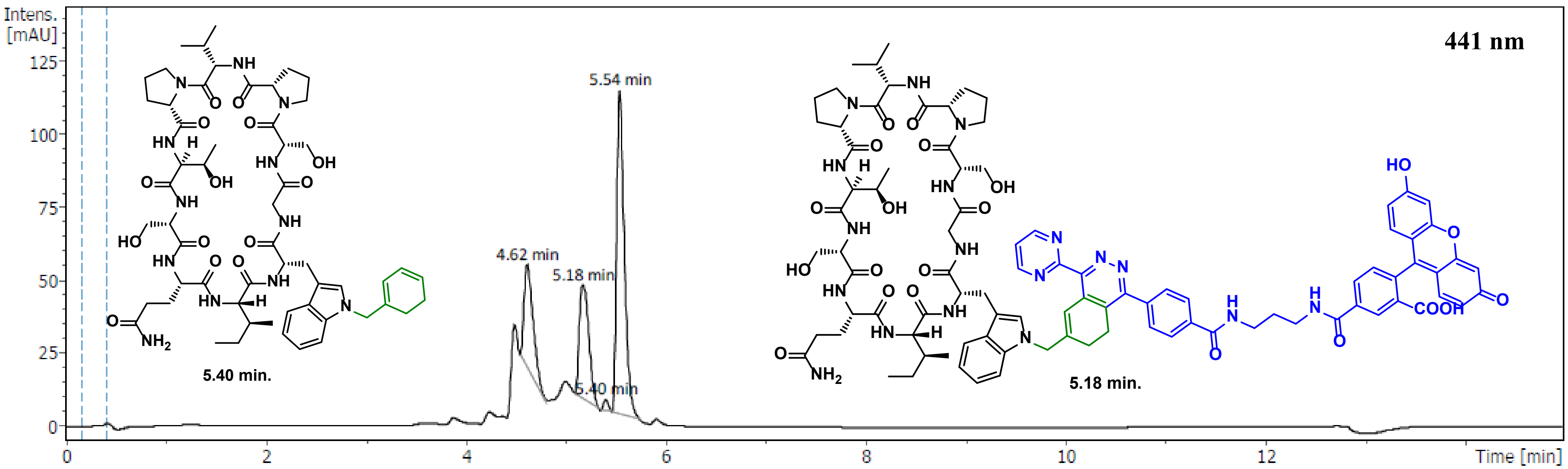
Intens. [mAU]
441 nm
5.54 min
4.62 min
5.18 min
5.40 min
5.40 min.
5.18 min.
Time [min]

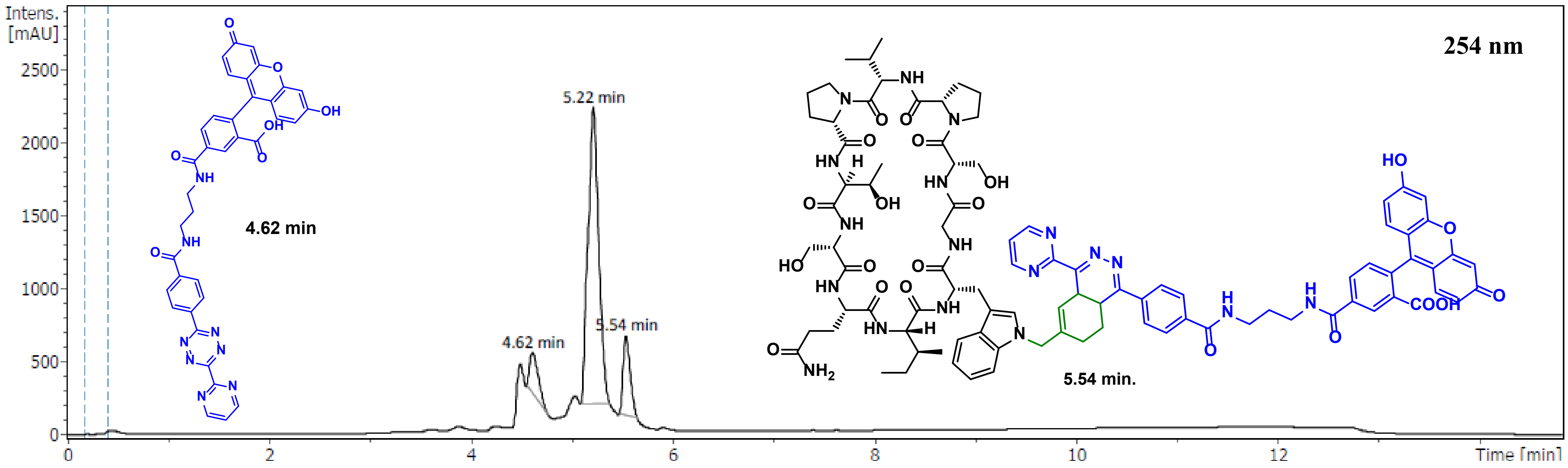
Intens. [mAU]
254 nm
5.22 min
4.62 min
5.54 min
4.62 min
5.54 min.
Time [min]

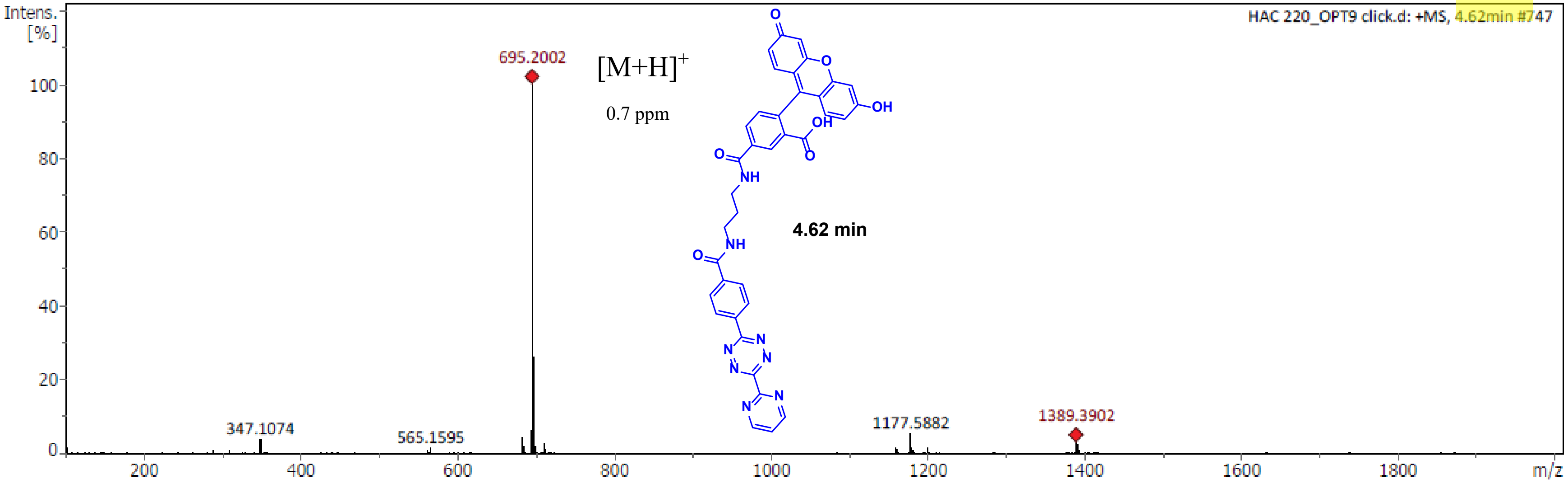
HAC 220_OPT9 click.d: +MS, 4.62min #747
Intens. [%]
695.2002
[M+H]+
0.7 ppm
4.62 min
347.1074
565.1595
1177.5882
1389.3902
m/z

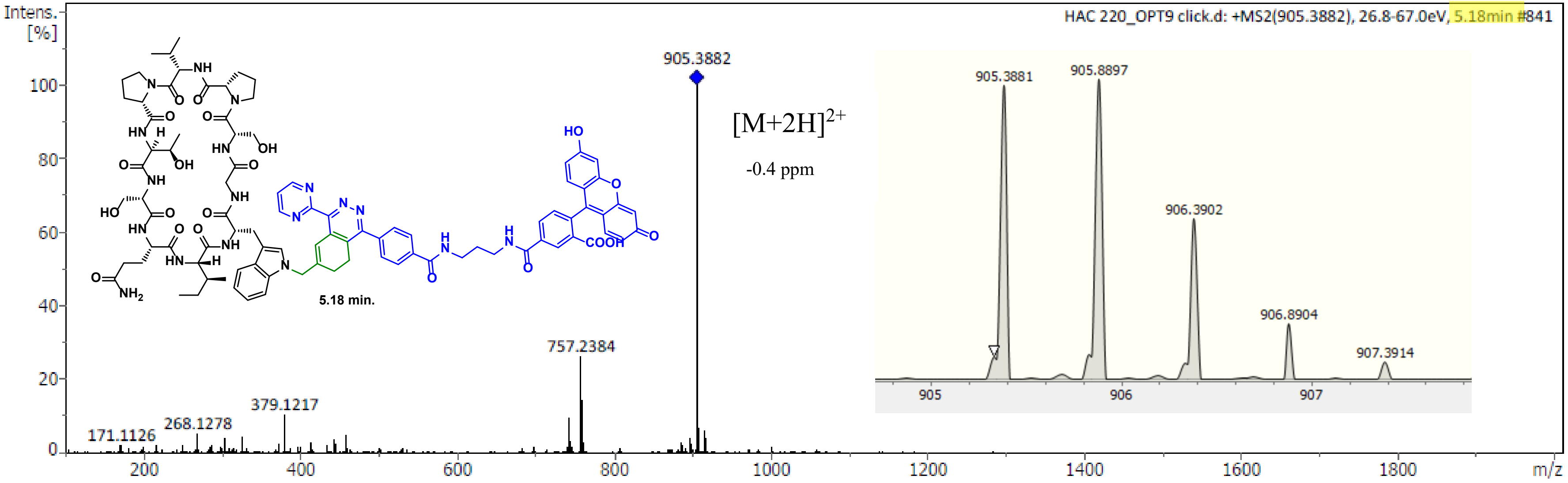
HAC 220_OPT9 click.d: +MS2(905.3882), 26.8-67.0eV, 5.18min #841
Intens. [%]
905.3882
[M+2H]2+
-0.4 ppm
5.18 min.
757.2384
379.1217
268.1278
171.1126
905.3881
905.8897
906.3902
906.8904
907.3914
m/z

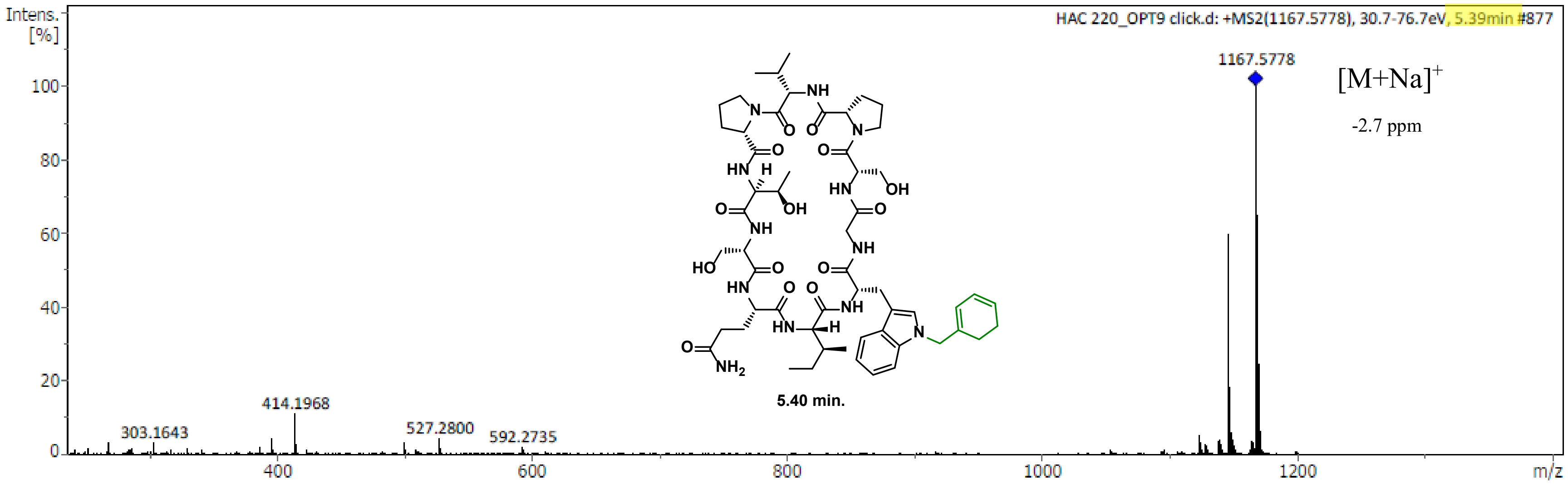

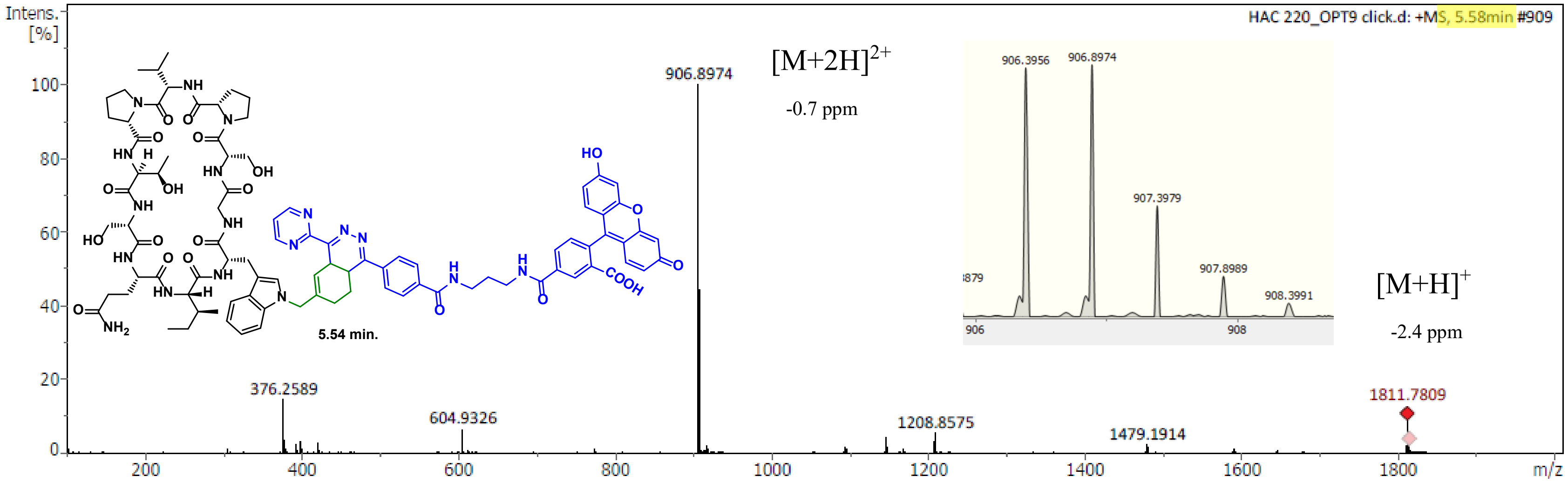

Figure S64 LC-HRMS (441/254 nm) for click labelling of **27** (5.40 min) with fluorescent tetrazine **28** (4.62 min). Dihydropyridazine-type (5.54 min) and pyridazine-type (5.18 min) products were detected.

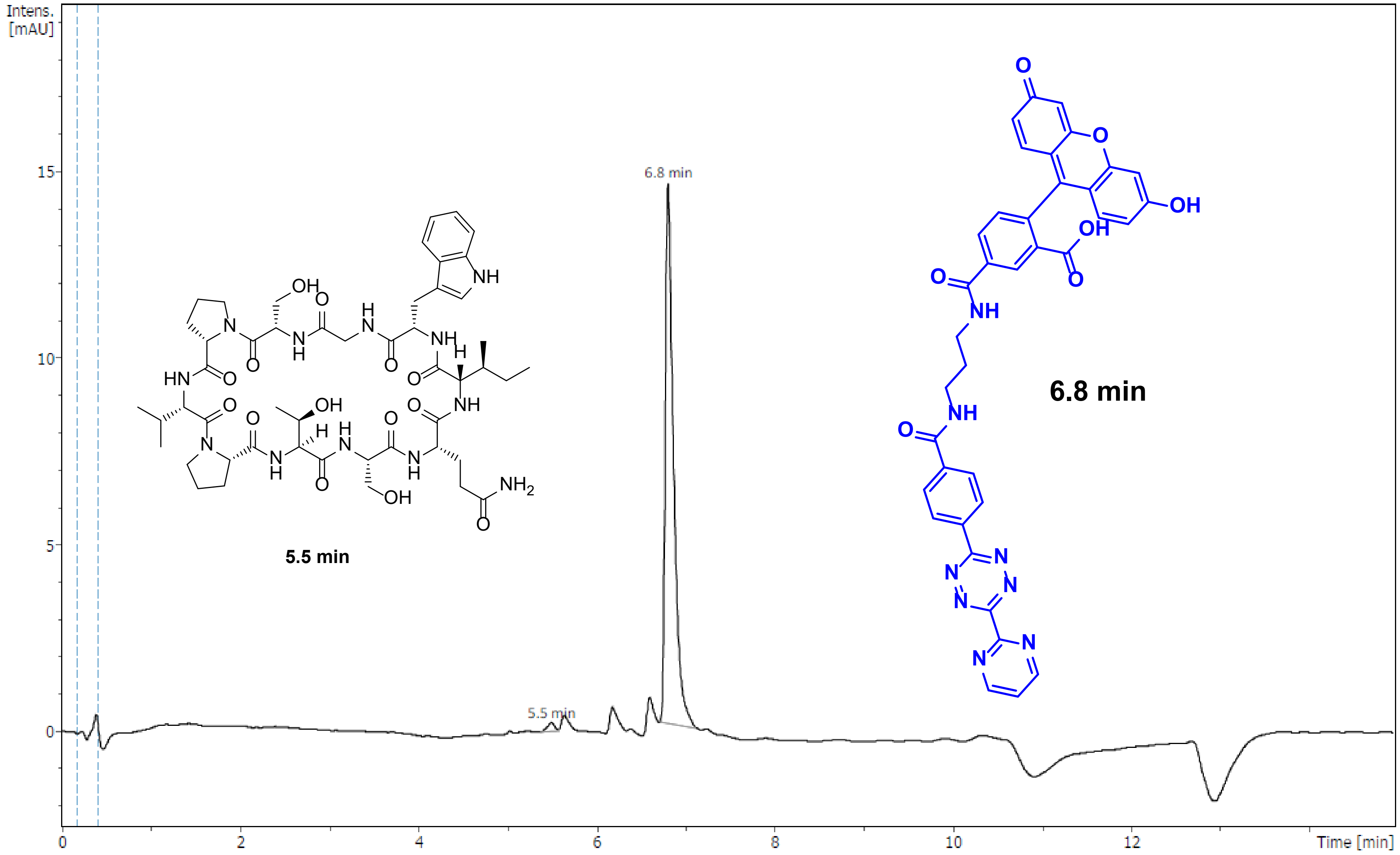

Intens.
[mAU]
15
10
5
0
0
2
4
6
8
10
12
Time [min]
6.8 min
5.5 min
5.5 min
6.8 min

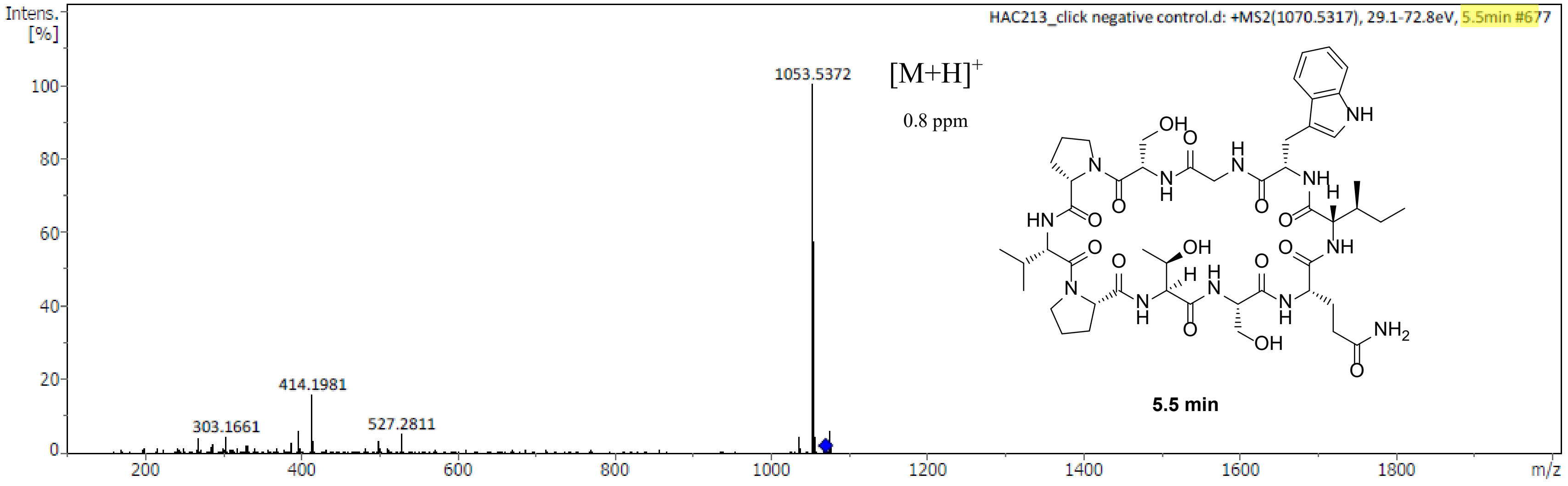

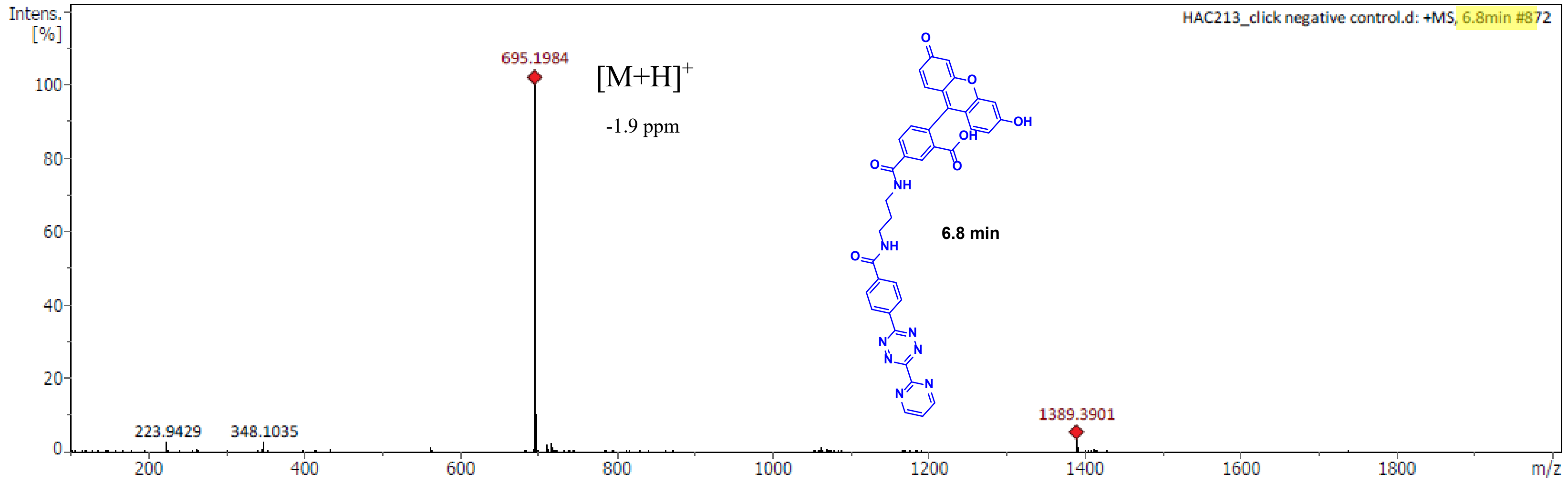

Figure S65 LC-HRMS (441 nm) for control labelling between unmodified peptide **24** (5.5 min.) and tetrazine **28** (6.8 min). As expected, no clicked products were observed.

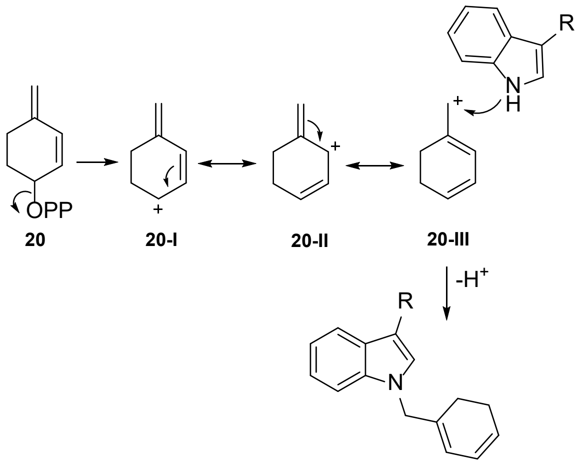

Figure S66 Proposed formation mechanism for 27. The carbocation generated from 20 can exist in 3 different resonance structures (20-I, 20-II and 20-III). Indole nitrogen nucleophilic attack to resonance structure 20-III structure could lead to isolated compound 27.

3 References

[1] K. Wang, W. Wang, J.-H. No, Y. Zhang, Y. Zhang, E. Oldfield, *J. Am. Chem. Soc.* **2010**, *132*, 6719–6727.

[2] V. J. Davisson, A. B. Woodside, T. R. Neal, K. E. Stremler, M. Muehlbacher, C. D. Poulter, *J. Org. Chem.* **1986**, *51*, 4768–4779.

[3] D. J. Phillips, K. S. Pillinger, W. Li, A. E. Taylor, A. E. Graham, *Tetrahedron* **2007**, *63*, 10528–10533.

[4] M. Liebhold, X. Xie, S.-M. Li, *Org. Lett.* **2012**, *14*, 4882–4885.

[5] X. Wu, H.-J. Wang, Y.-S. Huang, W.-D. Z. Li, *Org. Lett.* **2018**, *20*, 1871–1874.

[6] S. Garrais, J. Turkington, W. P. D. Goldring, *Tetrahedron* **2009**, *65*, 8418–8427.

[7] M. W. Grafton, L. J. Farrugia, A. Sutherland, *J. Org. Chem.* **2013**, *78*, 7199–7207.

[8] S. Essig, B. Schmalzbauer, S. Bretzke, O. Scherer, A. Koeberle, O. Werz, R. Müller, D. Menche, *J. Org. Chem.* **2016**, *81*, 1333–1357.

[9] F. M. Cordero, F. Pisaneschi, M. Gensini, A. Goti, A. Brandi, *Eur. J. Org. Chem.* **2002**, *2002*, 1941–1951.

[10] R. A. Bunce, D. A. Irwin, *Synth. Commun.* **1990**, *20*, 2979–2982.

[11] R. Ding, Z.-D. Huang, Z.-L. Liu, T.-X. Wang, Y.-H. Xu, T.-P. Loh, *Chem. Commun.* **2016**, *52*, 5617–5620.

[12] T. Schlatzer, J. Kriegesmann, H. Schröder, M. Trobe, C. Lembacher-Fadum, S. Santner, A. V. Kravchuk, C. F. W. Becker, R. Breinbauer, *J. Am. Chem. Soc.* **2019**, *141*, 14931–14937.

[13] B. M. K. Tong, H. Chen, S. Y. Chong, Y. L. Heng, S. Chiba, *Org. Lett.* **2012**, *14*, 2826–2829.

[14] C. Fischer, S. W. Smith, D. A. Powell, G. C. Fu, *J. Am. Chem. Soc.* **2006**, *128*, 1472–1473.

[15] A. M. Whittaker, R. P. Rucker, G. Lalic, *Org. Lett.* **2010**, *12*, 3216–3218.

[16] S. A. Kozmin, J. M. Janey, V. H. Rawal, *J. Org. Chem.* **1999**, *64*, 3039–3052.

[17] H. Wild, *J. Org. Chem.* **1994**, *59*, 2748–2761.

[18] S. S. Chaudhari, K. G. Akamanchi, *Synlett* **1999**, *1999*, 1763–1765.

[19] A. Kinbara, T. Yamagishi, N. Hanzawa, E. Kawashima, H. Miyaoka, *J. Org. Chem.* **2012**, *77*, 8999–9005.

[20] J. M. Kelly, F. J. Leeper, *Tetrahedron Lett.* **2012**, *53*, 819–821.

[21] P. Chand, P. L. Kotian, A. Dehghani, Y. El-Kattan, T.-H. Lin, T. L. Hutchison, Y. S. Babu, S. Bantia, A. J. Elliott, J. A. Montgomery, *J. Med. Chem.* **2001**, *44*, 4379–4392.

[22] L. Dalponte, A. Parajuli, E. Younger, A. Mattila, J. Jokela, M. Wahlsten, N. Leikoski, K. Sivonen, S. A. Jarmusch, W. E. Houssen, D. P. Fewer, *Biochemistry* **2018**, *57*, 6860–6867.

[23] H. Bisswanger, *Perspect. Sci.* **2014**, *1*, 41–55.

`

4 NMR spectra of pyrophosphates 2-23

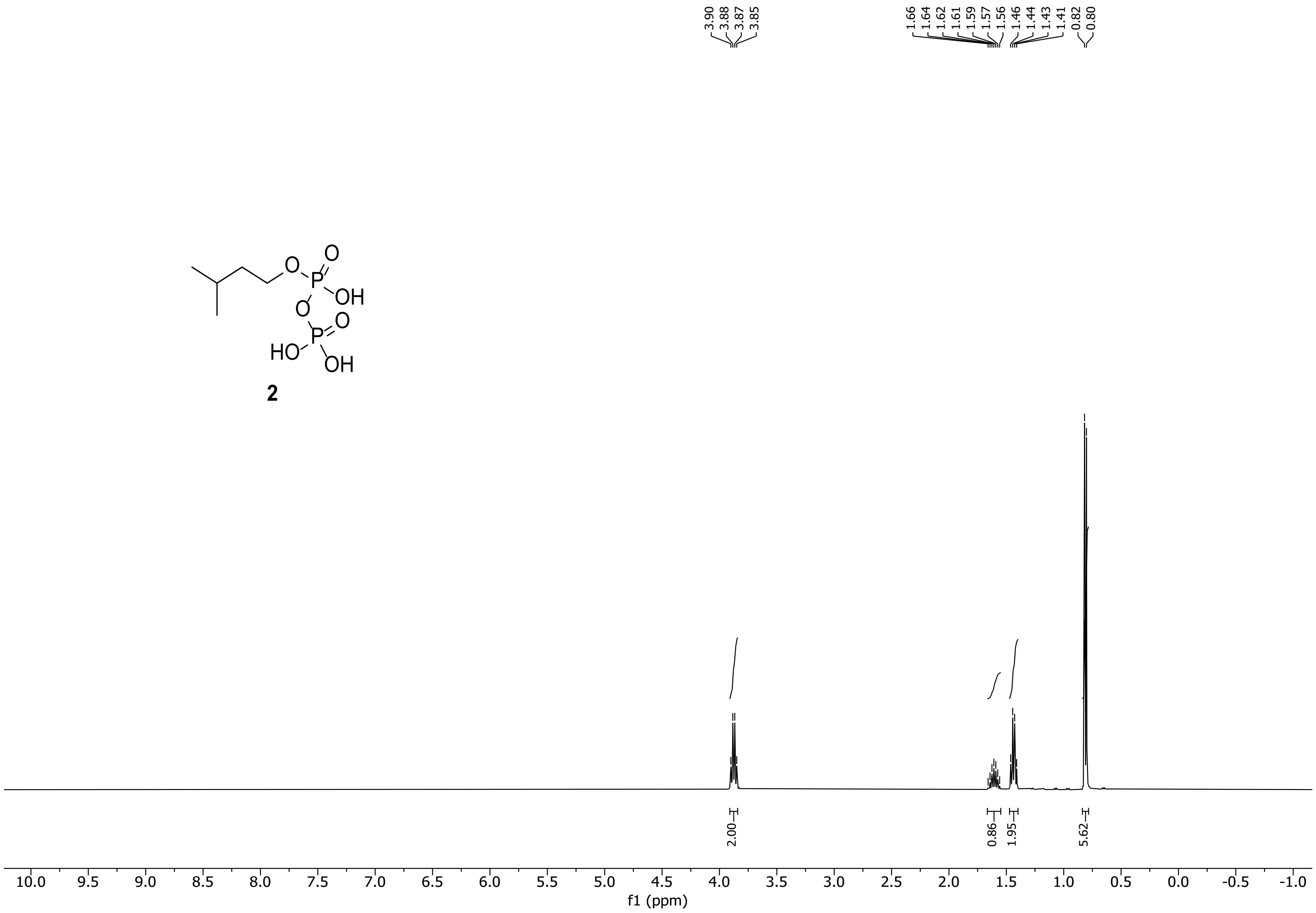

Figure S67 ^{1}H NMR (400 MHz) spectrum of isopentyl trihydrogen diphosphate (**2**) in D_2O.

`

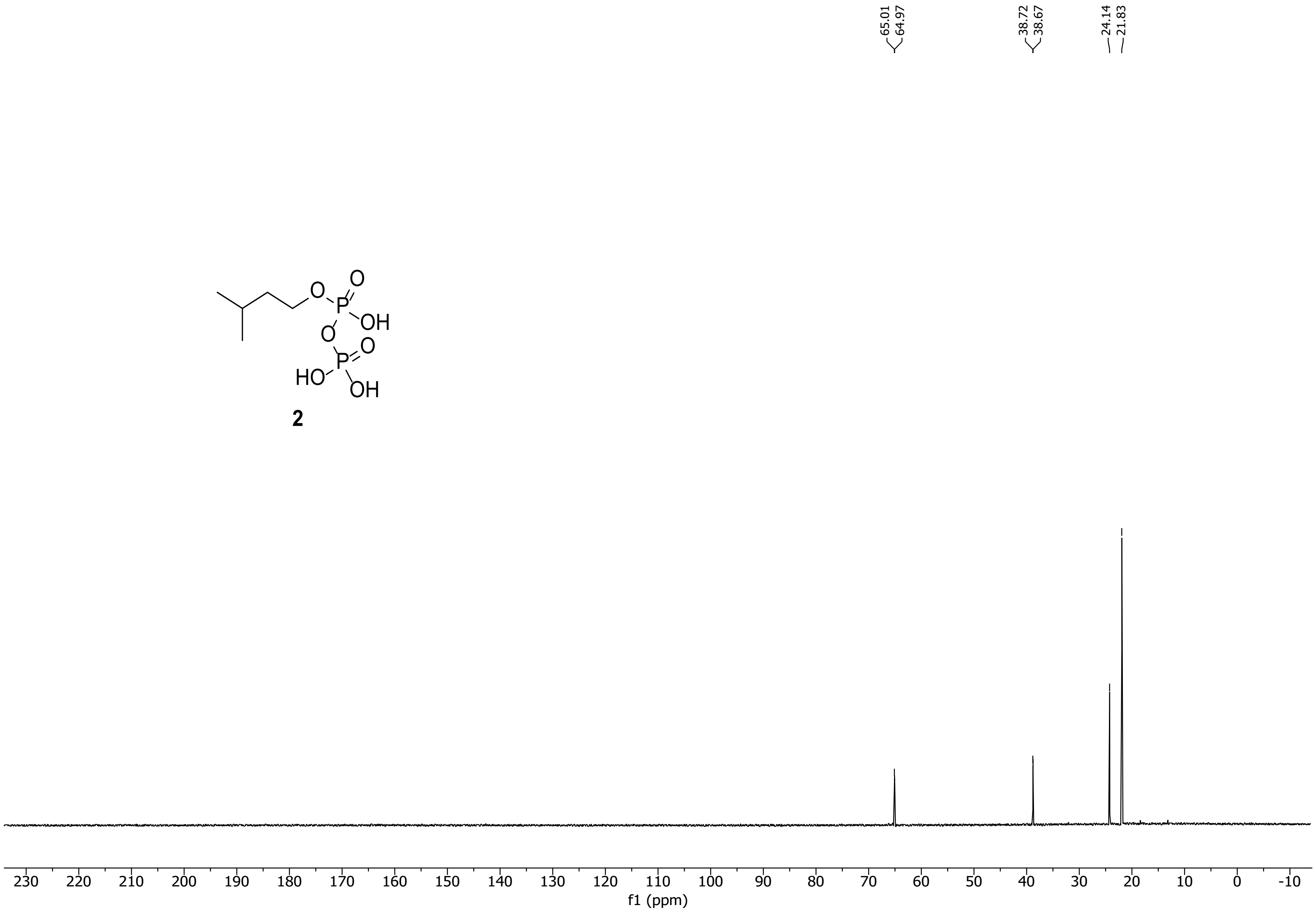

2

Figure S68 ^{13}C NMR (151 MHz) spectrum of isopentyl trihydrogen diphosphate (**2**) in D_2O.

`

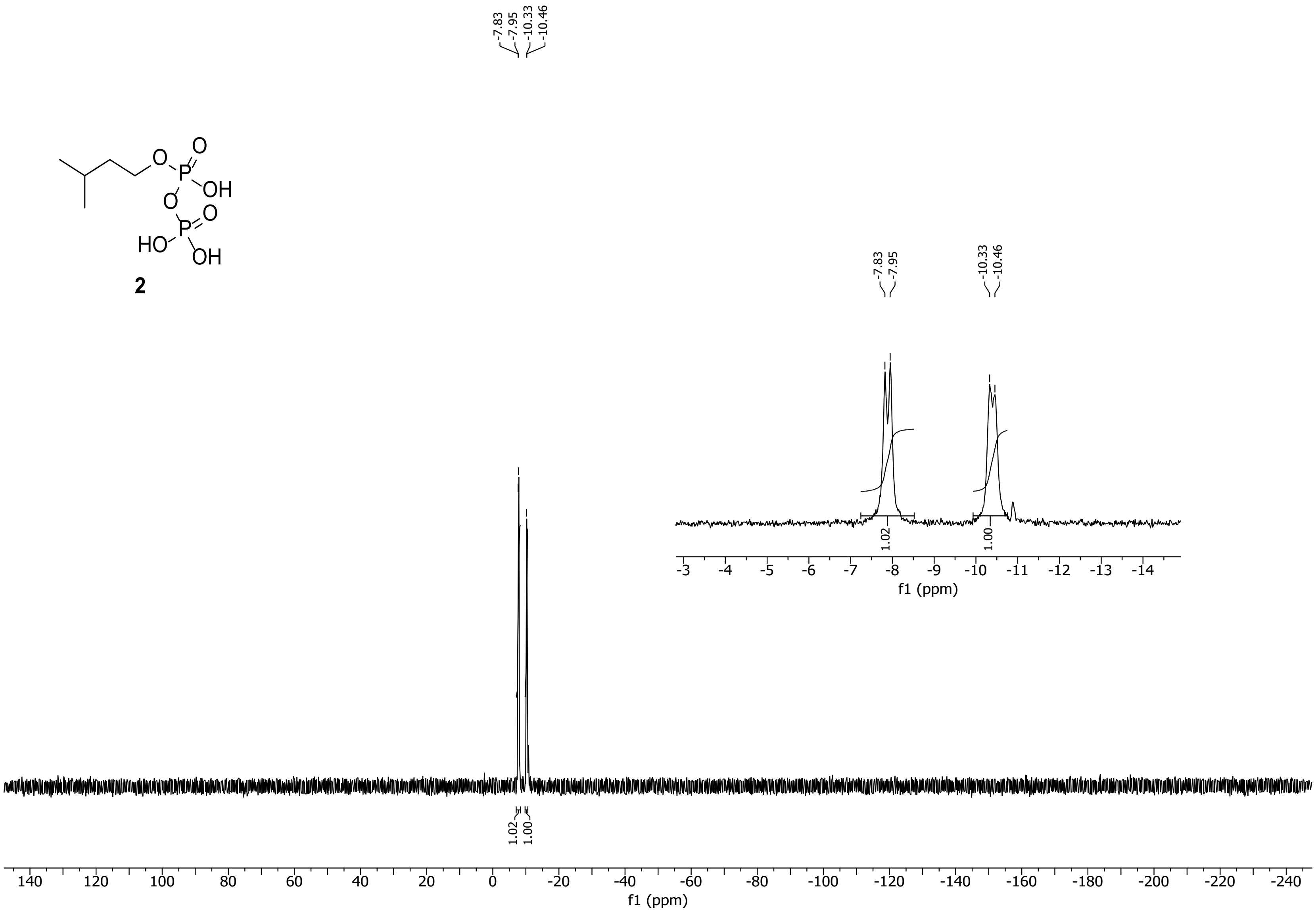

Figure S69 ^{31}P NMR (162 MHz) spectrum of isopentyl trihydrogen diphosphate (**2**) in D_2O.

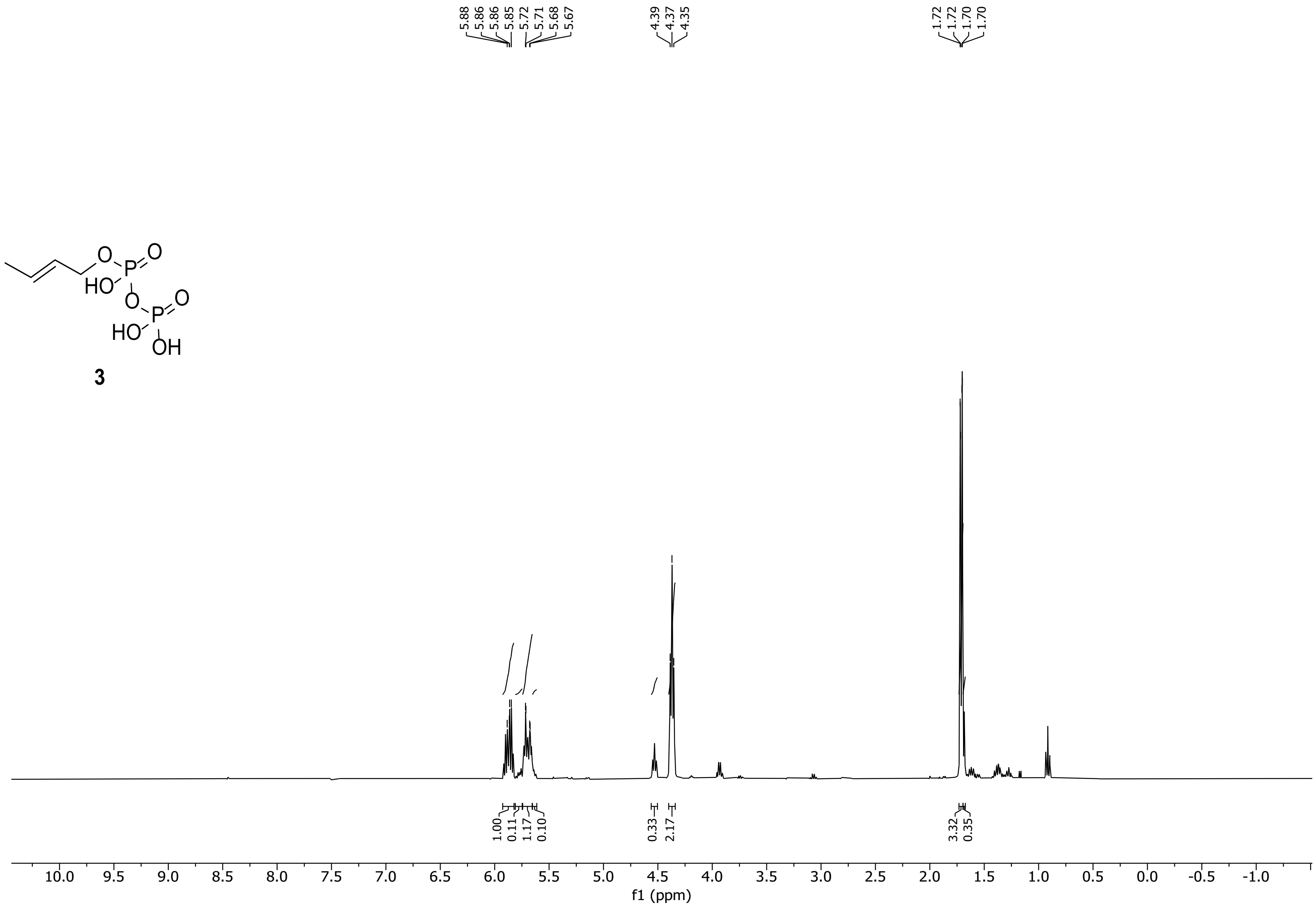

Figure S70 ^{1}H NMR (400 MHz) spectrum of (*E*)-but-2-en-1-yl trihydrogen diphosphate (**3**) n D_2O.

`

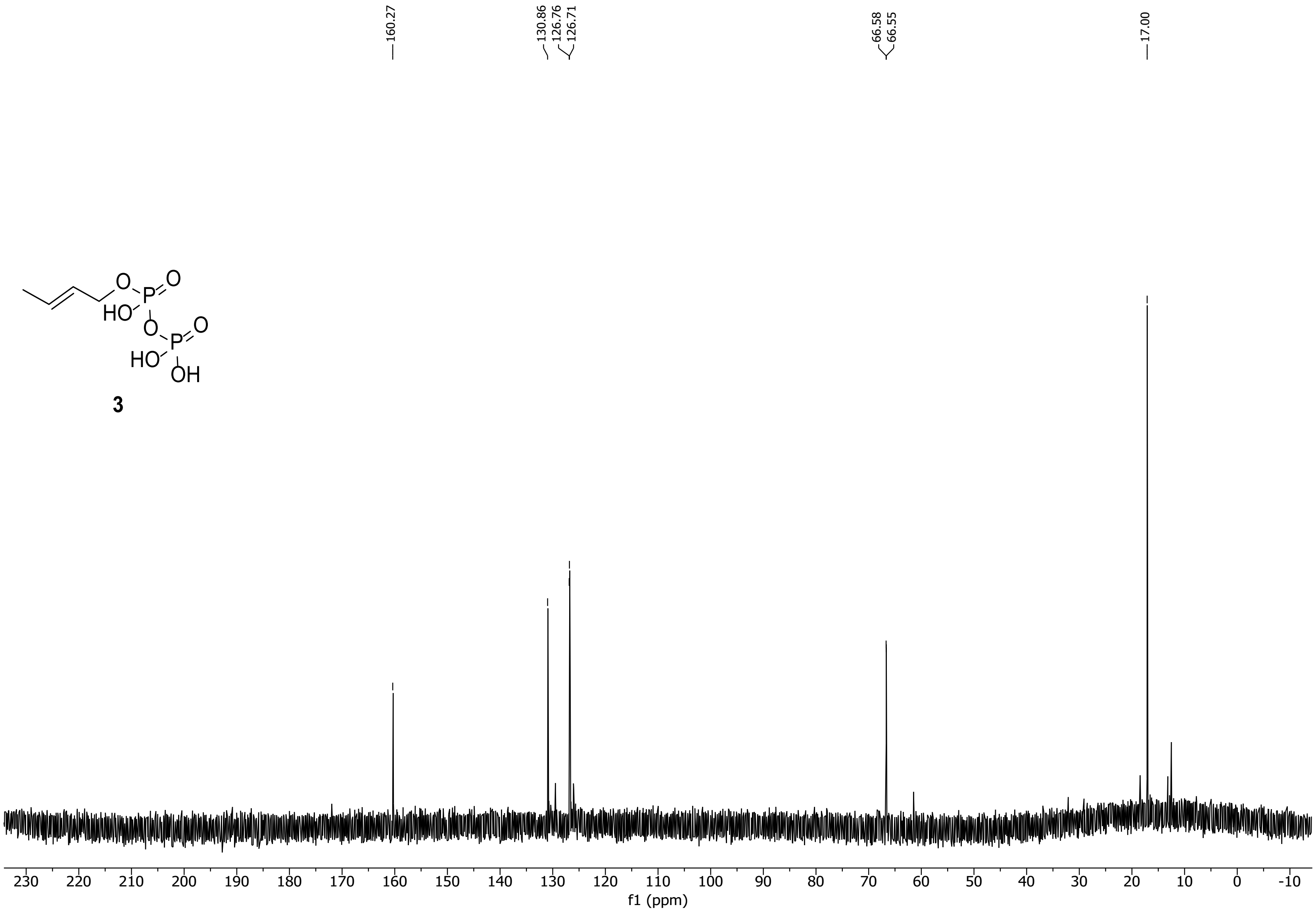

Figure S71 ^{13}C NMR (151 MHz) spectrum of (*E*)-but-2-en-1-yl trihydrogen diphosphate (**3**) n D_2O.

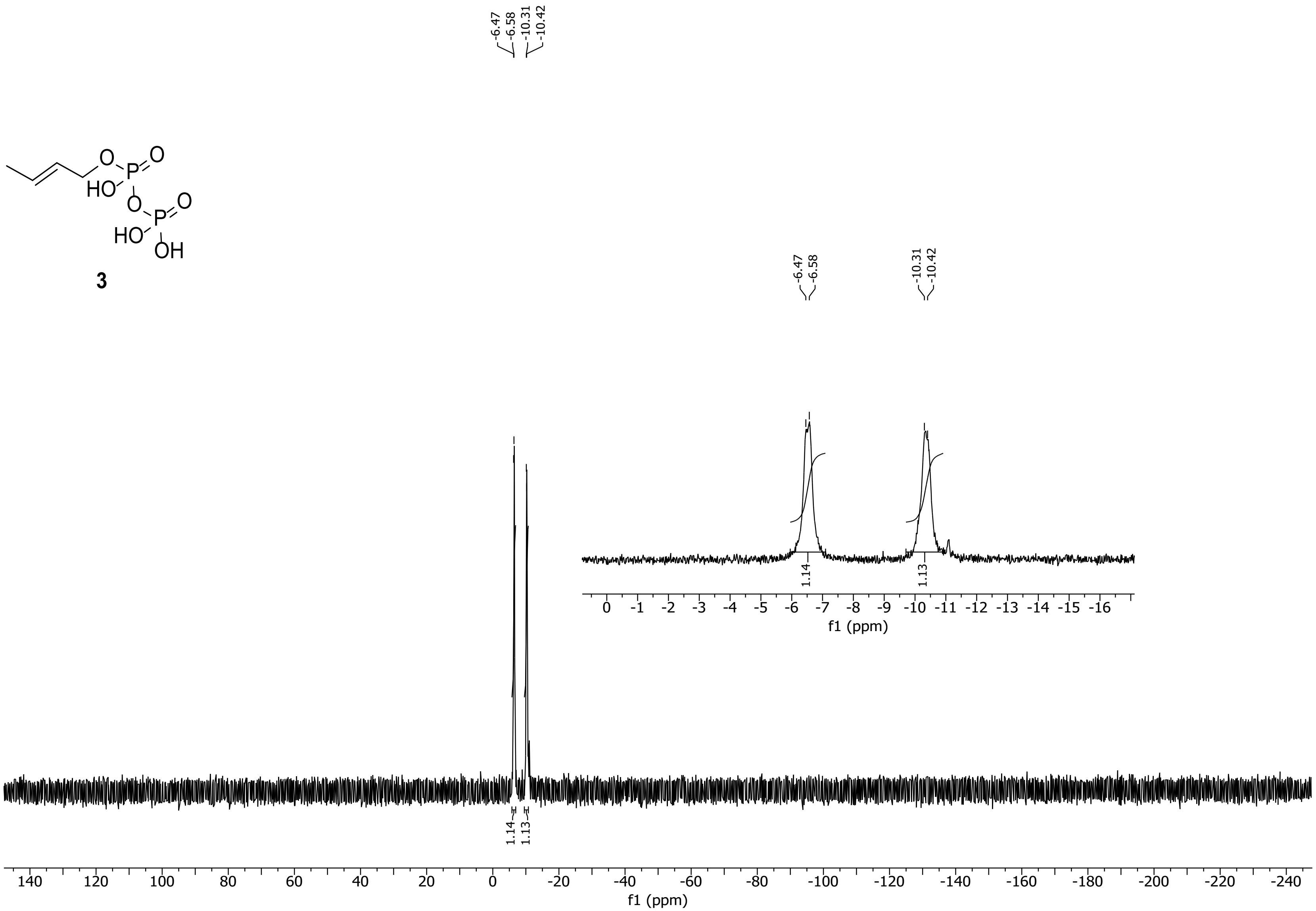

Figure S72 ^{31}P NMR (162 MHz) spectrum of (*E*)-but-2-en-1-yl trihydrogen diphosphate (**3**) n D_2O.

`

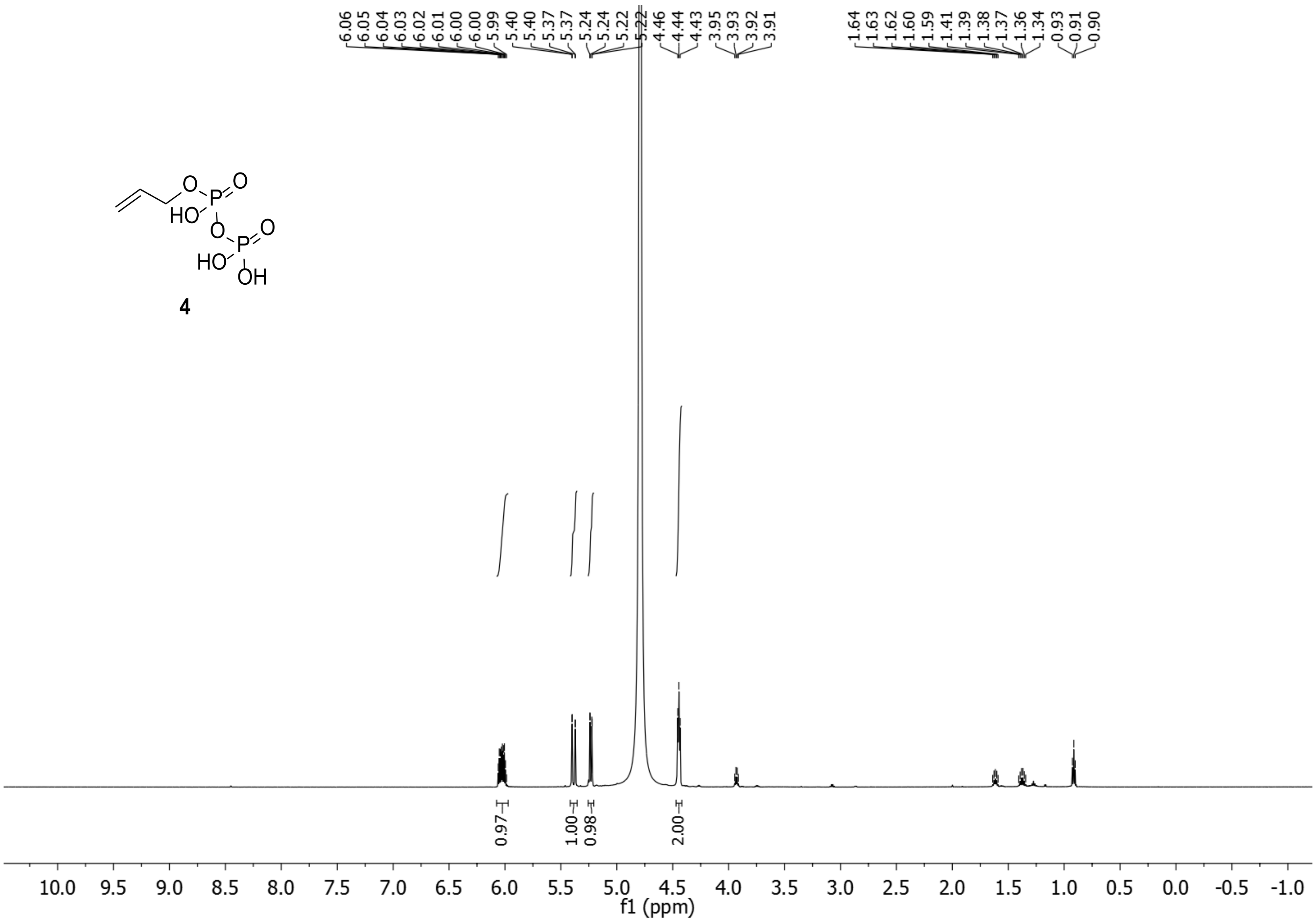

Figure S73 ^{1}H NMR (600 MHz) spectrum of allyl trihydrogen diphosphate (**4**) in D_2O.

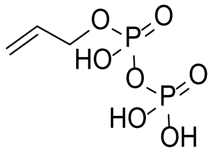

4

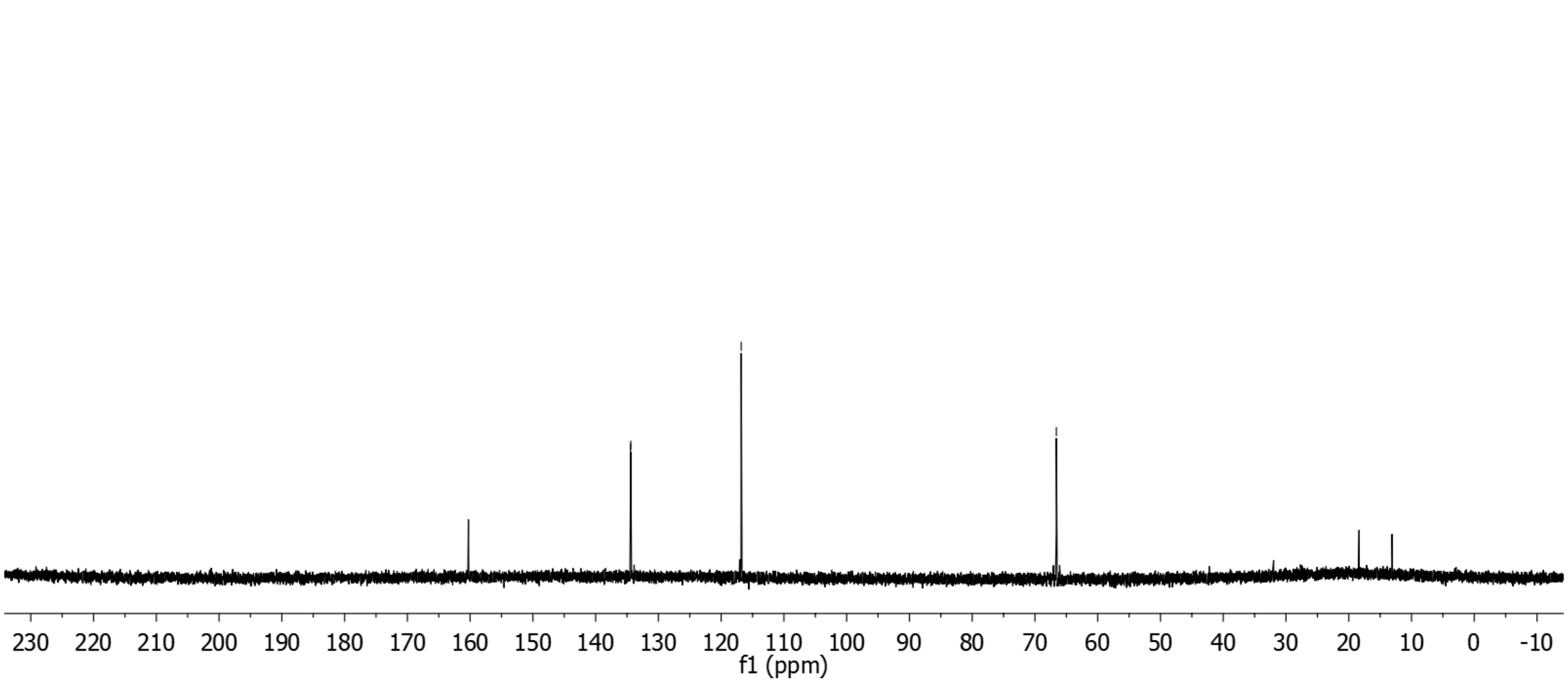

Figure S74 ^{13}C NMR (151 MHz) spectrum of allyl trihydrogen diphosphate (**4**) in D_2O.

`

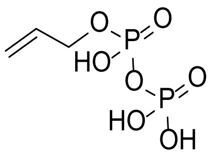

4

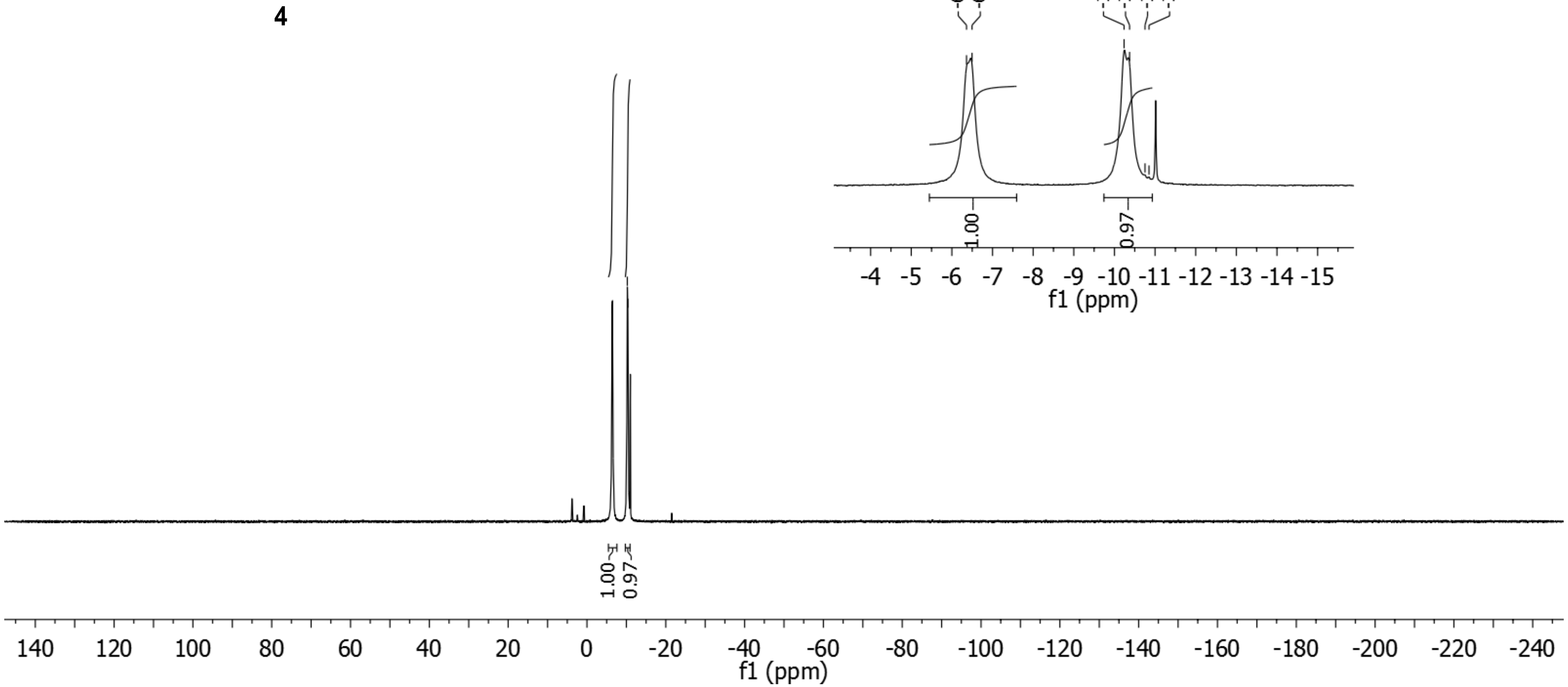

Figure S75 ^{31}P NMR (162 MHz) spectrum of allyl trihydrogen diphosphate (**4**) in D_2O.

`

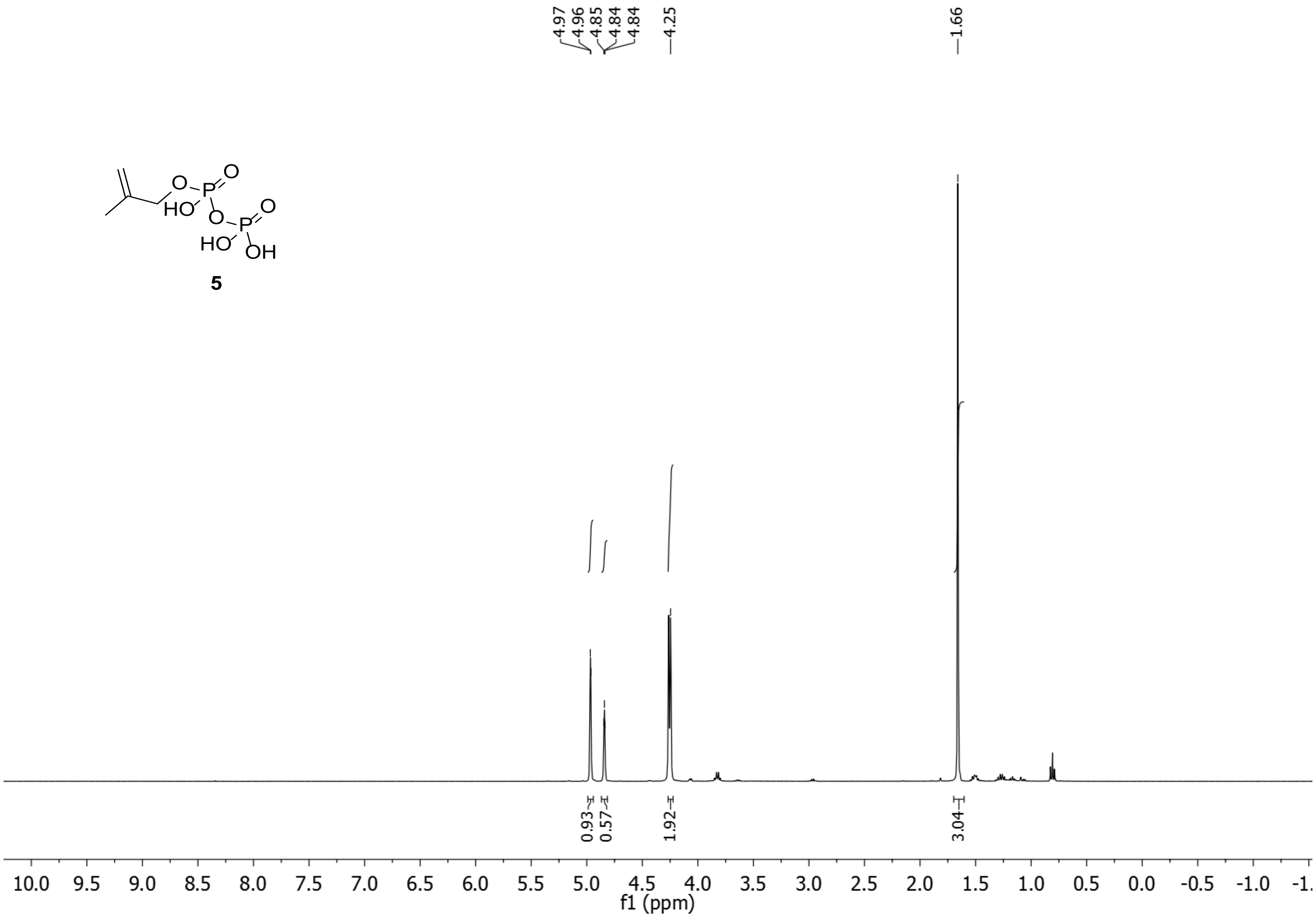

5

Figure S76 ¹H NMR (400 MHz) spectrum of 2-methylallyl trihydrogen diphosphate (**5**) in D_2O.

`

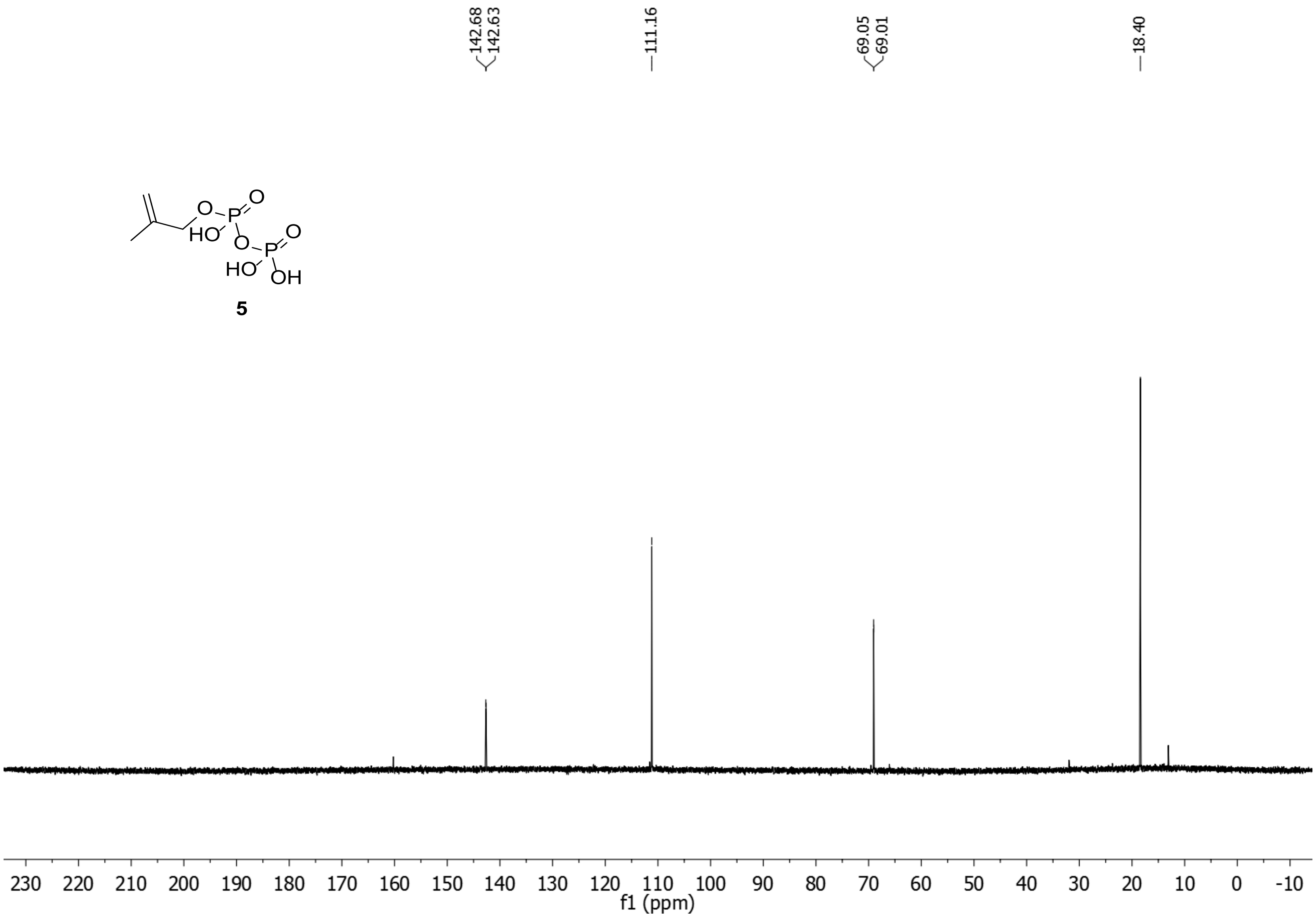

Figure S77 ^{13}C NMR (151 MHz) spectrum of 2-methylallyl trihydrogen diphosphate (**5**) in D_2O.

`

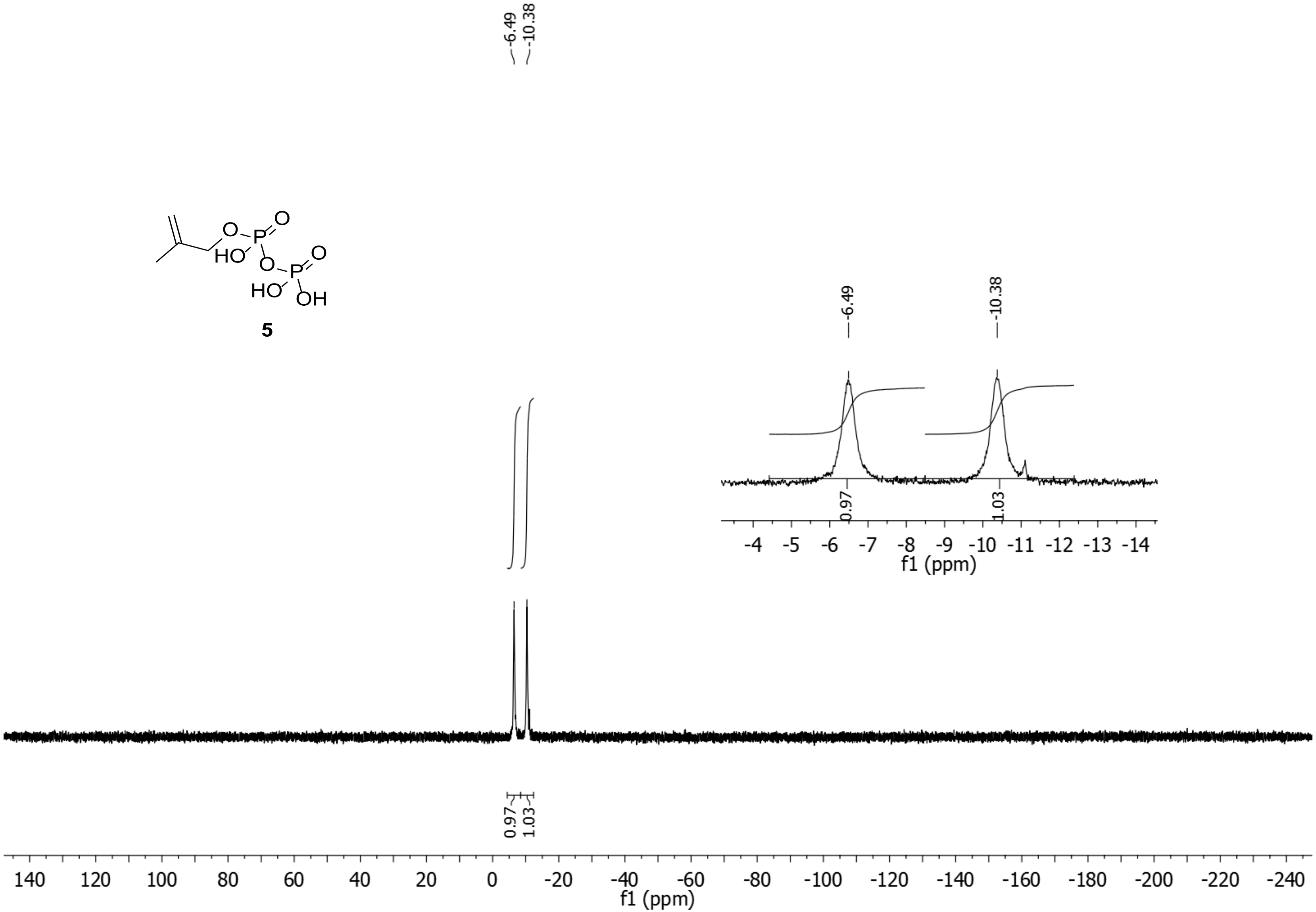

Figure S78 ^{31}P NMR (162 MHz) spectrum of 2-methylallyl trihydrogen diphosphate (**5**) in D_2O.

`

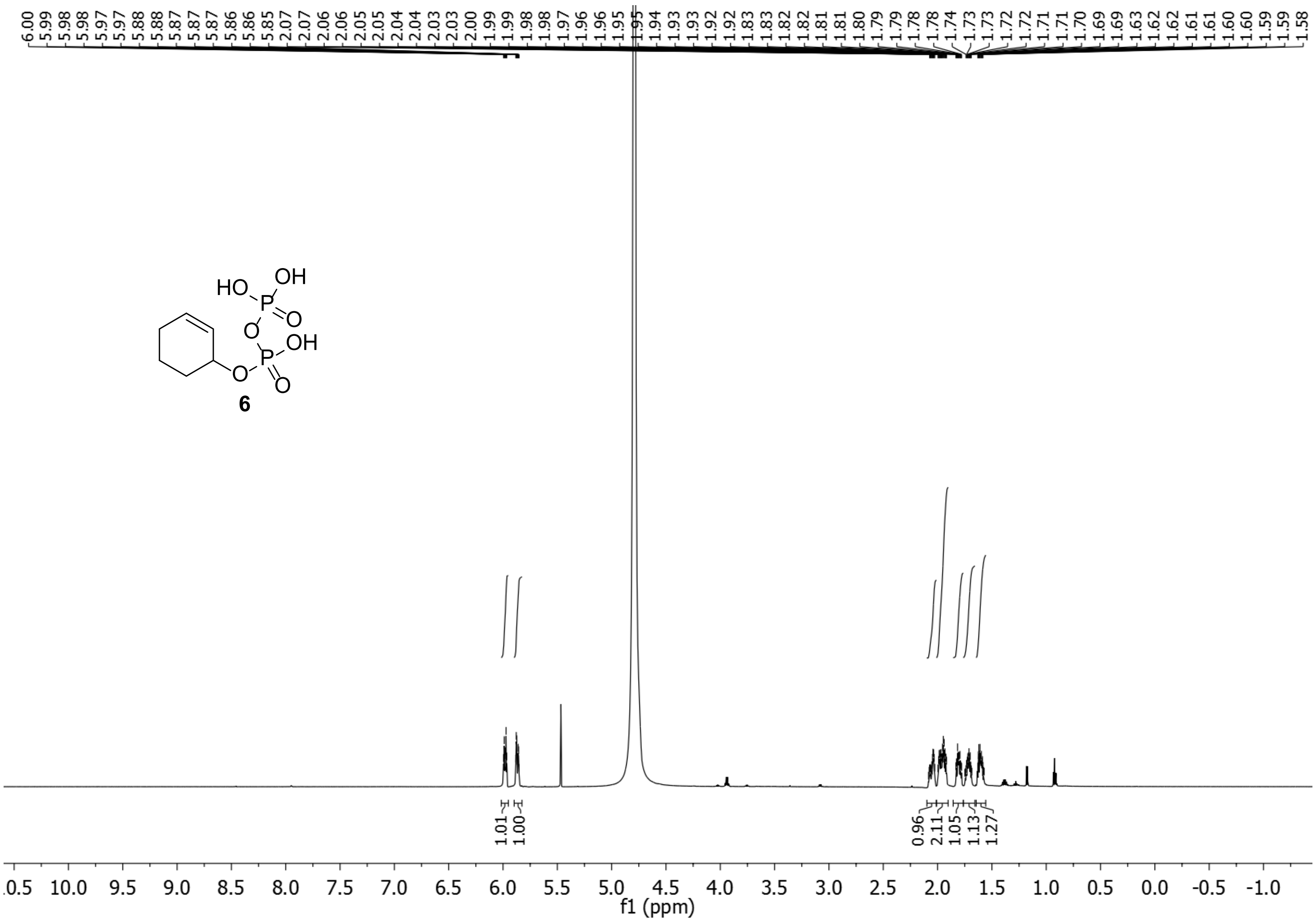

Figure S79 ^{1}H NMR (600 MHz) spectrum of (±)-cyclohex-2-en-1-yl trihydrogen diphosphate (**6**) in D_2O.

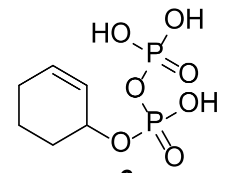

6

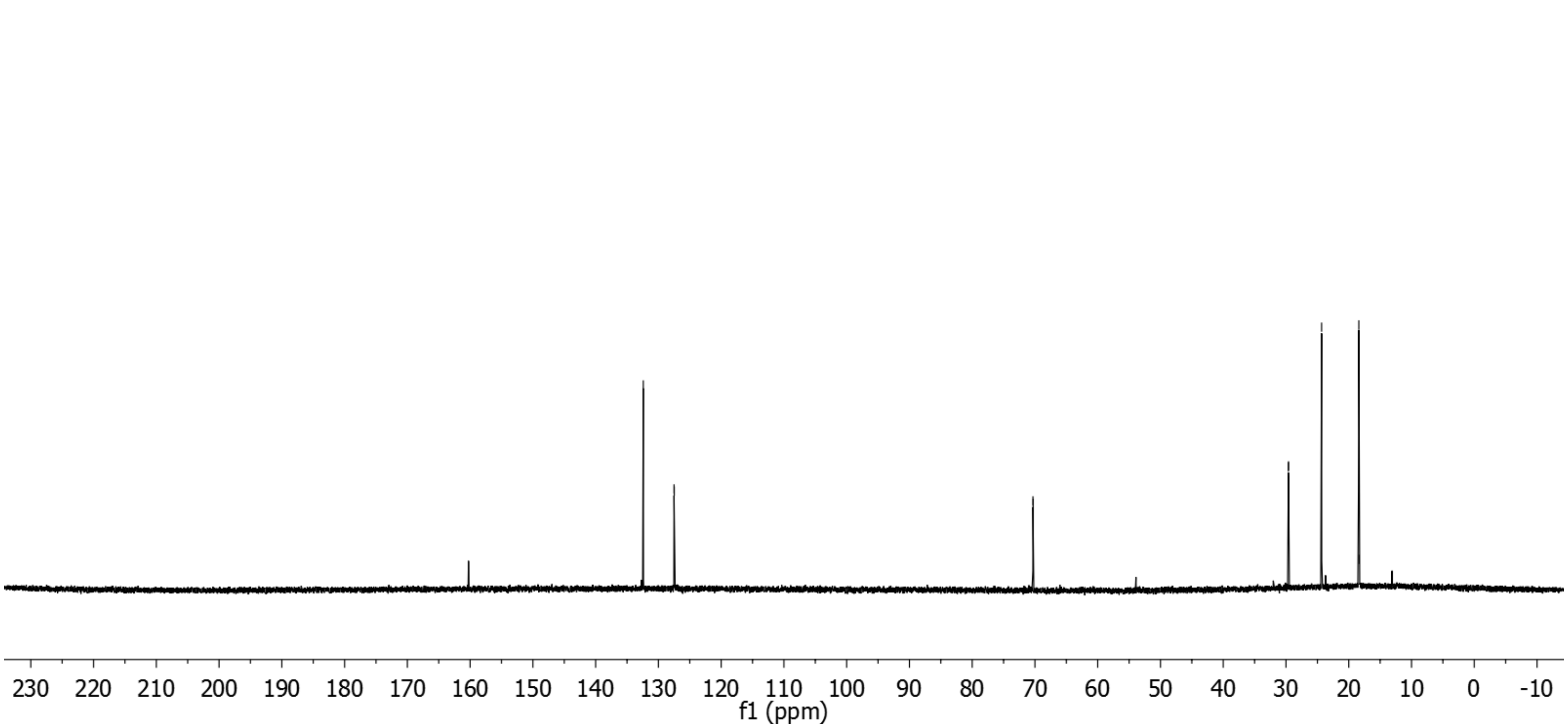

Figure S80 ^{13}C NMR (151 MHz) spectrum of (±)-cyclohex-2-en-1-yl trihydrogen diphosphate (**6**) in D_2O.

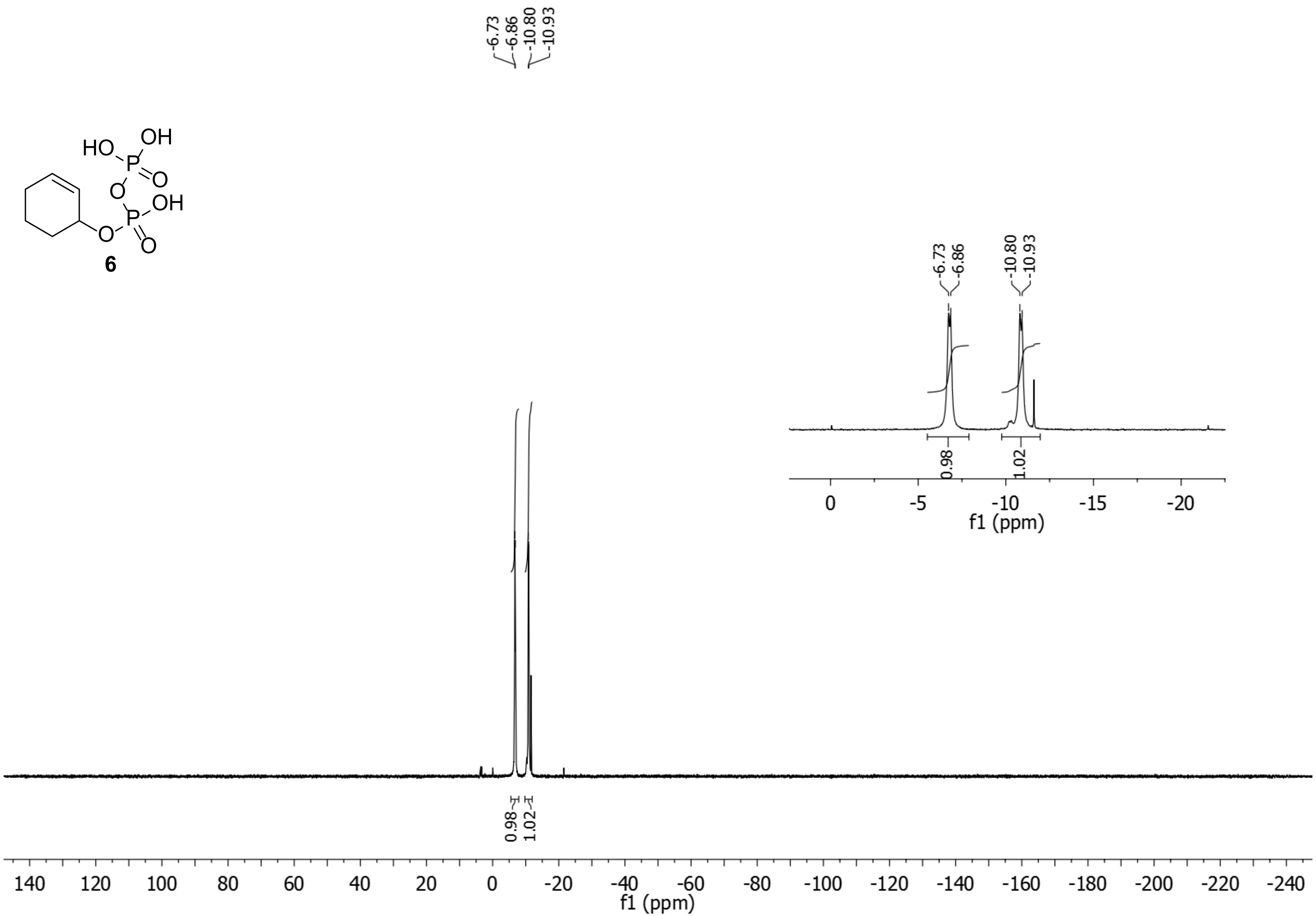

Figure S81 ^{31}P NMR (162 MHz) spectrum of (±)-cyclohex-2-en-1-yl trihydrogen diphosphate (**6**) in D_2O.

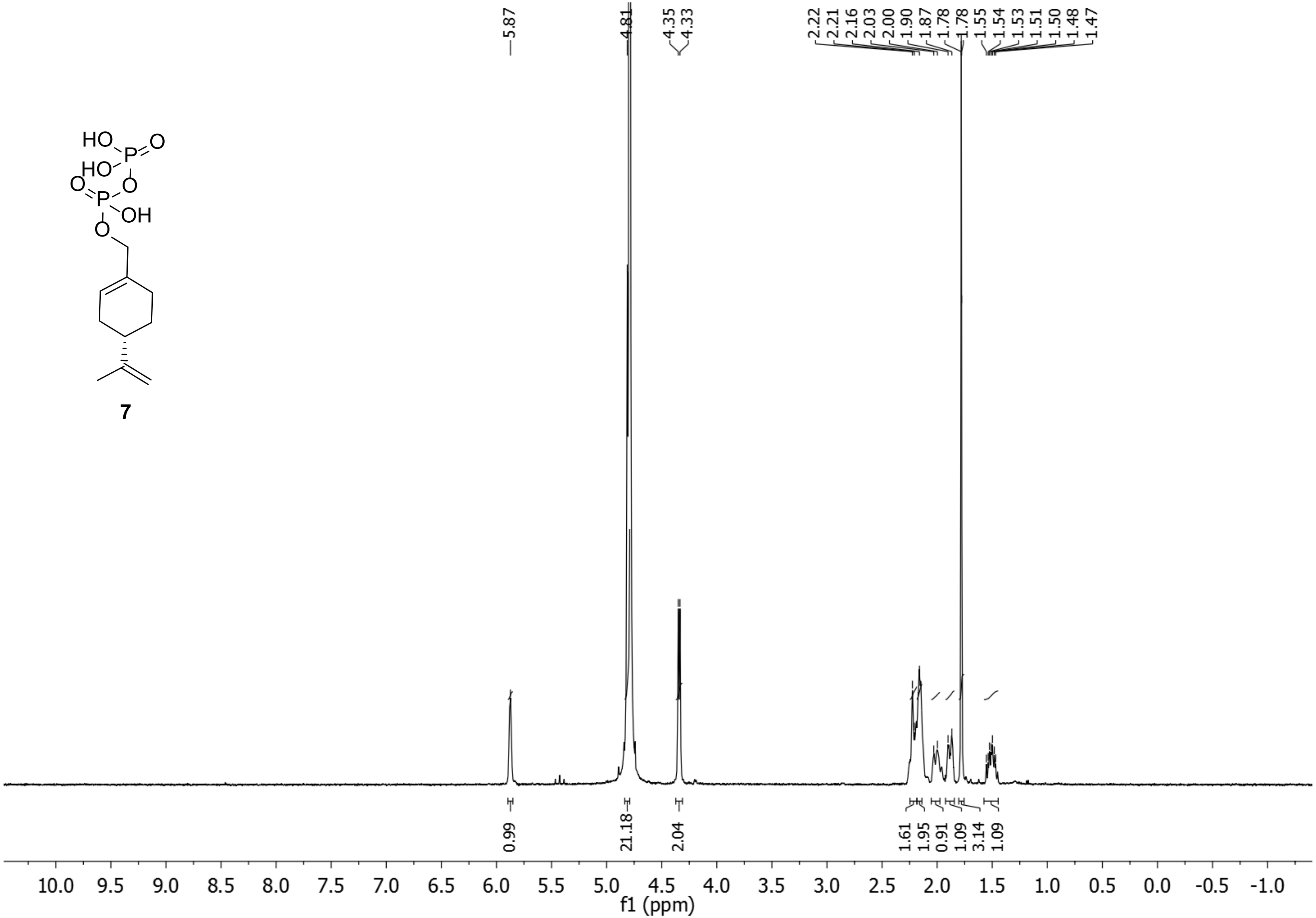

Figure S82 ^{1}H NMR (400 MHz) spectrum of ((S)-4-(prop-1-en-2-yl)cyclohex-1-en-1-yl)methyl trihydrogen diphosphate (**7**) in D_2O.

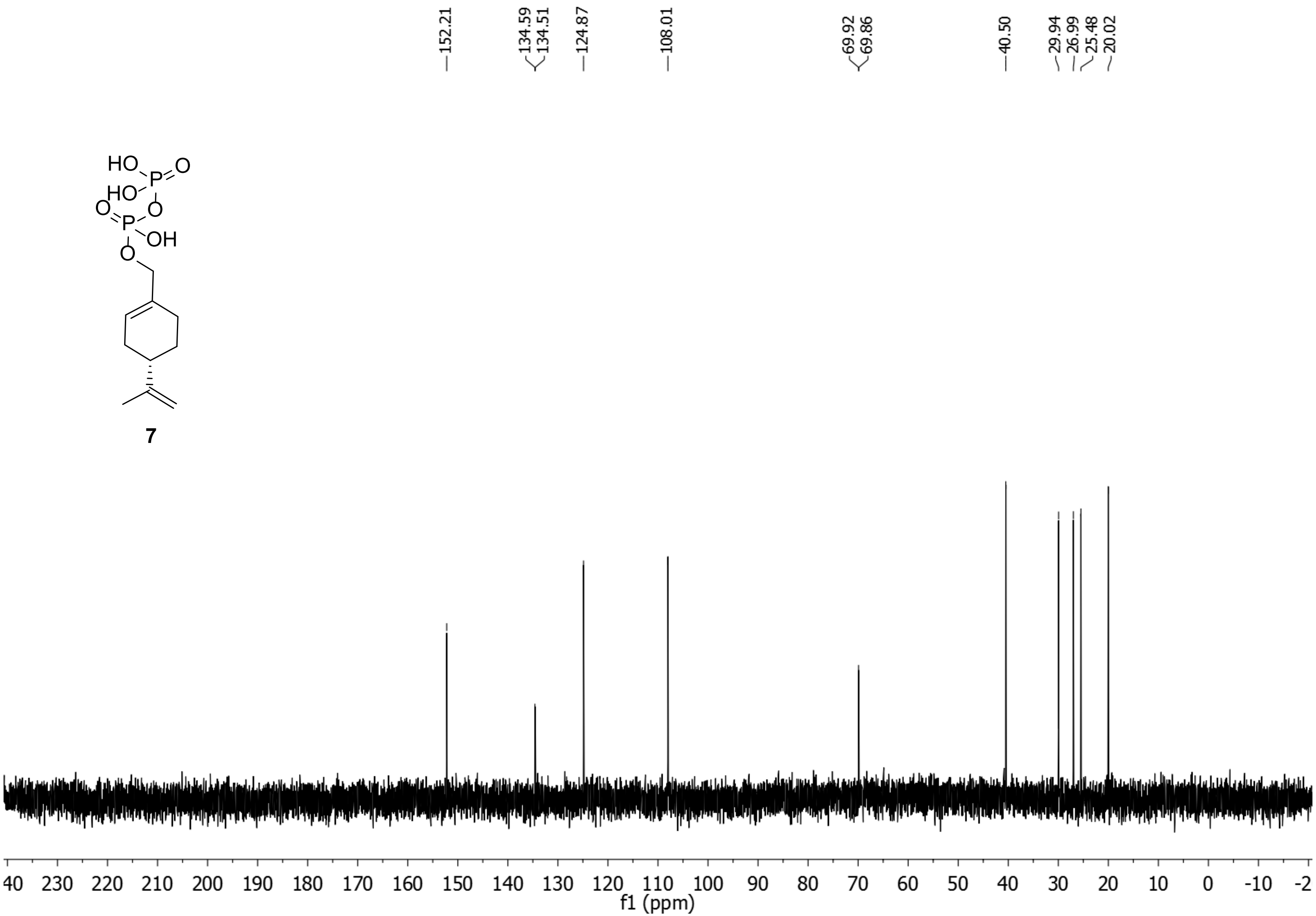

Figure S83 ^{13}C NMR (101 MHz) spectrum of ((S)-4-(prop-1-en-2-yl)cyclohex-1-en-1-yl)methyl trihydrogen diphosphate (**7**) in D_2O.

`

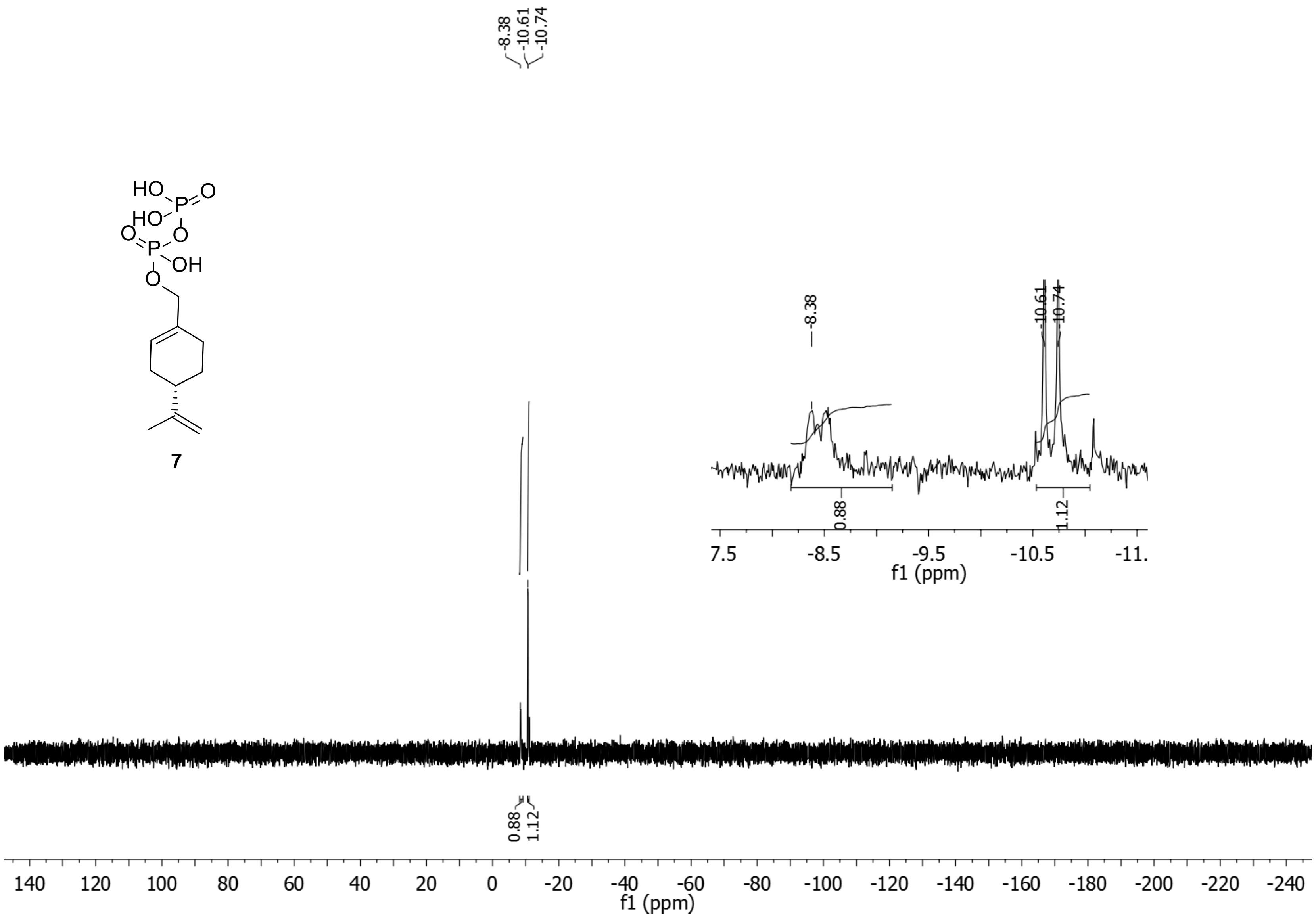

Figure S84 ^{31}P NMR (162 MHz) spectrum of ((S)-4-(prop-1-en-2-yl)cyclohex-1-en-1-yl)methyl trihydrogen diphosphate (**7**) in D_2O.

`

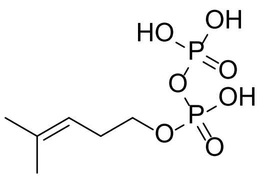

9

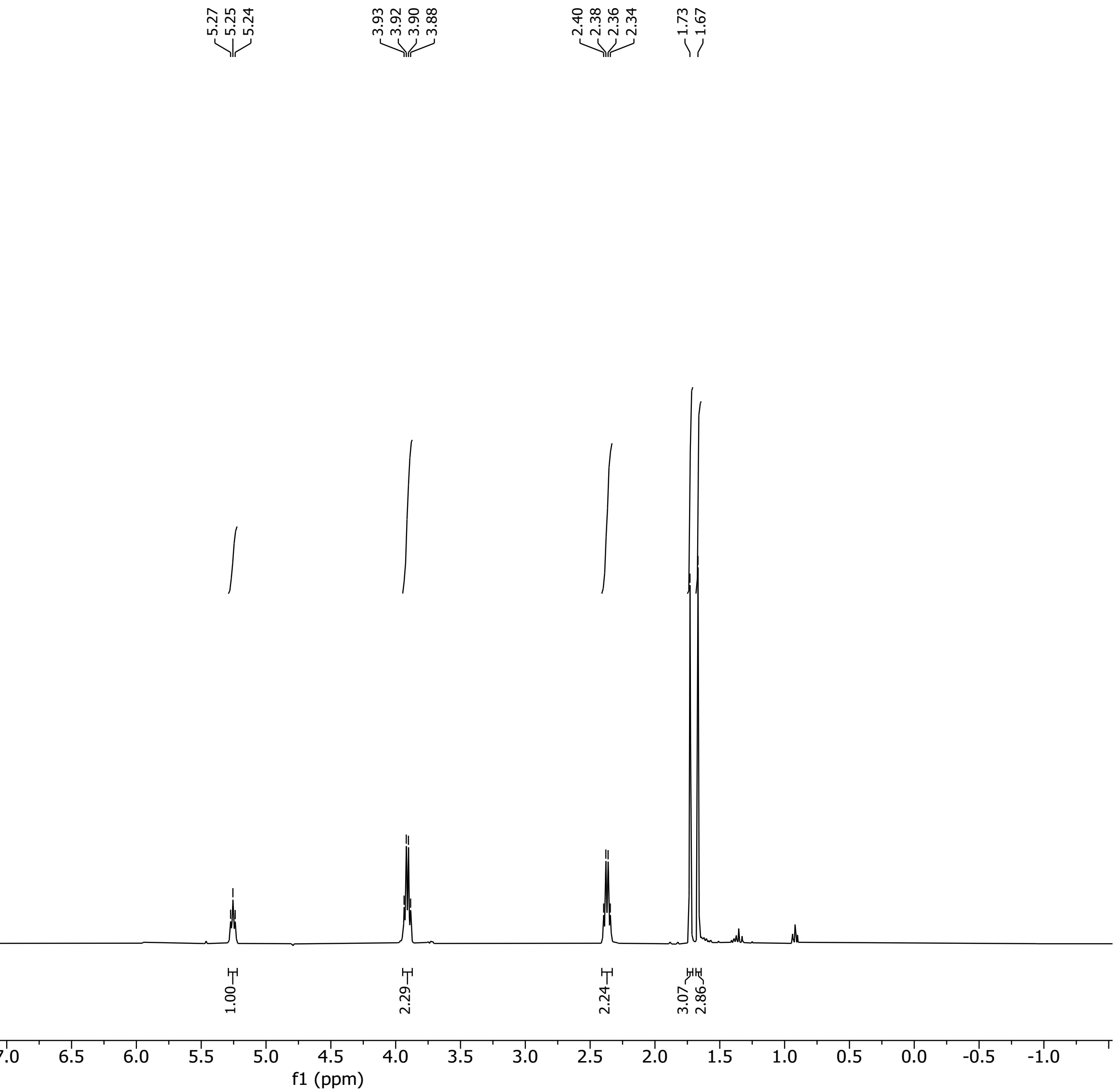

Figure S85 ^{1}H NMR (400 MHz) spectrum of 4-methylpent-3-en-1-yl trihydrogen diphosphate (**9**) in D_2O.

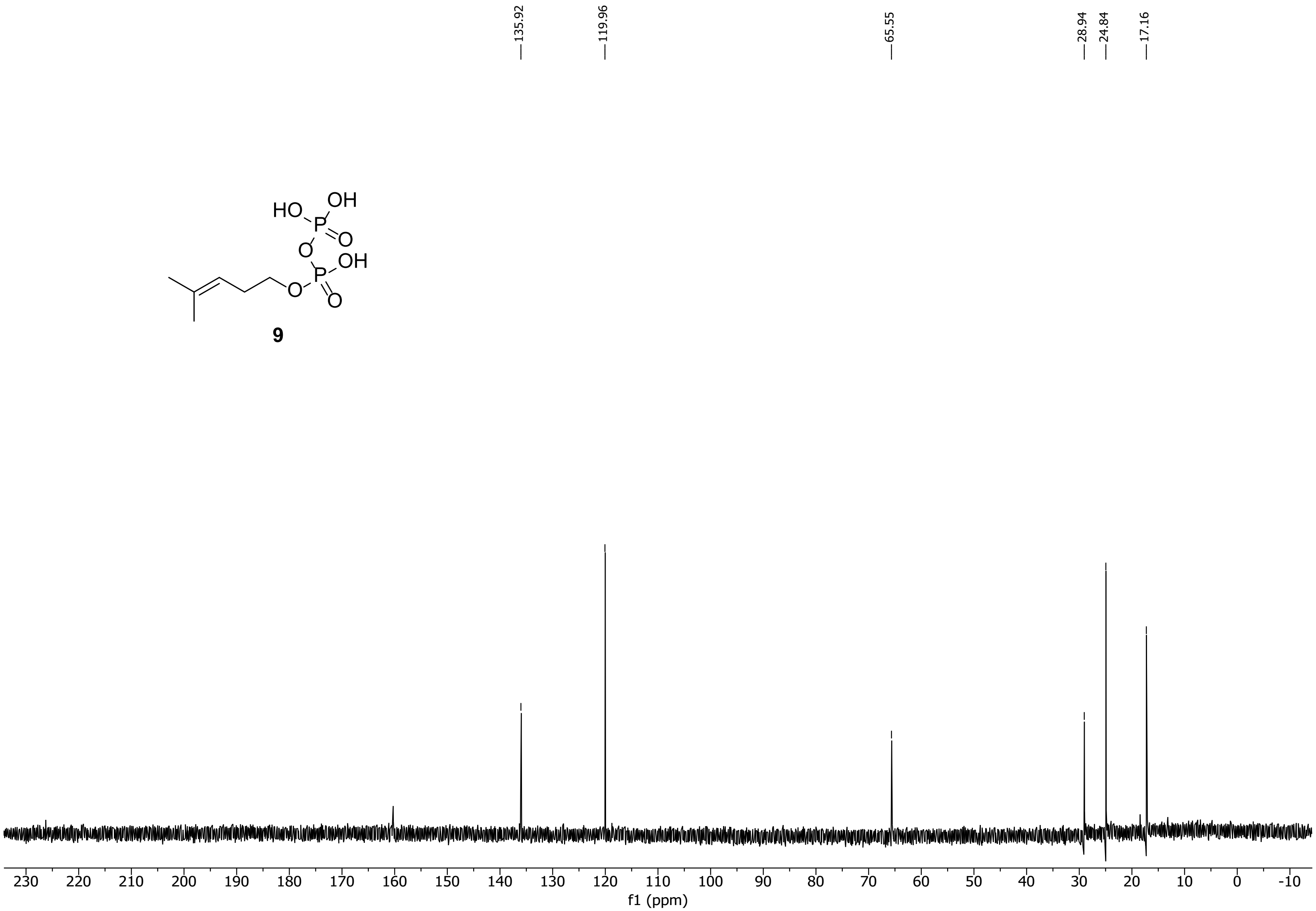

Figure S86 ^{13}C NMR (151 MHz) spectrum of 4-methylpent-3-en-1-yl trihydrogen diphosphate (**9**) in D_2O.

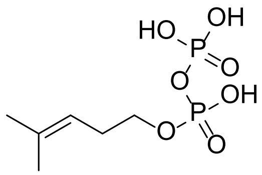

9

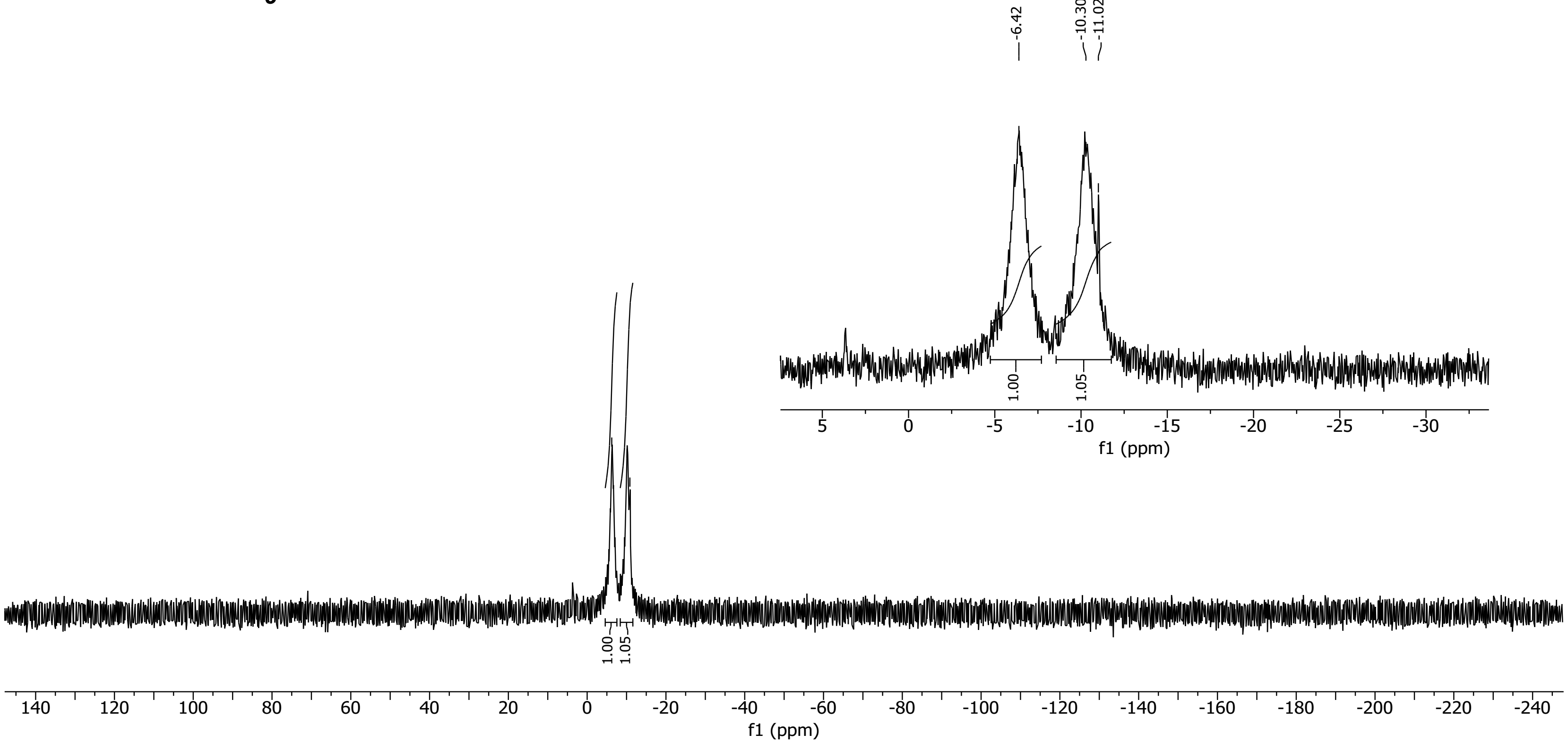

Figure S87 ^{31}P NMR (162 MHz) spectrum of 4-methylpent-3-en-1-yl trihydrogen diphosphate (**9**) in D_2O.

`

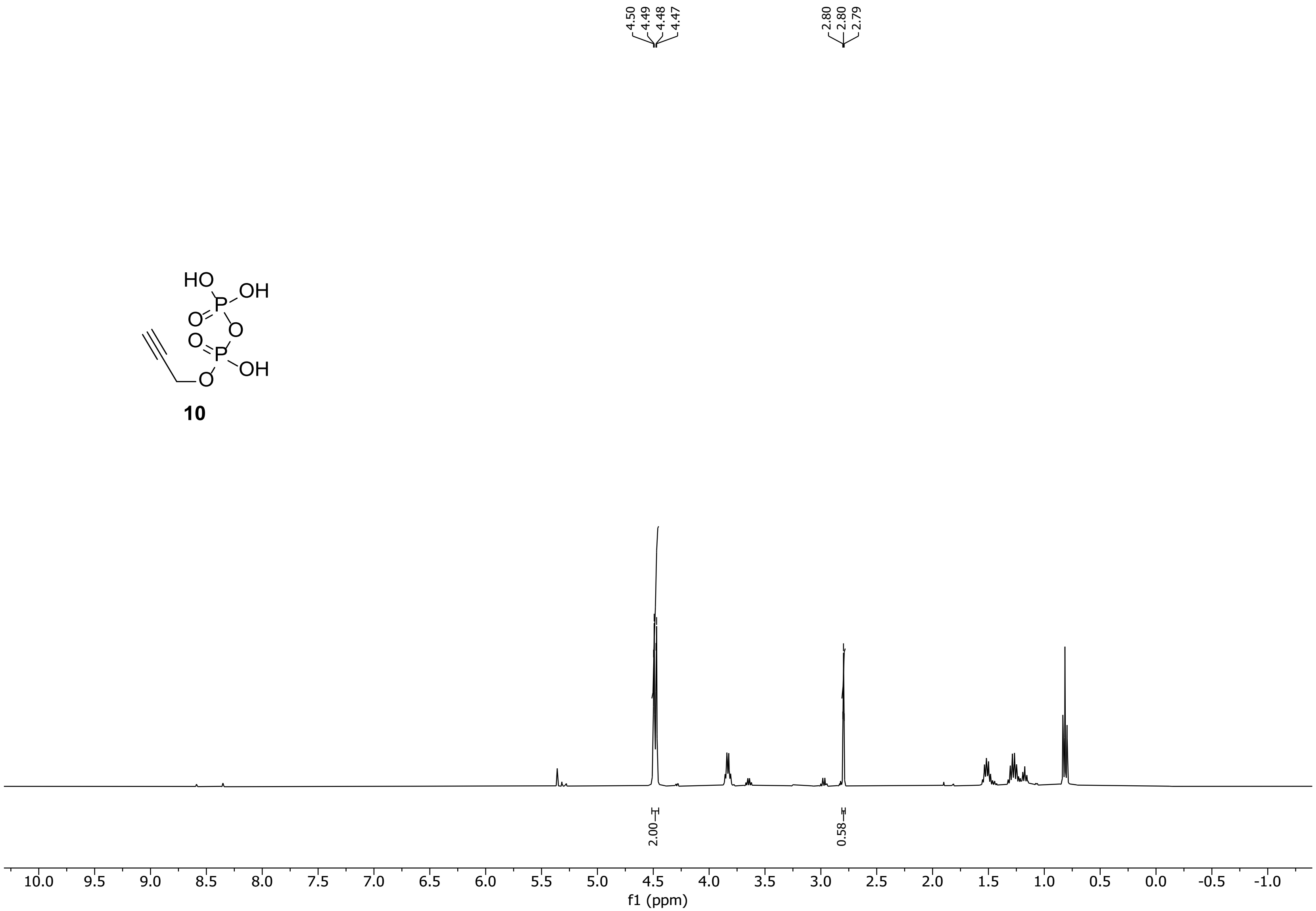

10

Figure S88 ^{1}H NMR (400 MHz) spectrum of prop-2-yn-1-yl diphosphate (**10**) in D_2O.

`

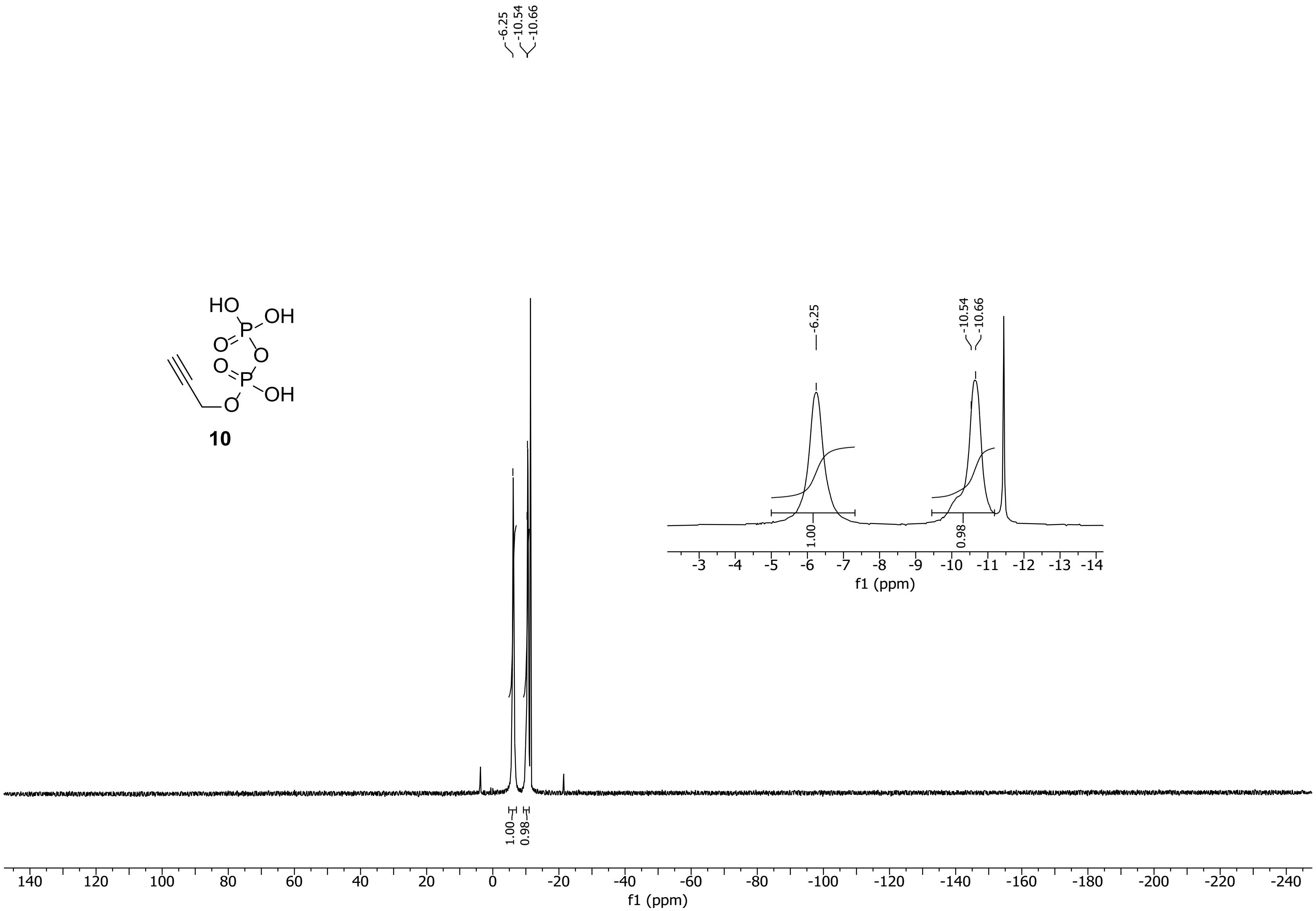

Figure S89 ^{31}P NMR (162 MHz) spectrum of prop-2-yn-1-yl diphosphate (**10**) in D_2O.

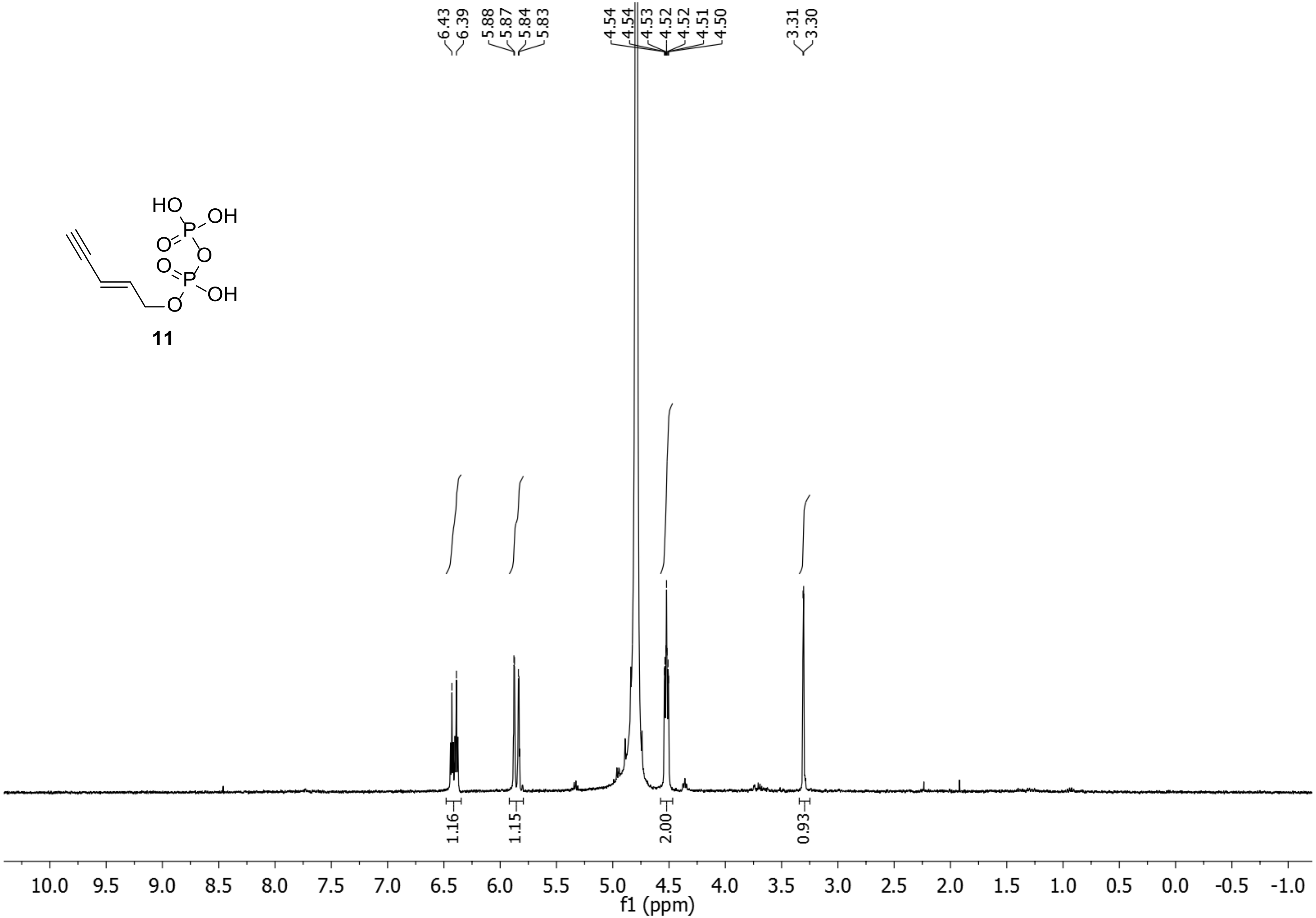

Figure S90 ^{1}H NMR (400 MHz) spectrum of (*E*)-pent-2-en-4-yn-1-yl trihydrogen diphosphate (**11**) in D_2O.

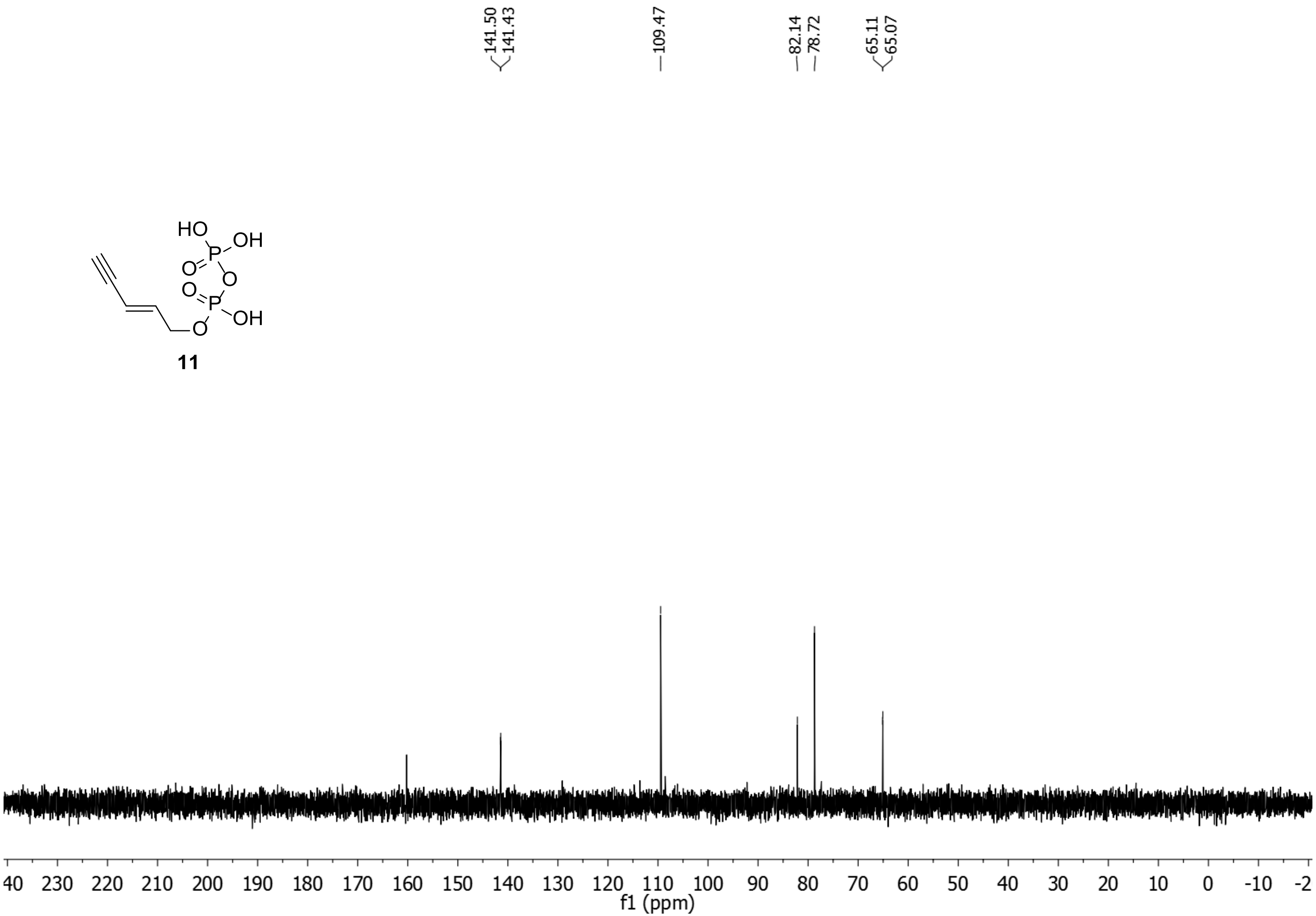

Figure S91 ^{13}C NMR (101 MHz) spectrum of (*E*)-pent-2-en-4-yn-1-yl trihydrogen diphosphate (**11**) in D_2O.

`

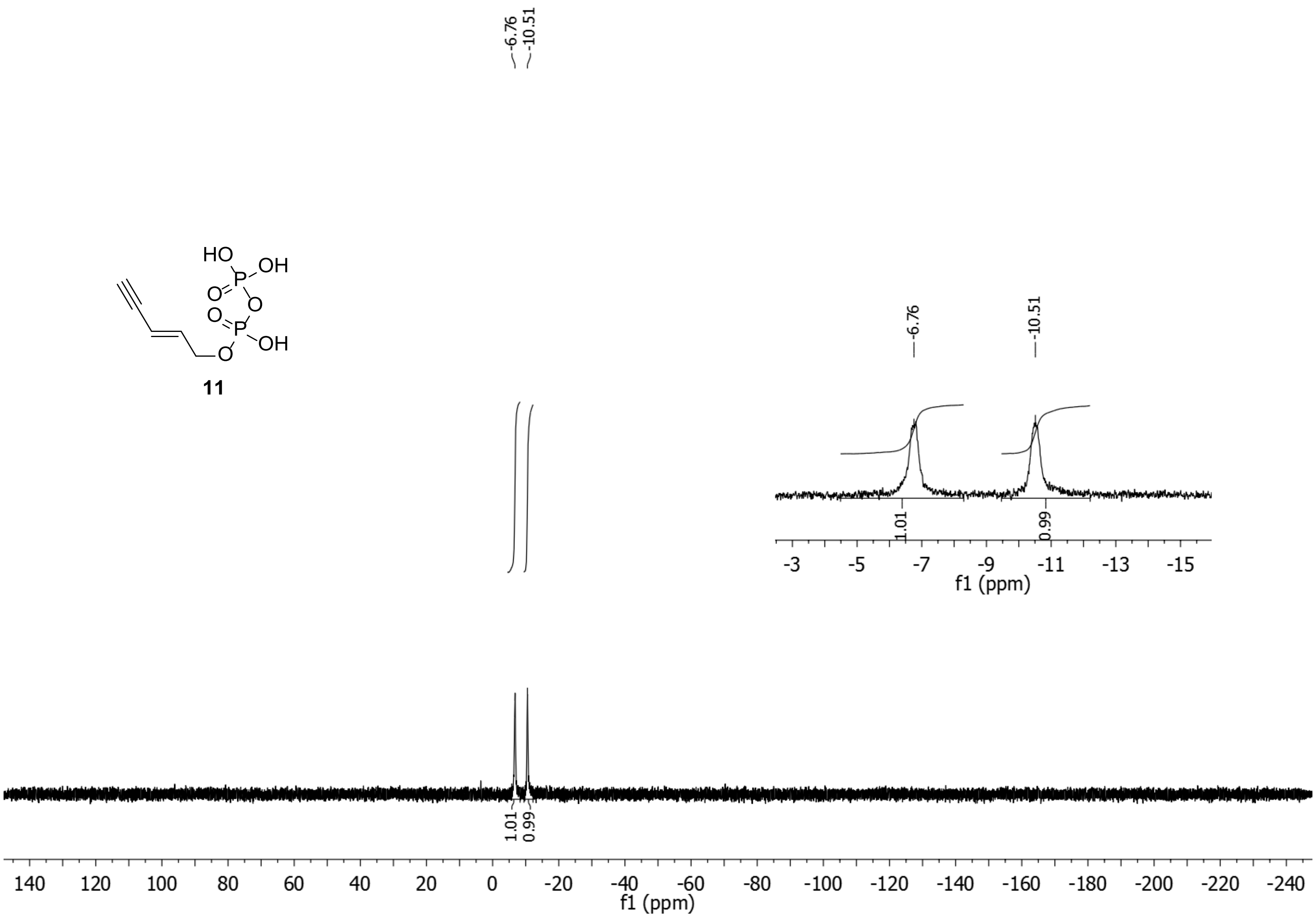

Figure S92 ^{31}P NMR (162 MHz) spectrum of (*E*)-pent-2-en-4-yn-1-yl trihydrogen diphosphate (**11**) in D_2O.

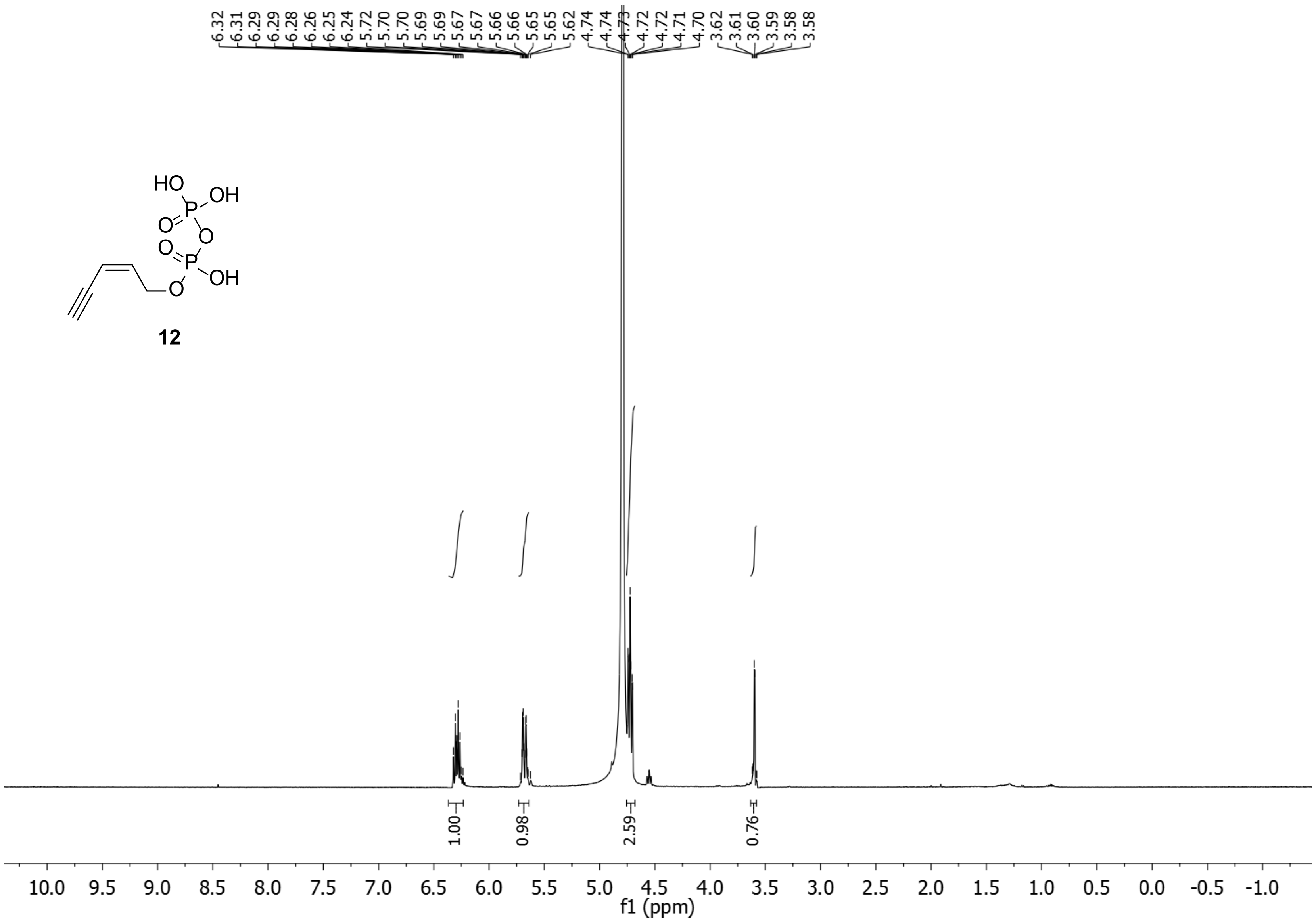

Figure S93 ^{1}H NMR (400 MHz) spectrum of (*Z*)-pent-2-en-4-yn-1-yl trihydrogen diphosphate (**12**) in D_2O.

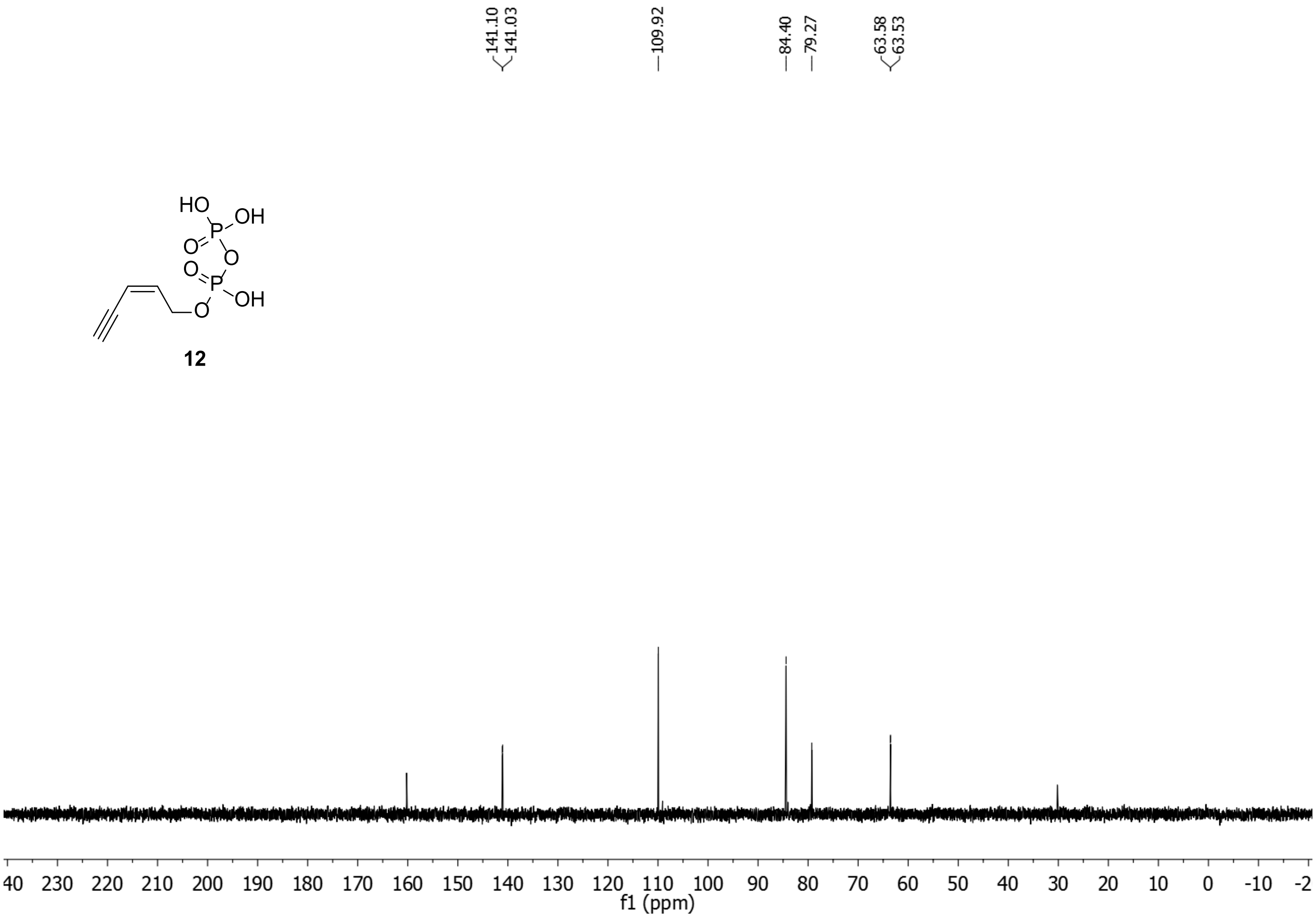

Figure S94 ^{13}C NMR (101 MHz) spectrum of (*Z*)-pent-2-en-4-yn-1-yl trihydrogen diphosphate (**12**) in D_2O.

`

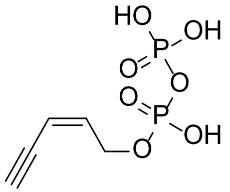

12

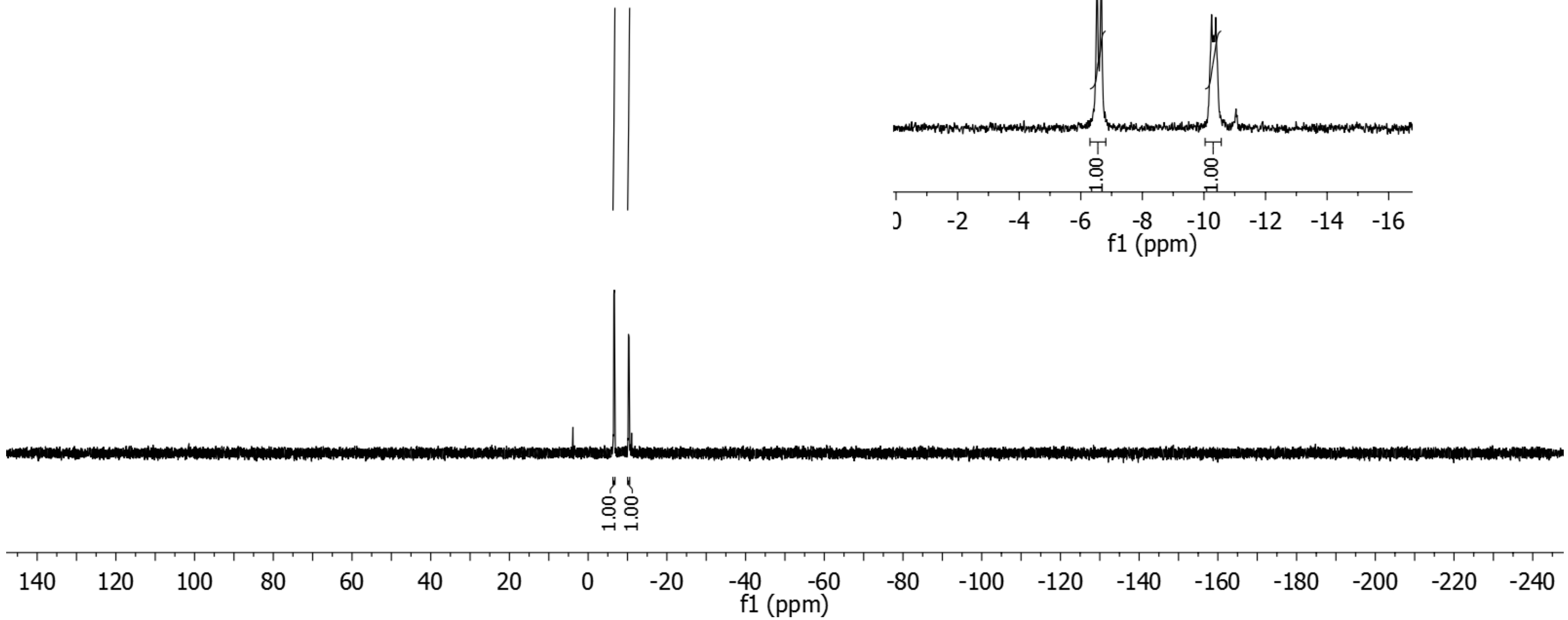

Figure S95 ^{31}P NMR (162 MHz) spectrum of (*Z*)-pent-2-en-4-yn-1-yl trihydrogen diphosphate (**12**) in D_2O.

`

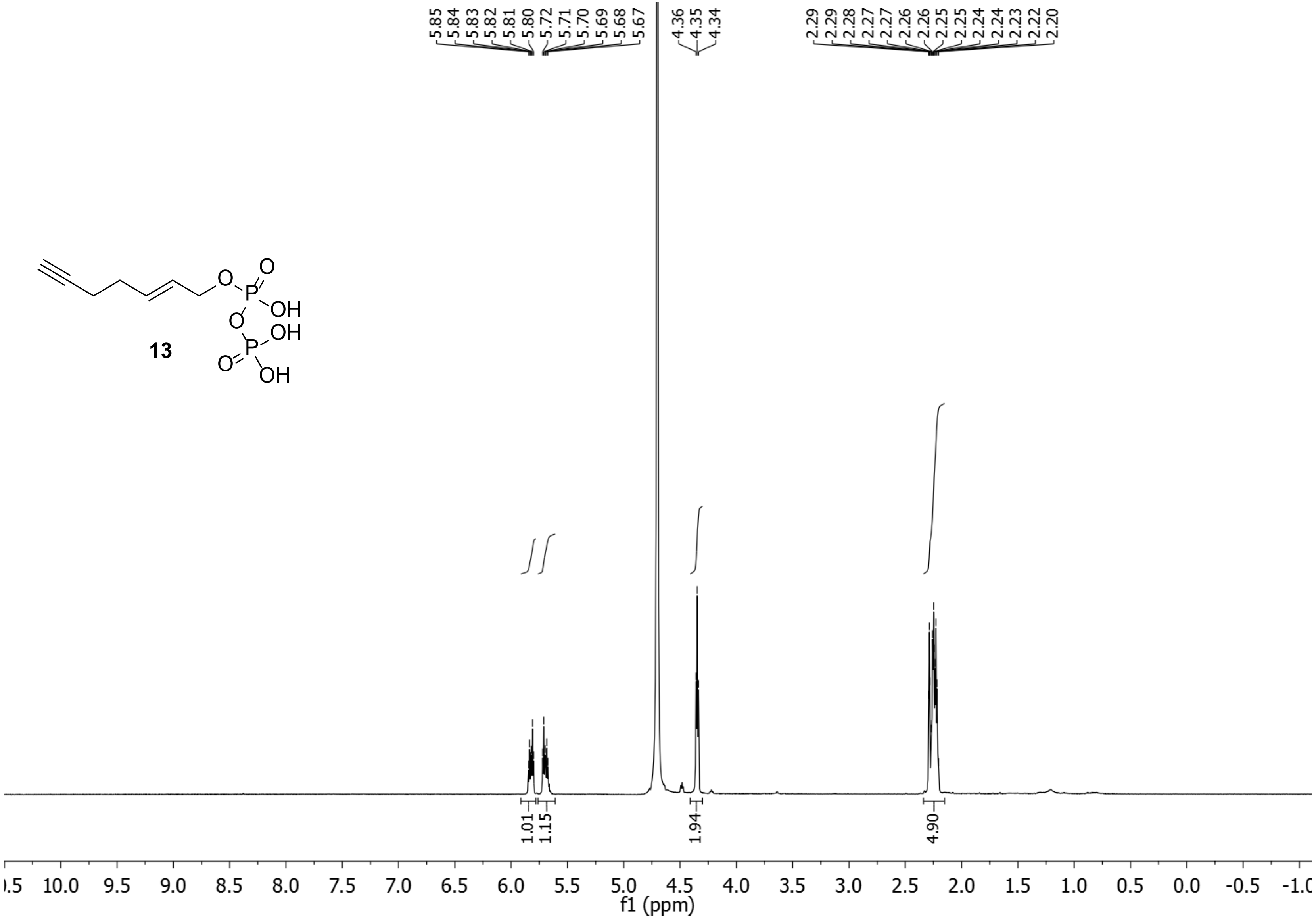

Figure S96 ^{1}H NMR (400 MHz) spectrum of (*E*)-hept-2-en-6-yn-1-yl trihydrogen diphosphate (**13**) in D_2O.

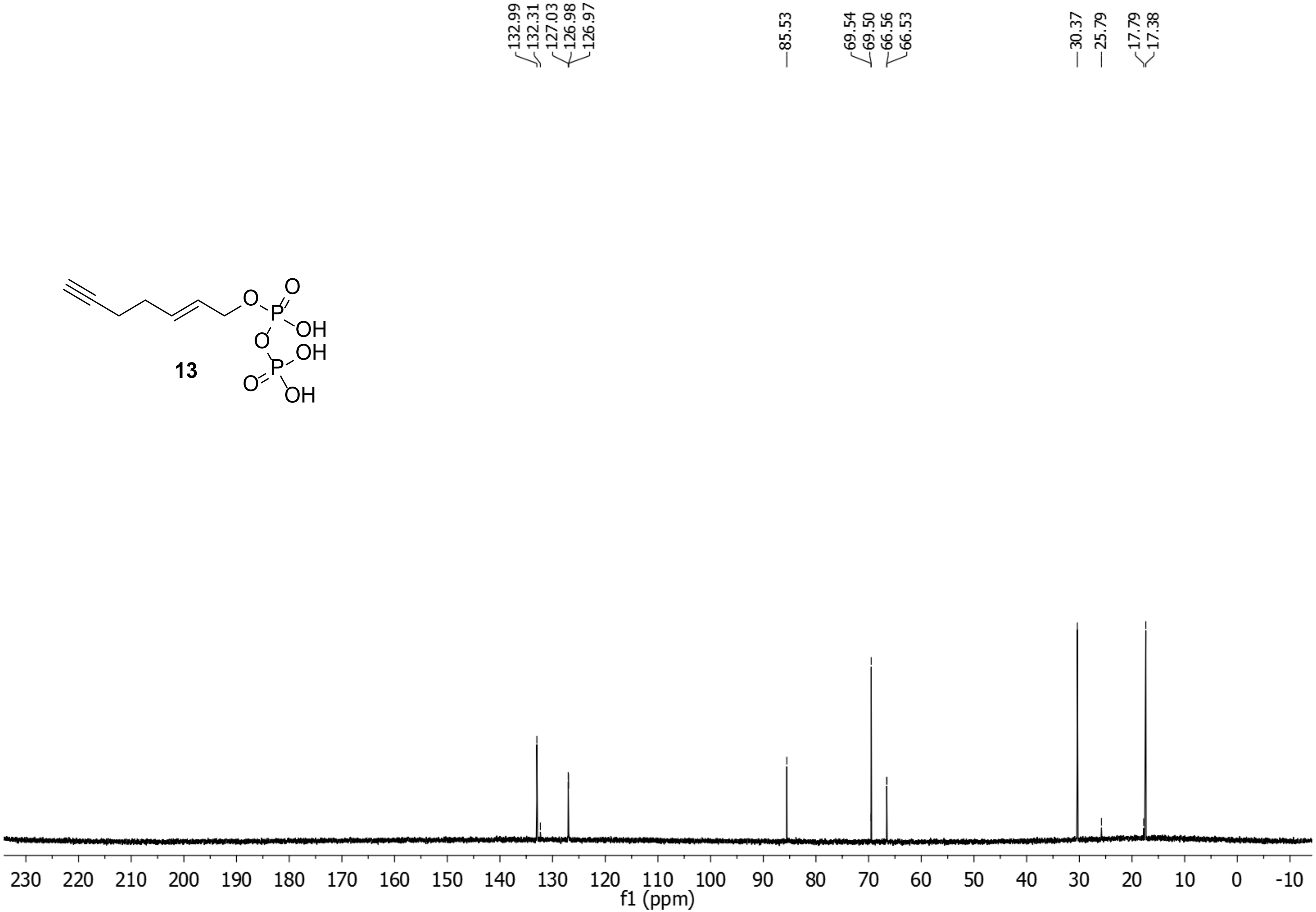

Figure S97 ^{13}C NMR (101 MHz) spectrum of (*E*)-hept-2-en-6-yn-1-yl trihydrogen diphosphate (**13**) in D_2O.

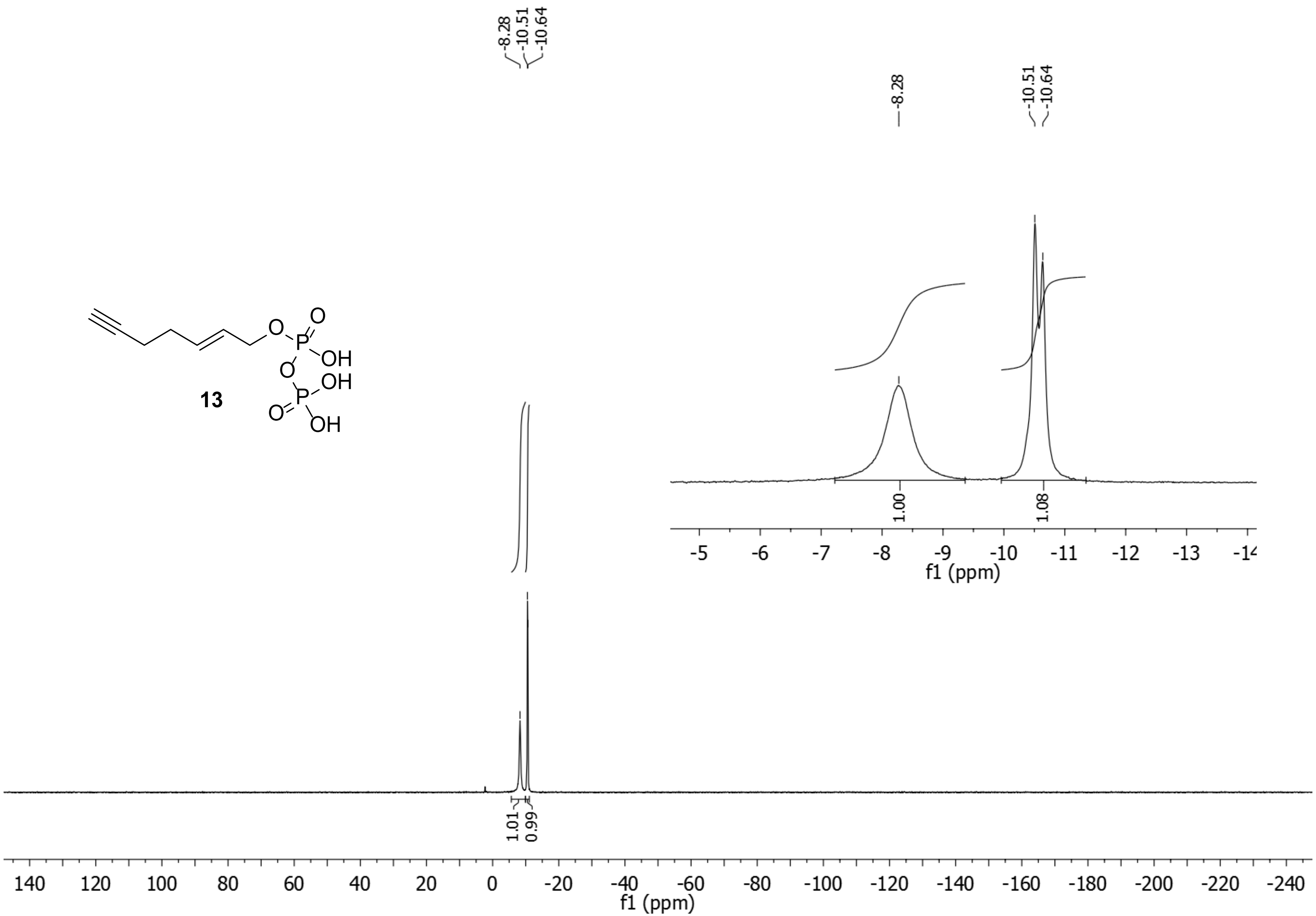

Figure S98 ^{31}P NMR (162 MHz) spectrum of (*E*)-hept-2-en-6-yn-1-yl trihydrogen diphosphate (**13**) in D_2O.

`

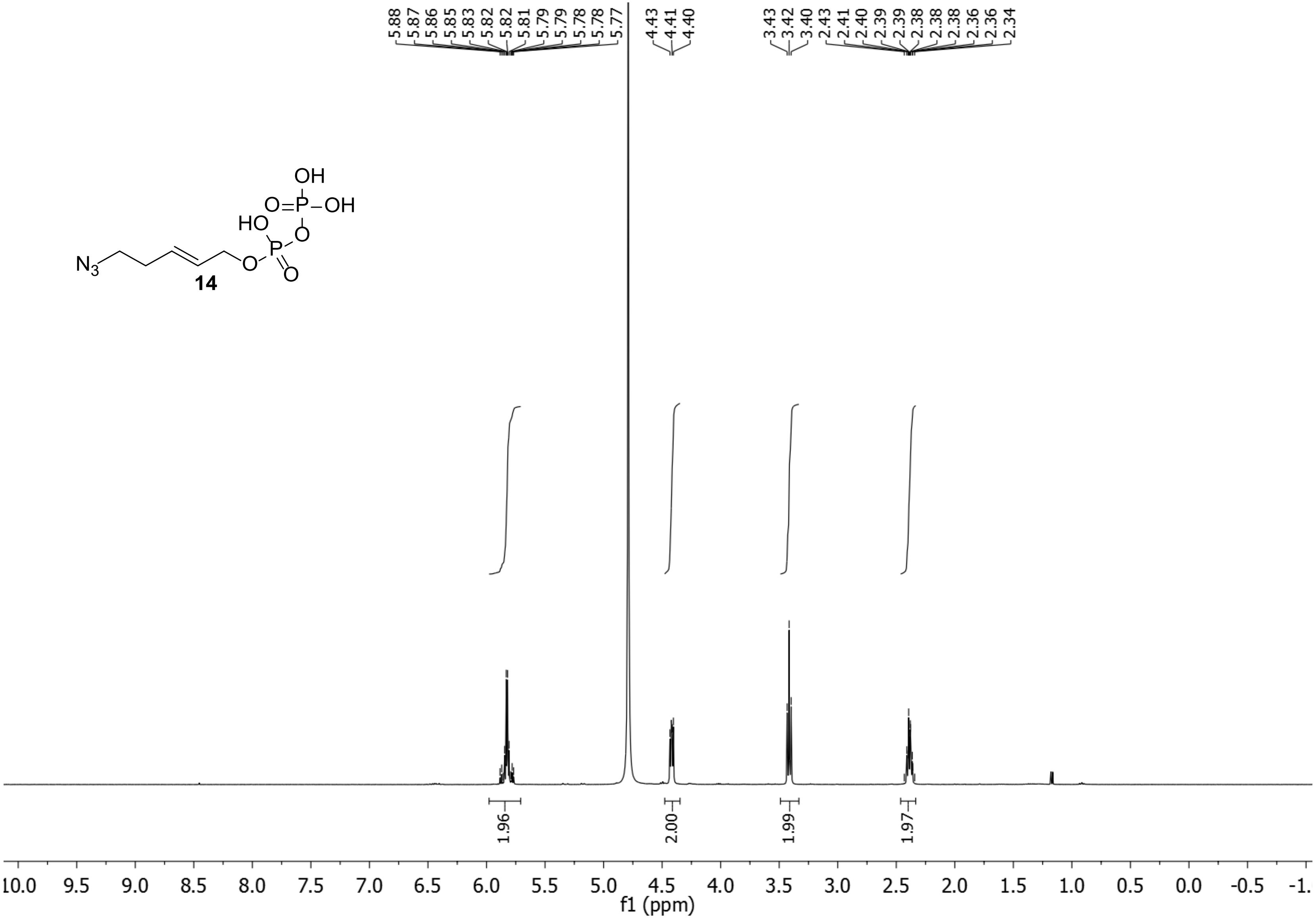

Figure S99 ^{1}H NMR (400 MHz) spectrum of ***(E)*-5-azidopent-2-en-1-yl trihydrogen diphosphate** (**14**) in D_2O.

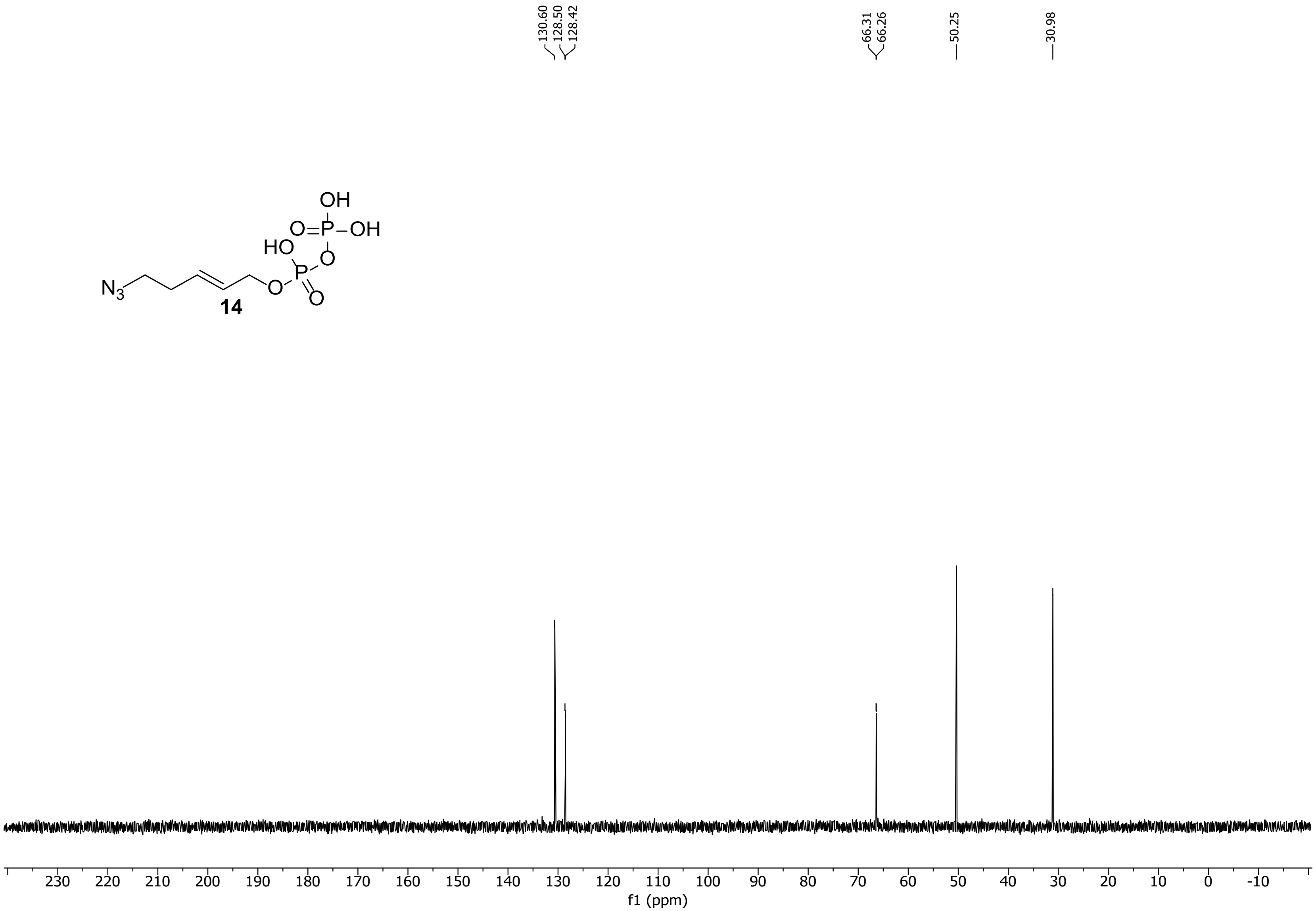

Figure S100 ^{13}C NMR (101 MHz) spectrum of **(*E*)-5-azidopent-2-en-1-yl trihydrogen diphosphate** (**14**) in D_2O.

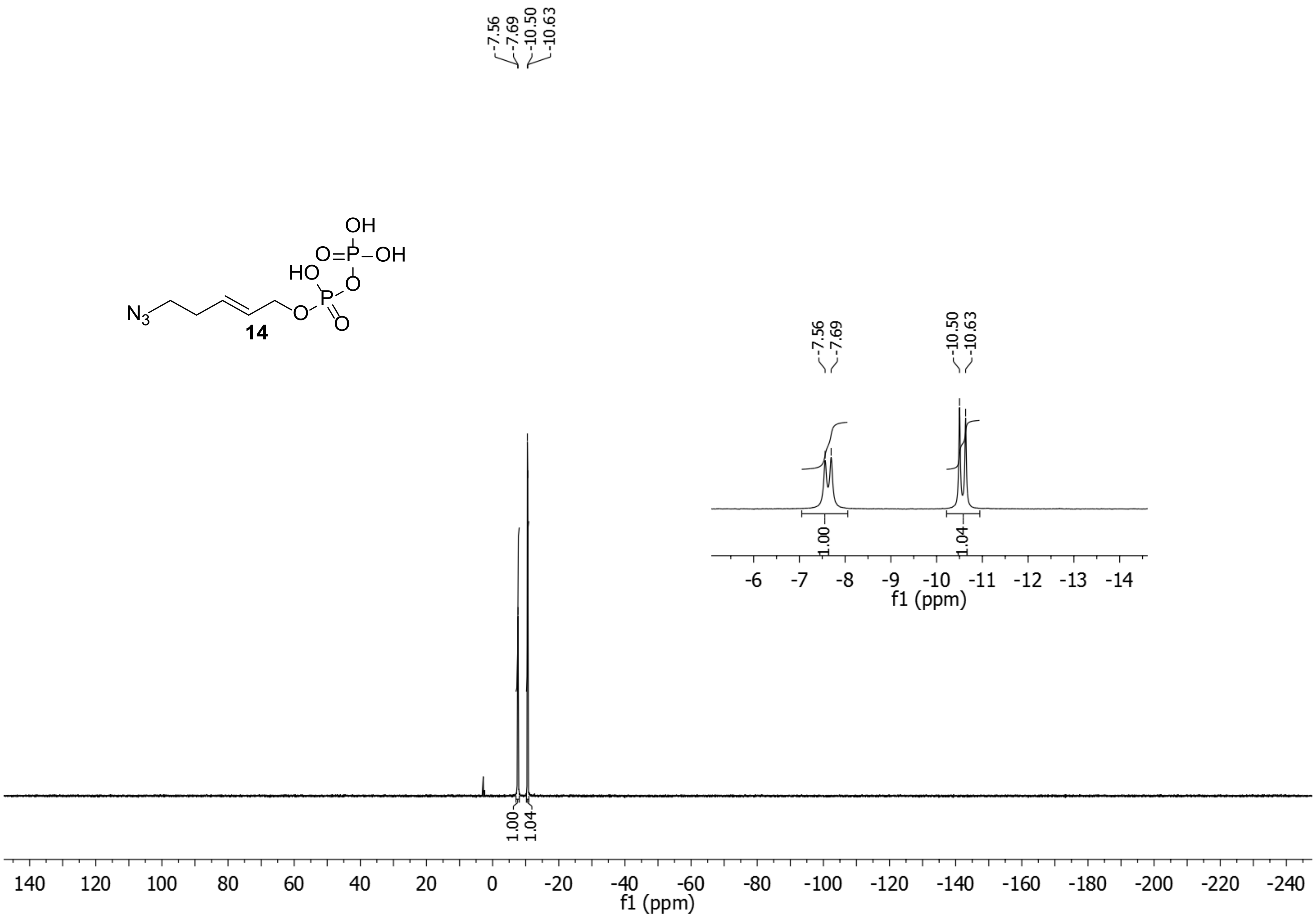

Figure S101 ^{31}P NMR (162 MHz) spectrum of **(*E*)-5-azidopent-2-en-1-yl trihydrogen diphosphate** (**14**) in D_2O.

`

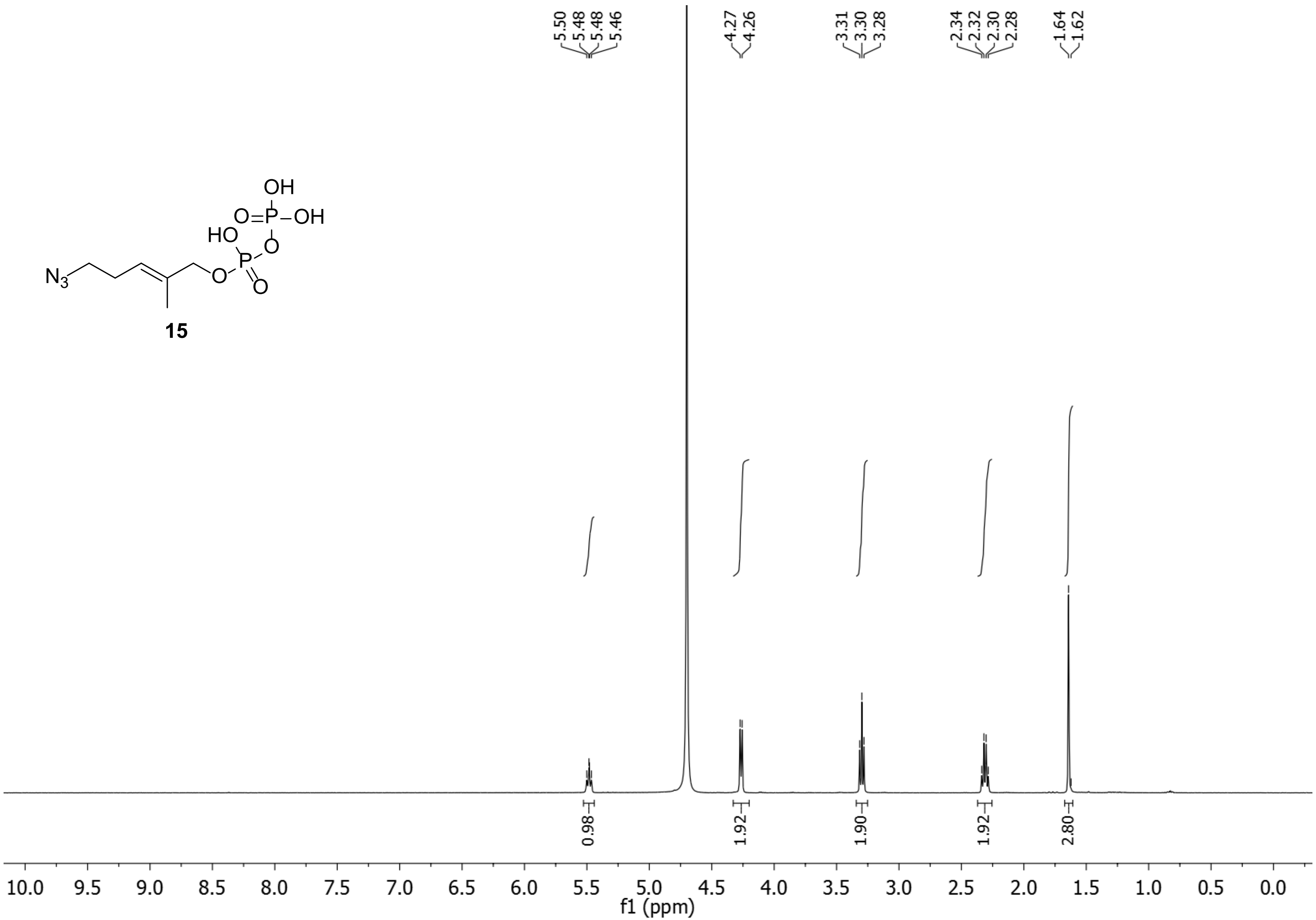

Figure S102 ^{1}H NMR (400 MHz) spectrum of (*E*)-5-azido-2-methylpent-2-en-1-yl trihydrogen diphosphate (**15**) in D_2O.

`

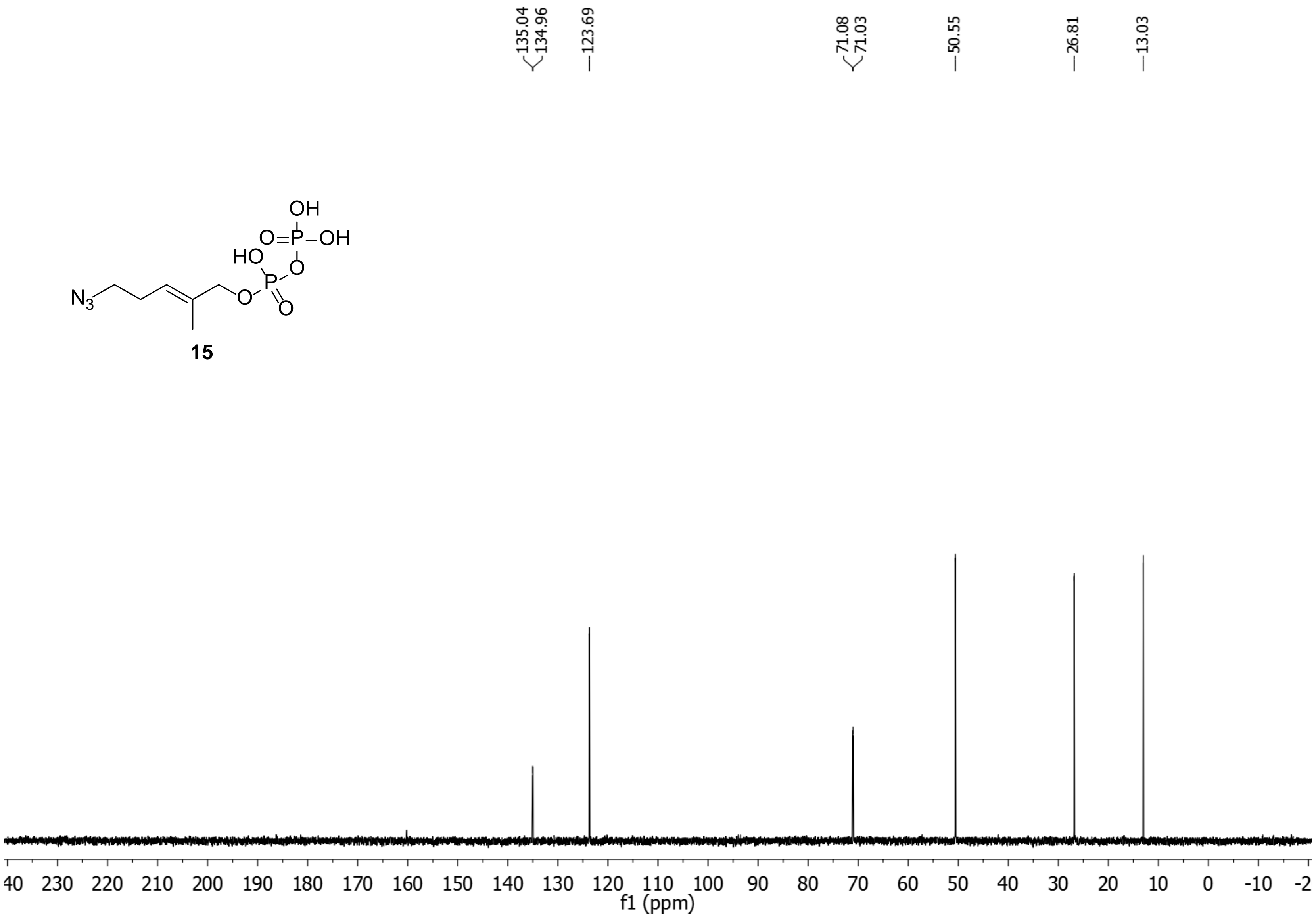

Figure S103 ^{13}C NMR (101 MHz) spectrum of (*E*)-5-azido-2-methylpent-2-en-1-yl trihydrogen diphosphate (**15**) in D_2O.

`

15

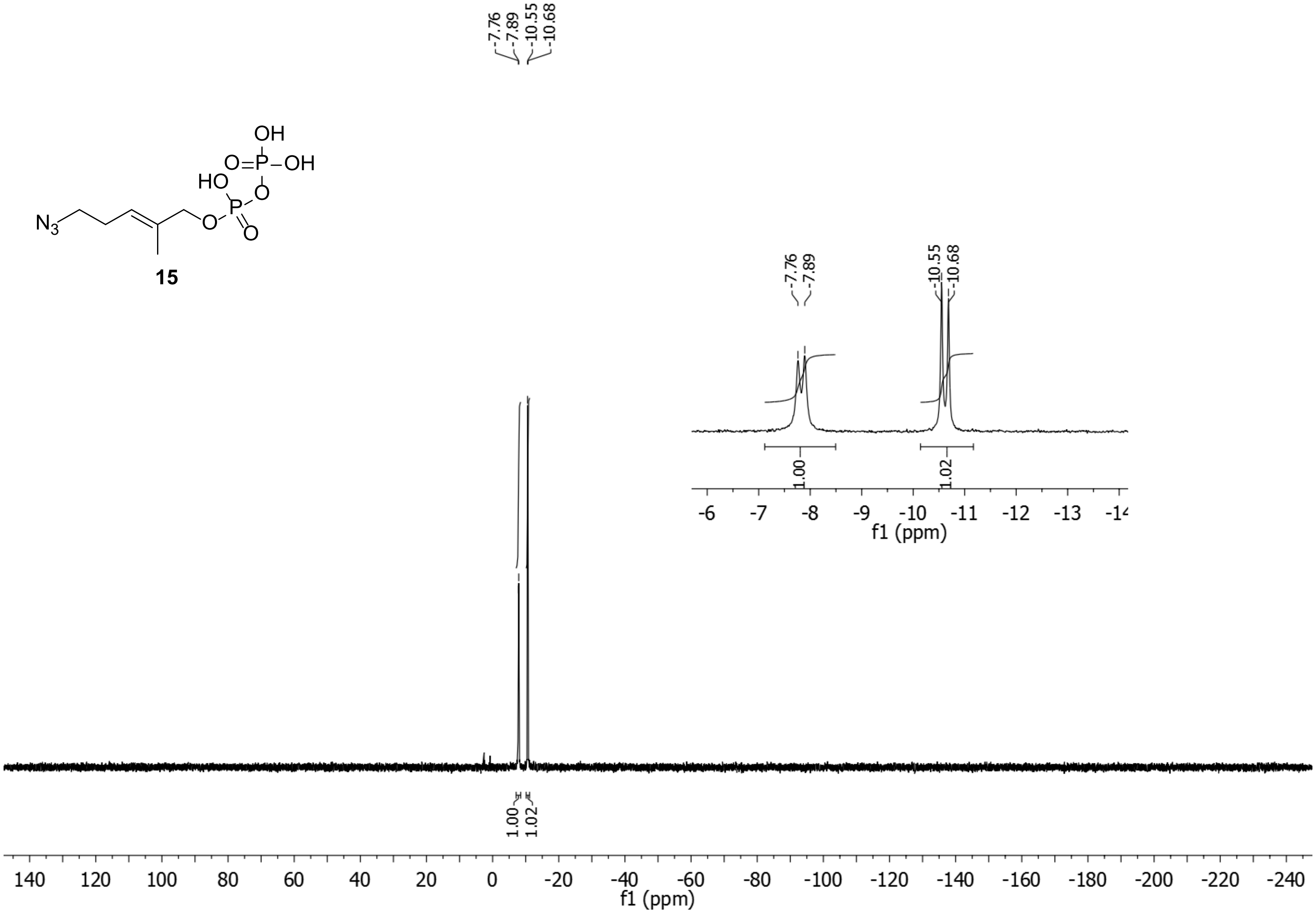

Figure S104 ^{31}P NMR (162 MHz) spectrum of (*E*)-5-azido-2-methylpent-2-en-1-yl trihydrogen diphosphate (**15**) in D_2O.

`

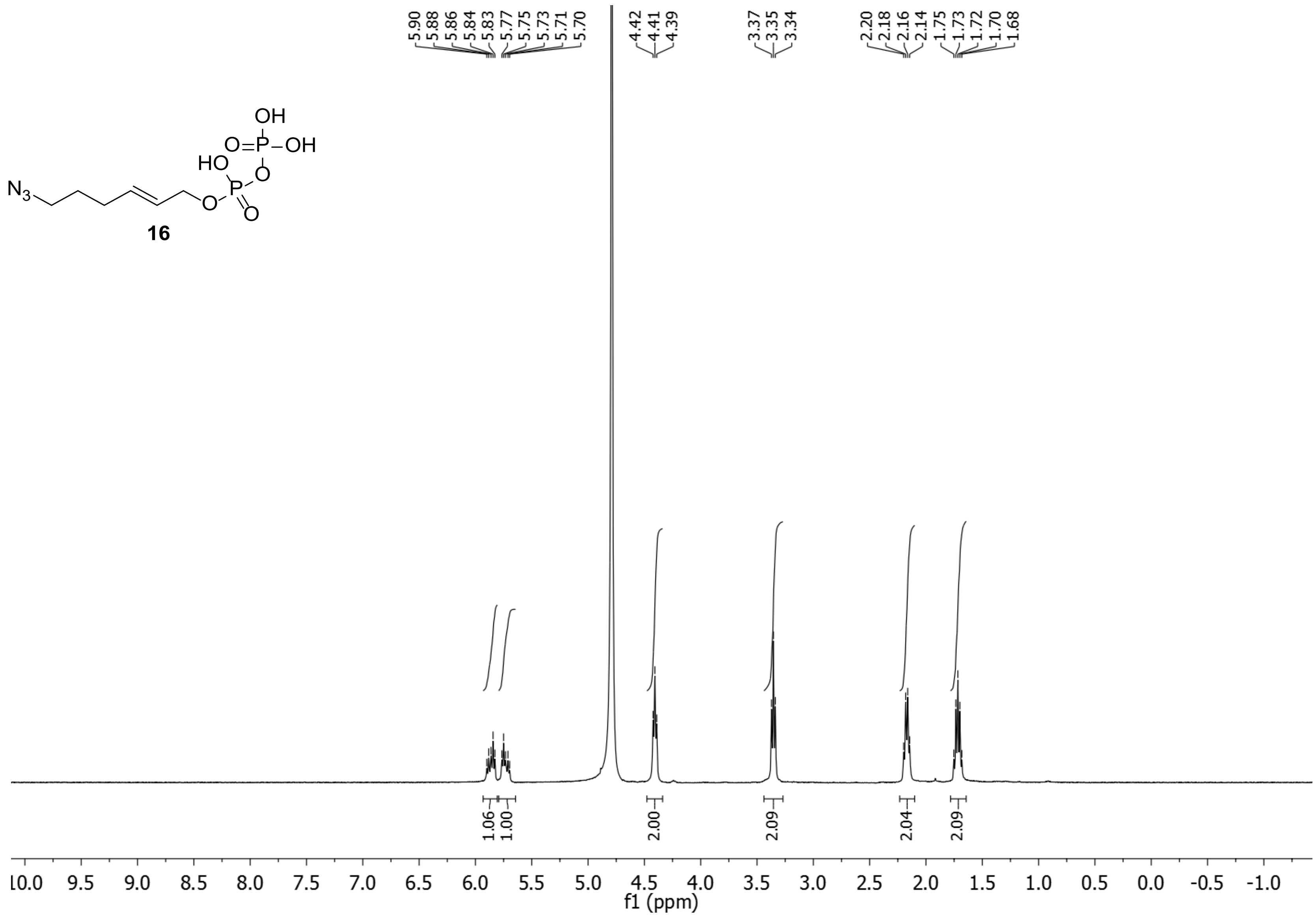

Figure S105 ^{1}H NMR (400 MHz) spectrum of (*E*)-6-azidohex-2-en-1-yl trihydrogen diphosphate (**16**) in D_2O.

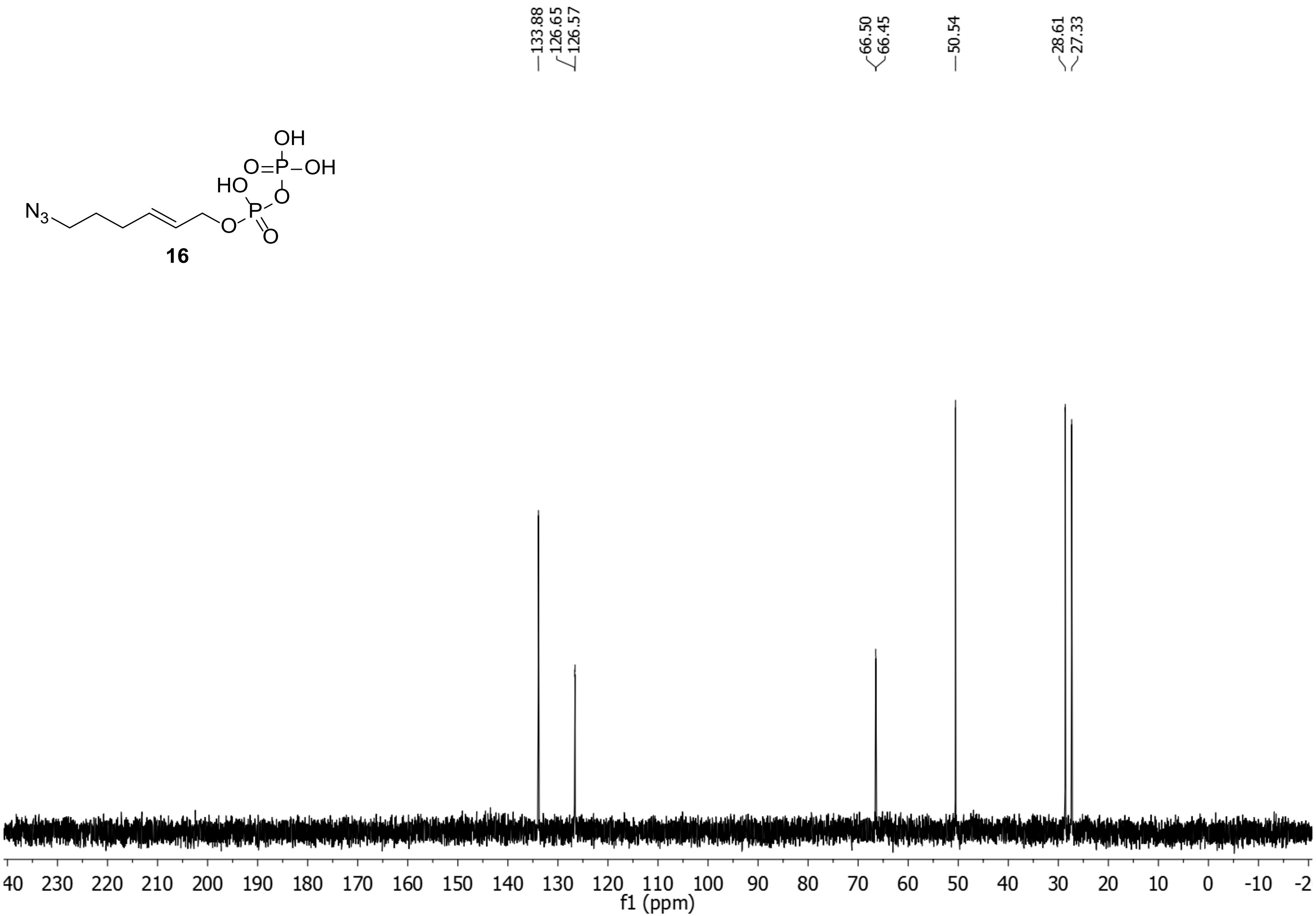

Figure S106 ^{13}C NMR (101 MHz) spectrum of (*E*)-6-azidohex-2-en-1-yl trihydrogen diphosphate (**16**) in D_2O.

`

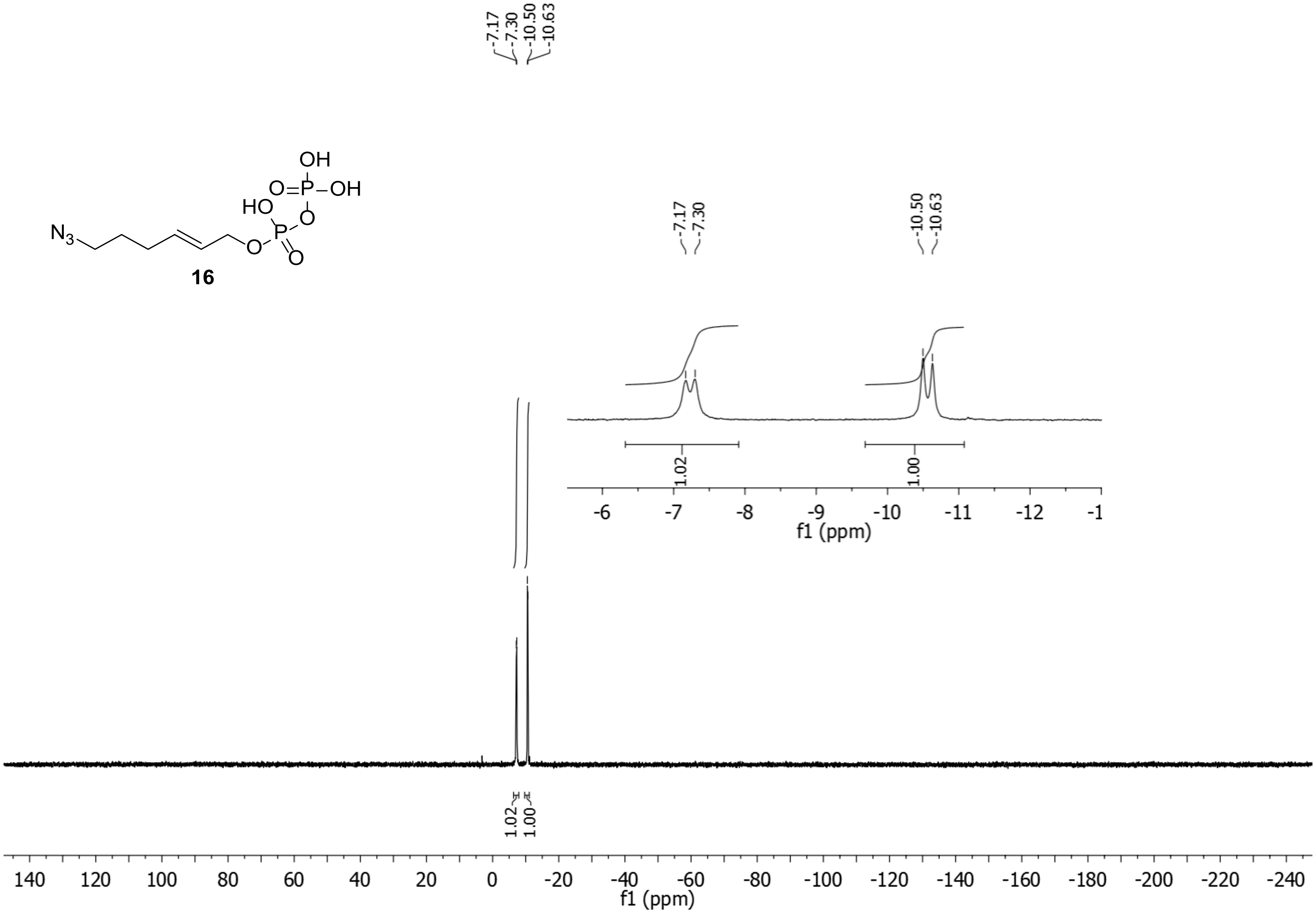

Figure S107 ^{31}P NMR (162 MHz) spectrum of (*E*)-6-azidohex-2-en-1-yl trihydrogen diphosphate (**16**) in D_2O.

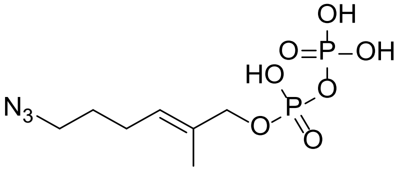

17

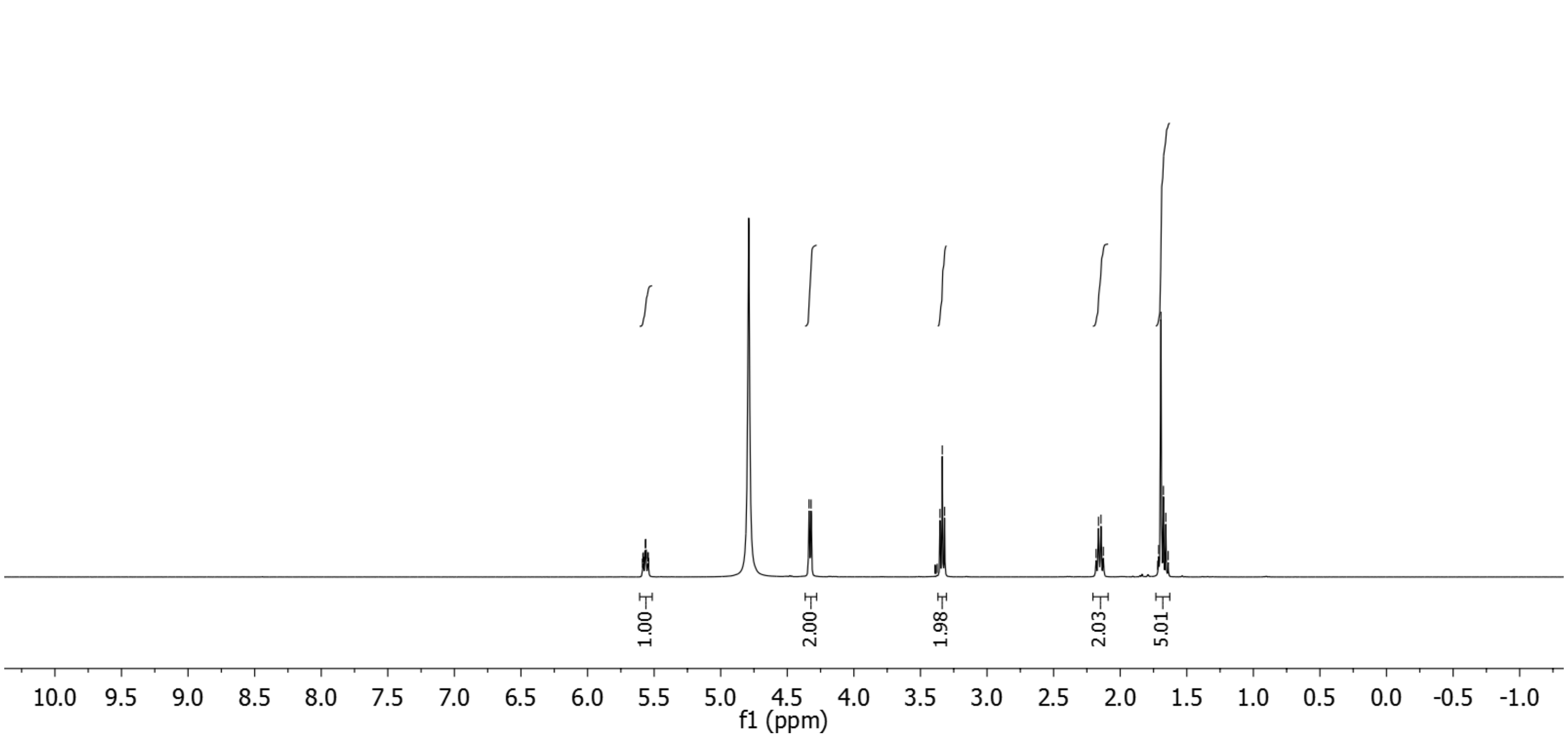

Figure S108 ^{1}H NMR (400 MHz) spectrum of (*E*)-6-azido-2-methylhex-2-en-1-yl trihydrogen diphosphate (**17**) in D_2O.

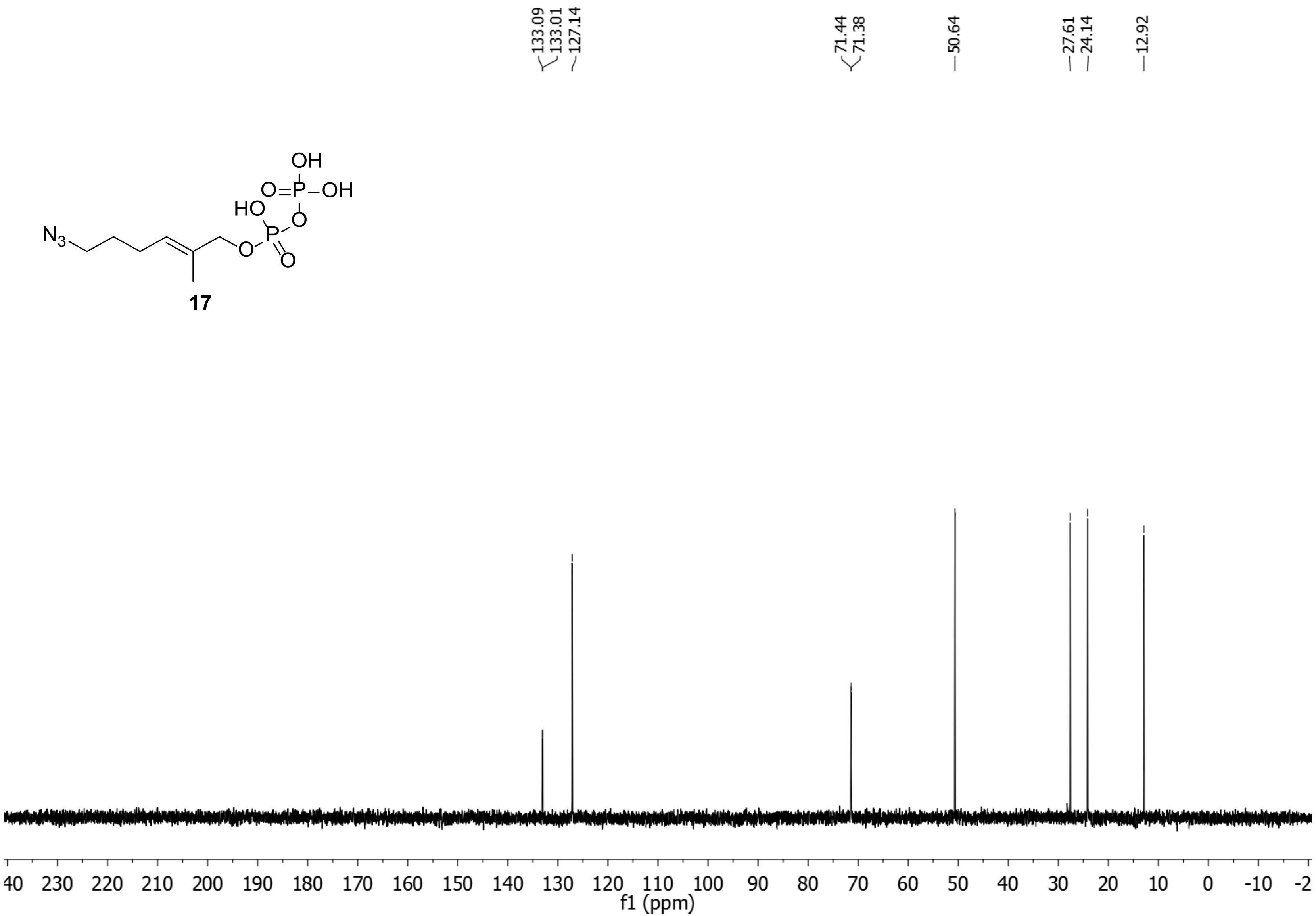

Figure S109 ^{13}C NMR (101 MHz) spectrum of (*E*)-6-azido-2-methylhex-2-en-1-yl trihydrogen diphosphate (**17**) in D_2O.

`

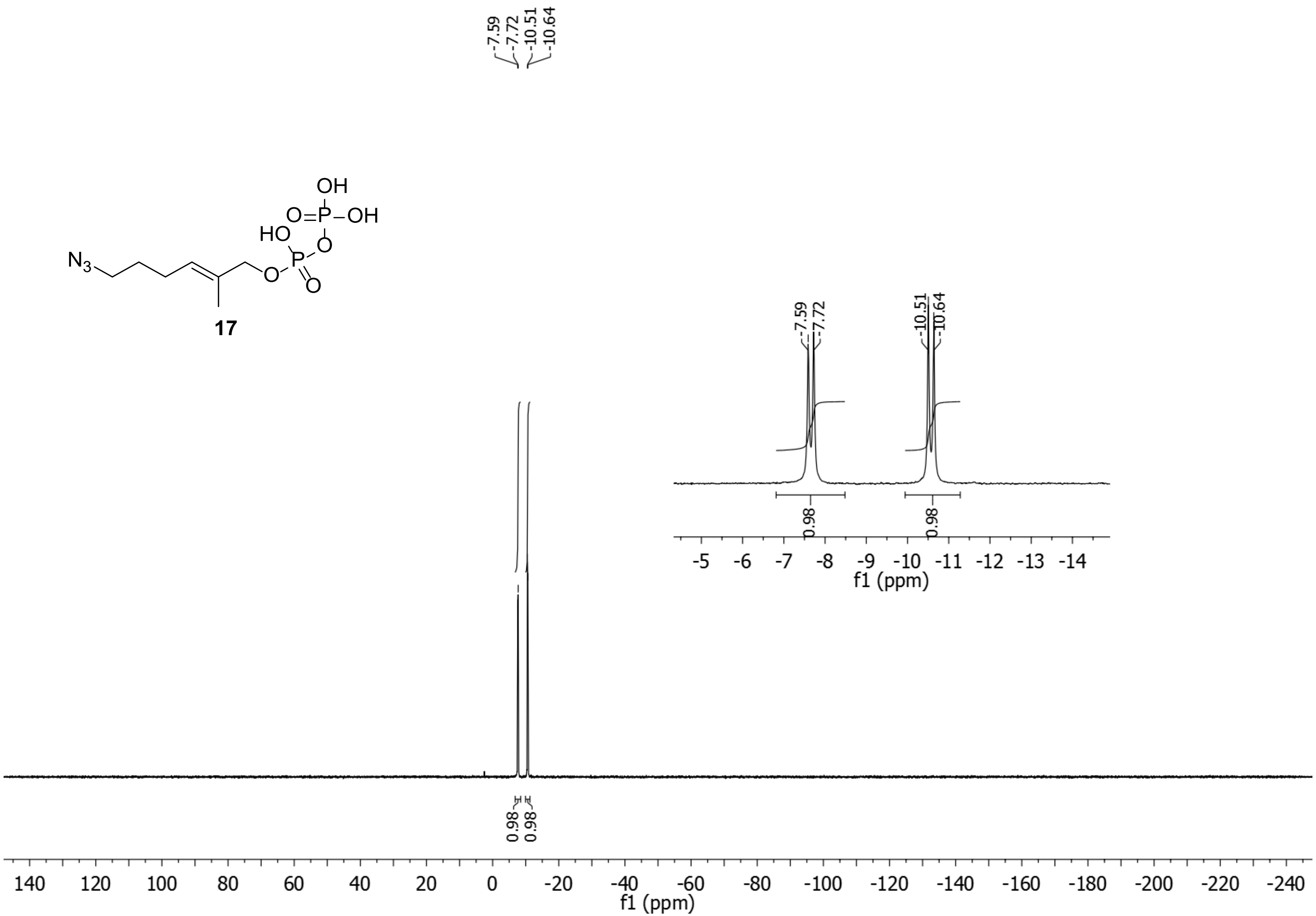

Figure S110 ^{31}P NMR (162 MHz) spectrum of (*E*)-6-azido-2-methylhex-2-en-1-yl trihydrogen diphosphate (**17**) in D_2O.

`

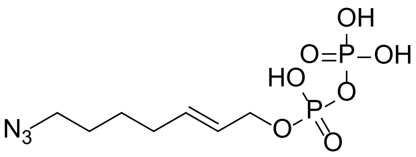

18

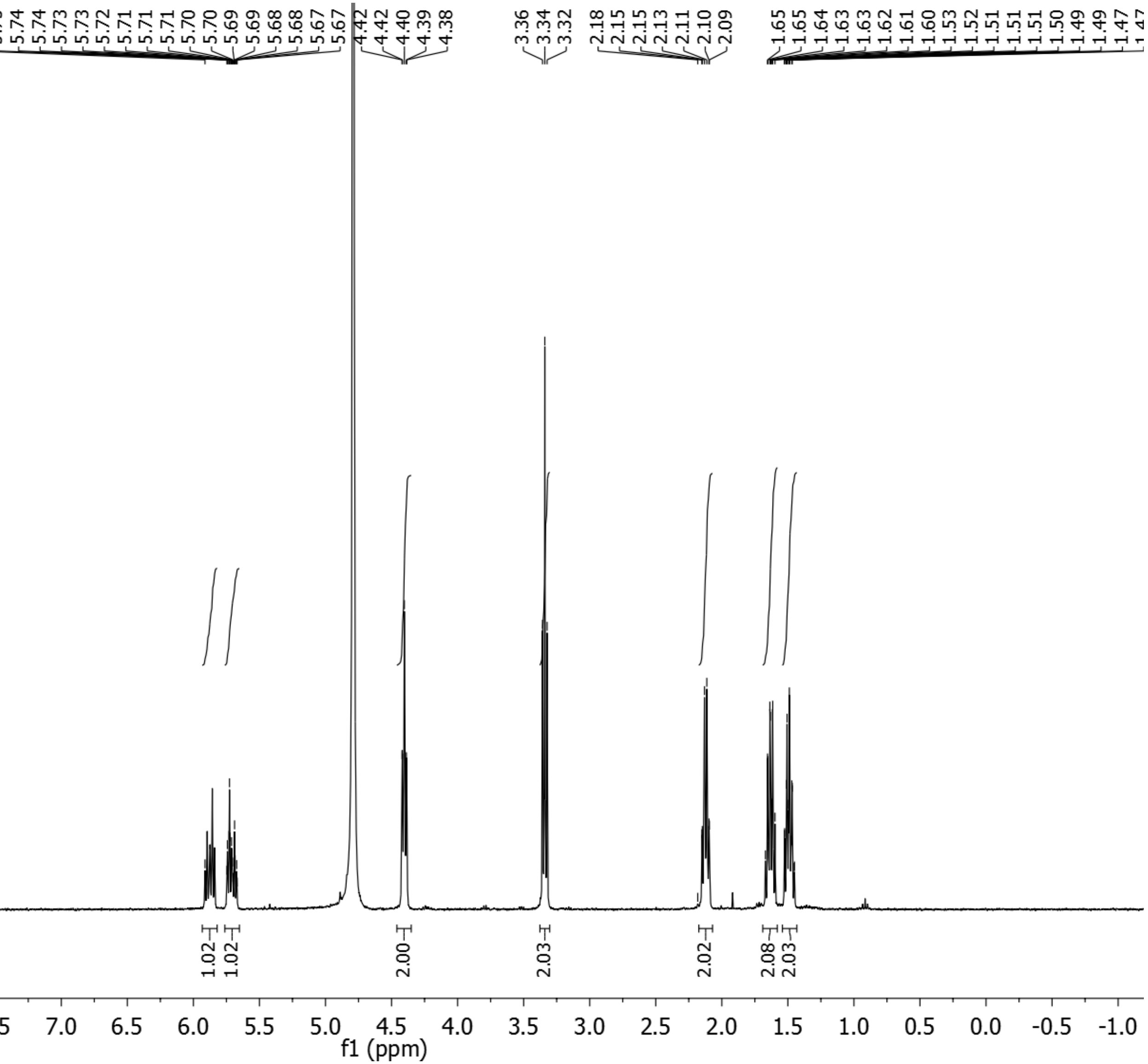

Figure S111 ^{1}H NMR (400 MHz) spectrum of (*E*)-7-azidohept-2-en-1-yl trihydrogen diphosphate (**18**) in D_2O.

`

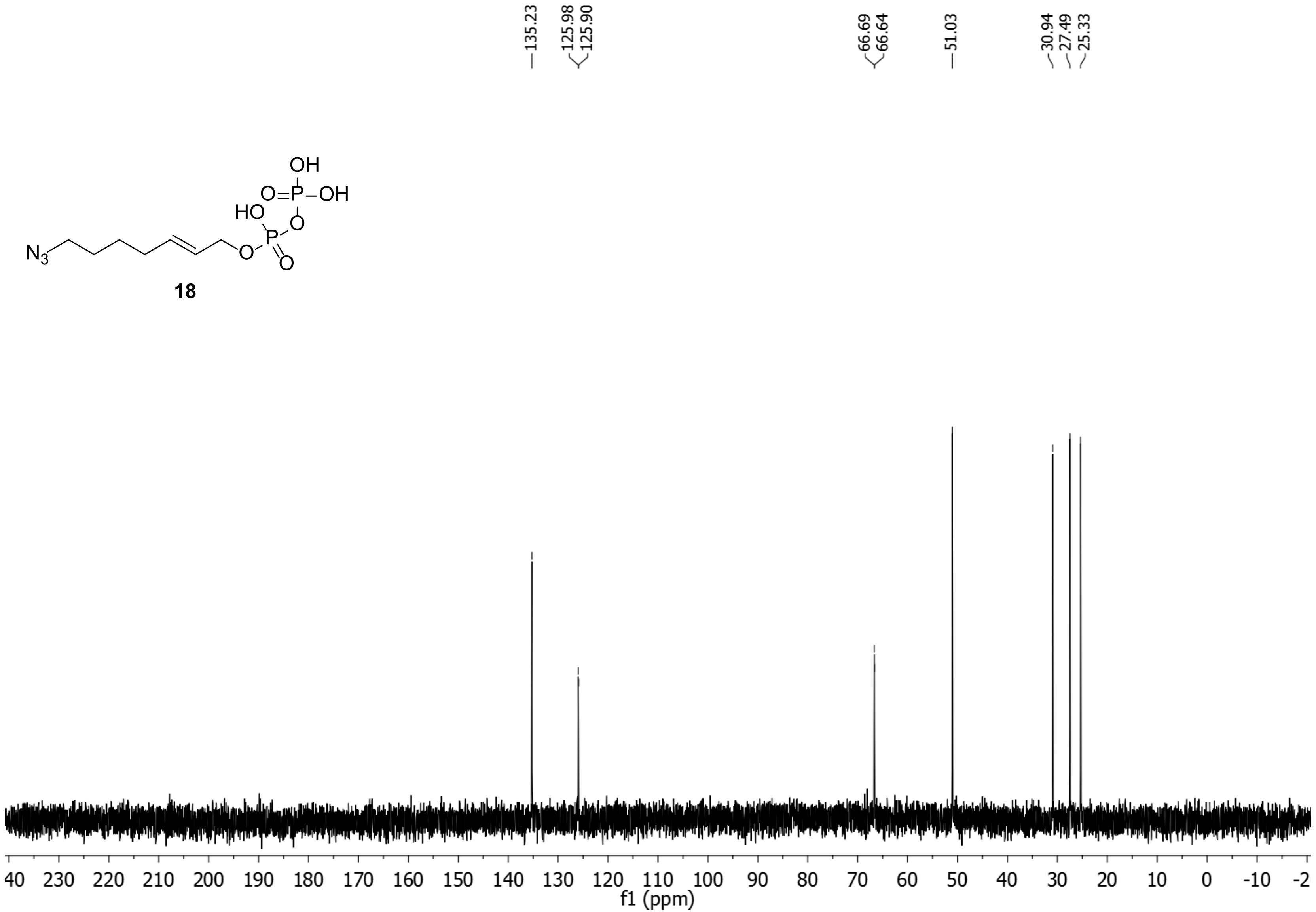

18

Figure S112 ^{13}C NMR (101 MHz) spectrum of (*E*)-7-azidohept-2-en-1-yl trihydrogen diphosphate (**18**) in D_2O.

`

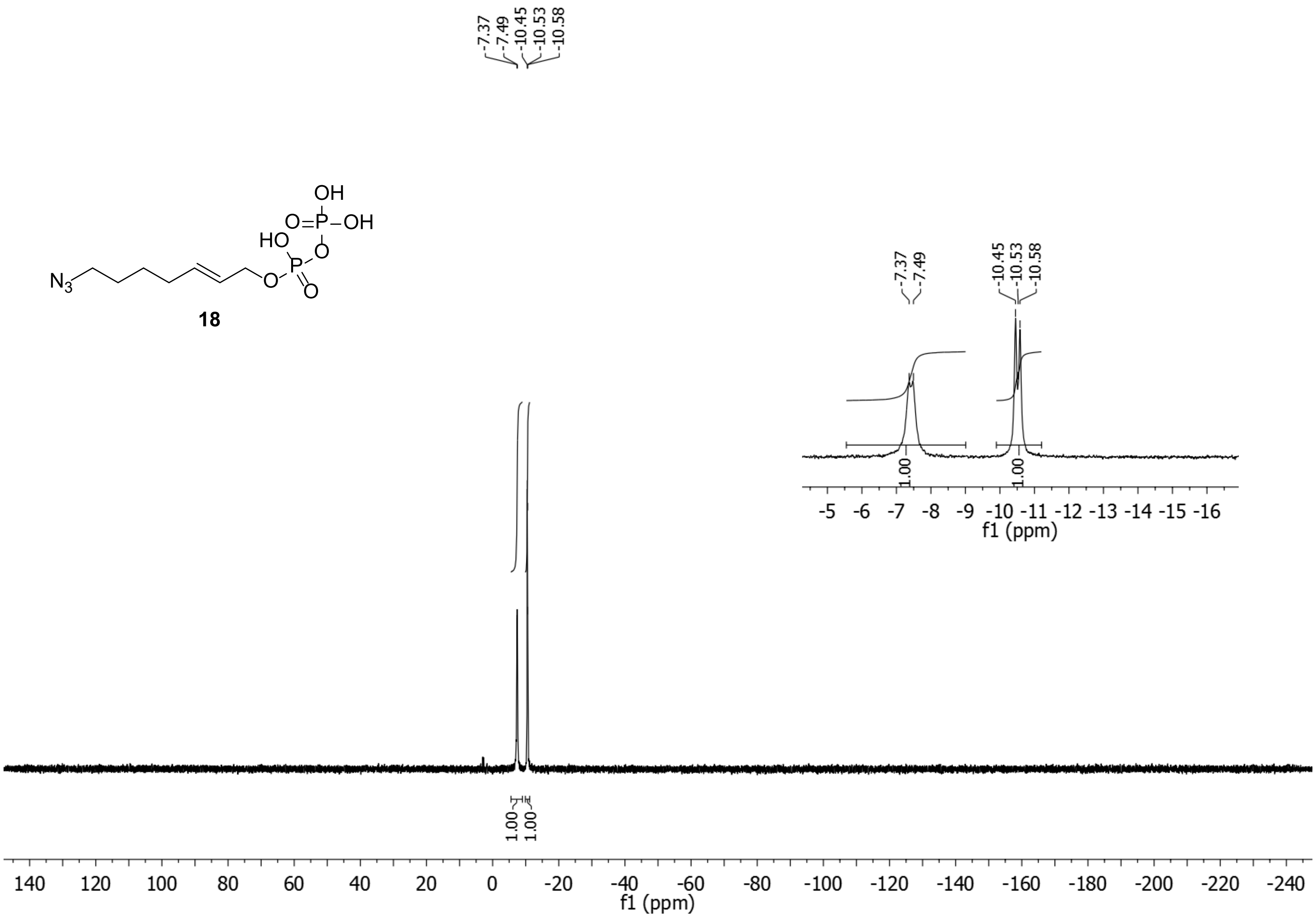

18

Figure S113 ^{31}P NMR (162 MHz) spectrum of (*E*)-7-azidohept-2-en-1-yl trihydrogen diphosphate (**18**) in D_2O.

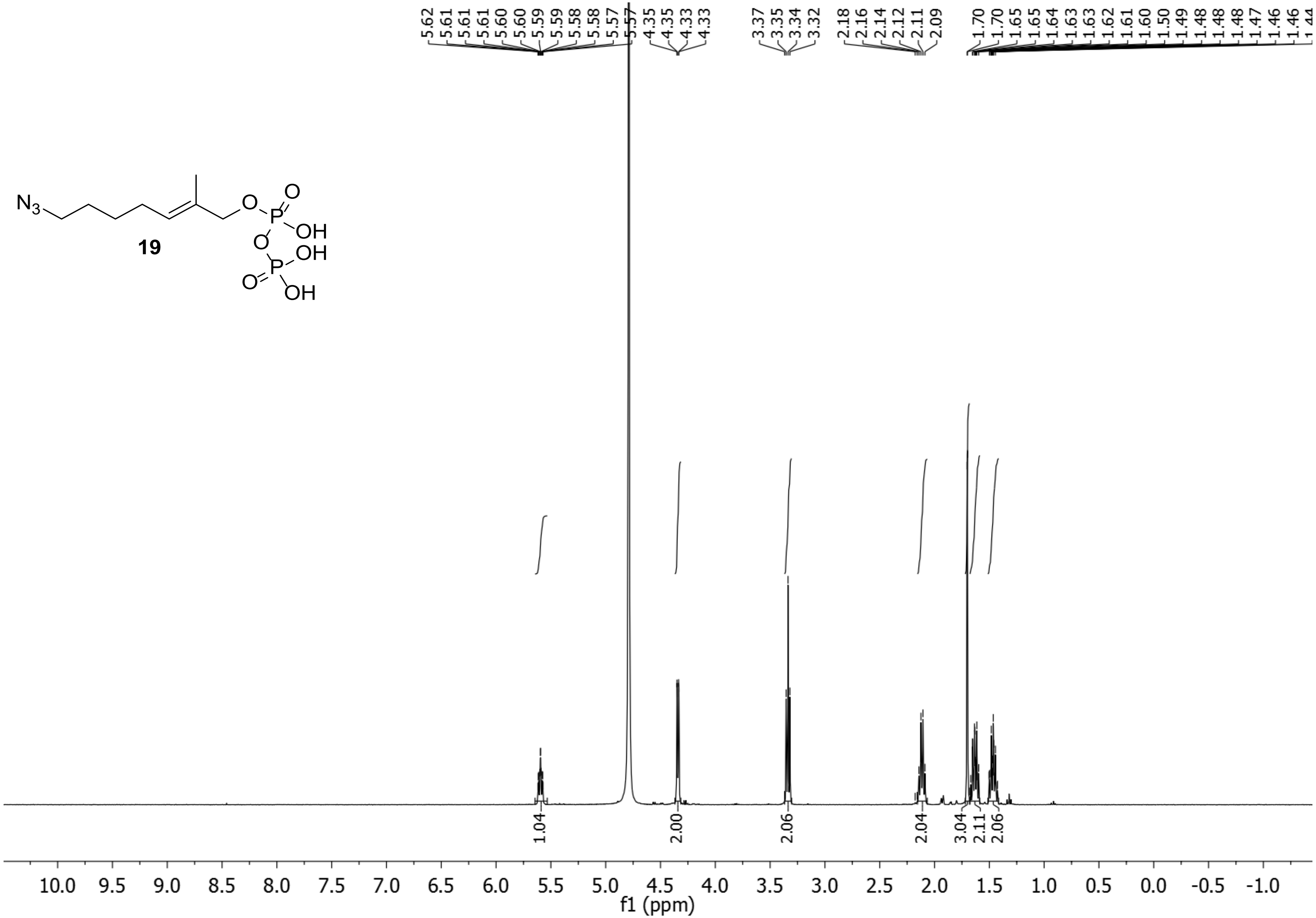

Figure S114 ^{1}H NMR (400 MHz) spectrum of (*E*)-7-azido-2-methylhept-2-en-1-yl trihydrogen diphosphate (**19**) in D_2O.

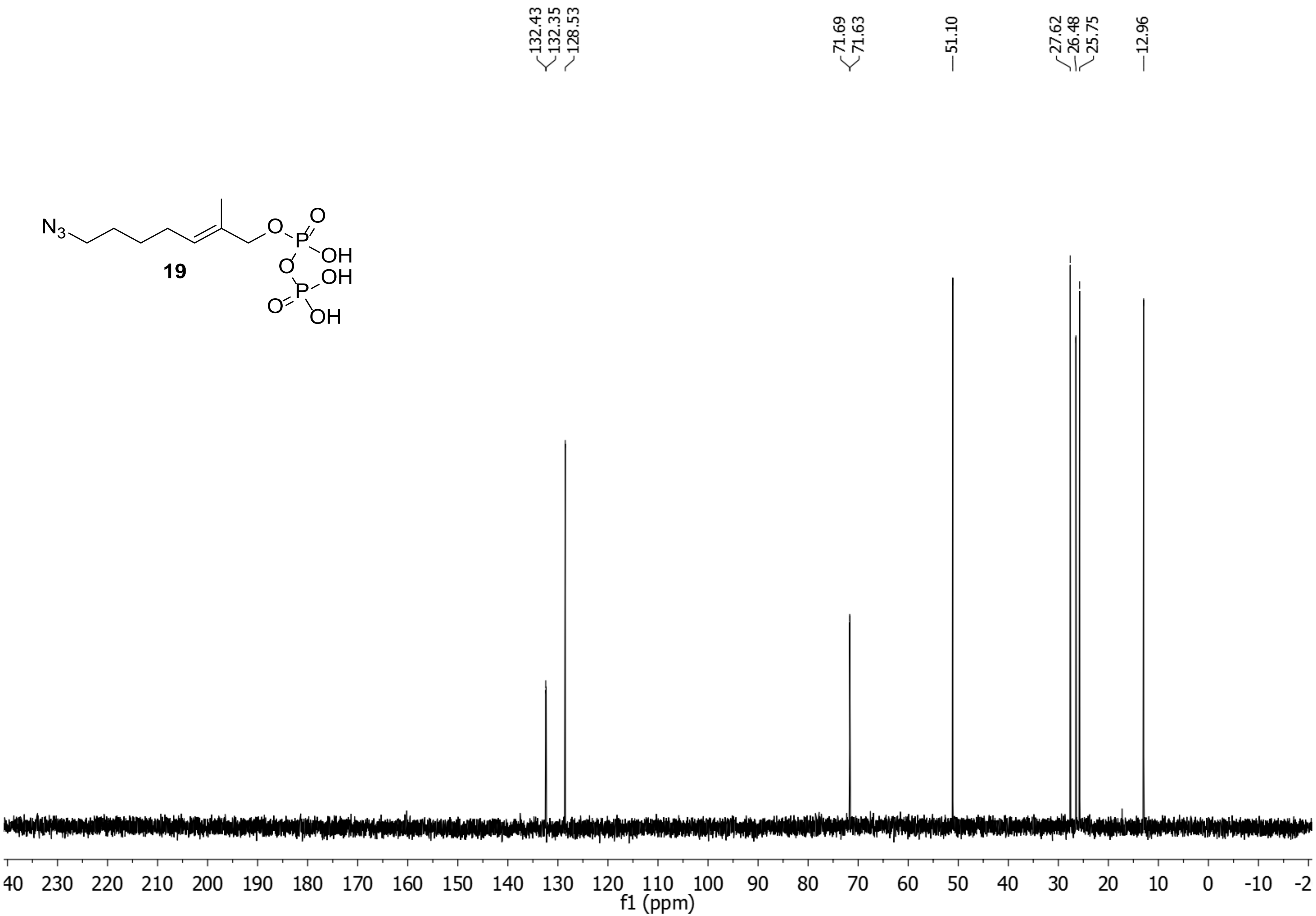

Figure S115 ^{13}C NMR (101 MHz) spectrum of (*E*)-7-azido-2-methylhept-2-en-1-yl trihydrogen diphosphate (**19**) in D_2O.

`

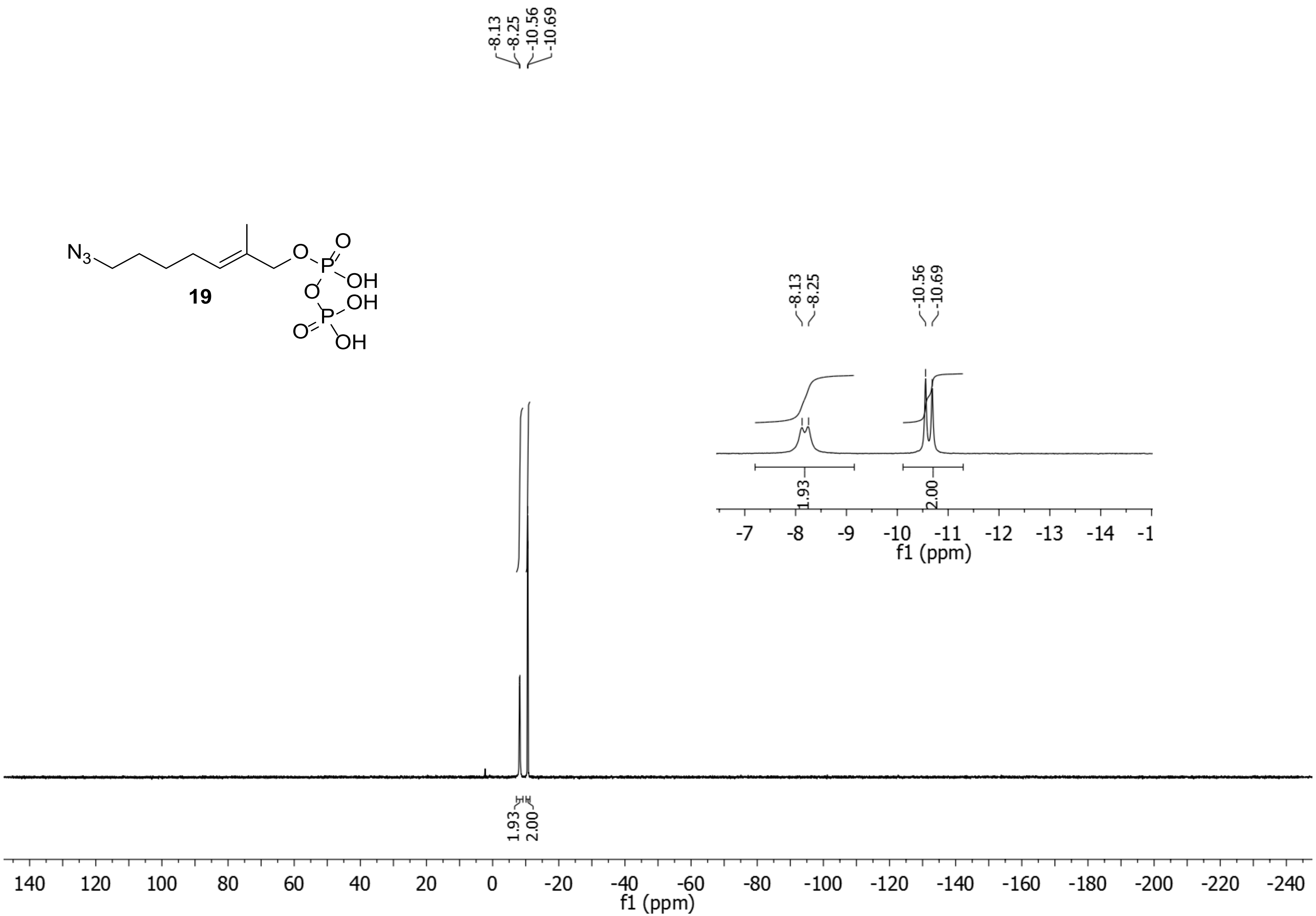

Figure S116 ^{31}P NMR (162 MHz) spectrum of (*E*)-7-azido-2-methylhept-2-en-1-yl trihydrogen diphosphate (**19**) in D_2O.

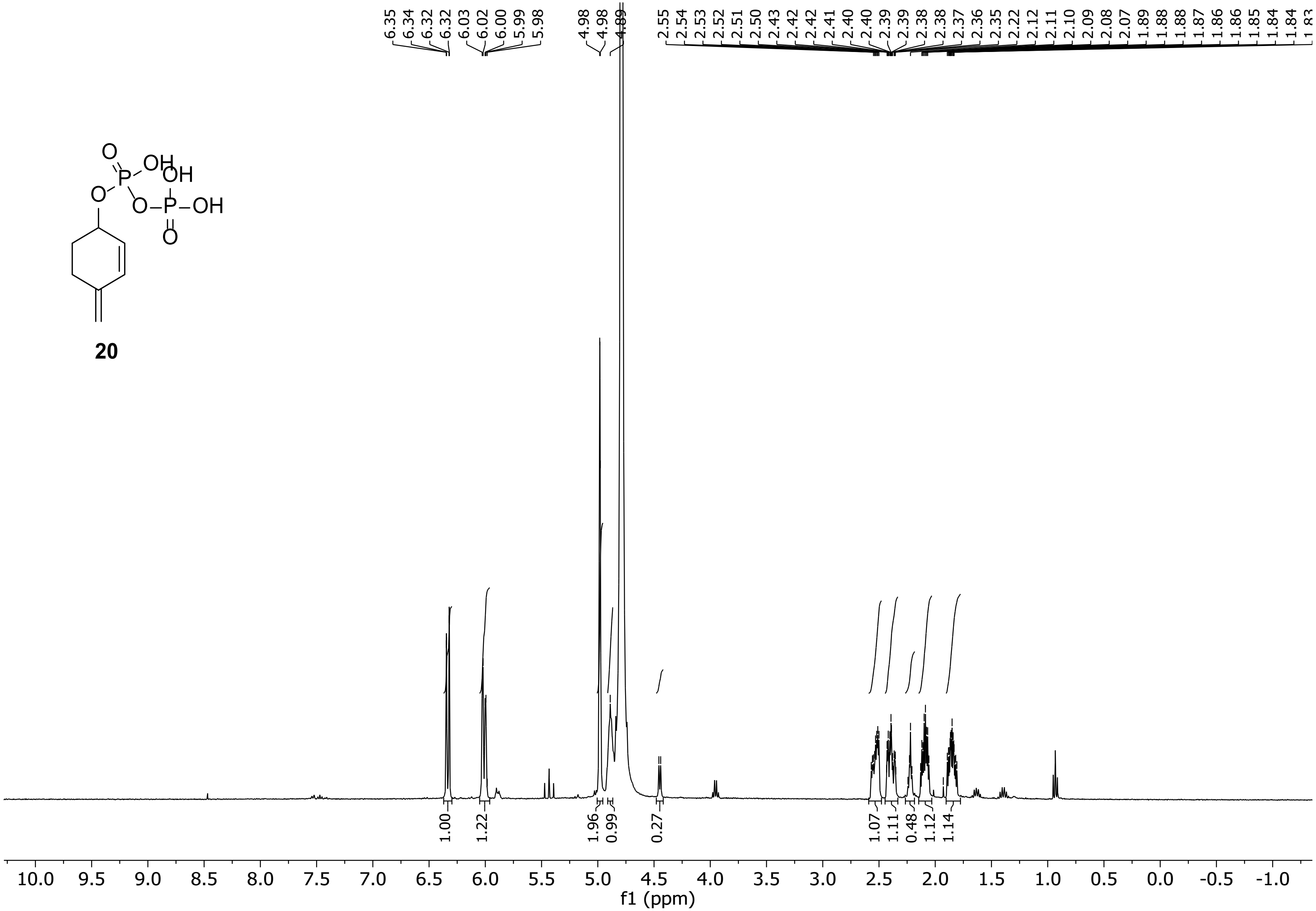

Figure S117 ^{1}H NMR (400 MHz) spectrum of (±)-4-methylenecyclohex-2-en-1-yl trihydrogen diphosphate (**20**) in D_2O.

`

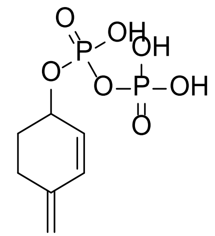

20

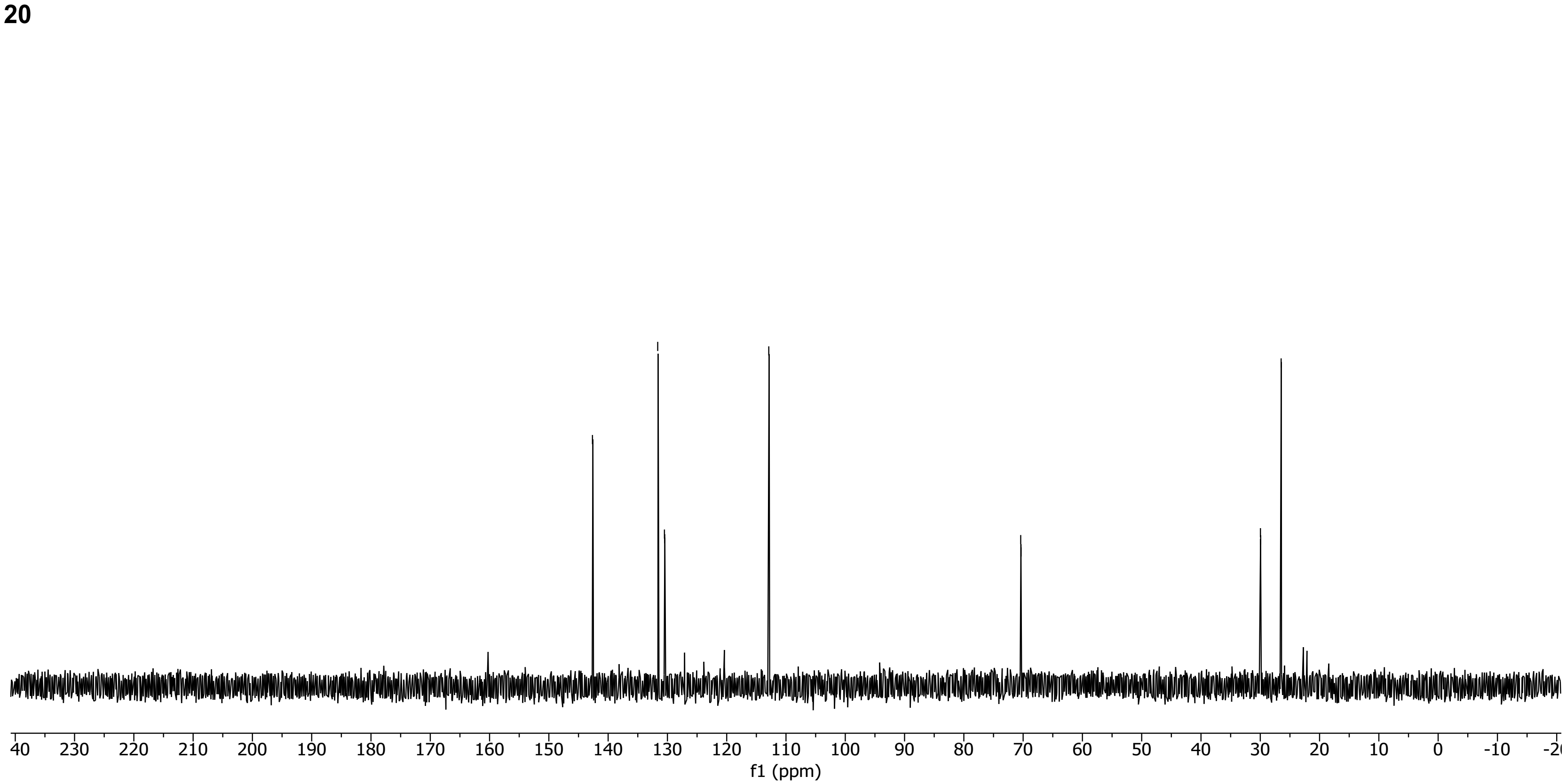

Figure S118 ^{13}C NMR (101 MHz) spectrum of (±)-4-methylenecyclohex-2-en-1-yl trihydrogen diphosphate (**20**) in D_2O.

`

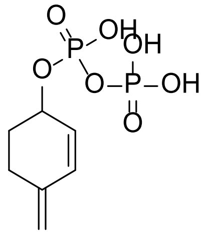

20

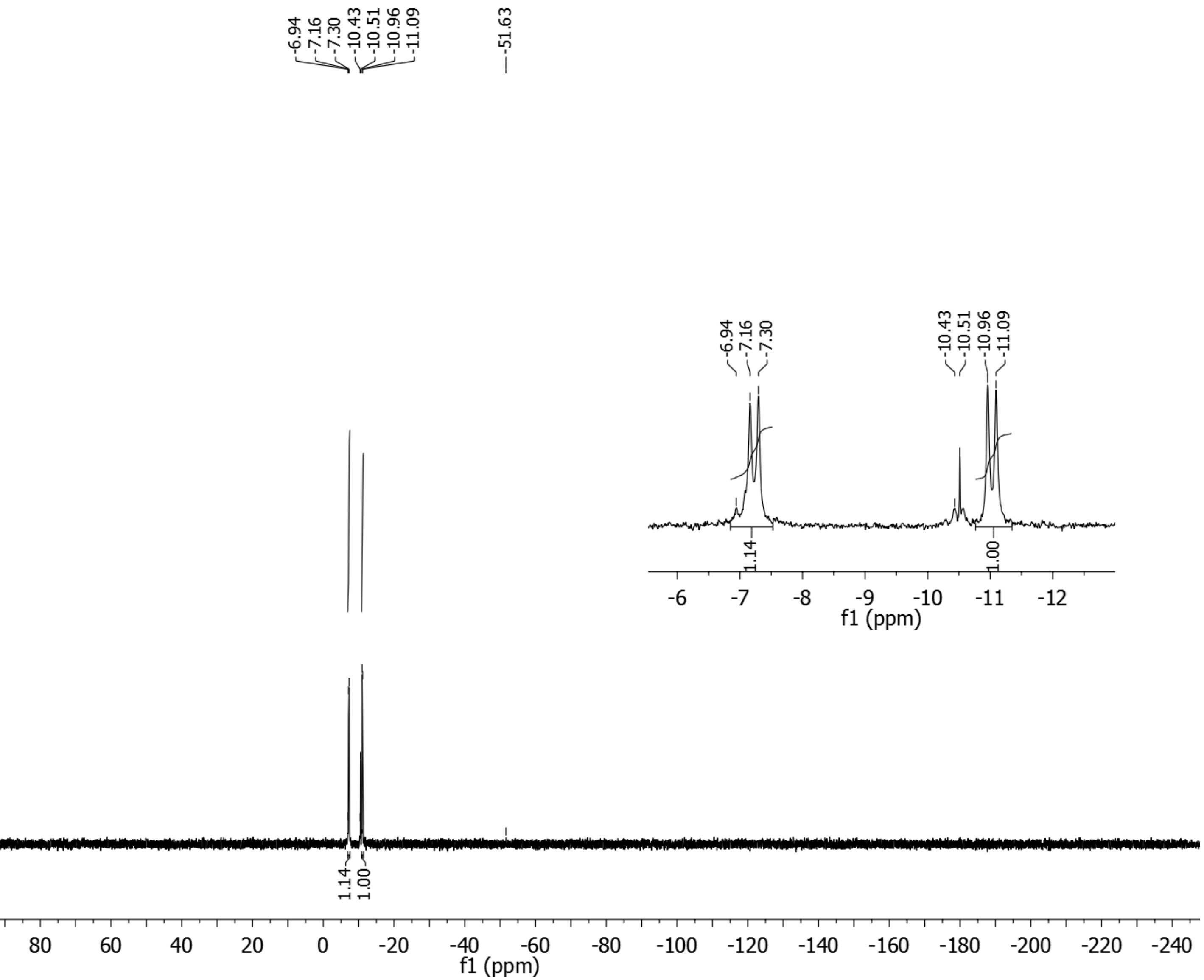

Figure S119 ^{31}P NMR (162 MHz) spectrum of (±)-4-methylenecyclohex-2-en-1-yl trihydrogen diphosphate (**20**) in D_2O.

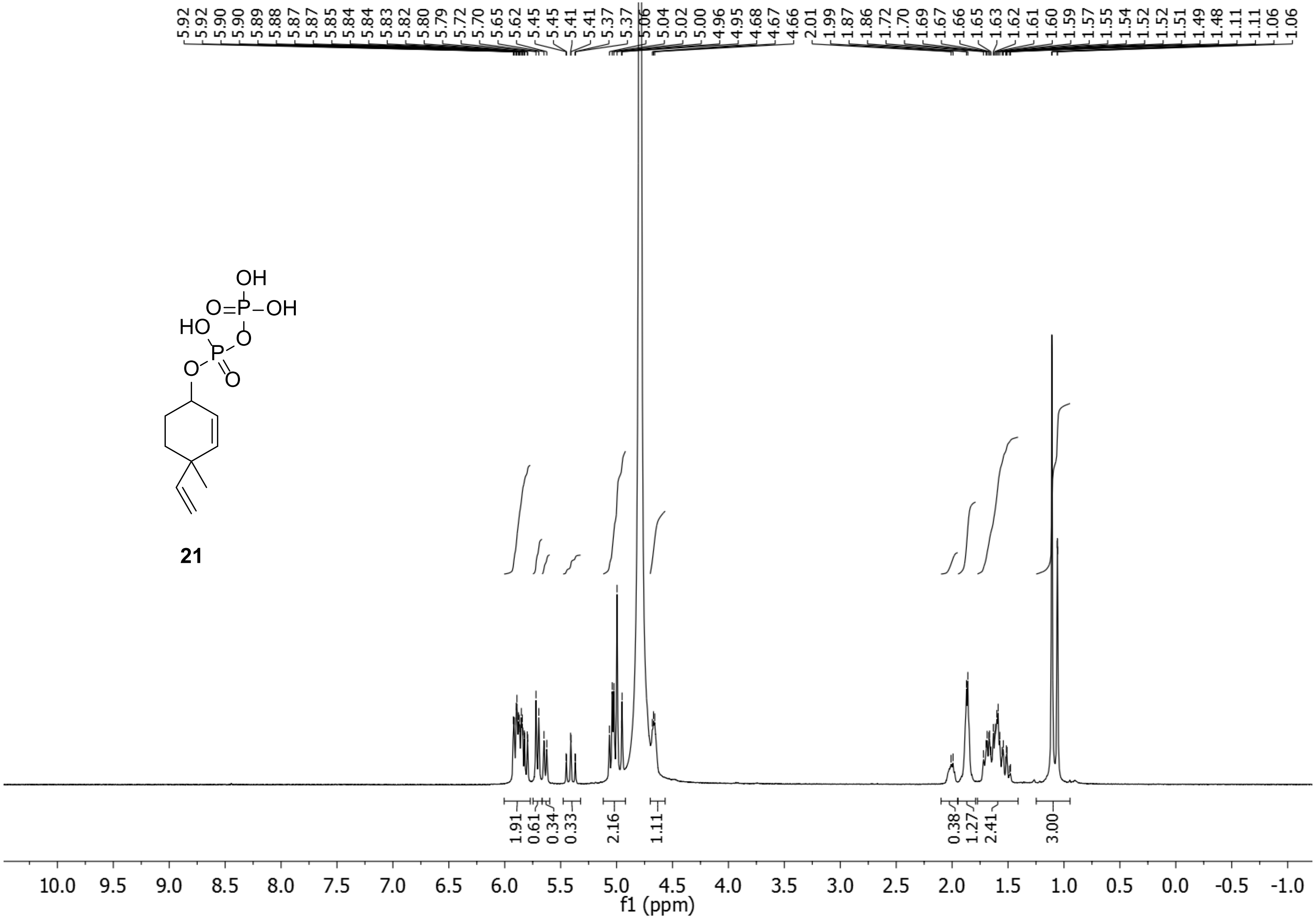

21

Figure S120 ^{1}H NMR (400 MHz) spectrum of 4-methyl-4-vinylcyclohex-2-en-1-yl trihydrogen diphosphate (**21**) in D_2O.

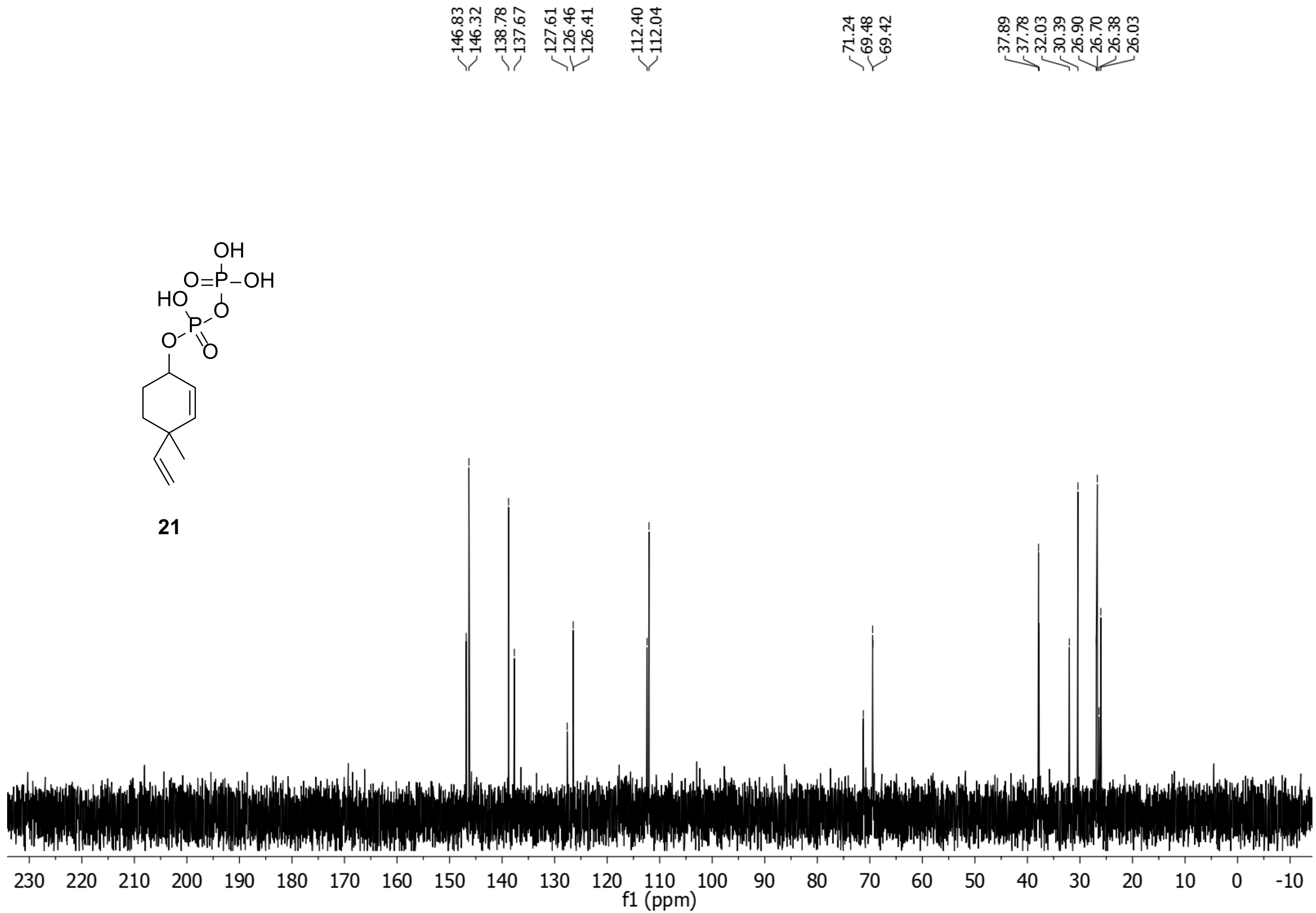

Figure S121 ^{13}C NMR (101 MHz) spectrum of 4-methyl-4-vinylcyclohex-2-en-1-yl trihydrogen diphosphate (**21**) in D_2O.

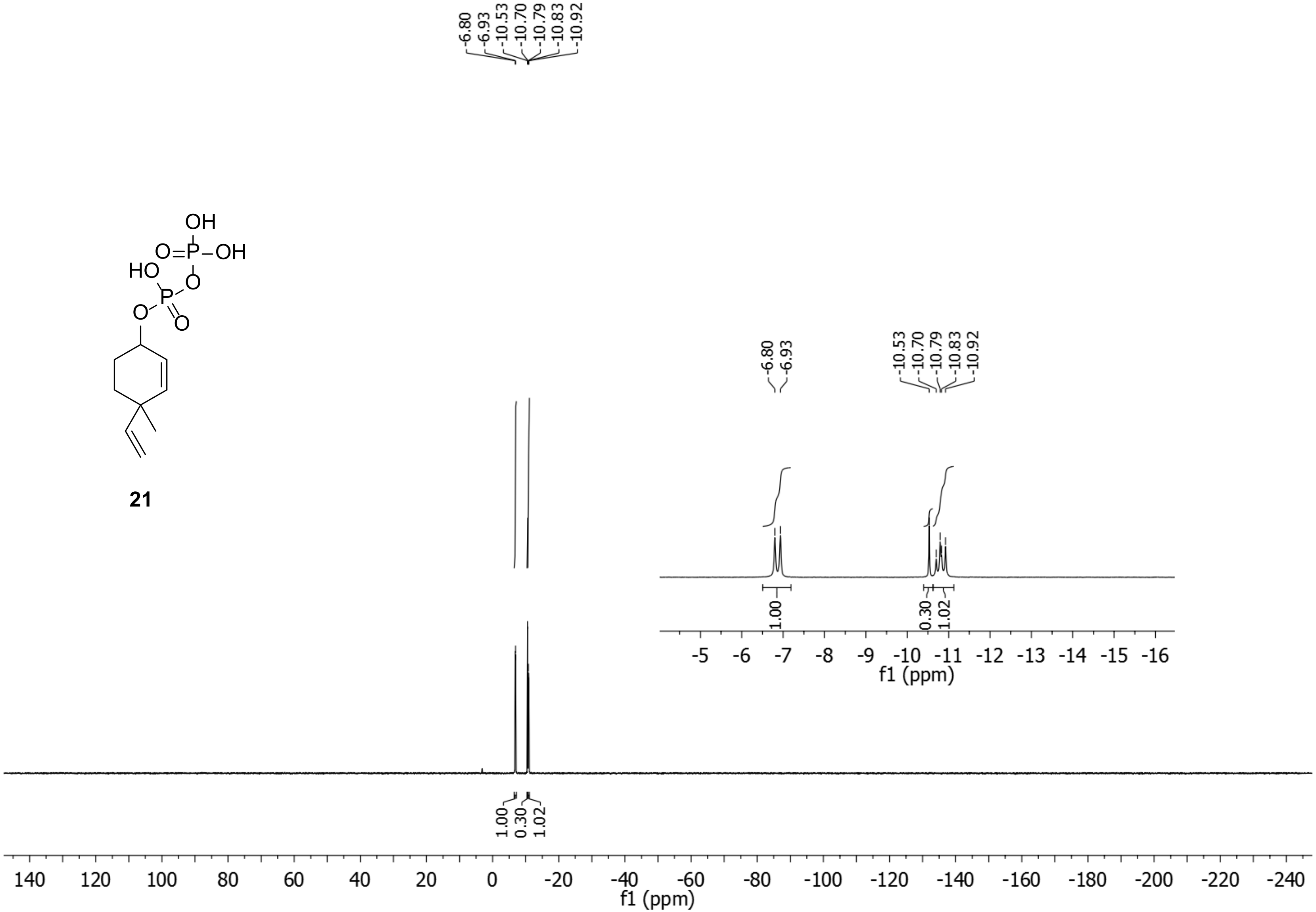

Figure S122 ^{31}P NMR (162 MHz) spectrum of 4-methyl-4-vinylcyclohex-2-en-1-yl trihydrogen diphosphate (**21**) in D_2O.

`

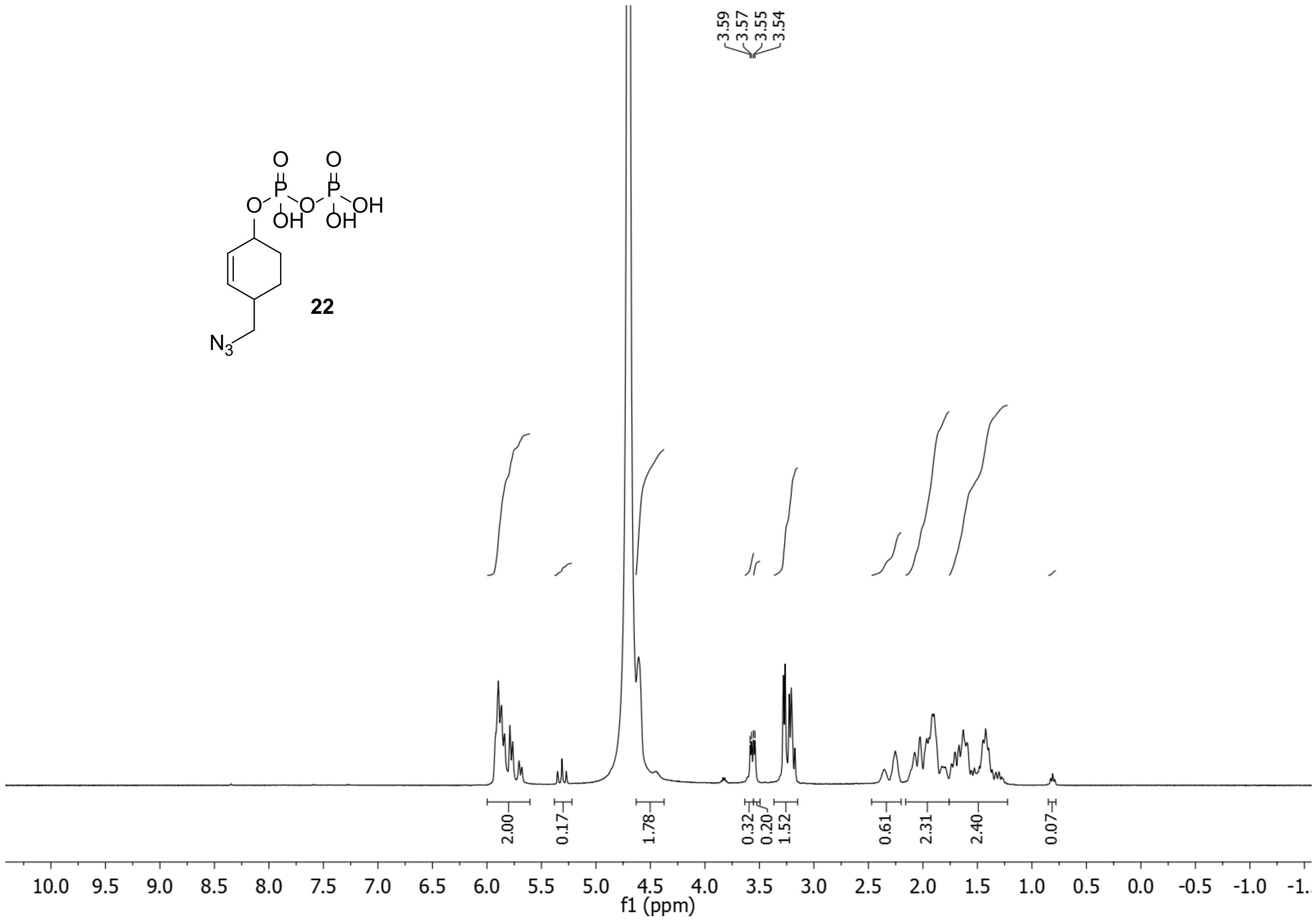

Figure S123 ^{1}H NMR (400 MHz) spectrum of 4-(azidomethyl)cyclohex-2-en-1-yl trihydrogen diphosphate (**22**) in D_2O.

`

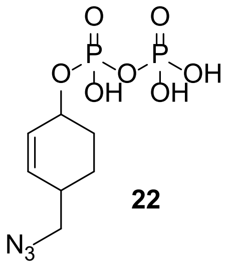

22

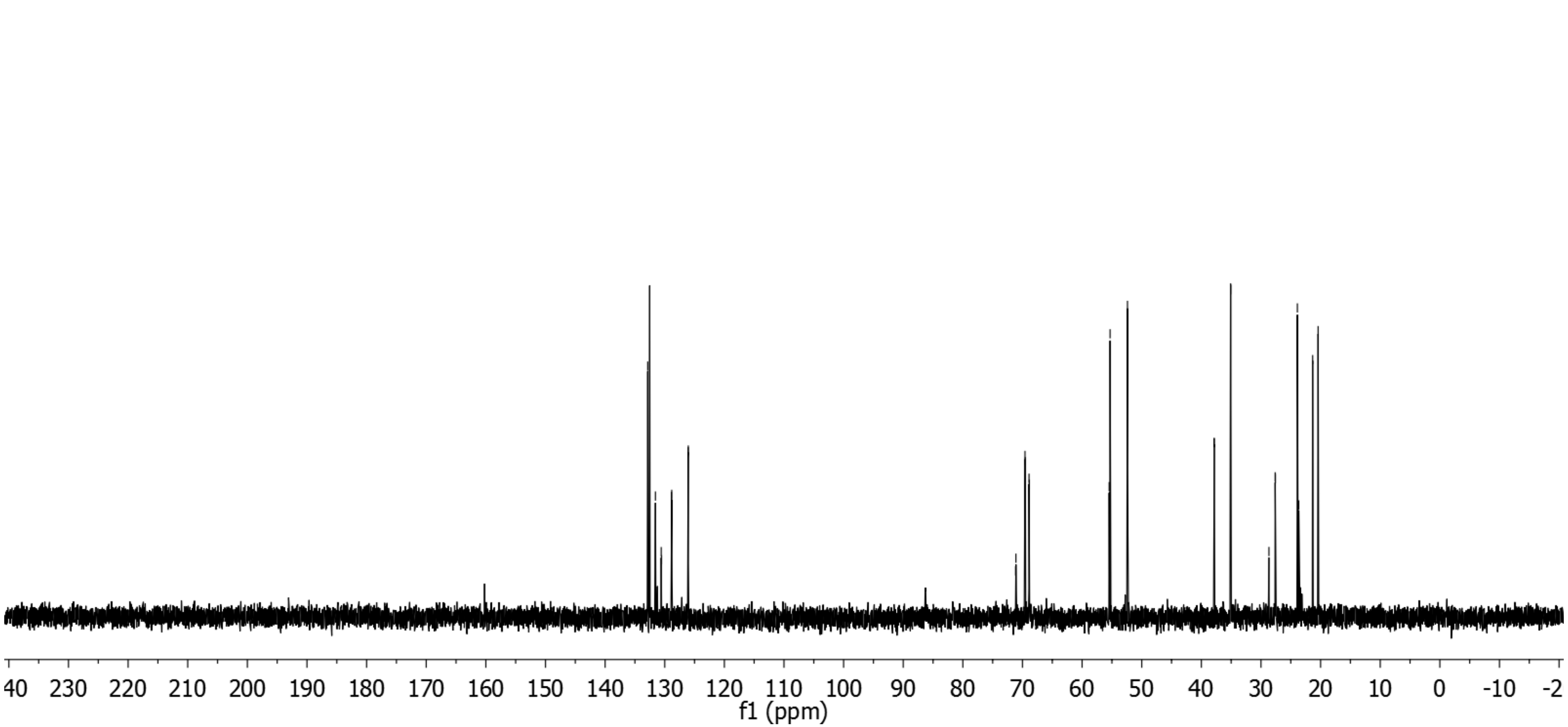

Figure S124 ^{13}C NMR (101 MHz) spectrum of 4-(azidomethyl)cyclohex-2-en-1-yl trihydrogen diphosphate (**22**) in D_2O.

`

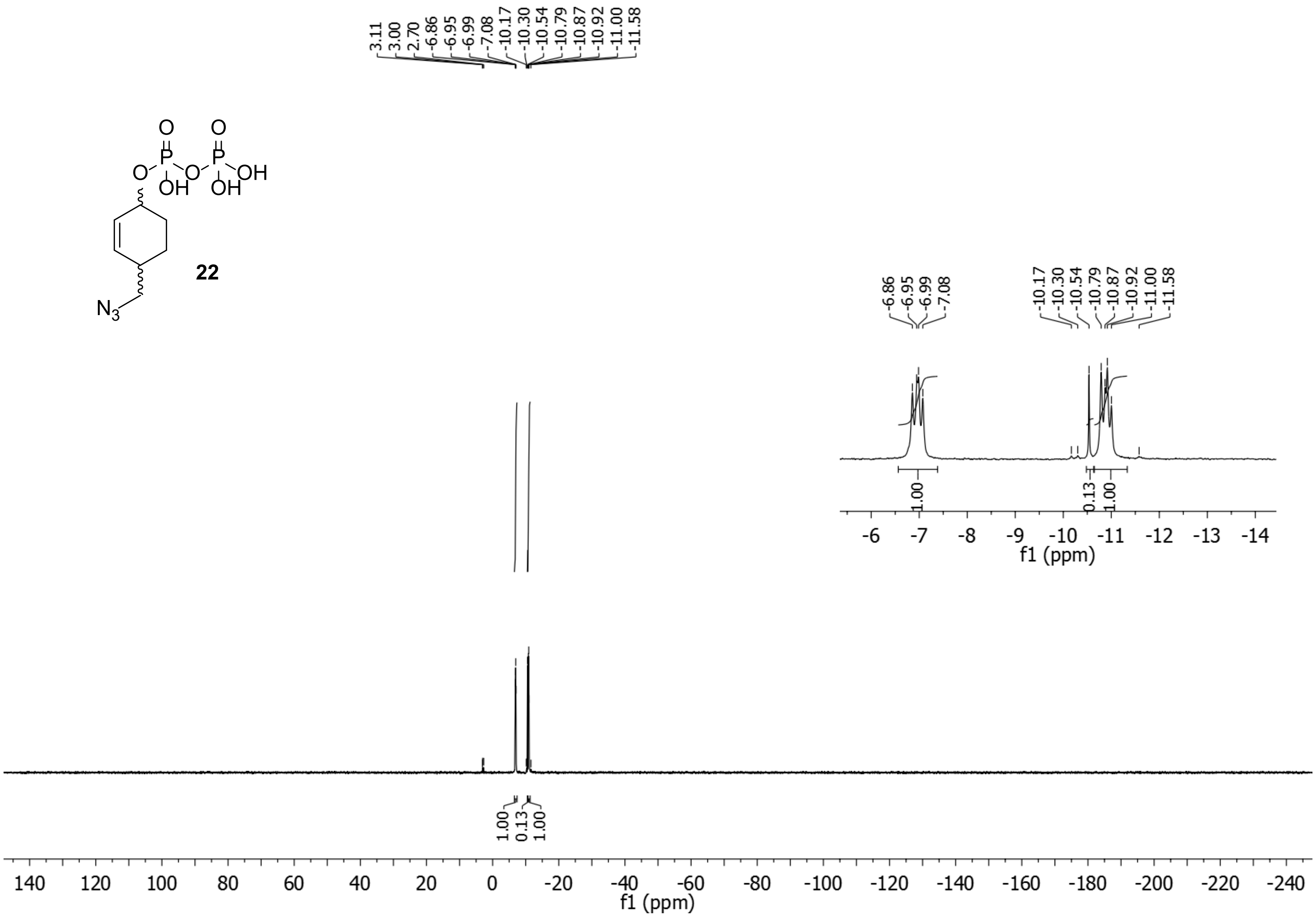

Figure S125 ^{31}P NMR (162 MHz) spectrum of 4-(azidomethyl)cyclohex-2-en-1-yl trihydrogen diphosphate (**22**) in D_2O.

`

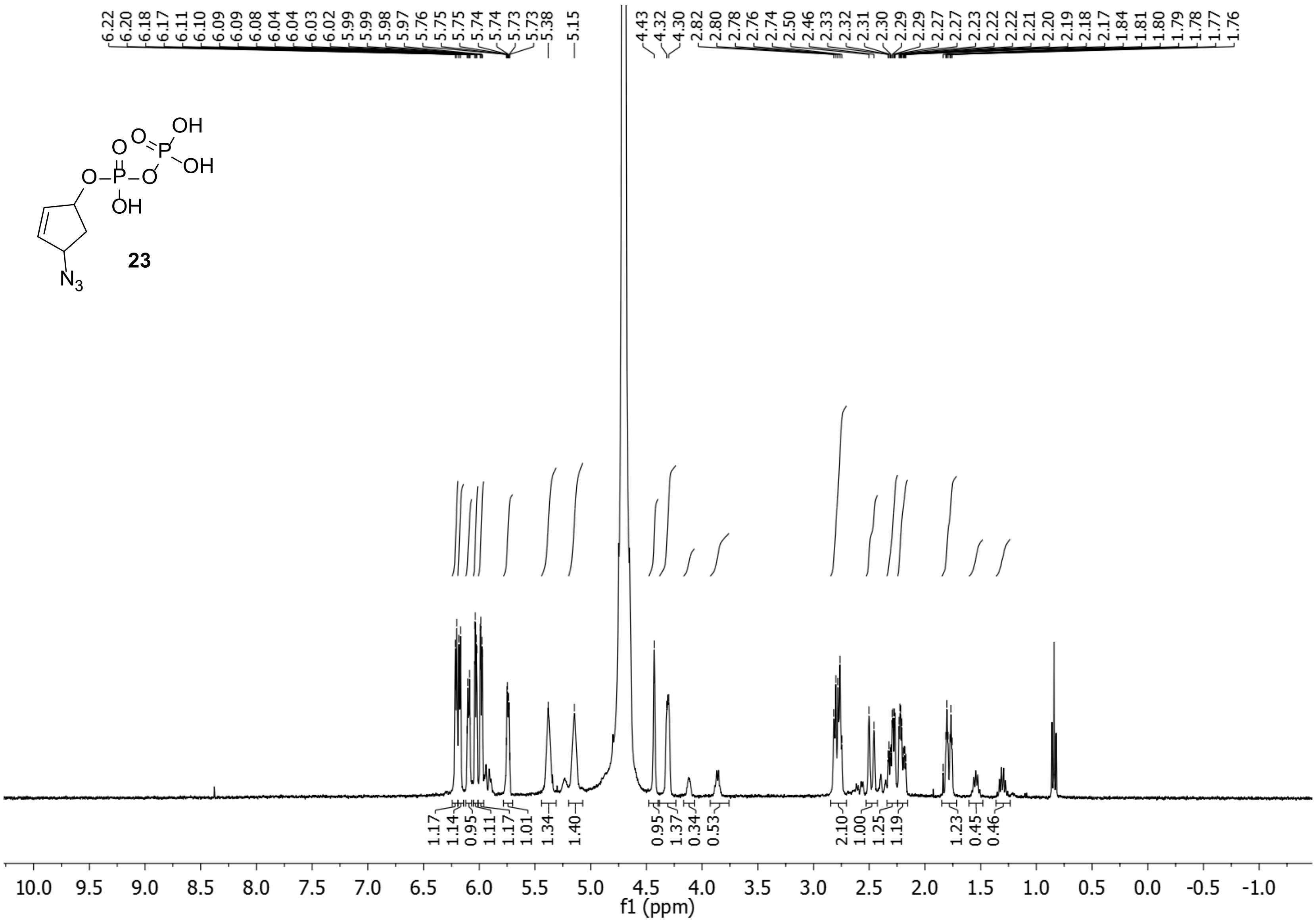

23

Figure S126 ^{1}H NMR (400 MHz) spectrum of 4-azidocyclopent-2-en-1-yl trihydrogen diphosphate (23) in D_2O.

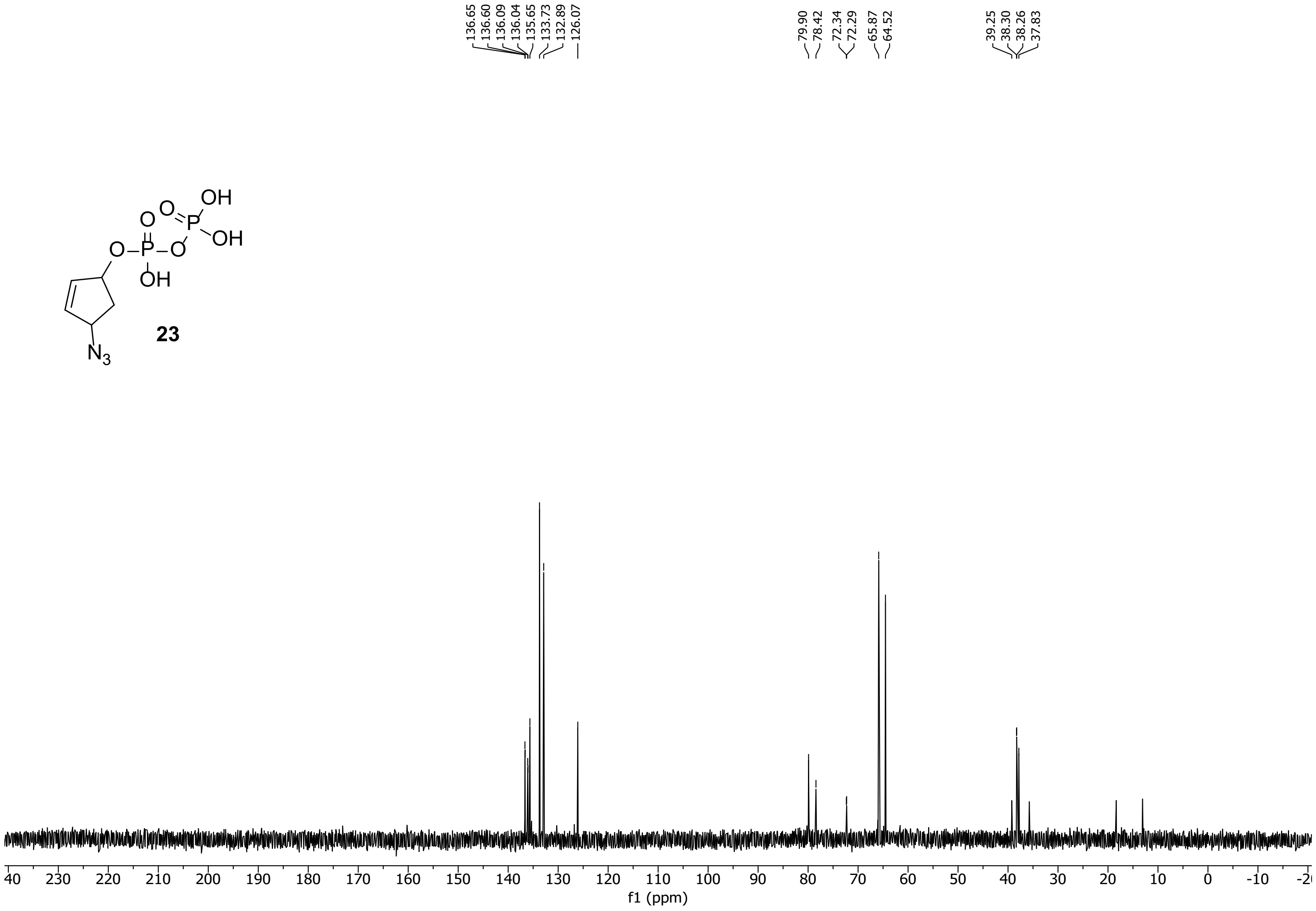

Figure S127 ^{13}C NMR (101 MHz) spectrum of 4-azidocyclopent-2-en-1-yl trihydrogen diphosphate (**23**) in D_2O.

`

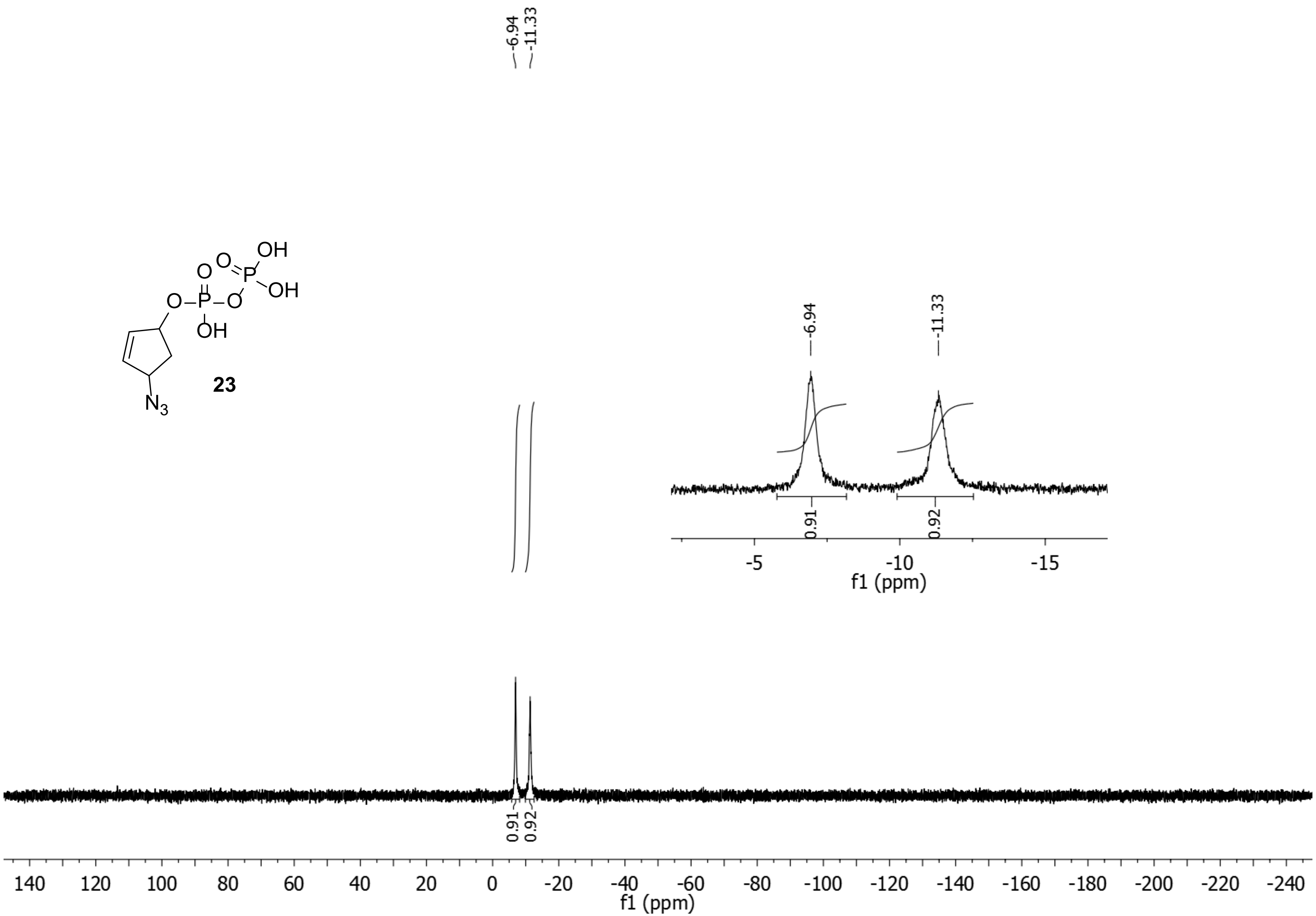

23

Figure S128 ^{31}P NMR (162 MHz) spectrum of 4-azidocyclopent-2-en-1-yl trihydrogen diphosphate (**23**) in D_2O.

`

5 NMR spectra of new intermediates

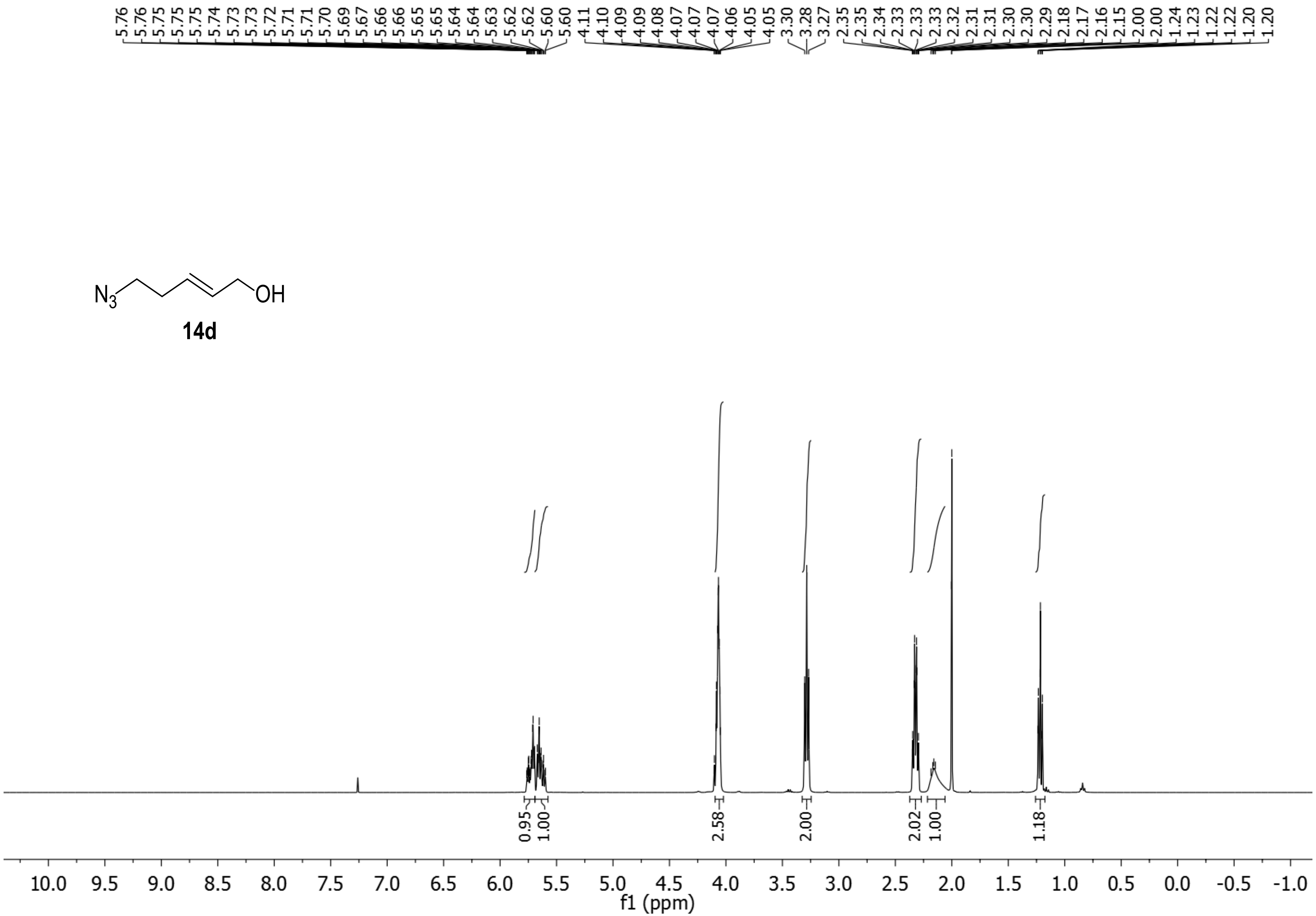

Figure S129 ^{1}H NMR (400 MHz) spectrum of (*E*)-5-azidopent-2-en-1-ol (**14d**) in $CDCl_3$.

`

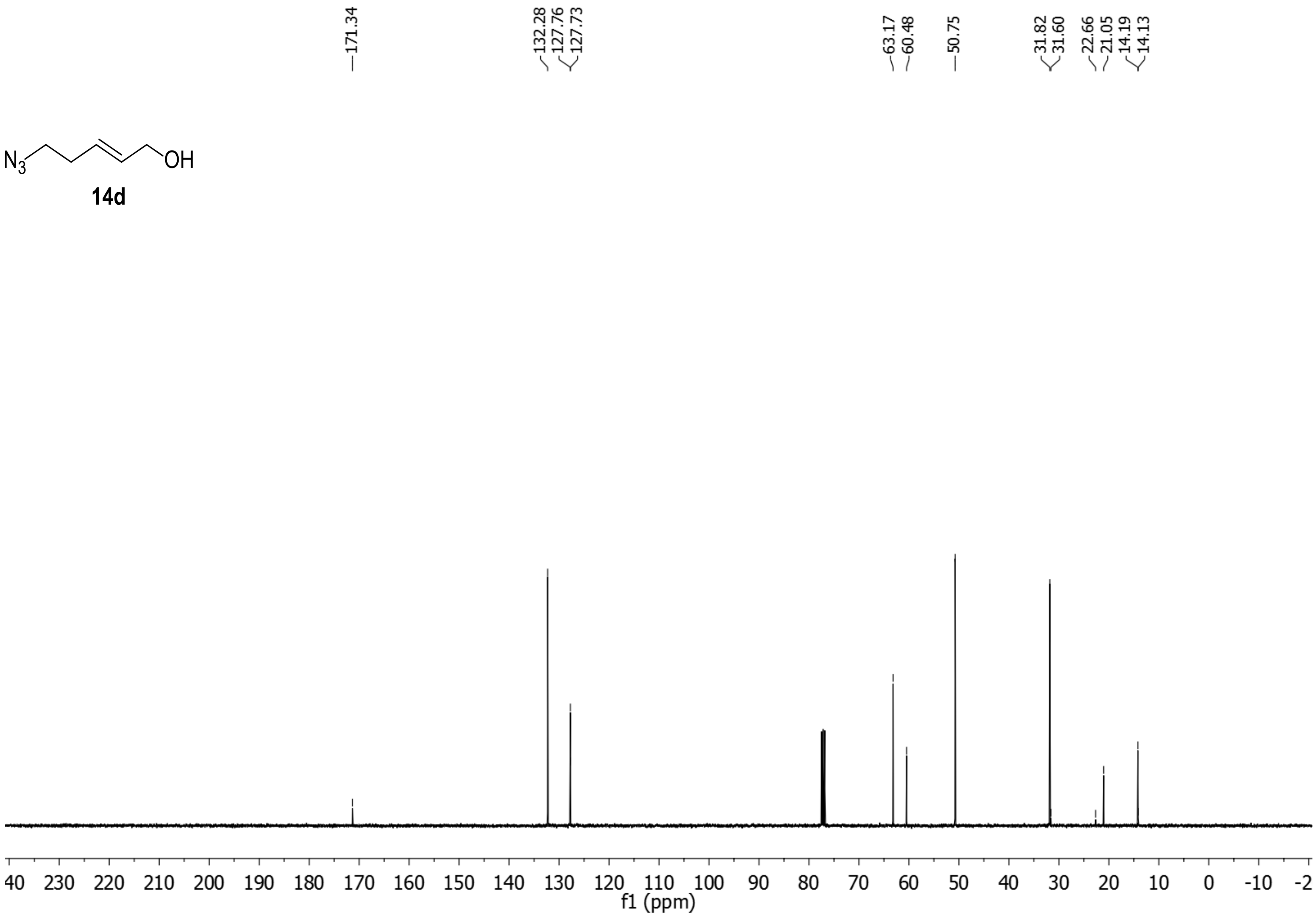

14d

Figure S130 ^{13}C NMR (101 MHz) spectrum of (*E*)-5-azidopent-2-en-1-ol (**14d**) in $CDCl_3$.

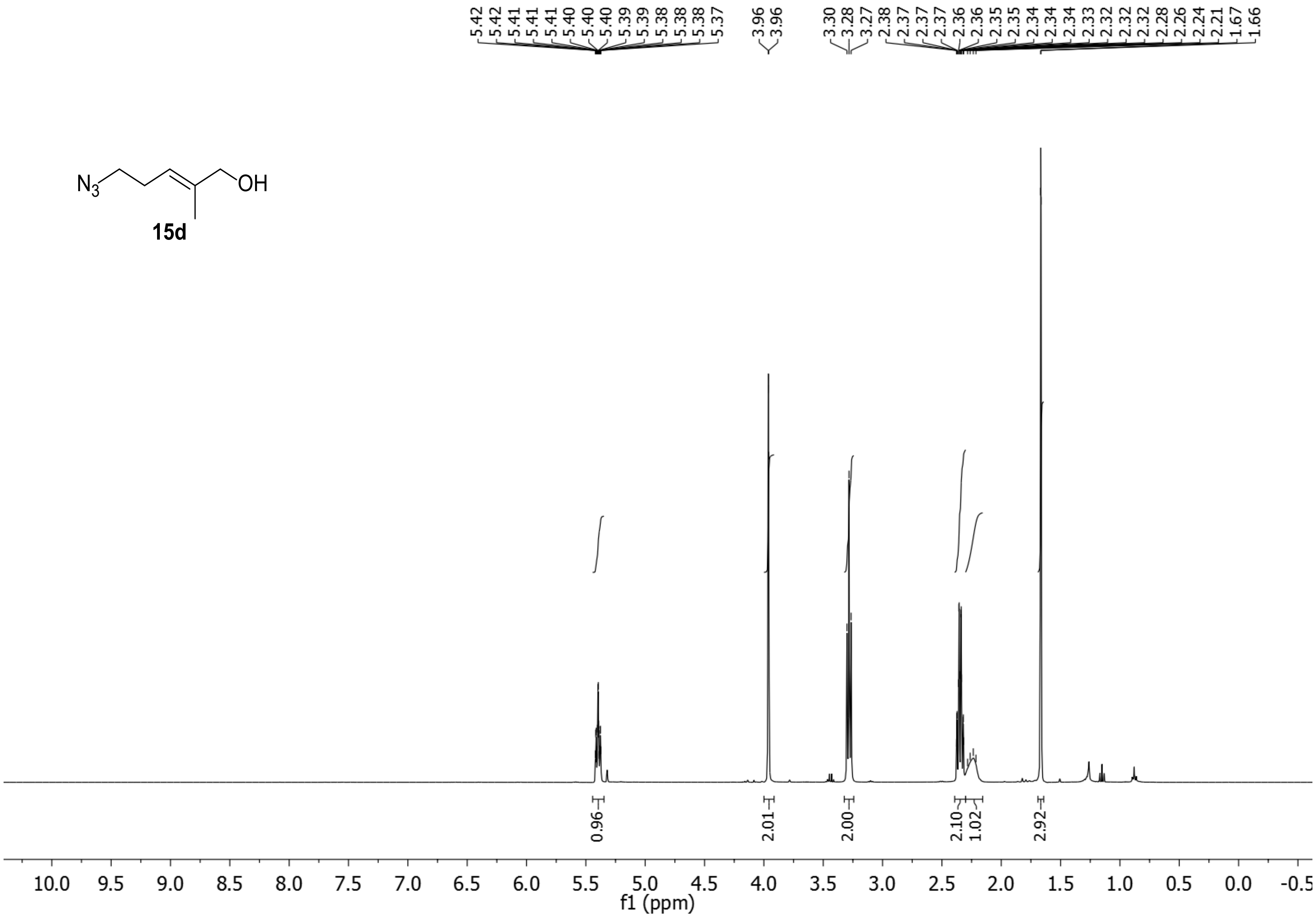

Figure S131 ^{1}H NMR (400 MHz) spectrum of (*E*)-5-azido-2-methylpent-2-en-1-ol (**15d**) in CD_2Cl_2.

`

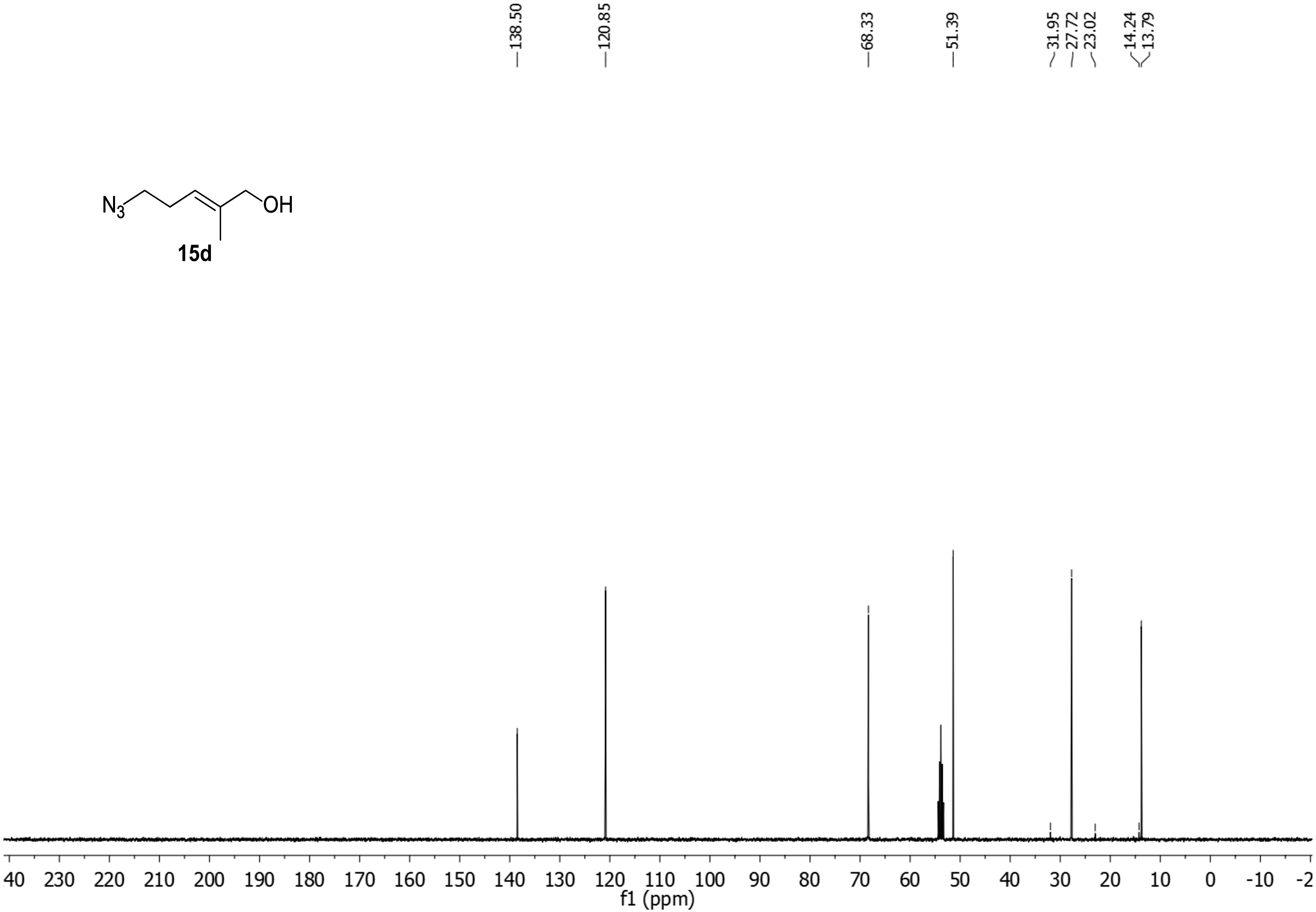

Figure S132 ^{13}C NMR (101 MHz) spectrum of (*E*)-5-azido-2-methylpent-2-en-1-ol (**15d**) in CD_2Cl_2.

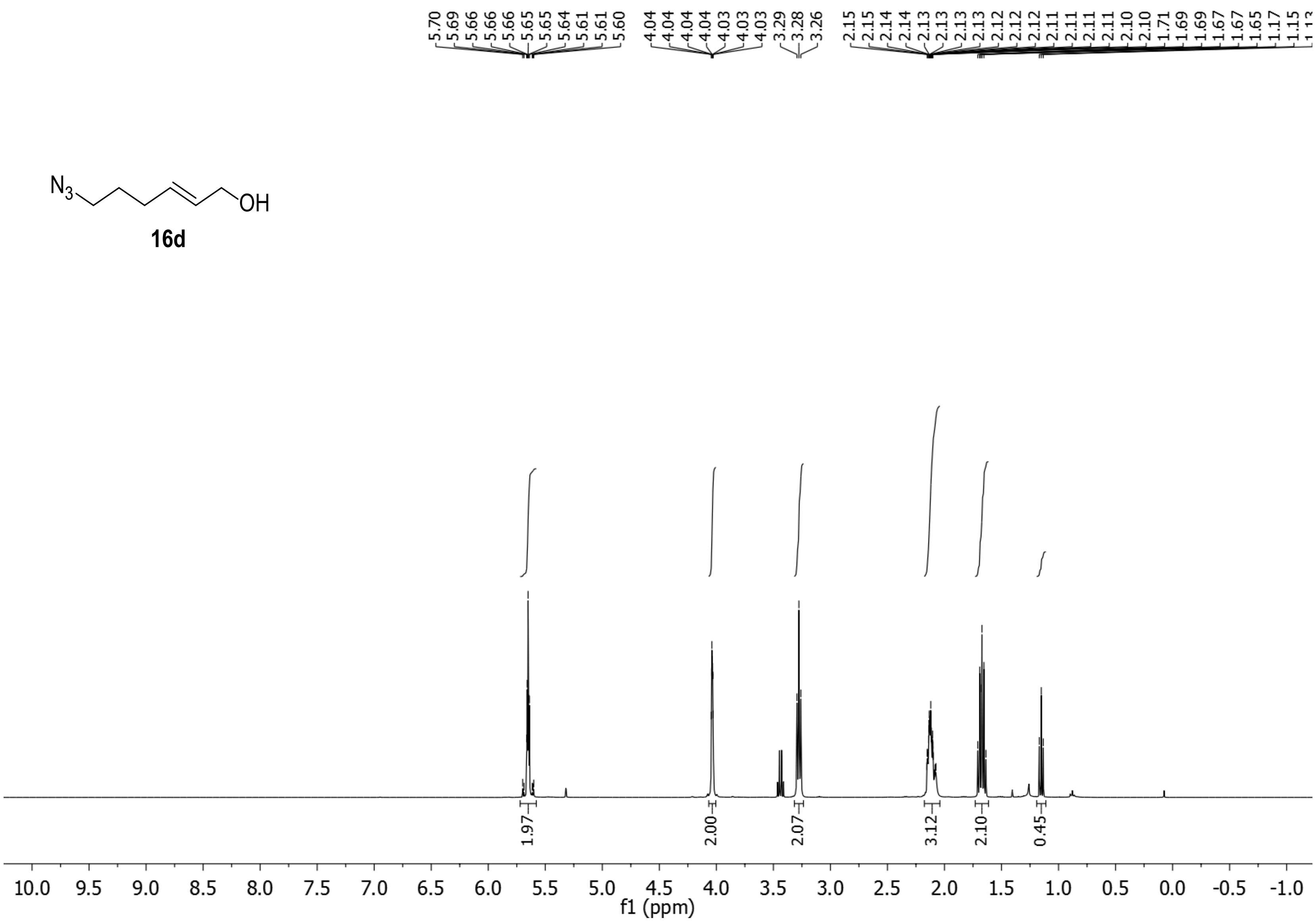

Figure S133 ^{1}H NMR (400 MHz) spectrum of (*E*)-6-azidohex-2-en-1-ol (**16d**) in CD_2Cl_2.

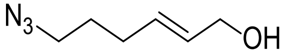

16d

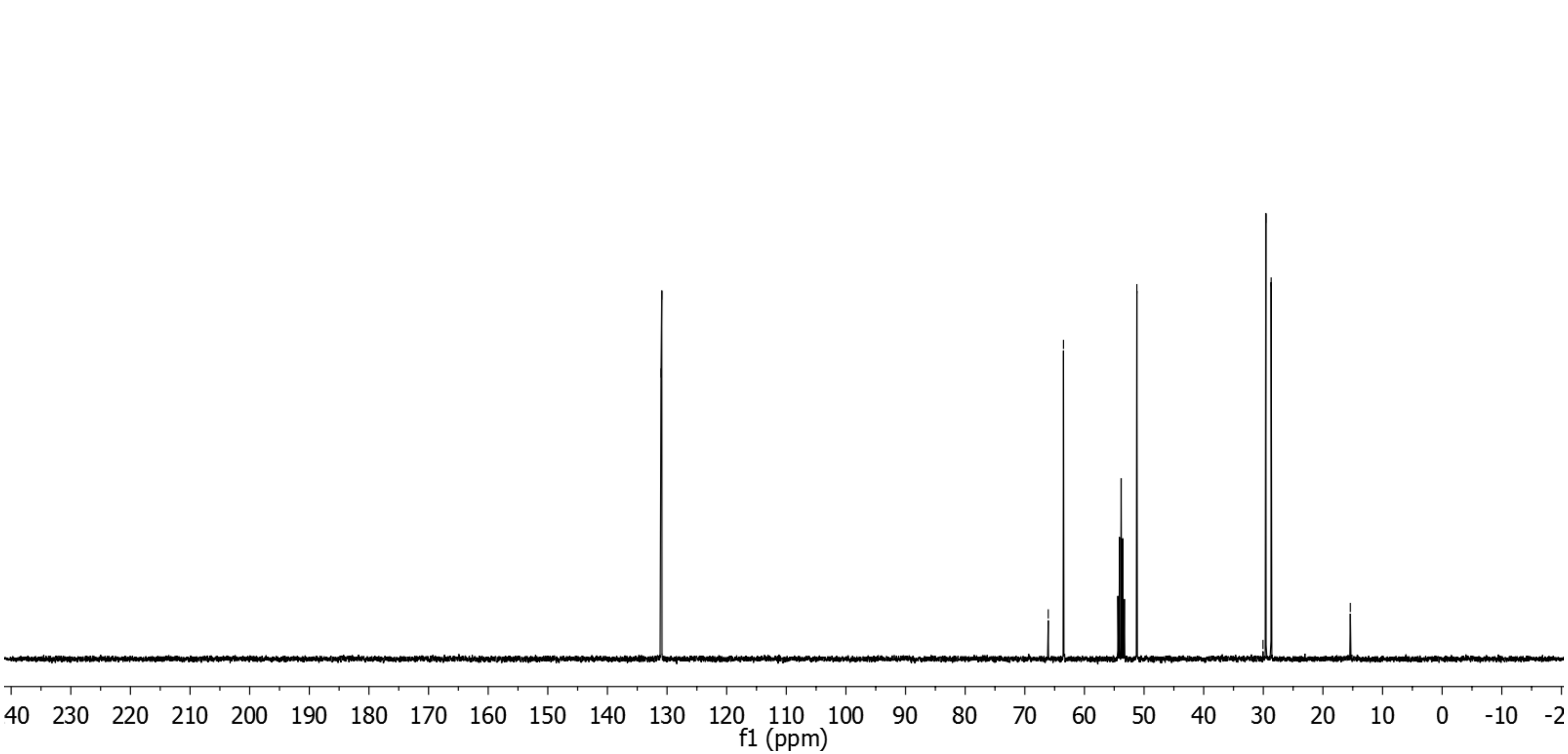

Figure S134 ^{13}C NMR (101 MHz) spectrum of (*E*)-6-azidohex-2-en-1-ol (**16d**) in CD_2Cl_2

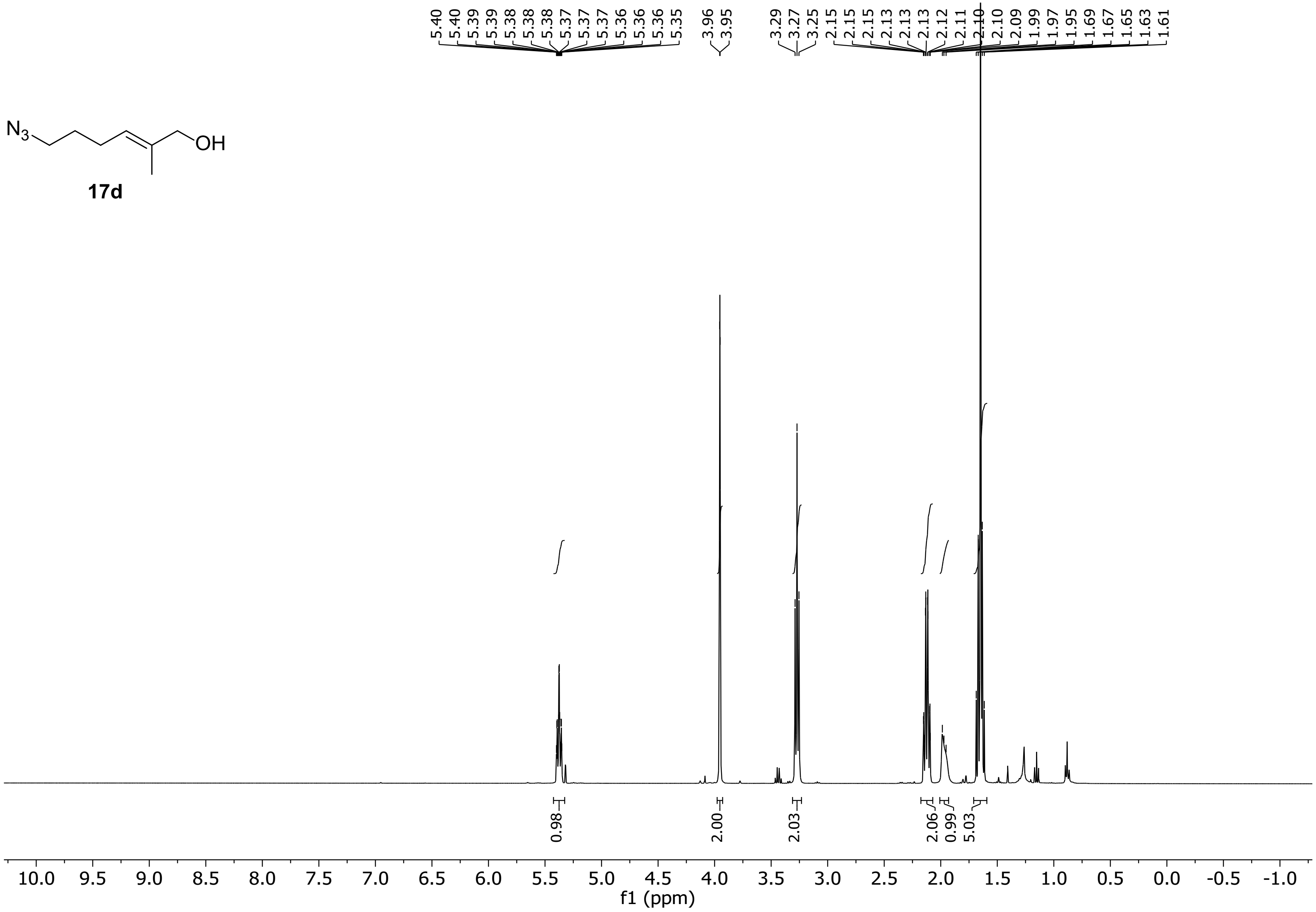

Figure S135 ^{1}H NMR (400 MHz) spectrum of (*E*)-6-azido-2-methylhex-2-en-1-ol (**17d**) in CD_2Cl_2.

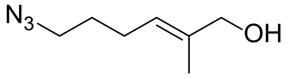

17d

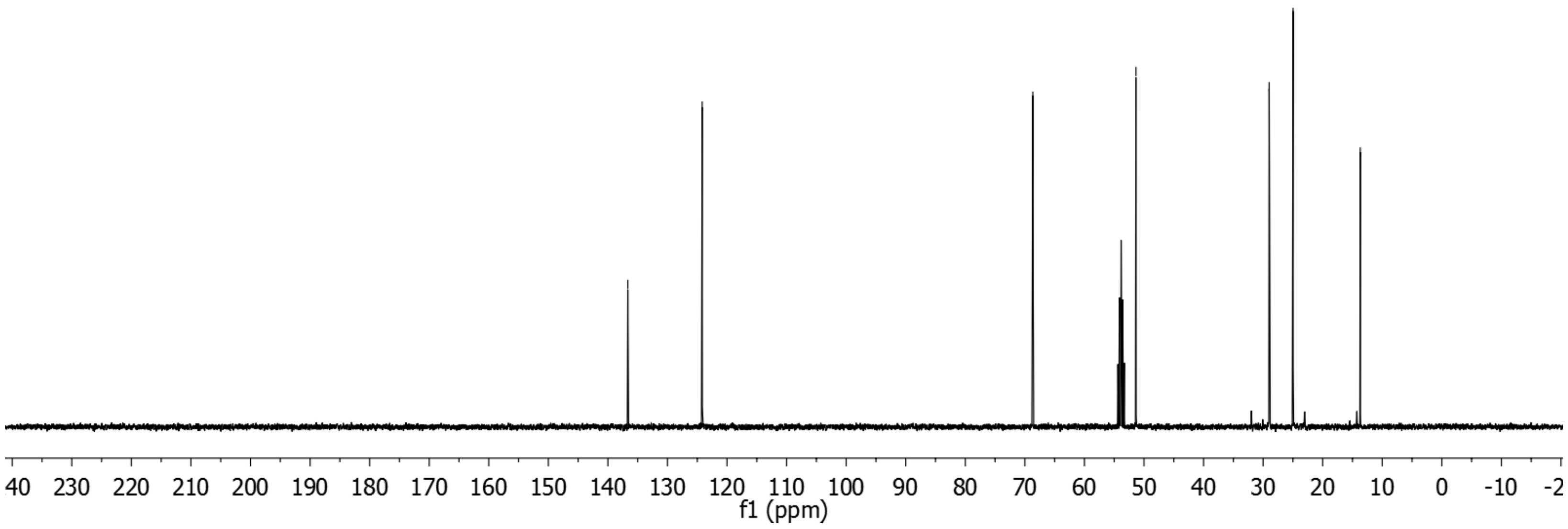

Figure S136 ^{13}C NMR (101 MHz) spectrum of (*E*)-6-azido-2-methylhex-2-en-1-ol (**17d**) in CD_2Cl_2.

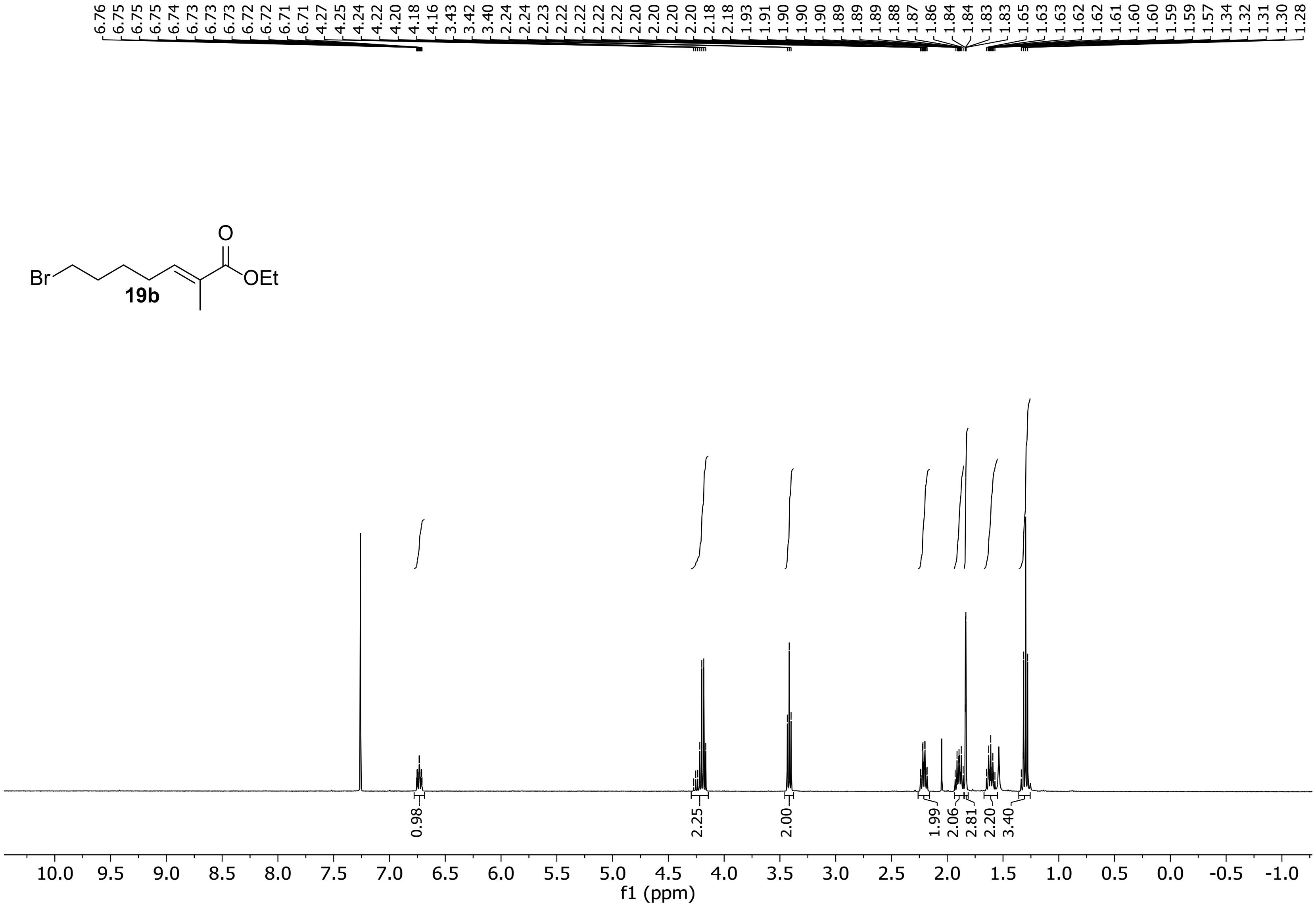

Figure S137 ^{1}H NMR (400 MHz) spectrum of ethyl (*E*)-7-bromo-2-methylhept-2-enoate (**19b**) in $CDCl_3$.

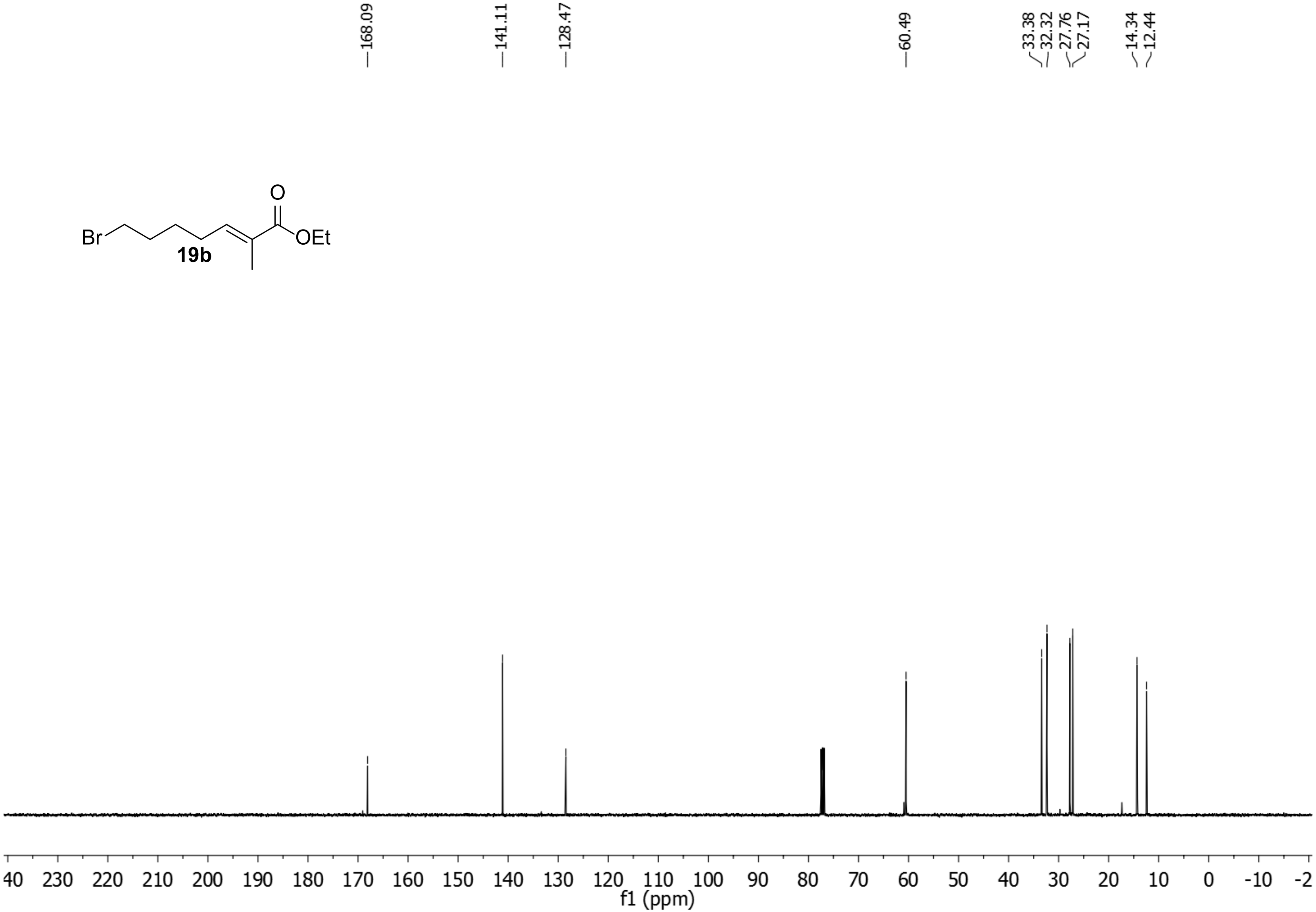

Figure S138 ^{13}C NMR (101 MHz) spectrum of ethyl (*E*)-7-bromo-2-methylhept-2-enoate (**19b**) in $CDCl_3$.

`

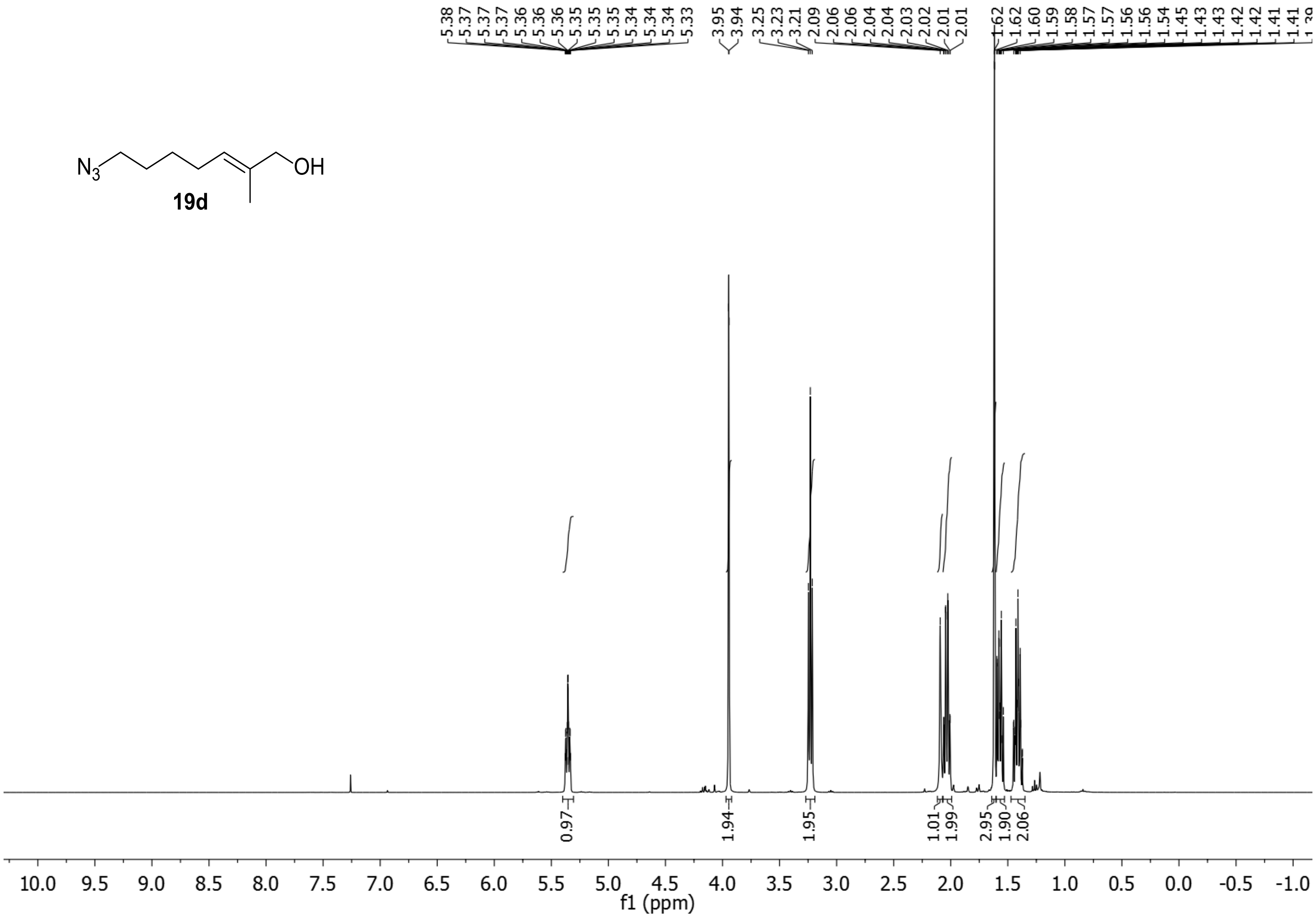

Figure S139 ^{1}H NMR (400 MHz) spectrum of (*E*)-7-azido-2-methylhept-2-en-1-ol (**19d**) in $CDCl_3$.

`

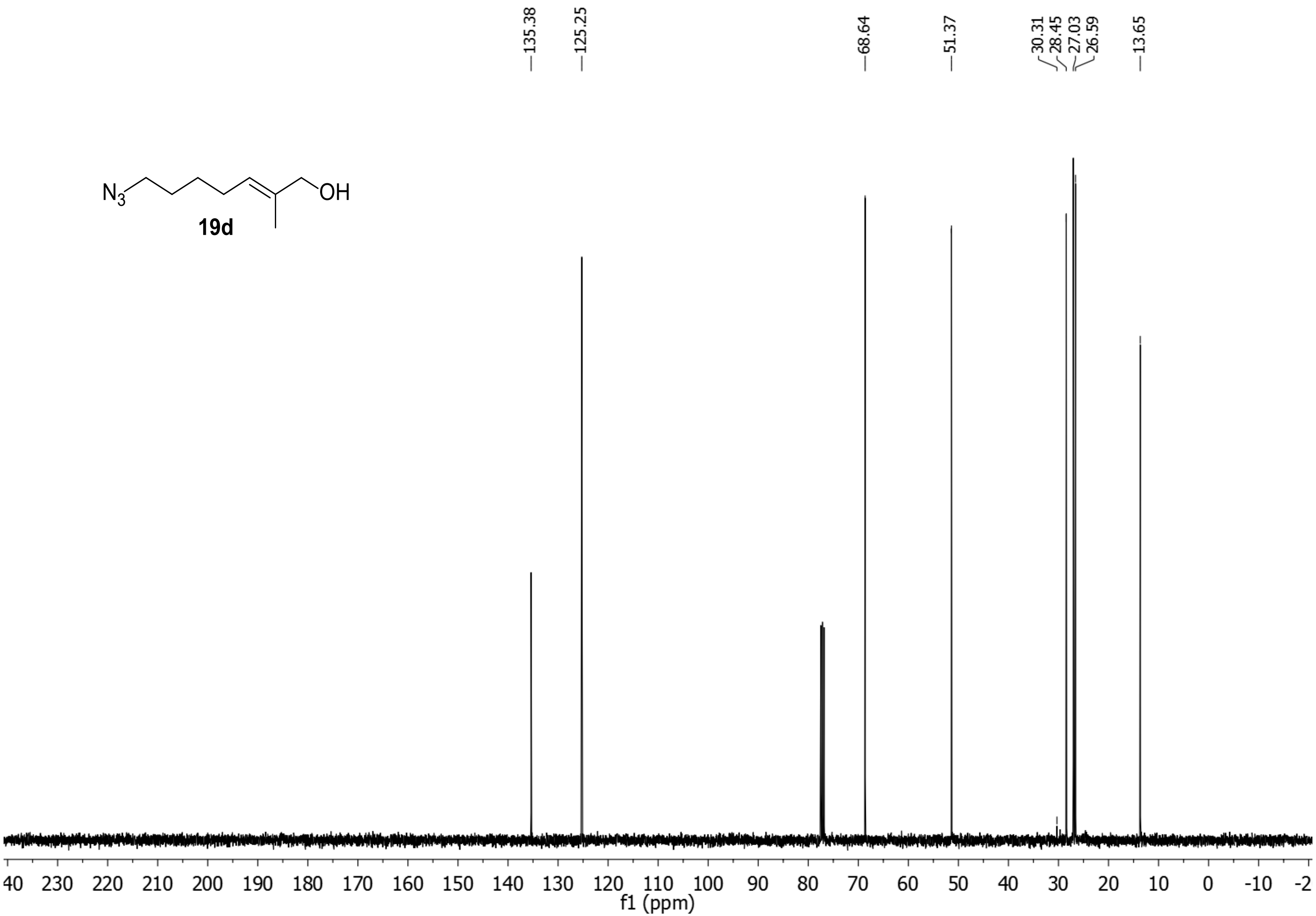

Figure S140 ^{13}C NMR (101 MHz) spectrum of (*E*)-7-azido-2-methylhept-2-en-1-ol (**19d**) in $CDCl_3$.

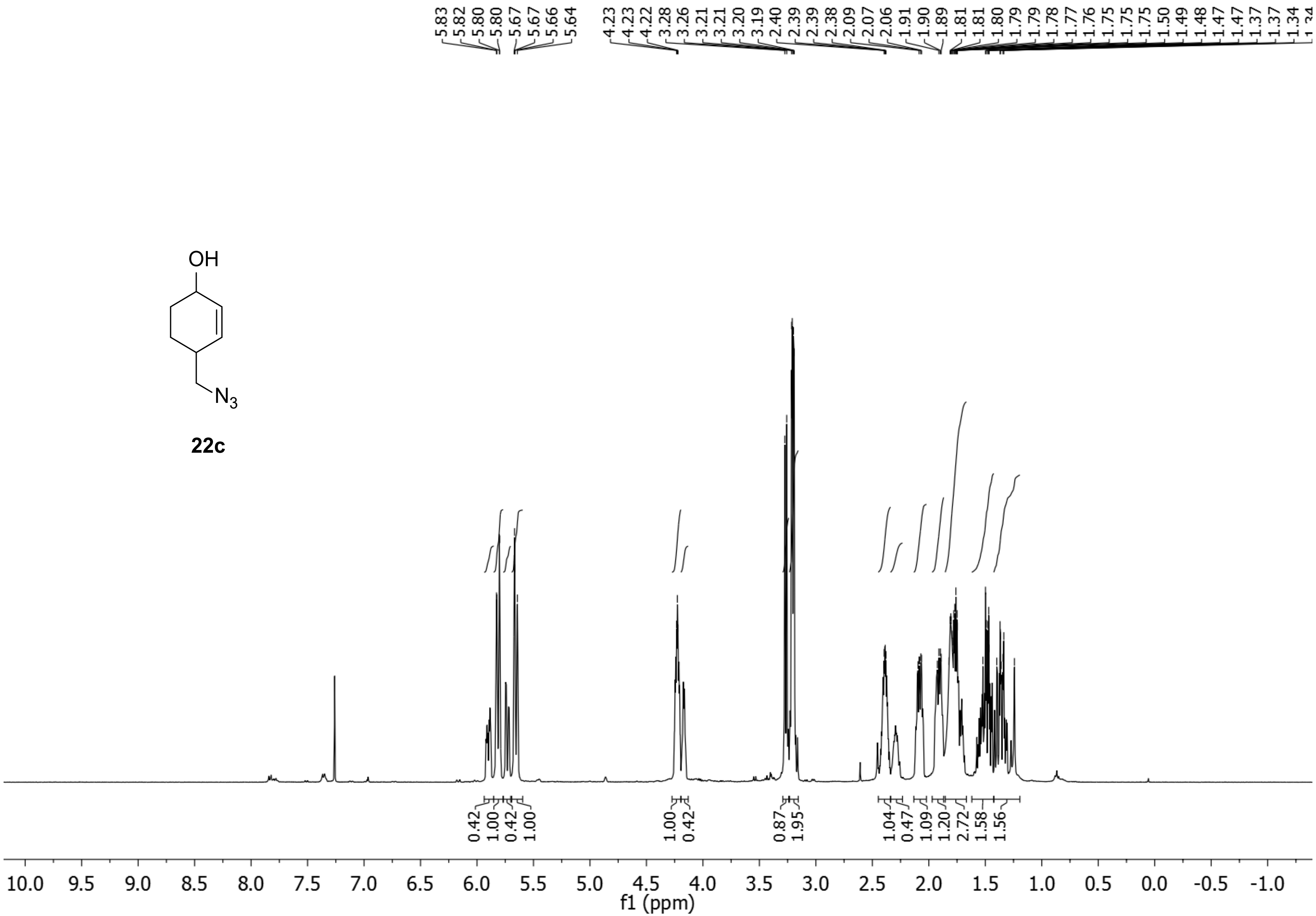

Figure S141 ^{1}H NMR (400 MHz) spectrum of 4-(azidomethyl)cyclohex-2-en-1-ol (**22c**) in $CDCl_3$.

`

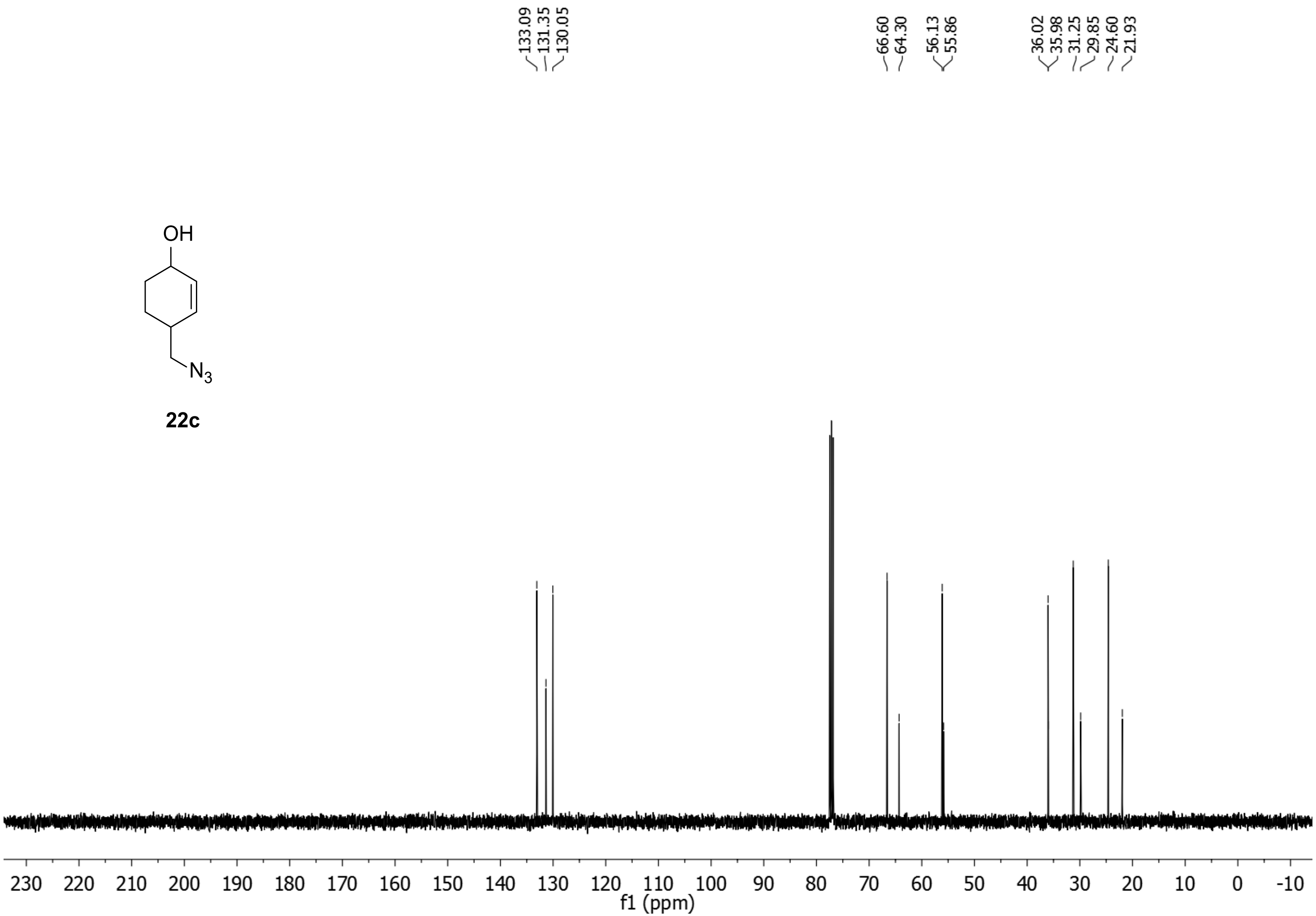

Figure S142 ^{13}C NMR (101 MHz) spectrum of 4-(azidomethyl)cyclohex-2-en-1-ol (**22c**) in $CDCl_3$.
